# A Bioinformatics Approach to Investigate Structural and Non-Structural Proteins in Human Coronaviruses

**DOI:** 10.3389/fgene.2022.891418

**Published:** 2022-06-14

**Authors:** Vittoria Cicaloni, Filippo Costanti, Arianna Pasqui, Monica Bianchini, Neri Niccolai, Pietro Bongini

**Affiliations:** ^1^ Toscana Life Science Foundation, Siena, Italy; ^2^ Department of Information Engineering and Mathematics, University of Siena, Siena, Italy; ^3^ Department of Biotechnology, Chemistry, and Pharmacy, University of Siena, Siena, Italy; ^4^ Department of Information Engineering, University of Florence, Firenze, Italy

**Keywords:** SARS-CoV-2, cross-reactivity, Siamese networks, long short-term memories, similarity score

## Abstract

Recent studies confirmed that people unexposed to SARS-CoV-2 have preexisting reactivity, probably due to previous exposure to widely circulating common cold coronaviruses. Such preexistent reactivity against SARS-CoV-2 comes from memory T cells that can specifically recognize a SARS-CoV-2 epitope of structural and non-structural proteins and the homologous epitopes from common cold coronaviruses. Therefore, it is important to understand the SARS-CoV-2 cross-reactivity by investigating these protein sequence similarities with those of different circulating coronaviruses. In addition, the emerging SARS-CoV-2 variants lead to an intense interest in whether mutations in proteins (especially in the spike) could potentially compromise vaccine effectiveness. Since it is not clear that the differences in clinical outcomes are caused by common cold coronaviruses, a deeper investigation on cross-reactive T-cell immunity to SARS-CoV-2 is crucial to examine the differential COVID-19 symptoms and vaccine performance. Therefore, the present study can be a starting point for further research on cross-reactive T cell recognition between circulating common cold coronaviruses and SARS-CoV-2, including the most recent variants Delta and Omicron. In the end, a deep learning approach, based on Siamese networks, is proposed to accurately and efficiently calculate a BLAST-like similarity score between protein sequences.

## Introduction

The severe acute respiratory syndrome coronavirus-2 (SARS-CoV-2) causes COVID-19 disease, which is often particularly severe in elderly patients associated with risk factors (Chen et al., 2020a). It was identified for the first time in January 2020 in patients suffering from pneumonia (Chen et al., 2020a); in the meantime, it has emerged as a pandemic pathogen causing more than 250 million confirmed cases and more than five million deaths worldwide (https://coronavirus.jhu.edu, assessed 19.11.21).

SARS-CoV-2 has a positive viral RNA genome expressing open-reading frames that code for structural and non-structural proteins (NSPs) (Zhong et al., 2005; Cui et al., 2019; Huang et al., 2020). The ORF1a and ORF1ab in the genomic RNA encode for various NSPs at the 5′ terminal and few structural proteins at the 3′ terminal. The structural ones include spike (S), nucleocapsid (N), membrane (M), and envelope (E) proteins (Raj, 2021). The S protein is characterized by different immunodominant sites (Zhou et al., 2005; Cao et al., 2010; Huang et al., 2020; Raj, 2021) and consists of two subunits: the S1 protein, containing the receptor-binding domain (RBD), and the S2 protein responsible for cell membrane fusion (Ren et al., 2003; Bisht et al., 2004). The N protein is also immunogenic and induces antibodies sooner than S after infection, a characteristic that makes it interesting for diagnostic assays (Meyer et al., 2014). Immunogenicity of other structural proteins has been less investigated: the relatively small M protein was found to have immunogenic epitopes, but titers generally increase later than 21 days after infection. Instead, responses to the E protein have been rarely detected so far. Conversely, in the study by Liu et al. (2007), anti–M antibodies were detected at 10 days post onset, while detection of anti-N and anti-S increased later. The translated polypeptides of ORF1ab are processed into approximately 1–15 NSPs with specific roles in the life cycle and pathogenicity of the virus (Raj, 2021).

**FIGURE 1:**
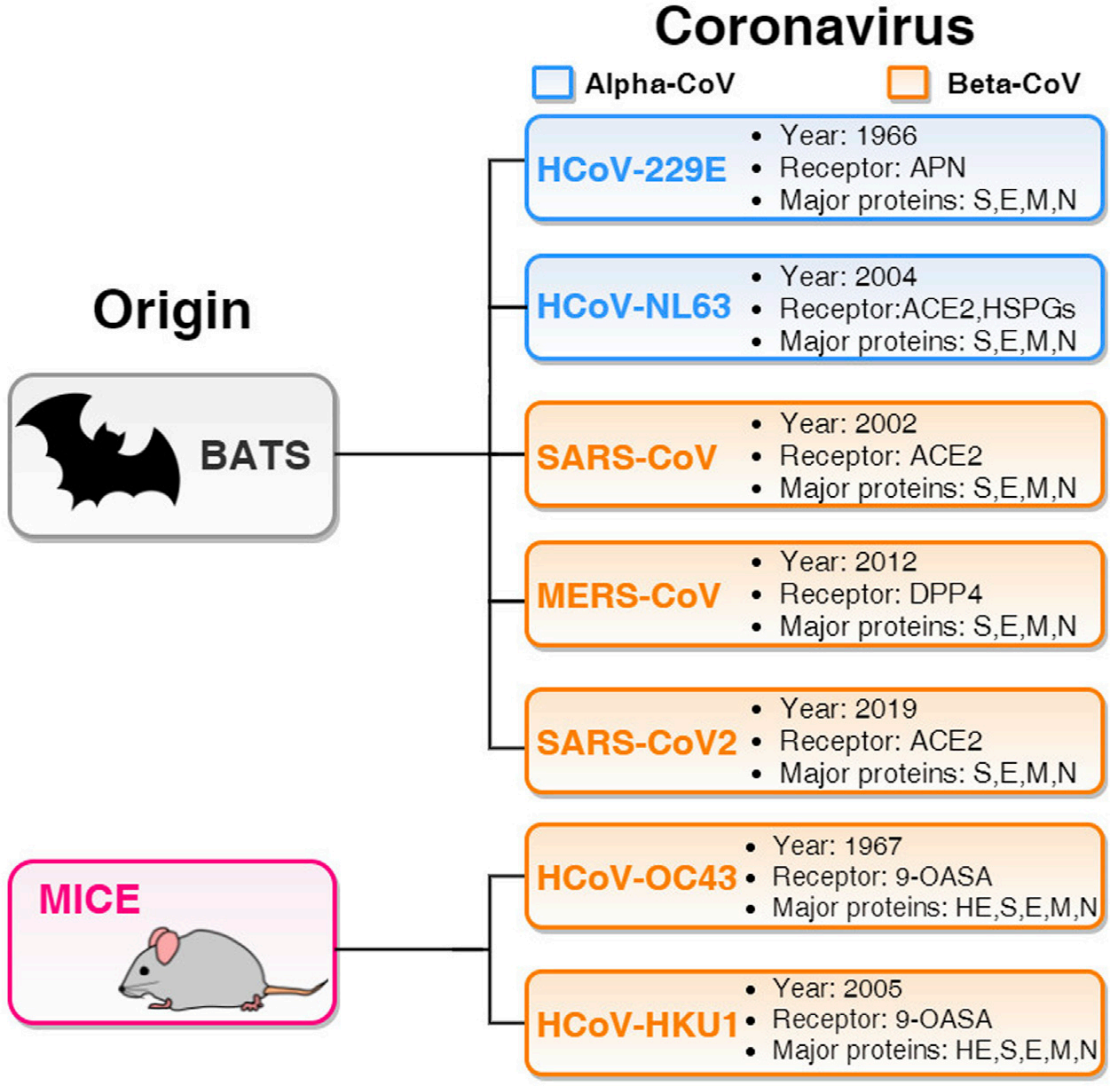
Basic features of seven HCoVs. The two blue panels list alpha-HCoVs and the five orange panels list beta-HCoVs. All the boxes include the year of identification, receptor, and major proteins. Two beta-HCoVs (HCoV-OC43 and HCoV-HKU1) originate in mice and contain hemagglutinin-esterase (HE) structural protein along with S, E, M, and N. Other five HCoVs originated in bats and have four major structural proteins S, E, M, and N.

Several studies have reported that some people unexposed to SARS-CoV-2 have preexisting reactivity to SARS-CoV-2 sequences (Mateus et al., 2020). The immunological mechanisms underlying this preexistent reactivity seem to be linked to previous exposure to widely circulating common cold coronaviruses (Mateus et al., 2020). Natural and experimental infection studies in humans investigated the cross-reactivity within distinct genera of coronaviruses (Huang et al., 2020). The Coronavirinae subfamily includes four distinct genera: the alpha- and beta-CoV family, which usually infects mammals and humans, and the gamma- and delta-CoV family, which generally infects birds. From the 1960s to the present time, seven coronaviruses have been documented to generate infection in humans (Ahsan et al., 2021). Among such seven human coronaviruses (HCoVs), two HCoVs (229E and NL63) belong to the alpha-HCoVs, whereas the other five (OC43, SARS, HKU1, MERS, and SARS-2) are beta-HCoVs (Ahsan et al., 2021). The natural hosts for most of the HCoVs are bats (Lau et al., 2020), with the only exception of HCoV-OC43 and HCoV-HKU1, which originated in mice (Cui et al., 2019). The bat-originated HCoVs include the four structural proteins mentioned above, while in mice-originated HCoVs, one more structural protein called hemagglutinin-esterase (HE) is observed. However, it is not entirely clear that the direct origin of SARS-CoV-2 is from the bat. For instance, some studies suggested the pangolin as a missing link between bats and humans (Zhang et al., 2020). For entering into the host cell and triggering infection, several human proteins assume the role of viral receptors: angiotensin-converting enzyme 2 (ACE2) for SARS-CoV (Li et al., 2003), SARS-CoV-2 (Hoffmann et al., 2020), and HCoV-NL63 (Hofmann et al., 2005); aminopeptidase N (APN) and dipeptidyl peptidase 4 (DPP4) are entry receptors for HCoV-229E and MERS-CoV, respectively; the mice-originated beta-CoVs HCoV-OC43 and HCoV-HKU1 need 9-O-acetylated sialic acid (9-OASA) as a viral receptor (Cui et al., 2019). The summary of these features is reported in Figure 1.

As the circulating common cold HCoVs (i.e., OC43 and HKU1) share some homologous sequences with SARS-CoV-2, HCoV cross-reactive T-cell responses could influence the susceptibility to SARS-CoV-2 infection and the course of COVID-19 (Mateus et al., 2020). In the present study, we have better investigated the genome similarities among the HCoVs. Indeed, in the study by Mateus et al., (2020), it was found that the preexisting reactivity against SARS-CoV-2 comes from memory T cells and cross-reactive T cells can specifically recognize a SARS-CoV-2 epitope and the homologous epitope from circulating common cold HCoVs. Specifically, CD4^+^ T cell responses to S protein, the most important target for vaccine development, were strongly correlated with the magnitude of the anti-SARS-CoV-2 IgG and IgA titers (Gussow et al., 2020a). However, as reported (Grifoni et al., 2020; Kundu et al., 2022), not only S protein but also M and N proteins accounted for 11%–27% of the total CD4^+^ response, with additional significant responses directed against nsp3, nsp4, nsp12, ORF3a, and ORF8. Regarding SARS-CoV-2 CD8^+^ T cell responses, S and M were both recognized and significant reactivity was also noted for nsp6, ORF3a, and N, which comprised nearly 50% of the total CD8^+^ T cell response. These findings underline the importance of determining the impacts of preexisting immune memory in COVID-19 disease severity, particularly to better and quickly investigate the emerging variants that may have an impact on the virus transmissibility and pathogenicity. Computational studies could be effective to examine the most recent Delta and Omicron variants. For instance, in a recent study (Kumar et al., 2021), it has been found that the Omicron S protein has a higher affinity for human angiotensin-converting enzyme 2 (ACE2) than the Delta variant due to a significant number of mutations in the SARS-CoV-2 receptor-binding domain (RBD), indicating higher potential for transmission. In this context, future efforts are needed to investigate the epidemiological and biological consequences of the variants. Starting with this assumption, we investigated the similarities among the S protein sequences of the SARS-CoV-2 (including wild type, Omicron, and Delta) and the other HCoVs, with particular attention to S proteins, to better understand the cross-reactivity of SARS-CoV-2 and other circulating HCoVs. This is an important starting point for exploring the pattern of immunodominance in COVID-19, for interpreting its pathogenesis, and for the calibration of pandemic control measures. This is why we performed systematic sequence alignments on structural proteins and NSPs among SARS-CoV-2 and all the other HCoVs listed in Figure 1.

Following this analysis, we have trained and tested a neural network model for aligning protein structures. The network is a Siamese long short-term memory (LSTM) (Hochreiter and Schmidhuber, 1997) model trained to score the alignments based on BLAST supervisions and tested on the set of COVID-19 proteins previously analyzed. We obtained very promising results, showing that neural networks can be a useful resource for scoring alignments, with lower computational demand than traditional methods, such as BLAST itself.

## Materials and Methods

The NCBI SARS-CoV-2 database (MM1) provides many useful COVID-related resources and data, among which the SARS-CoV-2 and other human coronavirus sequences were used in this study. In particular, our data set comprises sequences of two alpha-HCoVs, 229E and NL63, and five beta-HCoVS: OC43, HKU1, MERS-CoV, SARS-CoV, and SARS-CoV-2. Alignments were performed with BLAST (Zhang et al., 20002000) (MM2), both on the complete genome of the viruses and more specific protein-coding regions. The first experiment started with the multiple sequence alignment of the whole genomes of the SARS-CoV-2 and the other coronaviruses in our data set. Then, each coronavirus genome was independently aligned with the genome of SARS-CoV-2, evaluating the percentage sequence identity and homology. In the second experiment, which was also carried out with BLAST, the percentage sequence identity and homology of subsequences coding for specific structural and non-structural proteins were evaluated. The complete list of proteins of SARS-CoV-2 is available on the NCBI SARS-CoV-2 database (U.S. National Library of Medicine, 2019), and the used BLASTn and BLASTp parameters are shown in Table 1. Alignments were carried out for each of the proteins (genes) of SARS-CoV-2: ORF1ab polyprotein (ORF1ab), ORF1a polyprotein (ORF1a), spike glycoprotein (S), ORF3a protein (ORF3a), envelope protein (E), membrane glycoprotein (M), ORF6 protein (ORF6), ORF7a protein (ORF7a), ORF7b protein (ORF7b), ORF8 protein (ORF8), nucleocapsid protein (N), and ORF10 protein (ORF10). Finally, a multiple sequence alignment, among all considered alpha- and beta-HCoVS, was performed based on CLUSTAL to obtain a percent identity matrix and a similarity phylogram tree, constructed by using the neighbor-joining method.

**TABLE 1:** Parameters used in BLASTn and BLASTp in the alignment of the coronavirus genomes and proteins.

BLASTn		BLASTp	
Word size	28	Word size	3
Gap cost	Linear	Gap cost	Existence: 11 Extension: 1
Match/mismatch score	1, −2	Matrix	BLOSUM62
		Compositional adjustment	Conditional score matrix adjustment

After the traditional experimentation, we performed machine learning (ML) experiments to replicate the same alignments with an artificial intelligence technique. To efficiently process our sequences, we resorted to LSTM (Hochreiter and Schmidhuber, 1997) networks: a type of neural network which has been used for sequential data processing for more than 20 years, in a wide variety of applications, including many biological tasks. In particular, in order to perform pairwise alignments of protein sequences, we built a Siamese LSTM. Indeed, Siamese networks (Bromley et al., 1993) are neural networks comprising two identical modules. The network takes pairs of examples as the input, feeding one example to each module. Siamese neural networks are specialized in the regression of distance between the two given examples. Weight sharing allows training the network to extract the information it needs for distance estimation while keeping the two modules identical throughout the process. In our case, we exploit this paradigm for estimating the similarity score of the two sequences we are aligning, as the complementary of their distance.

**FIGURE 2:**
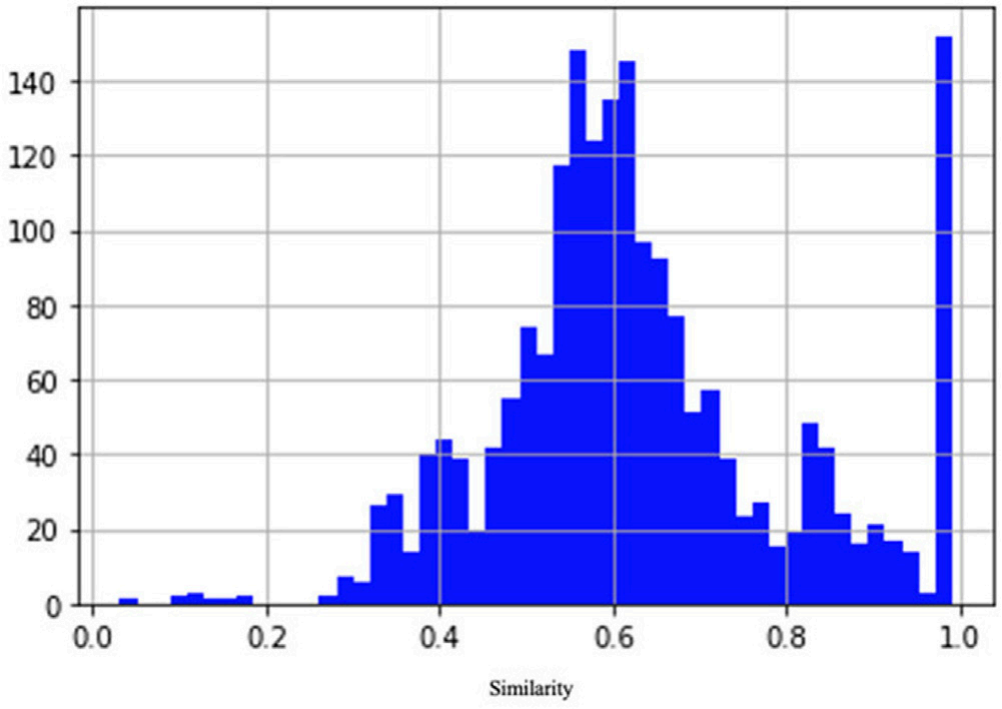
Distribution of similarity between pairs of examples in the data set. The number of pairs is proportional to similarity.

**FIGURE 3:**
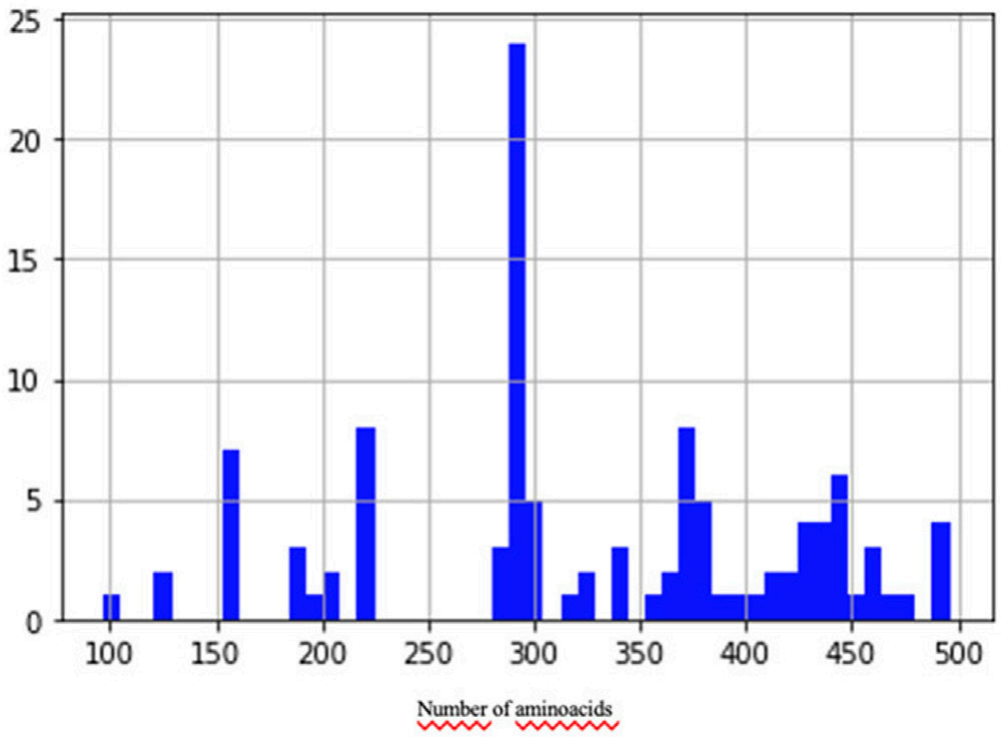
Distribution of protein sequence length in the data set.

To train and validate the neural network, we built a data set of examples based on the NCBI (NCBI Resource Coordinators, 2016) protein clusters (https://www.ncbi.nlm.nih.gov/proteinclusters). Each cluster comprises similar proteins up to a specific level of homology. This allowed us to build a data set with a relatively homogeneous and balanced distribution of distances between examples (number of pairs proportional to similarity), as shown in Figure 2. In particular, we sampled proteins from each of the following NCBI protein clusters: CHL002, CHL005, CHL008, CHL009, CHL204, CHL206, PLN4514, PLN4516, PLN4517, PLN4518, PLN4519, PLN0009, PLN0009, PLN0010, PLN0012, PLN0068, PLN0083, PLN0084, PLN0089, and PLN0091. Since 58% (35 out of 60) of COVID proteins have amino acid length between a minimum of 100 and a maximum of 550, the proteins in the clusters were filtered by length, keeping only the proteins matching this length range. This strategy allowed us to build a Siamese network which can better compare COVID proteins. After filtering, we obtained a data set of 109 proteins, with different lengths, distributed as in Figure 3. The 109 proteins have been then aligned with BLASTp, using the same parameters as in the previous experiments, and we obtained a data set of pairwise alignments comprising 1978 pairs.

The data set of 1978 protein pairs was split using a pseudo-random split generator into a training set, covering 80% of the examples (1,582 alignments), and a validation set, covering 20% of the examples (396 alignments). The latter set is used to search for the best hyperparameter values and a first assessment of the model performance. The real evaluation is carried out on the test set comprising COVID-19 sequences, which corresponds to the set of sequences from the previous experimentation.

The network and the experimentation code were written in *Python* language, using Keras (https://keras.io/) and Scikit-learn (Pedregosa et al., 2011) libraries. The neural network hyperparameters were selected after a grid search. Each Siamese module comprises a single 32-unit LSTM layer, with an input size of 550 × 21. The dimension of 21 depends on the number of amino acids (20) plus a character corresponding to an error in the protein sequencing or to an unknown amino acid. These 21 characters are one-hot encoded to form each protein sequence; sequences shorter than 550 are padded with a dedicated one-hot value. The representations coming from the two modules are combined by a merged layer, followed by a normalization layer. Finally, a single dense layer with ReLu activation estimates the distance between the two elements of the pair. The network is trained for 100 epochs, with batch size 32, mean squared error loss and Adam optimizer (Kingma and Ba, 2014).

## Results

**FIGURE 4:**
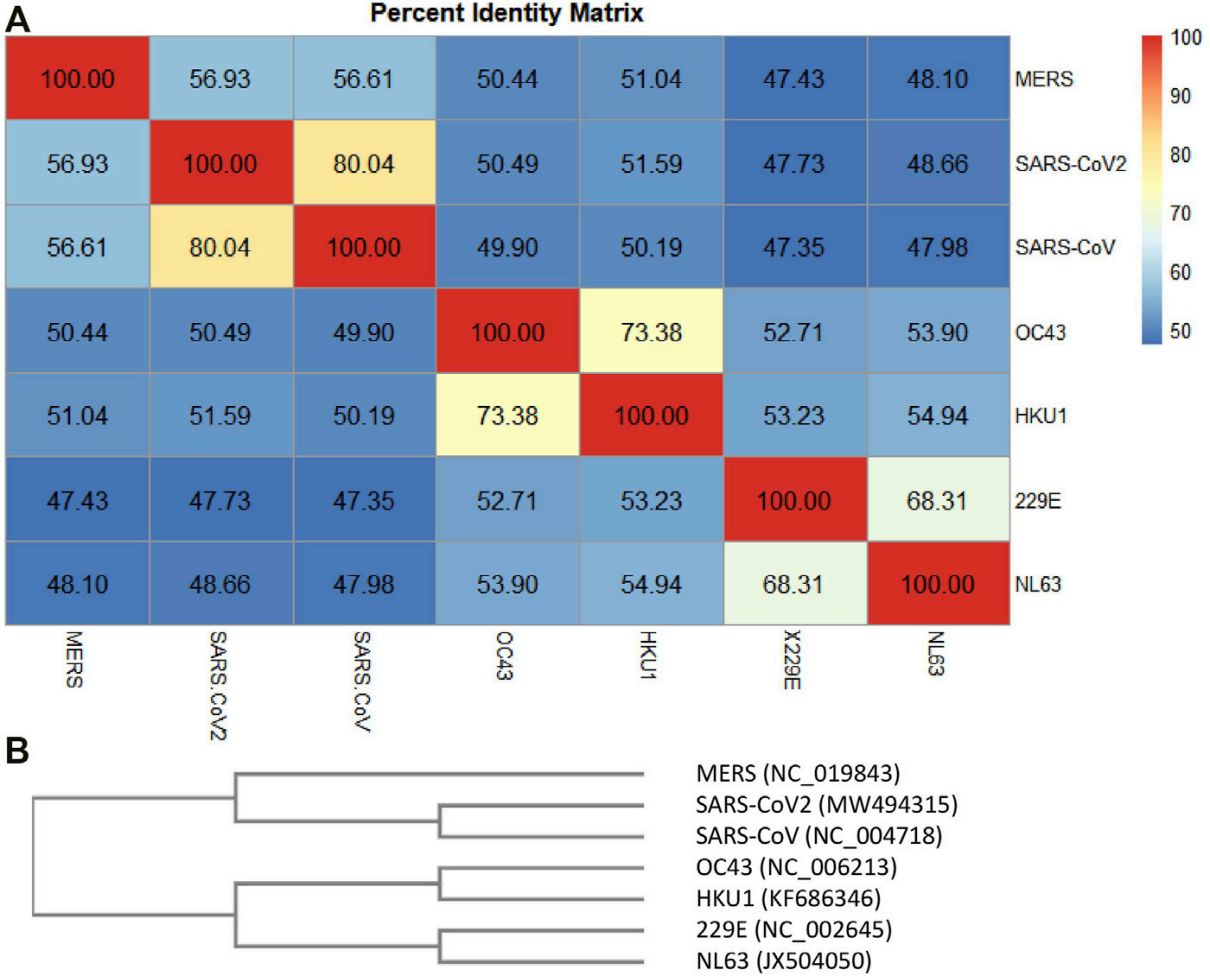
Percent identity matrix. In **(A)**, the percent identity matrix is reported, which shows the % of identities among the HCoVs. In **(B)**, the similarity phylogram tree is shown. The branch length is proportional to the number of nucleotide substitutions, that is, to the number of evolutionary events that took place after the branching point.

The circulating common cold HCoVs, usually responsible for mild respiratory symptoms and characterized by a low case fatality rate (CFR) (Gussow et al., 2020a), share partial sequence homology with SARS-CoV-2 and are extensively widespread in the population (Mateus et al., 2020). A deeper knowledge of preexisting cross-reactive T cell immunity to SARS-CoV-2 has broad implications because it could explain aspects of differential COVID-19 clinical outcomes, influence epidemiological models of herd immunity, or affect the performance of COVID-19 vaccines. Thus, we have first investigated potential similarities among the HCoV complete genome: the alpha-CoV 229E (NC_002645, 27317 base pair (bp)) and NL63 (JX504050, 27553 bp), the beta-CoV SARS-CoV-2 (MW494315 complete genome isolated in New York City, United States, 29903 bp), OC43 (NC_006213, 30741 bp), HKU1 (KF686346,29982 bp), MERS-CoV (NC_019843, 30119 bp), and SARS-CoV (NC_004718, 2 9751 bp). Our selection of the SARS-CoV-2 complete genome is based on recent studies that provided a first analysis of the SARS-CoV-2 viral genotypes collected from patients living in the NYC metropolitan area, an international hub that provides a picture of COVID-19 pandemic dynamics at the global level (Gonzalez-Reiche et al., 2020; Butler et al., 2021). The alignment of SARS-CoV-2 and the other HCoVs was obtained by BLAST. As expected, the higher sequence identity for the SARS-CoV-2 complete genome resulted with SARS-CoV (Identity 82%, Query cover 89%, and Gaps 1%), followed by HKU1 (Identity 72% but very low Query cover 5%) and by NL63 (Identity 72% but very low Query cover 2%). To obtain a whole picture of the similarities among all the considered viral strains, by performing a multiple sequence alignment (MSA), a percent identity matrix for all the sequences was generated (Figure 4A), and a similarity phylogram tree, confirming other phylogenetic analyses (Gussow et al., 2020b; Heinz and Stiasny, 2020; Jaimes et al., 2020; Chen et al., 2021; DingLiuJiaFung, 2021; Eguia et al., 2021), was created using the neighbor-joining algorithm (Figure 4B). For more details about the MSA, see Supplementary Materials (1. Schematic matches/mismatches of HCoV genomes and the whole MSA).

Each of the most closely related pairs of sequences is aligned to each other. Next, each new alignment is analyzed to build a sequence profile. Finally, alignment profiles are aligned to each other or to other sequences until a full alignment is built. Again, the higher sequence identity for the SARS-CoV-2 complete genome appeared to be with SARS-CoV (identity 80.04%), followed by MERS (56.93%) and the two beta-HCoVs, HcoV-C43 and HcoV-HKU1 (50.49 and 51.59%, respectively). The alpha-CoVs, HcoV-229E and HcoV-NL63, had lower identity values with respect to MERS, SARS-CoV, and SARS-CoV-2, despite which they showed 68.31% of identity each other. HCoV-OC43 and HCoV-HKU1 showed an identity equal to 73.38% of each other, followed by HCoV-229E and HCoV-NL63 (about 53%) and the high-CFR HCoVs (MERS, SARS-CoV, and SARS-CoV-2) with values around 50%. Figure 4B shows the phylogram of the HCoVs. A phylogram is a scaled phylogenetic tree in which the branch lengths are proportional to the amount of evolutionary divergence. Specifically, the branch length is determined by the number of nucleotide substitutions that have occurred after branching. If the branch lengths are proportional to the number of character changes, it is possible to notice the high similarity between the alpha-CoVs 229E and NL63 (number of mismatches about 3,400) and between the beta-CoVs with low-CFR (Gussow et al., 2020a) HKU1 and OC43 (number of mismatches about 2,900). Phylogenetically, SARS-CoV and SARS-CoV-2 share a most recent common ancestor within the subgenus Sarbecovirus (number of mismatches about 4,400) and are relatively distant to MERS-CoV (belonging to the subgenus Merbecovirus) in the genus Betacoronavirus (Chen et al., 2021).

In the case of T cell responses, no obvious pattern of antigen specificity was observed based on SARS-CoV-2 genome organization (Grifoni et al., 2020). Starting from this assumption, we better focused this study on the exploration of the similarity among different HCoV proteins, involved in CD4^+^ and CD8^+^ T cell responses. As reported in the study by Grifoni et al. (2020), the structural proteins S, M, and N are involved in CD4^+^ T cell response. Moreover, it is not negligible, and the additional significant response is directed against NSPs, such as nsp3, nsp4, nsp12, ORF3a, and ORF8 (Grifoni et al., 2020). Regarding SARS-CoV-2 CD8^+^ T cell responses, S and M were both recognized and significant reactivity was also observed for nsp6, ORF3a, and N (Grifoni et al., 2020). These data indicate that optimal vaccine T cell response to SARS-CoV-2 might benefit from additional classes of epitopes, such as the ones derived from the above-listed structural and non-structural proteins. This is the reason why we performed amino acid sequence alignment between the mentioned SARS-CoV-2 proteins and the other HCoVs (summarized in Table 2) with the aim of understanding their similarities in all HCoVs as a starting point for further epitope studies. In addition, preliminary identification of epitopes in SARS-CoV-2 was carried out using the Immune Epitope Database (IEDB) (https://www.iedb.org/). Experimental data on epitopes tested in the major number of T cell assays have been selected in the context of infectious diseases such as SARS-CoV-2 and then an alignment of the retrieved epitopes with homologous regions in other coronaviruses was performed. The obtained results are listed in Table 2. Moreover, the complete alignment is reported in the Supplementary Material, section 3.

**TABLE 2:** Protein sequence alignment results. Amino acid sequence alignment between the mentioned SARS-CoV-2 proteins and the other HCoVs. “—” in the column “Epitopes” indicates that no epitope has been reported in IEDB.

Structural/Non-structural proteins	Protein	HCoV	Accession	% Query cover	E-value	% Identity	% Gaps	Epitopes
Non-structural proteins	**Nsp3** (YP_009725299) SARS-CoV-2	229E	AGT21366	66	5e-55	28	14	—
		NL63	AFD98833	49	1e-48	28	12	—
		OC43	YP_009924321	74	1e-152	30	6	—
		HKU1	YP_009944271	75	1e-142	29	6	—
		MERS	YP_009047231	95	0	31	9	—
		SARS-CoV	NP_828862	100	0	76	1	—
	**Nsp4** (YP_009725300) SARS-CoV-2	229E	AGT21366	92	3e-46	27	8	—
		NL63	AFD98833	96	2e-53	29	10	—
		OC43	YP_009924322	95	5e-142	43	2	—
		HKU1	YP_459935	99	6e-137	42	2	—
		MERS	YP_009047232	98	3e-142	40	1	—
		SARS-CoV	NP_904322	100	0	80	0	—
	**Nsp6** (YP_009725302) SARS-CoV-2	229E	AGT21366	85	e-28	31	7	—
		NL63	AFD98833	80	6e-28	31	3	—
		OC43	YP_009924324	100	5e-44	32	4	—
		HKU1	YP_009944274	100	1e-44	30	2	—
		MERS	YP_009047218	100	6e-57	56	4	—
		SARS-CoV	YP_009944371	98	0	88	0	—
	**Nsp12** (YP_009725307) SARS-CoV-2	229E	ALJ99946	30	5e-129	64	0	—
		NL63	AIW52827	99	0	59	0	—
		OC43	AIW52827	99	0	66	0	—
		HKU1	AXT92527	99	0	67	0	—
		MERS	ATU80202	13	3e-60	69	0	—
		SARS-CoV	—	100	0	96	0	—
	**ORF3a** (YP_009724391) SARS-CoV-2 Epitope: LLYDANYFL	229E	—	—	—	—	—	—
		NL63	AFO70498	—	—	—	—	PLTARGRVA
		OC43	—	—	—	—	—	—
		HKU1	AXT92527	—	—	—	—	MKYHPNTVD
		MERS	AKQ21056	—	—	—	—	PLYVPE
		SARS-CoV	AAP41038	100	8e-156	72	0	LLYDANYFV
	**ORF7a** (YP_009724395) SARS-CoV-2 Epitope: QLRARSVSPKLFIRQEEVQELY	229E	—	—	—	—	—	—
		NL63	—	—	—	—	—	—
		OC43	—	—	—	—	—	—
		HKU1	—	—	—	—	—	—
		MERS	—	—	—	—	—	—
		SARS-CoV	AAP41038	—	—	—	—	QIGGYSEDRHSGVKDYVVVHGYF
	**ORF8** (YP_009724396) SARS-CoV-2 Epitope: IRVGARKSAPLIEL	229E	—	—	—	—	—	—
		NL63	—	—	—	—	—	—
		OC43	—	—	—	—	—	—
		HKU1	AZS52623	—	—	—	—	VPV-HMPVHPMVMP
		MERS	AVV62534	—	—	—	—	LEQ-DQKLTSLSEL
		SARS-CoV	AAP41043	31	0,001	30	15	YEG-NSPFHPLADN
Structural proteins	**S** (YP_009724390) SARS-CoV-2 Epitope: QYIKWPWYIW	229E	CAA71056	57	1e-111	31	12	TYIK
		NL63	QEG59362	60	2e-104	31	13	n.a.
		OC43	AIX10763	89	4e-142	38	5	YYVKWPWYVW
		HKU1	AGW27863	70	9e-141	35	6	MYVKWPWYVW
		MERS	QBM11748	78	e-177	35	7	YYNKWPWYIW
		SARS-CoV	AAR86775	100	0	76	2	QYIKWPWYVW
	**N** (YP_009724397) SARS-CoV-2 Epitope: SPRWYFYYL	229E	AGW80953	65	6e-24	29	12	SPKLHFYYL
		NL63	ABK63972	13	9e-14	47	1	PPKVHFYYP
		OC43	YP_009555245	74	6e-55	38	14	LPRWYFYYL
		HKU1	AKQ21062	—	—	—	—	APRWYFYYT
		MERS	QBM11755	91	5e-93	48	6	APRWYFYYT
		SARS-CoV	AYV99827	100	0	91	0	SPRWYFYYL
	**M** (QQD86931) SARS-CoV-2 Epitope: KEITVATSRTLSYYK	229E	QNT54758	99	2e-25	31	3	EYMTVAVPSTTIIYS
		NL63	AFV53151	90	2e-36	31	4	KYVIVATPSTTIVCD
		OC43	AAA45462	95	4e-55	41	0	AYMTVAKVTHLCTYK
		HKU1	YP_173241	93	3e-51	36	0	VYVTVAKVQVLCTYK
		MERS	AHX00737	90	2e-61	43	0	NEVTVAKPNVLIALK
		SARS-CoV	ACZ72273	100	3e-154	90	0	KEITVATSRTLSYYK

Thus, to evaluate the potential for cross-reactivity, we compared the structural and non-structural protein sequence homology among SARS-CoV-2 and the other HCoVs: MERS-CoV, SARS-CoV, HCoV-OC43, and HCoV-HKU1 and the alpha-CoVs HCoV-229E and HcoV-HKU1-NL63. The sequence alignments revealed a higher identity % of S, N, and M proteins between SARS-CoV-2 and SARS-CoV, followed by MERS for N and M proteins, and HCoV-OC43 for S protein, although the identity is only about 38%. Among the common cold HCoVs, HCoV-OC43 was found to have the most similar sequence for SARS-CoV-2 structural proteins. For ORF3a and ORF8, similarity was found only between SARS-CoV-2 and SARS-CoV, whereas no similarity was retrieved for ORF7a. As far as it concerns nsp3, nsp4, and nsp6, the amino acid sequence alignment showed higher identity for SARS-CoV, followed by MERS and HCoV-OC43. In the case of nsp12, the results for HCoV-HKU1 and HCoV-OC43 were quite comparable with high Query cover (99%) and Identity (67 and 66%, respectively). Similar to what has been observed with the genome alignment, the alpha-CoVs shared the smallest sequence identity and sequence coverage with SARS-CoV-2 structural and non-structural proteins.

This multiple alignment investigation could also be extended to the “variant of concern” (VOC) for SARS-CoV-2 (such as Delta and Omicron), which refers to viral variants with mutations in their spike protein receptor-binding domain (RBD) that dramatically improve binding affinity in the RBD-hACE2 complex, while also causing fast dissemination in human populations (Kumar et al., 2021).

**FIGURE 5:**
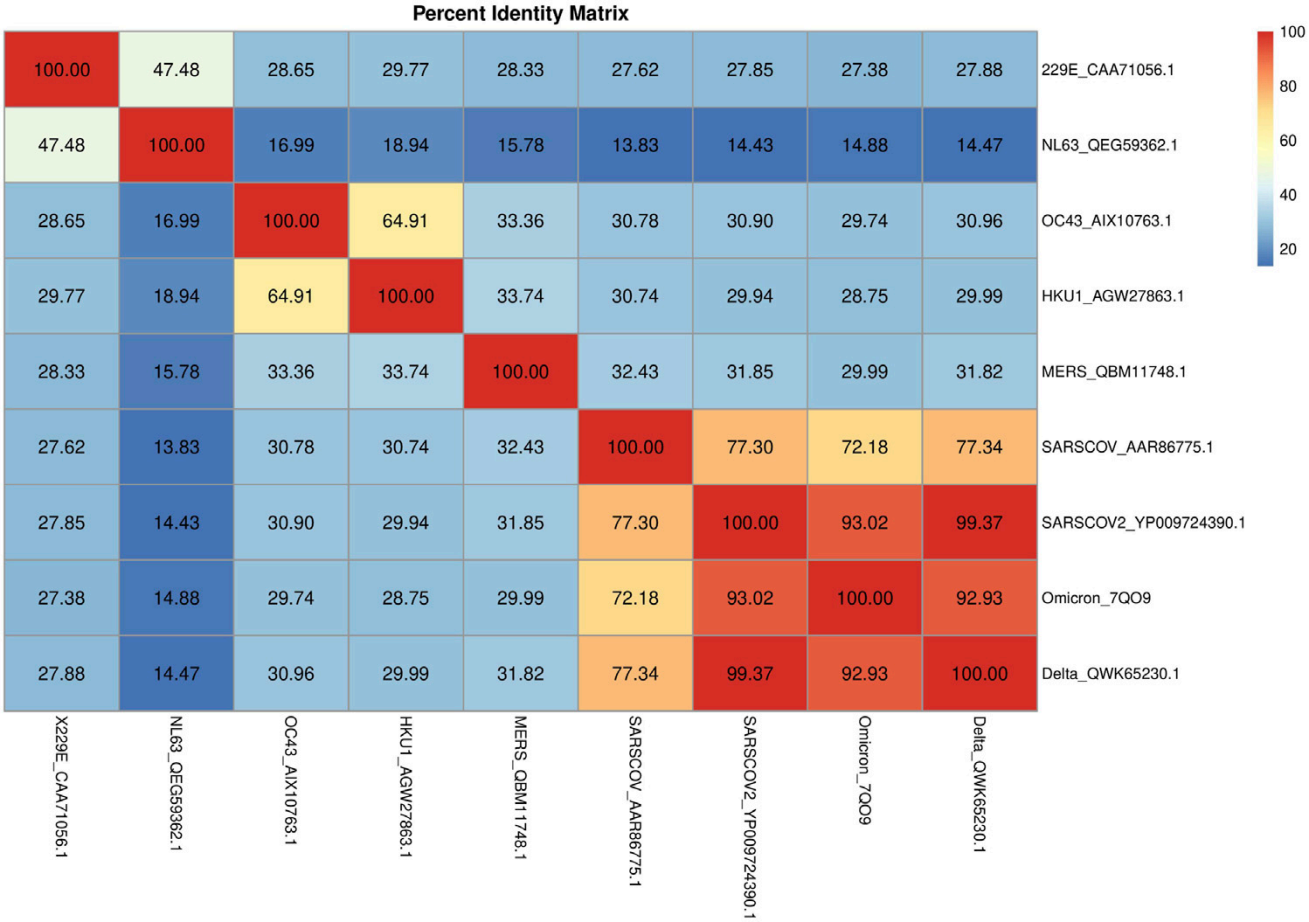
Percent identity matrix. In A, the percent identity matrix is reported, which shows the % of identities among the S proteins of HCoVs.

The S protein mediates the attachment of the virus to host cell-surface receptors and the fusion between virus and cell membranes. It is also the principal target of neutralizing antibodies generated following infection by SARS-CoV-2. Consequently, mutations that affect the antigenicity of the S protein are of particular importance. Thus, we have focused our attention on the S protein—see Supplementary Materials (3. Spike MSA)—in order to better investigate the similarities/differences in protein sequences among different HCoVs, including SARS-CoV-2 S and the most recent variants Omicron and Delta. We can confirm a lower sequence identity among alpha-CoVs and SARS-CoV-2. Moreover, concerning variants Delta_QWK65230.1 and Omicron_7QO9, the latter shared a smaller S protein sequence identity than the former (99.37 and 93.02%, respectively) (Figure 5). This trend holds true also for the higher sequence identity of Delta variants with the other HCoVs in comparison to Omicron, with the only exception constituted by the alpha-CoVs. Such smaller sequence identity between Omicron and SARS-CoV-2 S proteins could lead to significant changes, especially in the RBD region, that might contribute to high binding specificity with hACE2, which in turn may result in a higher transmission rate and considerable impact on pathogenesis when compared to the Delta variant.

The distance estimation experiments with Siamese LSTMs also showed very promising results. After selecting the best hyperparameters with a grid search over the validation set, the best model (described in Section 3) obtained an error of 0.48% on the validation set itself. Evaluating the same model on the COVID-19 test set confirmed the quality of the model on data it had not been adapted to, showing an error rate of 8%, as reported in Table 3.

**TABLE 3:** Error score of the Siamese network on training, validation, and COVID proteins sets.

% Error on the Training set	0.4
% error on validation set	0.48
% error on coronavirus set	8

The Siamese network has been trained to estimate the distance obtained from BLAST in the following way:

$$s_{i}=i_{i}*c_{i},$$

where $s_{i}$ is the measure of the similarity obtained from the multiplication of the BLAST identity score, $i_{i},$ by its cover score, $c_{i},$ with respect to the *ith* sequence. After that, we calculated the value of the distance as the complementary of similarity:

$$d_{i}=1-s_{i}.$$

The results of the network are as close to the BLAST score as the proteins represent near homologous conditions. The training set proteins contain highly conserved pairs. Conversely, the score of protein pairs which are far homologous is hardly calculated by the network.

In Table 4 the best alignment scores are reported. However, by choosing a larger training set containing more pairs of proteins with a lower degree of homology, it is possible to approximate the more accurate BLAST score.

**TABLE 4:** Best comparison of BLAST and the Siamese network distance score in COVID protein set after filtering the test data set.

Protein	HCoV	Blast distance	Siamese distance
Nsp4 SARS-CoV-2	OC43	0.6	0.65
	MERS	0.2	0.1
	SARS-CoV	0.14	0.22
ORF3a SARS-CoV-2	SARS-CoV	0.28	0.07
N SARS-CoV-2	MERS	0.56	0.4
	SARS-CoV	0.09	0.09

## Discussion

Grifoni et al. (2020) suggested that a COVID-19 vaccine consisting only of SARS-CoV-2 S protein would be able to elicit SARS-CoV-2–specific CD4^+^ T cell responses. However, the data also indicate that there are many potential CD4^+^ T cell targets in SARS-CoV-2, and the inclusion of additional SARS-CoV-2 structural antigens such as M and N would better mimic the natural SARS-CoV-2–specific CD4^+^ T cell response. Concerning SARS-CoV-2 CD8^+^ T cell responses, the S protein was an important target. SARS-CoV-2 M was just as strongly recognized, and significant reactivity was observed for other antigens (nsp6, ORF3a, and N), which comprised nearly 50% of the total CD8^+^ T cell response. Starting from this assumption, we have first performed a sequence alignment to better understand the complete genome similarity among the HCoVs and then we focused our attention on each protein involved in T cell response. By means of sequence alignment, we investigated the potential cross-reactive immunity to HCoVs of structural and non-structural proteins.

### Structural Proteins

• **S protein**: it is a type I transmembrane N-linked glycosylated protein (150–200 kDa) consisting of 1,273 amino acids and displays a varying degree of conservation across the Coronaviridae family (Yadav et al., 2021). As reported in Table 2, the sequence identity of S protein in SARS-CoV-2 to other beta-HCoVs goes from 35% (for HKU1 and MERS) to 76% for SARS-CoV, while it is lower (31%) for alpha-HCoVs, with a higher number of gaps found. Among the common cold HCoVs, characterized from low-CFR, the HCoV-OC43 was found to have the most similar S sequence to SARS-CoV-2, despite 38% of identity. Delta_QWK65230.1 and Omicron_7QO9 shared a sequence identity of, respectively, 99.37 and 93.02% with SARS-CoV-2. These results suggested that the Omicron variant, characterized by multiple mutations in the S protein, could reduce antibody neutralization and vaccine protection from infection.
• **N protein**: it is complexed in the structural organization of the nucleocapsid comprising three highly conserved domains (Yadav et al., 2021). SARS-CoV-2 is most similar to SARS-CoV, harboring sequence homology of 91% in N protein, followed by MERS-CoV (48%). Among the common cold beta-HCoVs, HCoV-OC43 shares the higher percentage of Query cover and gaps (respectively 74 and 14%). Differently, between the alpha-HCoVs, 229E resulted to have 65% of query cover and 29% of identity, whereas NL63 showed, respectively, 13 and 47%. No alignment is observed for HCoV-HKU1.
• **M protein**: it is an O-linked glycoprotein of around 25–30 kDa and is the most abundant among various structural proteins (Yadav et al., 2021). It facilitates the molecular assembly of virus particles and may be involved during pathogenesis. The M protein identity in SARS-CoV-2 was 90%, followed by MERS and HCoV-OC43. Regarding alpha-HCoVs, HCoV-229E and HCoV-NL63 share, respectively, 99 and 90% of query cover. Conversely, the sequence identity is lower.

### Non-Structural Proteins

• **Nsp3**: it is the largest protein encoded by the CoV genome, and the average molecular mass is around 200 kD, which is very important in the replication/transcription complex (Lei et al., 2018). SARS-CoV and MERS were found to have high query cover and 76 and 31% of sequence identity, respectively. Among common cold beta-HCoVs, OC43 and HKU1 have a query cover-up than 70%, but only 30 and 29% of sequence identity. The alpha-HCovs 229E and NL63 showed lower values of query cover and identity.
• **Nsp4**: it has an essential role in replication and in the assembly of the replicative structures (Raj, 2021). As reported in Table 2, nsp4 was found to have the highest identity values in SARS-CoV (80%) and in HCoV-OC43 (43%) among the beta-HCoVs with a low percentage of gaps too. In alpha-HCoVs, the sequence identity of NL63 is 29% and in 229E is 27%, with a query cover value more than 90%.
• **Nsp6**: it generates autophagosomes from the endoplasmic reticulum and is involved in autophagy (Raj, 2021). Nsp6 showed high query cover values. The sequence identity was found to be 88% in SARS-CoV, followed by MERS, OC43, and HKU1 (56, 32, and 30%). The query cover value is 100% in OC43, HKU1, and MERS and 98% in SARS-CoV.
• **Nsp12**: it is the RNA-dependent RNA polymerase, the central component of coronaviral replication and transcription machinery (Raj, 2021). The nsp12 query cover is around 99% in the beta-HCoVs, with the only exception of MERS. As far as it concerns the identity, in SARS-CoV, it is 96%, while in the other beta-HCoVs, it goes from 66% (HCoV-OC43) to 69% (HCoV-MERS). In the alpha-HCoVs, the sequence identity is lower.
• **ORF3a**, **ORF7a**, and **ORF8**: ORF3a plays a fundamental role in virus replication and release, ORF7a is a viral structural protein, and ORF8 is an accessory protein correlated with the ability of the virus to spread (Pereira, 2020). These ORFs are specific for SARS-CoV and do not show significant homology to proteins of other HCoVs.

Identification of conserved epitopes in structural and non-structural proteins is of strong interest to help design broad-spectrum vaccines against the present outbreak of SARS-CoV-2. Indeed, high-affinity neutralizing antibodies against conserved epitopes could provide immunity to SARS-CoV-2 and protection against future pandemic viruses. Our systematic MSA analysis of all structural and non-structural proteins allowed a better investigation of sequence similarities, and it is a starting point for a deeper epitope analysis (see Supplementary Material, section 3 for S protein MSA and epitope visualization). The % identity of all the proteins is summarized above, in particular the sequence alignments revealed a higher identity % of S, N, and M proteins between SARS-CoV-2 and SARS-CoV, followed by MERS, HCoV-OC43, and HCoV-HKU1. In nsp3, nsp4, and nsp6, amino acid sequence alignment showed a higher identity for SARS-CoV, followed by MERS and HCoV-OC43, whereas, concerning ORF3a and ORF8, the similarity was found only between SARS-CoV-2 and SARS-CoV, and no identity was found for ORF7a. In several studies, it has already been shown that the vast majority of N protein epitopes are characterized by low rates of evolutions. For the S protein, epitopes are preferentially located in regions that are predicted to be ordered and well-conserved and may help develop long-lasting, broad-spectrum SARS-CoV-2 vaccines (Chen et al., 2020b; Forcelloni et al., 2020). Owing to our alignment experiments carried out with Siamese LSTM neural networks, it was possible to show that such an alternative technique provides very low error rates and could be a starting point for further epitope investigation. This approach also became effective for the potential emerging SARS-CoV-2 variant investigation. Moreover, it could be a model applicable not only for SARS-CoV-2 but also for other biological queries.

## Conclusion

A preexisting immune memory due to exposure to common cold HCoVs seems to have a significant impact on the COVID-19 disease severity, thus suggesting the fundamental role of the protein sequence similarities with different circulating coronaviruses to understand SARS-CoV-2 cross-reactivity.

As expected, the sequence alignments revealed a higher identity % of S, N, and M proteins between SARS-CoV-2 and SARS-CoV, followed by MERS. Among the common cold HCoVs, HCoV-OC43 was found to have the most similar structural proteins to SARS-CoV-2 involved in T cell responses, followed by HCoV-HKU1. Regarding NSPs, nsp3, nsp4, and nsp6, amino acid sequence alignment showed a higher identity for SARS-CoV, followed by MERS and HCoV-OC43, with the only exception of nsp12, which was found to have a higher query cover and identity in the cases of HCoV-HKU1 and HCoV-OC43. Concerning ORF3a and ORF8, the similarity was found only between SARS-CoV-2 and SARS-CoV, whereas no identity was found for ORF7a. Similar to what was observed with genome alignments, the alpha-CoVs shared smaller sequence identity and sequence coverage with SARS-CoV-2 in structural and non-structural proteins. In conclusion, the aims of this work are as follows:

1. To perform MSA of both structural and non-structural proteins in a systematic way. The punctual list of identity (%) could be helpful in addressing deeper studies on cross-reactive epitopes. A deeper investigation of cross-reactive T-cell immunity to SARS-CoV-2 has extensive implications in differential COVID-19 clinical outcomes and can influence the performance of COVID-19 vaccines. Structural proteins and NSPs are involved in CD4^+^ and CD8+ T cell response; thus, we have better examined the similarity of their amino acid sequence across HCoVs as a starting point for further studies about cross-reactive T-cell recognition between circulating common cold HCoVs and SARS-CoV-2.

2. Finally, alignment experiments carried out with the Siamese LSTM neural networks showed that artificial intelligence techniques show very low error rates with respect to the ground truth provided by BLAST. This indicates that such models are competitive with the traditional methods for pair alignment, being less expensive in terms of computational costs. Taking into account that we align sequences of comparable length and *n* being the length of the longest one and *m* the number of units in the LSTM layer, BLAST has a time complexity of O (*n* ^2^), while the Siamese network has a time complexity in the order of O (*n*×*m*), with *m* << *n*. Being trained on BLAST supervisions with good results, our model is competitive with BLAST, but it cannot be better than it. Moreover, all the considerations made in the literature concerning the BLAST alignment scores hold true for our score. It is to be noted, though, that our model can be repurposed, according to the needs of the user, by training it on supervisions from any available alignment method.

## Data Availability

The original contributions presented in the study are included in the article/Supplementary Material; further inquiries can be directed to the corresponding author.
