# Supplementary material for: A Bioinformatics Approach to Investigate Structural and Non-Structural Proteins in Human Coronaviruses
Source: Front Genet. 2022 Jun 14;13:891418. doi: 10.3389/fgene.2022.891418 (PMC9237418; doi:10.3389/fgene.2022.891418)
Supplement: Supplementary file 1 [file DataSheet1.docx]

1. **Schematic matches/mismatches of HCoVs’ genomes.** This method highlights differences observed from the sequence in an alignment. Mismatches are shown in red.


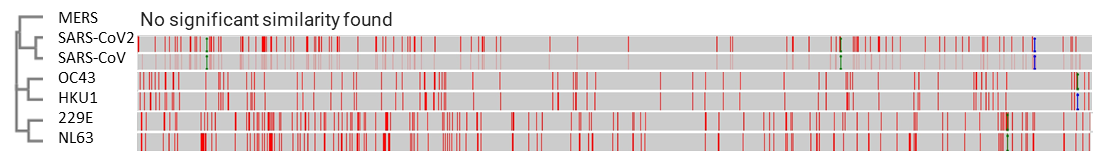


1. **Multiple Sequence Alignment (MSA)** is generally the alignment of three or more biological sequences (protein or nucleic acid) of similar length. From the output, homology can be inferred and the evolutionary relationships between the sequences studied.

CLUSTAL O(1.2.4) multiple sequence alignment

NC_019843.3 -------GATTTAAGTGAATAGCTTGGCTATCTCACTTCCCCTCGTTCTCTTG-CAGAAC 52

NC_004718.3 ATATTAGGTTTTTACCTACCCAGG--AAAAGCCAACCAAC-CTCGATCTCTTG-TAGATC 56

EPI_ISL_6640916_omicron -------------------------------------------------------AGATC 5

MW494315.1 ATTAAAGGTTTATACCTTCCCAGGTAACAAACCAACCAACTTTCGATCTCTTG-TAGATC 59

KF686346.1 --------------------------CGTCTATCAGCTTAC---GATCTCTTGTCAGATC 31

NC_006213.1 -------ATTGTGAGCGATTTGCGTGCGTGCATCCCGCTTCACTGATCTCTTGTTAGATC 53

NC_002645.1 -------------------------ACTTAAGTACCTTATC---TATCTACAGATAGAAA 32

JX504050.1 --------------------------CTTAAAGAATTTTTC---TATCTATAGATAGAGA 31

***

NC_019843.3 TTTGAT------------------------------------------------------ 58

NC_004718.3 TGTTCT------------------------------------------------------ 62

EPI_ISL_6640916_omicron TGTTCT------------------------------------------------------ 11

MW494315.1 TGTTCT------------------------------------------------------ 65

KF686346.1 TCATTA-AATCTAAACTTTTTAAACA---AGATTCCCTGTTATCCATGCTTGTGAGTGTG 87

NC_006213.1 TTTTTGTAATCTAAACTTTATAAAAACATCCACTCCCTGTAATCTATGCTTGTGGGCGTA 113

NC_002645.1 AGTTGCTT-TTTAGACTTTGTGTCTACTTTTCTCAACTAAACGAAATTTTTGCTATGGCC 91

JX504050.1 ATTTTCTTATTTAGACTTTGTGTCTACTCTTCTCAACTAAACGAAATTTTT-CTAGTGCT 90

NC_019843.3 --------------------TTTAACGAACTTAAATAAAAGCCCTGTTGTTTAGCGTATC 98

NC_004718.3 --------------------CTAAACGAACTTTAAAATCTGTGTAGCTGT---------- 92

EPI_ISL_6640916_omicron --------------------CTAAACGAACTTTAAAATCTGTGTGGCTGT---------- 41

MW494315.1 --------------------CTAAACGAACTTTAAAATCTGTGTGGCTGT---------- 95

KF686346.1 GT-------------TTAATCATAATCTTGT--ATTTTACTTTCCACACTTTT-CATCTC 131

NC_006213.1 GA-------------TTTTTCATAGTGGTGTTTATATTCATTTCTGCTGTTAA-CAGCTT 159

NC_002645.1 GGCATCTTTGATGCTGGAGTCGTAGTGTAATTGAAATTTCATTTGGGTTGCAACAGTTTG 151

JX504050.1 GTCATTTGTTATG--GCAGTCCTAGTGTAATTGAAATTTCGTCAAGTTTGTAA-ACTGGT 147

* * *

NC_019843.3 GTTGCACTTGTCTGGTGG------------------------------------------ 116

NC_004718.3 ------------------------------------------------------------ 92

EPI_ISL_6640916_omicron ------------------------------------------------------------ 41

MW494315.1 ------------------------------------------------------------ 95

KF686346.1 TCTGCCAGTGACGTGTTG-GTTGTCCTCAGCGTCCCTCCCATAGGTCGCAATGATTAAAA 190

NC_006213.1 TCAGCCAGGGACGTGTTGTATCCTAGGCAGTGGCCCGCCCATAGGTCACAATGTCGAAGA 219

NC_002645.1 GAAGCAAGTGCTGTGTGTCCTAGTCTAAGGGTTTCGTGTTCCGTCACGAGATTCCATTCT 211

JX504050.1 TAGGCAAGTGTTGTATTTTCTGTGTCTAAGCACTGGTGATTCTGTTCACTAGTGCATACA 207

NC_019843.3 ---------------------------------------GATTGTGGCATTAATTTGCCT 137

NC_004718.3 ------------------------------------------------------------ 92

EPI_ISL_6640916_omicron ------------------------------------------------------------ 41

MW494315.1 ------------------------------------------------------------ 95

KF686346.1 CCAG-----------CAAATACGGTCTCGGCTTCAAGTGGGCGCCAGAATTTCGTTGGCT 239

NC_006213.1 TCAA-----------CAAATACGGTCTCGAACTACACTGGGCTCCAGAATTTCCATGGAT 268

NC_002645.1 ACAAACGCCTTACTCGAGGTTCCGTCTCGTGTTTGTGTGGAAGCAAA------------- 258

JX504050.1 CTGA--TATTTAAGTGGTGTTCCGTCACTGCTTATTGTGGAAGCAAC------------- 252

NC_019843.3 GCTCATCTAGGCAGTGGACATATGCTCAACACTGGGTATAATTCTAATTGAATACTATTT 197

NC_004718.3 ----CGCTCGGCTGCATGCCTAGTGCACCTACGCAGTATAAACAATAATAAATTTTACTG 148

EPI_ISL_6640916_omicron ----CACTCGGCTGCATGCTTAGTGCACTCACGCAGTATAATTAATAACTAA--TTACTG 95

MW494315.1 ----CACTCGGCTGCATGCTTAGTGCACTCACGCAGTATAATTAATAACTAA--TTACTG 149

KF686346.1 GCTTCCGGATGCAGCGGAGGAGTTGGCTAGTCCTATGAAGTCAGATGAGGGT-------- 291

NC_006213.1 GTTTGAGGACGCAGAGGAGAAGTTGGATAACCCTAGTAGTTCAGAGGTGGAT-------- 320

NC_002645.1 ------------------------------------------------------------ 258

JX504050.1 ------------------------------------------------------------ 252

NC_019843.3 TTCAGTTAGAGCGTCGTGTCTCTTGTACGTCTCGGTCACAATAC--ACGGTTTCGTCCGG 255

NC_004718.3 TCGTTGACAAGAAACGAGTAACTCGTCCCTCTTCTGCAGACTGCTTACGGTTTCGTCCGT 208

EPI_ISL_6640916_omicron TCGTTGACAGGACACGAGTAACTCGTCTATCTTCTGCAGGCTGCTTACGGTTTCGTCCGT 155

MW494315.1 TCGTTGACAGGACACGAGTAACTCGTCTATCTTCTGCAGGCTGCTTACGGTTTCGTCCGT 209

KF686346.1 ----------------------------------------------GGGTTATGCCCCTC 305

NC_006213.1 ----------------------------------------------ATGATTTGCTCCAC 334

NC_002645.1 ------------------------------------------------------------ 258

JX504050.1 ------------------------------------------------------------ 252

NC_019843.3 TGCGTGGCAATTCGGGGCACATCATGTCTTTCGTGGCTGGTGTGACCGCGCAAGGTGCGC 315

NC_004718.3 GTTGCAGTCGATCATCAGCATACCTAGGTTTCGT-CCGGGTGTGAC--CGAAAGGTAAGA 265

EPI_ISL_6640916_omicron GTTGCAGCCGATCATCAGCACATCTAGGTTTTGT-CCGGGTGTGAC--CGAAAGGTAAGA 212

MW494315.1 GTTGCAGCCGATCATCAGCACATCTAGGTTTTGT-CCGGGTGTGAC--CGAAAGGTAAGA 266

KF686346.1 TACTGGTCAAGCGATGGAAAGTGTTGGATTCGTT-------------------------- 339

NC_006213.1 CACTGCGCAAAAGCTGGAAACAGACGGAATTTGT-------------------------- 368

NC_002645.1 --------------------------------GT-------------------------- 260

JX504050.1 --------------------------------GT-------------------------- 254

*

NC_019843.3 GCGGTACGTATCGAGCAGCGCTCAACTCTGAAAAACAT---------------CAAGACC 360

NC_004718.3 TGGAGAGCCTTGTTCTTGGTGTCAACGAGAAAACACACGTCCAACTCAGTTTGCCTGTCC 325

EPI_ISL_6640916_omicron TGGAGAGCCTTGTCCCTGGTTTCAACGAGAAAACACACGTCCAACTCAGTTTGCCTGTTT 272

MW494315.1 TGGAGAGCCTTGTCCCTGGTTTCAACGAGAAAACACACGTCCAACTCAGTTTGCCTGTTT 326

KF686346.1 -----------------TATGATAATCATGTGAAGATAGATTGTCGCTGCATTCTTGGAC 382

NC_006213.1 -----------------CCTGAAAATCATGTGATGGTGGATTGTCGCCGACTTCTTAAAC 411

NC_002645.1 -----------------TCTGTCTTTGTGGAAACCAGTAACTGTTCCTAATGGCCTGCAA 303

JX504050.1 -----------------TCTGTCGTTGTGGAAACCAATAACTGCTAACCATGTTTTACAA 297

*

NC_019843.3 ATGTGTCTCTAACTGTGCCACTCTGTGGTTCAGGAAACCTGGTTGAAAAACTTTCACCAT 420

NC_004718.3 TTCAGGTTAGAGACGTGCTAGTGCGTGGCTTCGGGGACTCTGTGGAAGAGGCCCTATCGG 385

EPI_ISL_6640916_omicron TACAGGTTCGCGACGTGCTCGTACGTGGCTTTGGAGACTCCGTGGAGGAGGTCTTATCAG 332

MW494315.1 TACAGGTTCGCGACGTGCTCGTACGTGGCTTTGGAGACTCCGTGGAGGAGGTCTTATCAG 386

KF686346.1 AAGAAT---GGCATGTGCAGTCAAATCTTATCCGTGATATTTTTGTTCATGAAGATCTAC 439

NC_006213.1 AAGAGT---GTTGTGTGCAGTCTAGCCTAATACGTGAAATTGTTATGAATGCAAGTCCAT 468

NC_002645.1 CCGTGT---GACACTTGCCGTAGCAAGTGATTCTGAAATTTCTGCAAATGGCTGTTCTAC 360

JX504050.1 TCAAGT---GACACTTGCTGTTGCAAGTGATTCGGAAATTTCAGGTTTTGGTTTTGCCAT 354

*** *

NC_019843.3 GG------------TTCATGGATGGCGA-------------------------------- 436

NC_004718.3 AGGCACGTGAACACCTCAAAAATGGCAC-------------------------------- 413

EPI_ISL_6640916_omicron AGGCACGTCAACATCTTAAAGATGGCAC-------------------------------- 360

MW494315.1 AGGCACGTCAACATCTTAAAGATGGCAC-------------------------------- 414

KF686346.1 ATGTTGTAGAAGTTCTAACTAAAACAGCCGTAAAGTCCGGTACGGCAATTTTAATTAAAT 499

NC_006213.1 ATGATTTGGAGGTGCTACTTCAAGATGCTTTGCAGTCCCGTGAAGCAGTTTTGGTTACAA 528

NC_002645.1 TATTGCGCAAGCCGTCCGCCGTTATAGC----GAGGCCGCTAGCAATGGTTTTAGGGCAT 416

JX504050.1 TCCTTCTGTAGCCGTTCGCACCTATAGC----GAAGCCGCTGCACAAGGTTTTCAGGCAT 410

NC_019843.3 -------------------------------------AAATGCCTATGAAGTGGTGAAGG 459

NC_004718.3 -------------------------------------TTGTGGTCTAGTAGAGCTGGAAA 436

EPI_ISL_6640916_omicron -------------------------------------TTGTGGCTTAGTAGAAGTTGAAA 383

MW494315.1 -------------------------------------TTGTGGCTTAGTAGAAGTTGAAA 437

KF686346.1 CACCTTTGCATAGCTT---------------------GGGTGGTTTTCCTAAAGGGTATG 538

NC_006213.1 CCCCCTTAGGTATGTCTTTAGAGGCATGCTATGTGAGAGGTTGTAATCCTAAAGGATGGA 588

NC_002645.1 GCCGATTTGTTTCATTAGATTTGCAGGATTGCATCGTTGGCATTGCAGACGATACATATG 476

JX504050.1 GCCGTTTTGTTGCTTTTGGCTTACAGGATTGTGTAACCGGTATTAATGATGATGATTATG 470

NC_019843.3 CCATGTTACTTAAAAAGGAGCCACTTCTCTATGTGCCCATCCGGCTGGCTGGACACACTA 519

NC_004718.3 AAGGCGTACTGCCCCAGCTTGAACAGCCCTATGTGTTCATTAAACGTTCTGATGCCTTAA 496

EPI_ISL_6640916_omicron AAGGCGTTTTGCCTCAACTTGAACAGCCCTATGTGTTCATCAAACGTTCGGATGCTCGAA 443

MW494315.1 AAGGCGTTTTGCCTCAACTTGAACAGCCCTATGTGTTCATCAAACGTTCGGATGCTCGAA 497

KF686346.1 TTATGGGCTTGTTCC------------------------GTTCATACAAGACTAAACGTT 574

NC_006213.1 CCATGGGTTTGTTTCGGCGTAGAAGTGTGTGTAACACTGGTCGTTGCACTGTTAATAAGC 648

NC_002645.1 TTATGGGTCTGCATGGCAATCAGACGTTGTTTTGCAACATAATGAAATTTTCTGACCGTC 536

JX504050.1 TCATTGCATTGACTGGTACTAATCAGCTCTGTGCCAAAATTTTACCTTTTTCTGATAGAC 530

*

NC_019843.3 GACACCTCCCAGGTCCTCGTGTGTACCTGGTTGAGAGGCTCATTGCTTGTGAAAATCCAT 579

NC_004718.3 GCACCAATCACGGCCACAAGGTCGTTGAGCTGG--------------------------- 529

EPI_ISL_6640916_omicron CTGCACCTCATGGTCATGTTATGGTTGAGCTGG--------------------------- 476

MW494315.1 CTGCACCTCATGGTCATGTTATGGTTGAGCTGG--------------------------- 530

KF686346.1 ATGTTGTACATCATCTTTCTATGA------CTACATCTACTACTAATTTTGGTGAAGATT 628

NC_006213.1 ATGTGGCCTATCAGTTATATATGATTGATCCTGCAGGTGTCTGTCTTGGTGCAGGTCAAT 708

NC_002645.1 CTTTTATGCTTCATGGGTGGTTGG------------------------------------ 560

JX504050.1 CCCTTAATTTGCGAGGTTGGCTCA------------------------------------ 554

*

NC_019843.3 TCATGGTTAACCAATTGGCTTATAGCTCTAGTGCAAATGGCAGCCTGGTTGGCACAACTT 639

NC_004718.3 ----------------------------TTGCAGAAATGGACGGCATTCAGTACGGTCGT 561

EPI_ISL_6640916_omicron ----------------------------TAGCAGAACTCGAAGGCATTCAGTACGGTCGT 508

MW494315.1 ----------------------------TAGCAGAACTCGAAGGCATTCAGTACGGTCGT 562

KF686346.1 TTTTGGGT---------------------------------------------------- 636

NC_006213.1 TCGTGGGT---------------------------------------------------- 716

NC_002645.1 ------------------------------------------------------------ 560

JX504050.1 ------------------------------------------------------------ 554

NC_019843.3 TGCAGGGCAAGCCTATTGGTATGTTCTTCCCTTATGACATCGAACTTGTCACAGGAAAGC 699

NC_004718.3 AGC--GGTATAACACTGGGAGTACTCGTGCCACATGTGGGCGAAACCCCAATTGCATACC 619

EPI_ISL_6640916_omicron AGT--GGTGAGACACTTGGTGTCCTTGTCCCTCATGTGGGCGAAATACCAGTGGCTTACC 566

MW494315.1 AGT--GGTGAGACACTTGGTGTCCTTGTCCCTCATGTGGGCGAAATACCAGTGGCTTACC 620

KF686346.1 -----TGGATTGTACCTTTTGGTTTTATGCCATCTTATGTTCACAAATGGTTTCAATTCT 691

NC_006213.1 -----TGGGTCATACCCTTAGCCTTTATGCCTGTGCAATCCCGGAAATTTATTGTTCCAT 771

NC_002645.1 ------------------------------------------------------------ 560

JX504050.1 ------------------------------------------------------------ 554

NC_019843.3 AAAATATTCTCCTGCGCAAGTA---------------TGGC------CGTGGTGGTTATC 738

NC_004718.3 GCAATGTTCTTCTTCGTAAGAACGGTAATAAGGGAGCCGGT------GGTCATAGCTATG 673

EPI_ISL_6640916_omicron GCAAGGTTCTTCTTCGTAAGAACGGTAATAAAGGAGCTGGT------GGCCATAGTTACG 620

MW494315.1 GCAAGGTTCTTCTTCGTAAGAACGGTAATAAAGGAGCTGGT------GGCCATAGTTACG 674

KF686346.1 GTAGGTTGTATATTGAAGAGAGTGATTTAATAATTTCAAATTTTAAATTTGATGA----- 746

NC_006213.1 GGGTTATGTACTTGCGTAAGCGTGGCGAAAAGGGTGCTTACAATAAAGATCATGGACGTG 831

NC_002645.1 ------------------------------------------------------------ 560

JX504050.1 ------------------------------------------------------------ 554

NC_019843.3 ACTACACCCCATTCCACTATGAGCGAGACAACACCTCTTGCCCTGAGTGGATGGACGATT 798

NC_004718.3 GCATCGATCTAAAGTCTTATGACTTAGGTGACGAGCTTGGCACTGATCCCATTGAAGATT 733

EPI_ISL_6640916_omicron GCGCCGATCTAAAGTCATTTGACTTAGGCGACGAGCTTGGCACTGATCCTTATGAAGATT 680

MW494315.1 GCGCCGATCTAAAGTCATTTGACTTAGGCGACGAGCTTGGCACTGATCCTTATGAAGATT 734

KF686346.1 ----------------TTATGATTTTAGTGTAGAAGATGTTTATGCTGAGGTTCATGCTG 790

NC_006213.1 GCGGTTTTGGACATGTTTATGATTTTAAAGTTGAAGATGCTTATGACCAGGTGCATGATG 891

NC_002645.1 ---------------------------------------------------------TTT 563

JX504050.1 ---------------------------------------------------------TTT 557

*

NC_019843.3 TTGAGGCGGATCCTAAAGGCAAATATG------------CCCAGAATCTGCTTAAGAAGT 846

NC_004718.3 ATGAACAAAACTGGAACACTAAGCATGGCAGTGGTGCACTCCGTGAACTCACTCGTGAGC 793

EPI_ISL_6640916_omicron TTCAAGAAAACTGGAACACTAAACATAGCAGTGGTGTTACCCGTGAACTCATGCGTGAGC 740

MW494315.1 TTCAAGAAAACTGGAACACTAAACATAGCAGTGGTGTTACCCGTGAACTCATGCGTGAGC 794

KF686346.1 AGCCTAAAGGTAAATATTCACAAAAAG------------CTTATGCTTTACTTAGACAAT 838

NC_006213.1 AGCCTAAGGGTAAGTTTTCTAAGAAGG------------CTTATGCTTTAATTAGAGGGT 939

NC_002645.1 TTTCCAATTCAAATTACCTTTTGGAGG------------AATTTGATGTTGTCTTCGGTA 611

JX504050.1 TTCCTAACAGCAATTATGTTCTTCAGG------------ACTTTGATGTTGTTTTTGGCC 605

* *

NC_019843.3 TGATTGGCGGTGATGTCACTCCA---GTTGACCAATACATGTGTGGCGTTGATGGAAAAC 903

NC_004718.3 TCAATGGAGGTGCAGTCACTCGCTATGTCGACAACAATTTCTGTGGCCCAGATGGGTACC 853

EPI_ISL_6640916_omicron TTAACGGAGGGGCATACACTCGCTATGTCGATAACAACTTCTGTGGCCCTGATGGCTACC 800

MW494315.1 TTAACGGAGGGGCATACACTCGCTATGTCGATAACAACTTCTGTGGCCCTGATGGCTACC 854

KF686346.1 ATCGTGGTATTAAACCCGTACTTTTTGTAGACCAGTATGGTTGTGACTATTCTGGTAAAT 898

NC_006213.1 ATCGTGGTGTTAAACCACTTCTCTATGTAGACCAGTATGGTTGTGATTATACTGGTAGTC 999

NC_002645.1 AGAGAGGTGGTGGTAATGTGACATACACTGACCAGTATCTCTGTGGCGCCGATGGCAAAC 671

JX504050.1 AT---GGTGCAGGAAGTGTGGTTTTTGTGGATAAGTACATGTGTGGTTTTGATGGTAAAC 662

** ** * * **** ***

NC_019843.3 CCATTAGTGCCTACGCATTTTTAATGGCCAAGGATGGAATAACCAAACTGGCT------- 956

NC_004718.3 CTCTTGATTGCATCAAAGATTTTCTCGCACGCGCGGGCAAGTCAATGTGCACT------- 906

EPI_ISL_6640916_omicron CTCTTGAGTGCATTAAAGACCTTCTAGCACGTGCTGGTAAAGCTTCATGCACT------- 853

MW494315.1 CTCTTGAGTGCATTAAAGACCTTCTAGCACGTGCTGGTAAAGCTTCATGCACT------- 907

KF686346.1 TAGCAGATTGTCTTCAAGCTTATGGTCATTATTCTTTGCAAGATATGAGACAAAAGCAGT 958

NC_006213.1 TTGCAGATGGCTTAGAGGCTTATGCTGATAAGACATTGCAAGAAATGAAGGCATTATTTC 1059

NC_002645.1 CTGT--TATGAGTGAAGATTTATGGCAGTTTGTTGACCATTTCGGTGAGAACG------- 722

JX504050.1 CTGT--GTTACCTAAAAACATGTGGGAATTTAGGGATTACTTTAATAATAATA------- 713

NC_019843.3 --------------------------GATGTTGAAGCGGACGTCGCAGCACGTGCTGATG 990

NC_004718.3 --------------------------CTTTCCGAA---CAACTTGATTACATCGAGTCGA 937

EPI_ISL_6640916_omicron --------------------------TTGTCCGAA---CAACTGGACTTTATTGACACTA 884

MW494315.1 --------------------------TTGTCCGAA---CAACTGGACTTTATTGACACTA 938

KF686346.1 CTGTATGGCTTGCCAATTGTGATTTTGATATTGTA------------------------- 993

NC_006213.1 CTACTTGGAGTCAGGAACTCCTTTTTGATGTAATT------------------------- 1094

NC_002645.1 AAGAAATTATCATCAATGGTCAT---ACTTACGTT------------------------- 754

JX504050.1 CTGATAGTATTGTTATTGGTGGTGTCACTTATCAA------------------------- 748

NC_019843.3 ACGAAGGCTTCATCACATTAAAGAACAATCTATATAGATTGGTTTGGCATGTTGAGCGTA 1050

NC_004718.3 AGAGAGGTGTCTACTGCTGCCGTGACCATGAGCATGAAATTGCCTGGTTCACTGAGCGCT 997

EPI_ISL_6640916_omicron AGAGGGGTGTATACTGCTGCCGTGAACATGAGCATGAAATTGCTTGGTACACGGAACGTT 944

MW494315.1 AGAGGGGTGTATACTGCTGCCGTGAACATGAGCATGAAATTGCTTGGTACACGGAACGTT 998

KF686346.1 --------------------------------------GTGGCTTGGCATGTAGTTCGTG 1015

NC_006213.1 --------------------------------------GTGGCATGGCATGTTGTGCGTG 1116

NC_002645.1 --------------------------------------TGTGCTTGGCTTACTAAGCGTA 776

JX504050.1 --------------------------------------CTAGCATGGGATGTTATACGTA 770

* *** **

NC_019843.3 AAGACGTTCCATATCCTAAGCAATCTATTTTTACTATTAATAGTGTGGTCCA-------- 1102

NC_004718.3 CTGATAAGAGCTACGAGCACCAGACACCCTTCGAAATTAAGAGTGCCAAGAA-------- 1049

EPI_ISL_6640916_omicron CTGAAAAGAGCTATGAATTGCAGACACCTTTTGAAATTAAATTGGCAAAGAA-------- 996

MW494315.1 CTGAAAAGAGCTATGAATTGCAGACACCTTTTGAAATTAAATTGGCAAAGAA-------- 1050

KF686346.1 ATTCACGATTTGTTATGCGCCTGCAGACTATAGCTACTATTTGTGGTATTAAATATGTTG 1075

NC_006213.1 ATCCACGTTATGTTATGAGATTGCAGAGTGCTGCTACTATACGTAGTGTTGCATATGTTG 1176

NC_002645.1 AGCCCTTAGATTACAAACGTCAGAACAACCTTGCCATTGAAGAGATTGAATATGTGCATG 836

JX504050.1 AAGACCTTTCTTATGAACAGCAAAATGTTTTAGCCATTGAGAGCATTCATTA---CCTTG 827

* *

NC_019843.3 ----------------------------------------AAAGGATGGTGTTGAAAACA 1122

NC_004718.3 ----------------------------------------ATTTGACACTTTCAAAGGGG 1069

EPI_ISL_6640916_omicron ----------------------------------------ATTTGACACCTTCAATGGGG 1016

MW494315.1 ----------------------------------------ATTTGACATCTTCAATGGGG 1070

KF686346.1 CACAACCTACAGAAGATGTAGTAGATGGAGATGTAGTTATACGTGAACCTGTACATTTAT 1135

NC_006213.1 CTAATCCTACTGAAGACTTGTGTGATGGTTCTGTTGTTATAAAAGAACCTGTGCATGTTT 1236

NC_002645.1 GTGATGCTTTGCATACACTACGCAATGGTTCTGTTCTTGAAATGGCTAAGGAAGTGAAGA 896

JX504050.1 GTACTACAGGTCATACTTTGAAGTCTGGTTGCAAACTTACTAATGCTAAGCCGCCTAAAT 887

*

NC_019843.3 CTCCTCCTCACTAT---TTTACTCTTGGATGCAAAATTTTAACGC--------------- 1164

NC_004718.3 AATGCCCAAAGTTTGTGTTTCCTCTTAACTCAAAAGTCAAAGTCA--------------- 1114

EPI_ISL_6640916_omicron AATGTCCAAATTTTGTATTTCCCTTAAATTCCATAATCAAGACTA--------------- 1061

MW494315.1 AATGTCCAAATTTTGTATTTCCCTTAAATTCCATAATCAAGACTA--------------- 1115

KF686346.1 TATCTGCTGATGCAATAGTTTTAAAGCTTCCTAGTTTGATGAAAG--------------- 1180

NC_006213.1 ATGCAGATGACTCTATTATTTTACGTCAATATAATTTAGTTGACA--------------- 1281

NC_002645.1 CATCTAGTAAAGTTGTGTTAAGCGATGCTCTTGACAAACTTTACAAAGTCTTTGGTTCTC 956

JX504050.1 ATTCTTCTAAGGTTGTTTTGAGTGGTGAATGGAATGCTGTGTATAGGGCGTTTGGTTCAC 947

* *

NC_019843.3 ---TCACCCCACGCAA-------------------------------------------- 1177

NC_004718.3 ---TTCAACCACGTGT-------------------------------------------- 1127

EPI_ISL_6640916_omicron ---TTCAACCAAGGGT-------------------------------------------- 1074

MW494315.1 ---TTCAACCAAGGGT-------------------------------------------- 1128

KF686346.1 ---TTATGACTCATAT---------GGATGATTTTTCTATTAAATCTATATATAATGTTG 1228

NC_006213.1 ---TTATGAGTCATTTTTATATGGAGGCAGATACAGTTGTAAATGCTTTTTATGGTGTTG 1338

NC_002645.1 CTGTTATGACAAATGGTTCCAACATCCTAGAGGCCTTTACTAAACCTGTGTTTATTAGTG 1016

JX504050.1 CATTTATTACAAATGGTATGTCATTGCTAGATATAATTGTTAAACCAGTTTTCTTTAATG 1007

*

NC_019843.3 -------------------------CAAGTGGAGTGGCGTTTCTGACTTGTCCCTCAAAC 1212

NC_004718.3 -------------------------TGAAAAGAAAAAGACTGAGGGTTTCATGGGGCGT- 1161

EPI_ISL_6640916_omicron -------------------------TGAAAAGAAAAAGCTTGATGGCTTTATGGGTAGA- 1108

MW494315.1 -------------------------TGAAAAGAAAAAGCTTGATGGCTTTATGGGTAGA- 1162

KF686346.1 ATTTGTGTGATTGTGGTTTTGTTATGCAGTATGGTTATGTAGATTGTTTTAATGATAAT- 1287

NC_006213.1 CTTTGAAAGATTGCGGTTTTGTTATGCAGTTTGGTTACATTGATTGCGAACAAGACTCG- 1397

NC_002645.1 CATTAGTTCAATGTACTTGTGGTACCAAGTCTTGGTCTGTTGGTGATTGGACCGGTTTT- 1075

JX504050.1 CTTTTGTTAAATGCAATTGTGGTTCTGAGAGTTGGAGTGTTGGTGCATGGGATGGTTAC- 1066

*

NC_019843.3 AAAAACTCCTTTACACCTTCTATGGTAAGGAGTCACTTGAGAACCCAACCTACATTTACC 1272

NC_004718.3 --------ATACGCTCTGTGTACCCTGTTGCATCTCCACAGGAGTGTAACAATATGCACT 1213

EPI_ISL_6640916_omicron --------ATTCGATCTGTCTATCCAGTTGCGTCACCAAATGAATGCAACCAAATGTGCC 1160

MW494315.1 --------ATTCGATCTGTCTATCCAGTTGCGTCACCAAATGAATGCAACCAAATGTGCC 1214

KF686346.1 ------------------------------------------------------------ 1287

NC_006213.1 ------------------------------------------------------------ 1397

NC_002645.1 ------------------------------------------------------------ 1075

JX504050.1 ------------------------------------------------------------ 1066

NC_019843.3 ACTCCGCATTCATTGAGTGTGGAAGTTGTGGTAATGATTCCTGGCTTACAGGGAATGCTA 1332

NC_004718.3 TGTCTACCTTGATGAAATGTAATCATTGCGATGAAGTTTCATGGCAGACGTGCGACTTTC 1273

EPI_ISL_6640916_omicron TTTCAACTCTCATGAAGTGTGATCATTGTGGTGAAACTTCATGGCAGACGGGCGATTTTG 1220

MW494315.1 TTTCAACTCTCATGAAGTGTGATCATTGTGGTGAAACTTCATGGCAGACGGGCGATTTTG 1274

KF686346.1 -----------------TGTGATTTTTATGGTTGGGTTTCAGGTAATATGATGGATGGTT 1330

NC_006213.1 -----------------TGTGATTTTAAAGGTTGGATTCCTGGTAACATGATAGATGGTT 1440

NC_002645.1 -----------------AAATCCTCTTGTTGCAACGTGATCAGTAATAAACTGTGTGTTG 1118

JX504050.1 -----------------TTATCTTCTTGTTGTGGCACACCTGCTAAGAAACTTTGTGTTG 1109

* * *

NC_019843.3 TCCAAGGGTTTGCCTGTGGATGTGGGGCATCATATACAGCTAATGATGTCGAAGTCCAAT 1392

NC_004718.3 TGAAAGCCACTTGTGAACATTGTGGCACTGAAAATTTAGTTATTGA---AGGACCTACTA 1330

EPI_ISL_6640916_omicron TTAAAGCCACTTGCGAATTTTGTGGCACTGAGAATTTGACTAAAGA---AGGTGCCACTA 1277

MW494315.1 TTAAAGCCACTTGCGAATTTTGTGGCACTGAGAATTTGACTAAAGA---AGGTGCCACTA 1331

KF686346.1 TTT------CTTGTCCATTGTGTTGTACAGTTTATGACTCTAGCGAAGTTAAAGCCCAAT 1384

NC_006213.1 TTG------CTTGCACCACTTGTGGTCATGTTTATGAAGTAGGTGATTTGATGGCACAAT 1494

NC_002645.1 TTC------CCGGTAA-------------------------------------------- 1128

JX504050.1 TTC------CTGGTAA-------------------------------------------- 1119

*

NC_019843.3 CATCTGGCATGATTAAGCCAAATGCTCTTCTTTGTGCTACTTGCCCC------------- 1439

NC_004718.3 CATGTGGGTACCTACCTACTAATGCTGTAGTGAAAATGCCATGTCCTGCCTGTCAAGACC 1390

EPI_ISL_6640916_omicron CTTGTGGTTACTTACCCCAAAATGCTGTTGTTAAAATTTATTGTCCAGCATGTCACAATT 1337

MW494315.1 CTTGTGGTTACTTACCCCAAAATGCTGTTGTTAAAATTTATTGTCCAGCATGTCACAATT 1391

KF686346.1 CATCTGGTGTTATTCCTGAAAATCCTGTGTTATTTACTAATAGTAC-------------- 1430

NC_006213.1 CTTCAGGTGTTTTGCCTGTTAACCCTGTATTGCATACTAAGAGTGCA------------- 1541

NC_002645.1 -------TGTTAAACCTGGTGATGCTGTGATTACCACTCAGCAAGC-------------- 1167

JX504050.1 -------TGTCGTTCCTGGTGATGTGATCATCACCTCAACTAGTGC-------------- 1158

* * * *

NC_019843.3 ------------------------------------------------------------ 1439

NC_004718.3 CAGAGATTGGACCTGAGCATAGTGTTGCAGATTATCACAACCACTCAAACATTGAAACTC 1450

EPI_ISL_6640916_omicron CAGAAGTAGGACCTGAGCATAGTCTTGCCGAATACCATAATGAATCTGGCTTGAAAACCA 1397

MW494315.1 CAGAAGTAGGACCTGAGCATAGTCTTGCCGAATACCATAATGAATCTGGCTTGAAAACCA 1451

KF686346.1 ------------------------------------------------------------ 1430

NC_006213.1 ------------------------------------------------------------ 1541

NC_002645.1 ------------------------------------------------------------ 1167

JX504050.1 ------------------------------------------------------------ 1158

NC_019843.3 --TTTGCTAAGGGTGATAGCTGTTCTTCTAATTGCAAACATTCAGTTGCTCAGTTGGTTA 1497

NC_004718.3 GACTCCGCAAGGGAGGTAGG---------------------------------------- 1470

EPI_ISL_6640916_omicron TTCTTCGTAAGGGTGGTCGC---------------------------------------- 1417

MW494315.1 TTCTTCGTAAGGGTGGTCGC---------------------------------------- 1471

KF686346.1 -------------TGATACTGTTAACCATGATTCTTTTAATTTGTATGGTTATTCTGTCA 1477

NC_006213.1 --GCAGGCTATGGTGGTTTTGGTTGTAAAGATTCTTTTACTCTGTATGGCCAAACTGTAG 1599

NC_002645.1 -------------TGGTGCTGGTATTAAGTATTTTTGTGGCATGACTCTTAAGTTTGTTG 1214

JX504050.1 -------------TGGTTGTGGTGTTAAATACTATGCTGGCTTAGTTGTTAAACATATTA 1205

* *

NC_019843.3 GTTACCTTTCTGAACGCTGTAATGTTATTGCTGATTCTAAGTCCTTCACACTTATCTTTG 1557

NC_004718.3 -----------------------------------------------ACTAGATGTTTTG 1483

EPI_ISL_6640916_omicron -----------------------------------------------ACTATTGCCTTTG 1430

MW494315.1 -----------------------------------------------ACTATTGCCTTTG 1484

KF686346.1 CACCATTTGGTTCTTGTATATATTGGTCGCCGCGTCCTGGATTGTGGATTCCTATAATTA 1537

NC_006213.1 TTTATTTTGGAGGTTGTGTGTATTGGAGTCCAGCACGTAATATATGGATTCCTATATTAA 1659

NC_002645.1 CAAATATTGAAGGTGTCTCTGTTTGGAGAGTGATTGCTCTTCAGAGTGTGGATTGCTTTG 1274

JX504050.1 CTAACATTACTGGTGTGTCTTTATGGCGTGTTACAGCTGTTCATTCTGATGGAATGTTTG 1265

*

NC_019843.3 GTGGCGTAGCTT------------------------------------------------ 1569

NC_004718.3 GAGGCTGTGTGT------------------------------------------------ 1495

EPI_ISL_6640916_omicron GAGGCTGTGTGT------------------------------------------------ 1442

MW494315.1 GAGGCTGTGTGT------------------------------------------------ 1496

KF686346.1 AATCTTCAGTCAAGTCTTATGATGATTTGGTTTATTCAGGTGTAGTAGGTTGTAAATCTA 1597

NC_006213.1 AATCCTCTGTTAAGTCATATGACAGTTTGGTTTATACTGGAGTTTTAGGTTGCAAGGCTA 1719

NC_002645.1 TTGCTTCTTCCA---------------------------------------------CTT 1289

JX504050.1 TGGCATCATCTT---------------------------------------------CTT 1280

NC_019843.3 ---------------------------------ACGCCTACTTTGGATGTGAGGAAGGTA 1596

NC_004718.3 ---------------------------------TTGCCTATGTTGGCTGCTATAATAAGC 1522

EPI_ISL_6640916_omicron ---------------------------------TCTCTTATGTTGGTTGCCATAACAAGT 1469

MW494315.1 ---------------------------------TCTCTTATGTTGGTTGCCATAACAAGT 1523

KF686346.1 TTGTTAAAGAAACTGCTCTTATTACTCATGCACTTTACTTAGATTATGTTCAATGTAAGT 1657

NC_006213.1 TTGTAAAGGAAACAAATCTCATTTGCAAAGCTTTGTACCTTGATTATGTTCAACACAAGT 1779

NC_002645.1 TTGTAGAAGAGGAACATGTTAATAGAATGGATACATTCTGCTTCAATGTACGCAATAGTG 1349

JX504050.1 ATGATGCACTCTTGCATAGAAATTCATTAGACCCTTTTTGCTTTGATGTTAACACTTTAC 1340

NC_019843.3 CTATGTACTTTGTGCCTAGAGCTAAGTCTGTTGTCTCAAGGATTGGAGACTCCATCTTTA 1656

NC_004718.3 GTGCCTACTGGGTTCCTCGTGCTAGTGCTGATAT------------TGGCTCAGGCCATA 1570

EPI_ISL_6640916_omicron GTGCCTATTGGGTTCCACGTGCTAGCGCTAACAT------------AGGTTGTAACCATA 1517

MW494315.1 GTGCCTATTGGGTTCCACGTGCTAGCGCTAACAT------------AGGTTGTAACCATA 1571

KF686346.1 GTGGTAATCTTGAACAAAATCATATTCTTGGCGTTAATAATTCTTGGTGTAGGCAACTGT 1717

NC_006213.1 GTGGCAATTTACACCAACGGGAGTTGCTAGGTGTTTCAGATGTGTGGCATAAACAATTGC 1839

NC_002645.1 TTACTGATGAGTGTCGTCTGGCCATGTTGGGTGCTGAAATGACTAGTAATGTCAGAAGAC 1409

JX504050.1 TTTCTAATCAATTACGTCTAGCTTTTCTTGGTGCTTCTGTTACAGAAGATGTTAAATTTG 1400

* * *

NC_019843.3 CAGGCTGTACTGG---CTCTTGGAACAAGGTCACTCAAATTGCTAACATGTTCTTGGAAC 1713

NC_004718.3 CTGGCATTACTGGTGACAATGTGGAGACCTTGAATGAGGATCTCCTTGAGATACTGAGTC 1630

EPI_ISL_6640916_omicron CAGGTGTTGTTGGAGAAGGTTCCGAAGGTCTTAATGACAACCTTCTTGAAATACTCCAAA 1577

MW494315.1 CAGGTGTTGTTGGAGAAGGTTCCGAAGGTCTTAATGACAACCTTCTTGAAATACTCCAAA 1631

KF686346.1 TGCTTAATAGAGGTGATTATAATATGCTTCTAAAAAATATTGACTTGTTTGTTAAGCGTC 1777

NC_006213.1 TATTAAATAGAGGTGTTTATAAACCTCTGTTAGAGAATATTGATTATTTTAATATGCGGC 1899

NC_002645.1 AAGTTGCTTCAGGTGTCATAGACATTAGTACCGGTTGGTTTGATGTTTATGATG------ 1463

JX504050.1 CTGCTAGCACTGGTGTTATTGACATTAGTGCTGGTATGTTTGGTCTTTACGATG------ 1454

**

NC_019843.3 AGACTCAGCATTCCCTTAACTTTGTGGGAGAGTTCGTTGTCAACGATGTTGTCCTC---- 1769

NC_004718.3 GTGAACGTGTTAACATTAACATTGTTGGCGATTTTCATTTGAATGAAGAGGTTGCCATCA 1690

EPI_ISL_6640916_omicron AAGAGAAAGTCAACATCAATATTGTTGGTGACTTTAAACTTAATGAAGAGATCGCCATTA 1637

MW494315.1 AAGAGAAAGTCAACATCAATATTGTTGGTGACTTTAAACTTAATGAAGAGATCGCCATTA 1691

KF686346.1 GTGCTGATTTTGCTT---GCAAGTTTGCAGTTTGTGGAG---ATGGTTTTGTACCTTTTT 1831

NC_006213.1 GCGCTAAATTTAGTTTAGAAACTTTTACTGTTTGTGCAG---ATGGCTTTATGCCTTTTC 1956

NC_002645.1 ------------------ACATCTTTGCTGAAAGCAAAC---CATGGTTTGTTCGCAAGG 1502

JX504050.1 ------------------ACATATTGACAAACAATAAAC---CTTGGTTTGTACGCAAAG 1493

* *

NC_019843.3 -----GCAATTCTCTCTGGAACCACAACTAATGTTGACAAAATACGCCAGCTTCTCAAAG 1824

NC_004718.3 TTTTGGCATCTTTCTCTGCTTCTACAAGTGCCTTTATTGACACTATAAAGAGTCTTGATT 1750

EPI_ISL_6640916_omicron TTTTGGCATCTTTTTCTGCTTCCACAAGTGCTTTTGTGGAAACTGTGAAAGGTTTGGATT 1697

MW494315.1 TTTTGGCATCTTTTTCTGCTTCCACAAGTGCTTTTGTGGAAACTGTGAAAGGTTTGGATT 1751

KF686346.1 TACTAGATGGTTTAATTCCCCGTAGTTATTATCTAATTCAGAGTGGTATTTTCTTTACAT 1891

NC_006213.1 TTTTAGATGATTTAGTTCCACGCGCATATTATTTGGCAGTAAGTGGTCAAGCATTTTGTG 2016

NC_002645.1 CTGAAGACATTTTTGGCCCTTGTTGGTCCGCTCTTGCTTCTGCACTTAAACAACTTAAAG 1562

JX504050.1 CTTCTGGGCTTTTTGATGCAATCTGGGATGCTTTTGTTGCCGCTATTAAGCTTGTACCAA 1553

* * * * *

NC_019843.3 GTGTCACCCTTGACAAGTTGCGTGATTATTTAGCTGACTATGACGTAGCAGTCACTGCCG 1884

NC_004718.3 ACAAGTCTTTCAAAACCATTGTTGAGTCCTGCGGTAACTATAAAGTTACC---------- 1800

EPI_ISL_6640916_omicron ATAAAGCATTCAAACAAATTGTTGAATCCTGTGGTAATTTTAAAGTTACA---------- 1747

MW494315.1 ATAAAGCATTCAAACAAATTGTTGAATCCTGTGGTAATTTTAAAGTTACA---------- 1801

KF686346.1 CTTTGATGTCTCAA---------------------------------------------- 1905

NC_006213.1 ATTATGCAGATAAA---------------------------------------------- 2030

NC_002645.1 TCACTACAGGTGAA---------------------------------------------- 1576

JX504050.1 CTACTACTGGTGTT---------------------------------------------- 1567

NC_019843.3 GCCCATTCATGGATAATGCTATTAATGTTGGTGGTACA--GGATTACAGTATGCCGCCAT 1942

NC_004718.3 -------AAGGGAAAGCCCGTAAAAGGTGCTTGGAACATTGGACAACAGAGATCAGTTTT 1853

EPI_ISL_6640916_omicron -------AAAGGAAAAGCTAAAAAAGGTGCCTGGAATATTGGTGAACAGAAATCAATACT 1800

MW494315.1 -------AAAGGAAAAGCTAAAAAAGGTGCCTGGAATATTGGTGAACAGAAATCAATACT 1854

KF686346.1 ------------------------------------------------------------ 1905

NC_006213.1 ------------------------------------------------------------ 2030

NC_002645.1 ------------------------------------------------------------ 1576

JX504050.1 ------------------------------------------------------------ 1567

NC_019843.3 TACTGCACCTTATGTAGTTCTCACTGGCTTAGGTGAGTCCTTTAAGAAAGTTGCAACCAT 2002

NC_004718.3 AACACCACTGTGTGGT-------------------------------------------- 1869

EPI_ISL_6640916_omicron GAGTCCTCTTTATGCA-------------------------------------------- 1816

MW494315.1 GAGTCCTCTTTATGCA-------------------------------------------- 1870

KF686346.1 ------------------------------------------------------------ 1905

NC_006213.1 ------------------------------------------------------------ 2030

NC_002645.1 ------------------------------------------------------------ 1576

JX504050.1 ------------------------------------------------------------ 1567

NC_019843.3 ACCGTATAAGGTTTGCAACTCTGTTAAGGATACTCTGGCTTATTATGCTCACAGCGTGTT 2062

NC_004718.3 ------------------------------------------------------------ 1869

EPI_ISL_6640916_omicron ------------------------------------------------------------ 1816

MW494315.1 ------------------------------------------------------------ 1870

KF686346.1 ------------------------------------------------------------ 1905

NC_006213.1 ------------------------------------------------------------ 2030

NC_002645.1 ------------------------------------------------------------ 1576

JX504050.1 ------------------------------------------------------------ 1567

NC_019843.3 GTACAGAGTTTTTCCTTATGACATGGATTCTGGTGTGTCATCCTTTAGTGAACTACTTTT 2122

NC_004718.3 ----------TTTCCCTCACAGGCTGCTGGTGTTATCAGATCAATTTTTGCGCGCACACT 1919

EPI_ISL_6640916_omicron ----------TTTGCATCAGAGGCTGCTCGTGTTGTACGATCAATTTTCTCCCGCACTCT 1866

MW494315.1 ----------TTTGCATCAGAGGCTGCTCGTGTTGTACGATCAATTTTCTCCCGCACTCT 1920

KF686346.1 ----------TTTTCACAAGAAGTTTCTGATATGTGTTTAAAAATGTGTATTTTGTTTAT 1955

NC_006213.1 ----------CTTTGCCATGCCGTTGTGTCTAAGAGTAAAGAGTTACTTGATGTGTCTCT 2080

NC_002645.1 ----------CTTGTGAGATTTGTTAAGTCTATTTGCAATTCAGCTGTTGCTGTCGTG-- 1624

JX504050.1 ----------TTGGTTAGGTTTGTTAAGTCTATTGCTTCAACTGTTTTAACTGTCTCT-- 1615

* *

NC_019843.3 TGATTGCGTTGATCTTTCAGTAGCTTCTACCTATTTTTTAGTCCGCATCTTGCAAGATAA 2182

NC_004718.3 TGATGCAGCAAACCACTCAATTCCTGAT-------------------------------- 1947

EPI_ISL_6640916_omicron TGAAACTGCTCAAAATTCTGTGCGTGTT-------------------------------- 1894

MW494315.1 TGAAACTGCTCAAAATTCTGTGCGTGTT-------------------------------- 1948

KF686346.1 GGACAGAGTTTCAGTTGCTACATTTTATATAGAGCATTATGTTAATAGGTTGGTTACTCA 2015

NC_006213.1 GGATTCTTTAGGTGCAGCTATACATTATTTGAATTCTAAGATTGTTGATTTGGCTCAACA 2140

NC_002645.1 ----------GGTGGTACTATACAAATTCTCGCTAGTGTGCCTGAGAAGTTTTTGAATGC 1674

JX504050.1 ----------AATGGTGTTATTATTATGTGTGCAGATGTTCCAGATGCTTTTCAATCAGT 1665

NC_019843.3 GACTGGCGACTTTATGTCTACAATTATTACTTCCTGCCAAACTGCTGTTAGTAAGCTTCT 2242

NC_004718.3 ----------------------------------TTGCAAAGAGCAGCTGTCACCATACT 1973

EPI_ISL_6640916_omicron ----------------------------------TTACAGAAGGCCGCTATAACAATACT 1920

MW494315.1 ----------------------------------TTACAGAAGGCCGCTATAACAATACT 1974

KF686346.1 ATTTAAGTTATTGGGTACTACACTTGTTAATAAAATGGTTAATTGGTTTAATACCATGTT 2075

NC_006213.1 TTTTAGTGATTTTGGAACAAGTTTCGTTTCTAAAATTGTTCATTTCTTTAAGACTTTTAC 2200

NC_002645.1 GTTTGACGTGTTTGTCACAGCTATTCAAACTGTCTTTGACTGTGCTGTTGAAACTTGTAC 1734

JX504050.1 TTATCGCACATTTACACAAGCTATTTGTGCTGCATTTGATTTTTCTTTAGATGTATTTAA 1725

NC_019843.3 AGATACATGTT---TTGAAGCTACAGAAGCAACATTTAACTTCTTGTTAGATTTGGCAGG 2299

NC_004718.3 TGATGGTATTT---CTGAACAGTCATTACGTCTTGTCGACGCCATGGTTTATACTTCAGA 2030

EPI_ISL_6640916_omicron AGATGGAATTT---CACAGTATTCACTGAGACTCATTGATGCTATGATGTTCACATCTGA 1977

MW494315.1 AGATGGAATTT---CACAGTATTCACTGAGACTCATTGATGCTATGATGTTCACATCTGA 2031

KF686346.1 AGATGCTAGTGCACCTGCTACAGGCTGGCTTCTTTACCAATTATTGAATGGTCTTTTTGT 2135

NC_006213.1 TACTAGCACTGCTCTTGCATTTGCATGGGTTTTATTTCATGTTTTGCATGGTGCTTATAT 2260

NC_002645.1 TATTGCCGGTA---AAGCATTTGACAAGGTTTTTGACTATGTTTTGCTTGATAATGCGCT 1791

JX504050.1 AATTGGTGATG---TTAAATTTAAACGACTTGGTGATTATGTTCTTACTGAAAACGCTCT 1782

* * * *

NC_019843.3 ATTGTTCAGAATCTTTCTC----------------------------------------- 2318

NC_004718.3 CCTGCTCACCAACAGTGTCATTATT----------------------------------- 2055

EPI_ISL_6640916_omicron TTTGGCTACTAACAATCTAGTTGTA----------------------------------- 2002

MW494315.1 TTTGGCTACTAACAATCTAGTTGTA----------------------------------- 2056

KF686346.1 AGTATCTCAAGCCAACTTTAATTTT----------------------------------- 2160

NC_006213.1 AGTAGTGGAGAGTGATATATATTTT----------------------------------- 2285

NC_002645.1 TGTAAAACTTGTCACCACAAAGCTTAAGGGTGTTCGTGAACGTGGCCTTAATAAAGTTAA 1851

JX504050.1 TGTTCGTTTGACTACTGAAGTTGTTCGTGGTGTTCGTGATGCTCGCATAAAGAAAGCCAT 1842

*

NC_019843.3 ------------------------------------------------------------ 2318

NC_004718.3 ------------------------------------------------------------ 2055

EPI_ISL_6640916_omicron ------------------------------------------------------------ 2002

MW494315.1 ------------------------------------------------------------ 2056

KF686346.1 -------------GTTGCTTTAATACCTGATTATGCTAAAATTTTAGTTAATAAATTTTA 2207

NC_006213.1 -------------GTTAAAAACATTCCTCGTTATGCTAGTGCTGTTGCACAAGCATTTCA 2332

NC_002645.1 GTATGCAACAGTTGTTGTTGGTTCCACTGAAGAAGTTAAATCTTCACGTGTTGAACGTAG 1911

JX504050.1 GTTTACTAAAGTAGTTGTAGGTCCTACAACTGAAGTTAAGTTTTCTGTTATTGAACTTGC 1902

NC_019843.3 -------------------------------------------------CGCAATGCCTA 2329

NC_004718.3 ----------------------------------ATGGCATATGTAACTGGTGGTCTTGT 2081

EPI_ISL_6640916_omicron ----------------------------------ATGGCCTACATTACAGGTGGTGTTGT 2028

MW494315.1 ----------------------------------ATGGCCTACATTACAGGTGGTGTTGT 2082

KF686346.1 CACTTTTTTTAAGTTATTATTAGAGTGTGTTACAGTTGATGTTTTAAAAGATATGCCTGT 2267

NC_006213.1 GAGTGTTGCTAAAGTTGTACTGGACTCTTTAAGAGTTACTTTTATTGATGGCCTTTCTTG 2392

NC_002645.1 CACTGCTGTACTTACAATCGCCAACAATTATTCCAAACTTTTTGATGAAGGGTATACTGT 1971

JX504050.1 CACTGTTAATTTGCGTCTTGTTGATTGTGCACCTGTAGTTTGCCCTAAAGGTAAGATTGT 1962

NC_019843.3 TGTGTACACTTCACAAGGGTTTGTGGTGGTCAATGGCAAAGTTTCTACACTTGTCAAACA 2389

NC_004718.3 ACAACAGACTTCTCAGTGGTTGTCTAATCTTTTGGGCACTACTGTTGAAAAACTCAGGCC 2141

EPI_ISL_6640916_omicron TCAGTTGACTTCGCAGTGGCTAACTAACATCTTTGGCACTGTTTATGAAAAACTCAAACC 2088

MW494315.1 TCAGTTGACTTCGCAGTGGCTAACTAACATCTTTGGCACTGTTTATGAAAAACTCAAACC 2142

KF686346.1 TCTTAAAACTATTAATGGTTTAGTTTGTATTGTAGGCAATAAGTTTTATAACGTTAGTAC 2327

NC_006213.1 TTTTAAGATTGGACGTAGAAGAATTTGTCTTTCAGGCAGAAAAATTTATGAAGTTGAGCG 2452

NC_002645.1 TGTAATTGGCGATGTGGCGTACTTTGTTAGTGACGGCTACTTCCGTCTTATGGCCAGTCC 2031

JX504050.1 TGTTATTGCTGGACAAGCTTTTTTCTATAGTGGTGGTTTTTATCGTTTTATGGTTGATCC 2022

** *

NC_019843.3 AGTGTTAGACTTGCTTAATAAG-------------------------------------- 2411

NC_004718.3 TATCTTTGAATGGATTGAGGCGAAACTTAGTGCAGGAGTTGAATTTCTCAAGGATGCTTG 2201

EPI_ISL_6640916_omicron CGTCCTTGATTGGCTTGAAGAGAAGTTTAAGGAAGGTGTAGAGTTTCTTAGAGACGGTTG 2148

MW494315.1 CGTCCTTGATTGGCTTGAAGAGAAGTTTAAGGAAGGTGTAGAGTTTCTTAGAGACGGTTG 2202

KF686346.1 A------GGGTTAATTCCTGGT-------------------------------------- 2343

NC_006213.1 T------GGCTTGTTACATTCA-------------------------------------- 2468

NC_002645.1 AAATAGTGTGTTGACTACTGCA-------------------------------------- 2053

JX504050.1 TACAACTGTATTAAATGATCCT-------------------------------------- 2044

* *

NC_019843.3 ------------------------------------------------------------ 2411

NC_004718.3 GGAGATTCTCAAATTTCTCATTACAGGTGTTTTTGACATCGTCAAGGGTCAAATACAGGT 2261

EPI_ISL_6640916_omicron GGAAATTGTTAAATTTATCTCAACCTGTGCTTGTGAAATTGTCGGTGGACAAATTGTCAC 2208

MW494315.1 GGAAATTGTTAAATTTATCTCAACCTGTGCTTGTGAAATTGTCGGTGGACAAATTGTCAC 2262

KF686346.1 ------------------------------------------------------------ 2343

NC_006213.1 ------------------------------------------------------------ 2468

NC_002645.1 ------------------------------------------------------------ 2053

JX504050.1 ------------------------------------------------------------ 2044

NC_019843.3 ---------GGTATGCAACTTTTGCATACAAAGGTCTCCTGGGCTGGTTCTAAAATCATT 2462

NC_004718.3 TGCTTCAGATAACATCAAGGATTGTGTAAAATGCTTCATTGATGTTGTTAACAAGGCACT 2321

EPI_ISL_6640916_omicron CTGTGCAAAGGAAATTAAGGAGAGTGTTCAGACATTCTTTAAGCTTGTAAATAAATTTTT 2268

MW494315.1 CTGTGCAAAGGAAATTAAGGAGAGTGTTCAGACATTCTTTAAGCTTGTAAATAAATTTTT 2322

KF686346.1 ------------------------------------------------------------ 2343

NC_006213.1 ------------------------------------------------------------ 2468

NC_002645.1 ------------------------------------------------------------ 2053

JX504050.1 ------------------------------------------------------------ 2044

NC_019843.3 GCTGTTATCTACAGCGGCAGGGAGTCTCTAATATTCCCATCGGGAACCTATTACTGTGTC 2522

NC_004718.3 CGAAATGTGCATTGATCAAGTCACTATCGCTGGCGCAAAGTTGCGATCACTCA------- 2374

EPI_ISL_6640916_omicron GGCTTTGTGTGCTGACTCTATCATTATTGGTGGAGCTAAACTTAAAGCCTTGA------- 2321

MW494315.1 GGCTTTGTGTGCTGACTCTATCATTATTGGTGGAGCTAAACTTAAAGCCTTGA------- 2375

KF686346.1 ------------------------------------------------------------ 2343

NC_006213.1 ------------------------------------------------------------ 2468

NC_002645.1 ------------------------------------------------------------ 2053

JX504050.1 ------------------------------------------------------------ 2044

NC_019843.3 ACCACTAAGGCTAAGTCCGTTCAACAAGATCTTGACGTTATTTTGCCTGGTGAGTTT--- 2579

NC_004718.3 ---ACTTAGGTGAAGTC--TTCATCGCTCAAAGCAAGGGACTTTACCGTCAGTGTATACG 2429

EPI_ISL_6640916_omicron ---ATTTAGGTGAAACA--TTTGTCACGCACTCAAAGGGATTGTACAGAAAGTGTGTTAA 2376

MW494315.1 ---ATTTAGGTGAAACA--TTTGTCACGCACTCAAAGGGATTGTACAGAAAGTGTGTTAA 2430

KF686346.1 --------------TTTGTTTTACCATGTAATGCACAGG--------------------- 2368

NC_006213.1 --------------TCCCAATTGCCATTAGATGTTTATGATTTAACC---ATGCCTA--- 2508

NC_002645.1 --------------GTCTATAAACCATTGTTTGCTTTTAATGTGAATGTTATGGGTA--- 2096

JX504050.1 --------------GTTTTTACTGGTGATTTATTCTACACTATTAAGTTTAGTGGTT--- 2087

NC_019843.3 -TCCAAGAAGCAGTTAGGACTGCTCCAACCTACTGACAATTCTACAACTGTTAGTGTTAC 2638

NC_004718.3 TGGCAAGGAGCAGCTGCAACTACTCATGCCTCTTAAGGCACCAAAAGAAGTAACCTTTCT 2489

EPI_ISL_6640916_omicron ATCCAGAGAAGAAACTGGCCTACTCATGCCTCTAAAAGCCCCAAAAGAAATTATCTTCTT 2436

MW494315.1 ATCCAGAGAAGAAACTGGCCTACTCATGCCTCTAAAAGCCCCAAAAGAAATTATCTTCTT 2490

KF686346.1 -----------------------------------------AACAACAAATTTATTTTTT 2387

NC_006213.1 -----------------------GTCAAGTTCAGAAAGCCAAGCAAAAACCTATTTATTT 2545

NC_002645.1 -----------------------CTAGACCTGAAAAATT------------------TCC 2115

JX504050.1 -----------------------TTAAGCTTGATGGTTTTAACCATCAGTTTGTTACTGC 2124

NC_019843.3 TG------------------------TATCCAGTAACATGGTTGAAACTGTTGTGGGTCA 2674

NC_004718.3 TGAAGGTGATTCACATGACACAGTACTTACCTCTGAGGAGGTTGTTCTCAAGAACGGTGA 2549

EPI_ISL_6640916_omicron AGAGGGAGAAACACTTCCCACAGAAGTGTTAACAGAGGAAGTTGTCTTGAAAACTGGTGA 2496

MW494315.1 AGAGGGAGAAACACTTCCCACAGAAGTGTTAACAGAGGAAGTTGTCTTGAAAACTGGTGA 2550

KF686346.1 TGAAGGCGTTGCAGAATCTGTTATAGTAGAAGATGATGTTATTGAGAATGTCAAATCTTC 2447

NC_006213.1 AAAAGGTTCTGGTTCTGATTTTTCATTAGCGGATAGTGTAGTTGAAGTTGTTACAACTTC 2605

NC_002645.1 AACCACTGTGACTTGTGAAAATTTAGAGTCTGCTGTTTTGTTTGTTAATGACAAAATTAC 2175

JX504050.1 TAGTTCTGCTACAGATGCCATTATTGCTGTTGAGCTGTTGTTATTGGATTTTAAAACTGC 2184

* *

NC_019843.3 ACTTGAGCAAACTAATATGCATAGTCCTGATGTTATAGTAGGTGACTATGTCATTA---- 2730

NC_004718.3 ACTCGAAGCACTCGAGACGCCCGTTGATAGCTTCACAAATGGAGCTATCGTTGGCACACC 2609

EPI_ISL_6640916_omicron TTTACAACCATTAGAACAACCTACTAGTGAAGCTGTTGAAGCTCCATTGGTTGGTACACC 2556

MW494315.1 TTTACAACCATTAGAACAACCTACTAGTGAAGCTGTTGAAGCTCCATTGGTTGGTACACC 2610

KF686346.1 TTTATCA----------------------------------------------------- 2454

NC_006213.1 ACTTACA----------------------------------------------------- 2612

NC_002645.1 TGAATTCCAATTGGATTACAGTATTGATGTCATTGATAATGAAATAATTGTCAAAC---- 2231

JX504050.1 AGTTTTT------GTGTACACATGTGTGGTTGATGGCTGTAGTGTCATTGTTAGAC---- 2234

NC_019843.3 --TTAGTGAAAAATTGTTTGTGCGTAGTAAGGAAGAAGACGGATTTGCCTTCTACC---- 2784

NC_004718.3 AGTCTGTGTAAATGGCCTCATGCTCTTAGAGATTAAGGACAAAGAACAATACTGCG---- 2665

EPI_ISL_6640916_omicron AGTTTGTATTAACGGGCTTATGTTGCTCGAAATCAAAGACACAGAAAAGTACTGTG---- 2612

MW494315.1 AGTTTGTATTAACGGGCTTATGTTGCTCGAAATCAAAGACACAGAAAAGTACTGTG---- 2666

KF686346.1 ------------------------------------------------------------ 2454

NC_006213.1 ------------------------------------------------------------ 2612

NC_002645.1 --CTAATATCAGCCTATGTGTTCCACTTTATGTGAGAGACTATGTTGACAAATGGGATGA 2289

JX504050.1 --GTGATGCTACATTCGCTACACATGTGTGTTTTAAGGACTGTTATAATGTTTGGGAGCA 2292

NC_019843.3 ------------------------------------------------------------ 2784

NC_004718.3 ------------------------------------------------------------ 2665

EPI_ISL_6640916_omicron ------------------------------------------------------------ 2612

MW494315.1 ------------------------------------------------------------ 2666

KF686346.1 ------------------------------------------------------------ 2454

NC_006213.1 ------------------------------------------------------------ 2612

NC_002645.1 TTTTTGCAGACAATATAGTAACGAGTCTTGGTTTGAGGATGATTACAGGGCTTTTATCAG 2349

JX504050.1 ATTCTGCATTGATAATTGTGGTGAGCCATGGTTTTTGACTGATTATAATGCTATCTTGCA 2352

NC_019843.3 ------------------------------------------------------------ 2784

NC_004718.3 ------------------------------------------------------------ 2665

EPI_ISL_6640916_omicron ------------------------------------------------------------ 2612

MW494315.1 ------------------------------------------------------------ 2666

KF686346.1 ----------------------------------------------TCTTATGAGTATTG 2468

NC_006213.1 ----------------------------------------------CCATGTGGTTATTC 2626

NC_002645.1 TGTTTTGGACATCACTGATGCTGCTGTGAAAGCTGCAGAGTCTAAAGCTTTCGTTGATAC 2409

JX504050.1 GAGTAATAACCCTCAATGTGCTATTGTTCAAGCATCAGAGTCTAAAGTTTTGCTTGAGAG 2412

NC_019843.3 -----------CTGCTTGCACTAATGGTCATGCTGTACCGACTCTCTTTAGACTTAAGGG 2833

NC_004718.3 -----------CATTGTCTCCTGGTTTACTGGCTACAAACAATGTCTTTCGCTTAAAAGG 2714

EPI_ISL_6640916_omicron -----------CCCTTGCACCTAATATGATGGTAACAAACAATACCTTCACACTCAAAGG 2661

MW494315.1 -----------CCCTTGCACCTAATATGATGGTAACAAACAATACCTTCACACTCAAAGG 2715

KF686346.1 TCAACCACCTAAATCTGTAGAAAAAATTTGTATTATAGATAATATGTACATGGGTAAGTG 2528

NC_006213.1 TGAACCACCTAAAGTTGCAGCTAAAATTTGCATTGTGGATAATGTTTATATGGCCAAGGC 2686

NC_002645.1 TATTGTTCCACCTTGCCCATCTATTTTGAAAGTTATAGATGGAGGCAAAATATGGAATGG 2469

JX504050.1 GTTTTTACCTAAGTGTCCTGAAATACTGTTGAGTATTGATGATGGCCATTTATGGAATCT 2472

**

NC_019843.3 AGGTGCACCTGTAAAAAAAGTAGCCTTTGGCGGTGATCAAGTACATGAGGTTGCTGCTGT 2893

NC_004718.3 GGGTGCACCAATTAAAGGTGTAACCTTTGGAGAAGATACTGTTTGGGAAGTTCAAGGTTA 2774

EPI_ISL_6640916_omicron CGGTGCACCAACAAAGGTT---ACTTTTGGTGATGACACTGTGATAGAAGTGCAAGGTTA 2718

MW494315.1 CGGTGCACCAACAAAGGTT---ACTTTTGGTGATGACACTGTGATAGAAGTGCAAGGTTA 2772

KF686346.1 TGGTGATAAATTTTTCCCTATTGTCATGAATGATAAAAATATTTGTCTTTTAGATCAGGC 2588

NC_006213.1 TGGTGACAAATATTACCCTGTTG---TGGTTGATGATCATGTTGGACTCTTGGATCAAGC 2743

NC_002645.1 TGTTATTAAAAATGTTAACTCTG------------------------------------- 2492

JX504050.1 TTTTGTTGAAAAGTTTACTTTTG------------------------------------- 2495

*

NC_019843.3 AAGAAGTGTTACTGTCGAGTACAACATTCATGCTGTATTAGACACACTACTTGCTTCTTC 2953

NC_004718.3 CAAGAATGTGAGAATCACATTTGAGCTTGATGAACGTGTTGACAAAGTGCTTAATGAAAA 2834

EPI_ISL_6640916_omicron CAAGAGTGTGAATATCACTTTTGAACTTGATGAAAGGATTGATAAAGTACTTAATGAGAG 2778

MW494315.1 CAAGAGTGTGAATATCACTTTTGAACTTGATGAAAGGATTGATAAAGTACTTAATGAGAA 2832

KF686346.1 TTGGCGTTTTCCATGTGCAGGTAGAAAAGTTAATTTTAACGAGAAACCTGTTGTTATGGA 2648

NC_006213.1 ATGGAGAGTTCCTTGTGCTGGAAGGCGTGTTACATTTAAGGAACAGCCTACAGTAAAGGA 2803

NC_002645.1 --------------TTAGAGACTGGCTTAAGTCTTTGAAGTTAAATCTCACACAACAGGG 2538

JX504050.1 --------------TTACAGATTGGTTAAAAACTCTTAAGCTTACACTTACTTCTAATGG 2541

NC_019843.3 TAGTCTTAGAACCTTTG------------------------------------TTGTAGA 2977

NC_004718.3 GTGCTCT---GTCTACA------------------------------------CTGTTGA 2855

EPI_ISL_6640916_omicron GTGCTCT---GCCTATA------------------------------------CAGTTGA 2799

MW494315.1 GTGCTCT---GCCTATA------------------------------------CAGTTGA 2853

KF686346.1 GATTCCGTCTT----TG---ATGACAGTTAAGGTTATGTTTGATTTAGATT--CTACTTT 2699

NC_006213.1 GATTATAAGCA----TGCCTAAGATTATTAAGGTTTTTTATGAGCTTGACA--ACGATTT 2857

NC_002645.1 TTTGCTTGGAACATGTGCAAAGCGTTTTAAACGTTGGCTTGGCATTTTGCTAGAGGCCTA 2598

JX504050.1 TCTTTTAGGTAATTGTGCCAAACGTTTTAGACGTGTTTTGGTAAAATTGCTTGATGTCTA 2601

NC_019843.3 TAAGTCTTTGTCAATTGAGGAGTTTGCTGACGTAGTAAAGGAACAAGTCTCAGACTTGCT 3037

NC_004718.3 ATCCGGTACCGAAGTTACTGAGTTTGCATGTGTTGTAGCAGAGGCTGTTGTGAAGACTTT 2915

EPI_ISL_6640916_omicron ACTCGGTACAGAAGTAAATGAGTTCGCCTGTGTTGTGGCAGATGCTGTCATAAAAACTTT 2859

MW494315.1 ACTCGGTACAGAAGTAAATGAGTTCGCCTGTGTTGTGGCAGATGCTGTCATAAAAACTTT 2913

KF686346.1 TGATGATATTTTAGGTAAAGTTTGTTCAGAATTTGAAGTAGAAAAGGGTGTTACTGTAGA 2759

NC_006213.1 TAATACTATTTTAAATACTGCGTGTGGAGTGTTTGAAGTGGATGATACTGTTGATATGGA 2917

NC_002645.1 TAATGCGTTTTTAGACACTGTGGTTTC------TACTGTTAAAATTGGTGGCTTGACCTT 2652

JX504050.1 TAATGGTTTTCTTGAAACTGTCTGTAG------TGTCGCATACACTGCTGGTGTTTGCAT 2655

* *

NC_019843.3 TGTTAAATTACTGCGTGGAATGCCGATTCCAGATTTTGATTTAGACGATTTTATTGACGC 3097

NC_004718.3 ACAACCAGTTTCTGATCTCCTTACCAACATGGGTATTGATCTTGATGAGTGGAGTGTAGC 2975

EPI_ISL_6640916_omicron GCAACCAGTATCTGAATTACTTACACCACTGGGCATTGATTTAGATGAGTGGAGTATGGC 2919

MW494315.1 GCAACCAGTATCTGAATTACTTACACCACTGGGCATTGATTTAGATGAGTGGAGTATGGC 2973

KF686346.1 TGATTTTGTTGCTGTTGTTTGTGATGCTATAGAGAATGCTTTAAACTCTTGTAAAGAGCA 2819

NC_006213.1 GGAATTTTATGCTGTGGTGATTGATGCCATAGAAGAGAAACTTTCTCCATGTAAGGAGCT 2977

NC_002645.1 TAAAACATATGCTTTTGATAAACCTTACATTGTGATACGTGATATCGTGTGTAAGGTTGA 2712

JX504050.1 CAAATATTATGCTGTTAATGTTCCATATGTAGTTATTAGTGGTTTTGTAAGTCGTGTAAT 2715

*

NC_019843.3 ACCA-----------------------------------------------------TGC 3104

NC_004718.3 TACA-----------------------------------------------------TTC 2982

EPI_ISL_6640916_omicron TACA-----------------------------------------------------TAC 2926

MW494315.1 TACA-----------------------------------------------------TAC 2980

KF686346.1 TCCAGTGGTTGGTTATCAA--GTTCGTGCATTTTTAAATAAACTTAATGATAATGTTGTT 2877

NC_006213.1 TGAAGGTGTAGGTGCTAAA--GTTAGTGCCTTTTTACAGAAATTAGAGGATAATCCCCTA 3035

NC_002645.1 AAATAAAACAGAAGCAGAATGGATTGAGCTTTTTCCACATAATGACAGGATTAAGTCTTT 2772

JX504050.1 TCGTAGAGAAAGGTGTGACATGACTTTTCCTTGTGTTAGTTGTGTCACCTTT-----TTC 2770

NC_019843.3 TATTGCTTTAACGCTGAGGGTGATGCATCCTGGTCTTCTACTATGATCTTCTCTCTTCAC 3164

NC_004718.3 TACTTATTTGATGATGCTGGTGAAGAAAACTTTTCATCACGTATGTATTGTTCCTTTTAC 3042

EPI_ISL_6640916_omicron TACTTATTTGATGAGTCTGGTGAGTTTAAATTGGCTTCACATATGTATTGTTCTTTTTAC 2986

MW494315.1 TACTTATTTGATGAGTCTGGTGAGTTTAAATTGGCTTCACATATGTATTGTTCTTTTTAC 3040

KF686346.1 TATTTATTTGATGAGGCTGGTGATGAAGCAATGGCCTCTCGTATGTATTGTACTTTT--- 2934

NC_006213.1 TTTTTATTTGATGAGGCTGGCGAGGAAGTTCTTGCTCCTAAATTGTATTGTGCCTTTACA 3095

NC_002645.1 TAGTACTTTCGAGAGTGCTTACATGCCAATTGCAGACCCTACACATTTT----------- 2821

JX504050.1 TATGAATTTTTAGACACTTGTTTTGGTGTTAGTAAACCTAATGCC-ATT----------- 2818

* *** * * *

NC_019843.3 CCCGTCGAGTGTGACGAGGAGTGTTCTGAA---GTAGAGGCTTCAGATTTAGAAGAAGGT 3221

NC_004718.3 CCTCCAGATGAGGA------------AGAA---GAGGACGATGCAGAGTGTGAGGAAGAA 3087

EPI_ISL_6640916_omicron CCTCCAGATGAGGA------------TGAA---GAAGAAGGT---GATTGTGAAGAAGAA 3028

MW494315.1 CCTCCAGATGAGGA------------TGAA---GAAGAAGGT---GATTGTGAAGAAGAA 3082

KF686346.1 GCTATTGAGGATGT------------TGAAGACGTTATCAGTAGTGAAGCTGTCGAAGAT 2982

NC_006213.1 GCTCCTGAAGATGA------------TGACTTTCTTGAGGAAAGTGATGTTGAAGAAGAT 3143

NC_002645.1 GACATTGAAGAAGT------------TGAA---CTTTTAGATGCAGAGTTTGTAGAACCA 2866

JX504050.1 GATGTTGAACATTT------------AGAG---CTTAAAGAAACTGTTTTTGTTGAACCT 2863

** ** * * ***

NC_019843.3 GAATCAGAGTGC------------------------ATTTCTGAGACTTCAACTGAACAA 3257

NC_004718.3 GAAATTGAT--------------------------------------------------- 3096

EPI_ISL_6640916_omicron GAGTTTGAG--------------------------------------------------- 3037

MW494315.1 GAGTTTGAG--------------------------------------------------- 3091

KF686346.1 ACTATTGATGGTATCGTTGAAGACACTATTAATGACGATGAAGATGTTGTTACTGGTGAC 3042

NC_006213.1 GATGTAGAAGGT------------------------------------------------ 3155

NC_002645.1 GGCTGTGGTGGTATTTTGGCAGTAATA---------GATGAGCACGTCTTTTATAAGAAG 2917

JX504050.1 AAGGATGGTGGTCAATTTTTTGTTTCT---------GGTGATTATCTTTGGTATGTTGTA 2914

*

NC_019843.3 GTTGACGTTTCTCATGAGACTTCT---------------GACGACGAGTGGGCTGCTGCA 3302

NC_004718.3 ---------------GAAACCTGT---------------GAACATGAGTACGGTACAGAG 3126

EPI_ISL_6640916_omicron ---------------CCATCAACT---------------CAATATGAGTATGGTACTGAA 3067

MW494315.1 ---------------CCATCAACT---------------CAATATGAGTATGGTACTGAA 3121

KF686346.1 AATGACGATGAAGATGTTGTTACTGGTGACAATGACGATGAAGATGTTGTTACTGGTGAC 3102

NC_006213.1 ------------------------------------------------------------ 3155

NC_002645.1 GATGGTGTTTATTATCCATCAAATGGTACTAACATTCTACCTGTTGCATTTACAAAAGCC 2977

JX504050.1 GATGACATTTATTATCCAGCTTCATGTAATGGTGTATTGCCAGTTGCTTTTACAAAATTA 2974

NC_019843.3 GTTGATGAAGCGTTCCCTCTCGATGAAGCAGAAGATGTTACTGAATCTGTGCAAGAAGAA 3362

NC_004718.3 GATGATTATCAAGGTCTCCCTCTGGAATTTGGTGCCTCAGCTGAAACAGTTCGAGTTGAG 3186

EPI_ISL_6640916_omicron GATGATTACCAAGGTAAACCTTTGGAATTTGGTGCCACTTCTGCTGCTCTTCAACCTGAA 3127

MW494315.1 GATGATTACCAAGGTAAACCTTTGGAATTTGGTGCCACTTCTGCTGCTCTTCAACCTGAA 3181

KF686346.1 AATGACGATGAAGATGTTGTTACTGGTGACAATGACGATGAAGATGTTGTTACTGGTGAC 3162

NC_006213.1 ------------------------------------------------------------ 3155

NC_002645.1 GCTGGTGGTAAAGTTTCATTTTCTGATGACGTTGAAGTAAAAGACATTGAACCTGTTTAC 3037

JX504050.1 GCAGGTGGTAAAATATCTTTTTCTGATGATGTTATAGTTCATGATGTTGAACCTACCCAT 3034

NC_019843.3 GCACAACCAGTAGAAGTACCTGTTGAAGATATTGCGCAG------GTTGTCATAGCTGAC 3416

NC_004718.3 GAAGAAGAAGAGGAAGACTGGCTGGATGATACTACTGAGCAATC---------------- 3230

EPI_ISL_6640916_omicron GAAGAGCAAGAAGAAGATTGGTTAGATGATGATAGTCAACAAACTGTTGGTCAACAAGAC 3187

MW494315.1 GAAGAGCAAGAAGAAGATTGGTTAGATGATGATAGTCAACAAACTGTTGGTCAACAAGAC 3241

KF686346.1 AATGACGATGAAGATGTTGTTACTGGTGACAATGACGATGAAGATGTTGTTACTGGTGAC 3222

NC_006213.1 ------------------------------------------------------------ 3155

NC_002645.1 AGAGTCAAGCTTTGCTTTGAGTTTGAAGATGAAAAACTTGTAGATGTTTGTGAAAAGGCA 3097

JX504050.1 AAAGTCAAGCTCATATTTGAGTTTGAAGATGATGTTGTTACCAGTCTTTGTAAGAAGAGT 3094

NC_019843.3 ACCTTACAGGAAACTC------CTGTTGTGCCTGATAC---------------------- 3448

NC_004718.3 ------------------------------------------------------------ 3230

EPI_ISL_6640916_omicron GGCAGTGAGGACAATCAGACAACTACTATTCAAACAAT---------------------- 3225

MW494315.1 GGCAGTGAGGACAATCAGACAACTACTATTCAAACAAT---------------------- 3279

KF686346.1 AATGACGATGAAGATGTTGTTACTGGTGACAATGACGA---------------TGAAGAT 3267

NC_006213.1 ------------------------------------------------------------ 3155

NC_002645.1 ATTGGCAAGAAAATTAAACATGAAGGTGACTGGGATAGCTTTTGTAAGACTATTCAATCA 3157

JX504050.1 TTTGGTAAGTCCATTATTTATACAGGTGATTGGGAAGGTCTACATGAAGTTCTTACATCT 3154

NC_019843.3 ------------------------------------------------------------ 3448

NC_004718.3 ------------------------------------------------------------ 3230

EPI_ISL_6640916_omicron ------------------------------------------------------------ 3225

MW494315.1 ------------------------------------------------------------ 3279

KF686346.1 GTTGTTACTGGTGACAATGACGATGAAGATGTTGTTACTGGTGACAATGACGATGAAGAT 3327

NC_006213.1 ------------------------------------------------------------ 3155

NC_002645.1 GCACTTTCTGTTGTTTCTTGCTATGTAAATCTACCTACTTATTACATTTATGATGAAGAA 3217

JX504050.1 GCAATGAATGTCATTGGGCAACATATTAAGTTGCCACAATTTTATATTTATGATGAAGAG 3214

NC_019843.3 ------------------------------------------------------------ 3448

NC_004718.3 ------------------------------------------------------------ 3230

EPI_ISL_6640916_omicron ------------------------------------------------------------ 3225

MW494315.1 ------------------------------------------------------------ 3279

KF686346.1 GTTGTTACTGGTGACAATGACGATGAAGATGTTGTTACTGGTGACAATGACGATGAAGAT 3387

NC_006213.1 ---------------------GAGGAAACTGATTTAACTGTCACAAGTGCTGGACAGCCT 3194

NC_002645.1 GGCGGT---------------AATGACTTGAGTTTGCCCGTTATGATTTCTGAATGGCCT 3262

JX504050.1 GGTGGT---------------TATGATGTTTCTAAACCAGTTATGATTTCACAATGGCCT 3259

NC_019843.3 --------------------------TGTTGAAGTCCCACCGCAAGTGGTGA---AACTT 3479

NC_004718.3 -----------------------------------------------AGAGATTGAGCCA 3243

EPI_ISL_6640916_omicron --------------------------TGTTGAGGTTCAACCTCAATTAGAGATGGAACTT 3259

MW494315.1 --------------------------TGTTGAGGTTCAACCTCAATTAGAGATGGAACTT 3313

KF686346.1 GTTGTTACTGGTGACAATGACGATGAAGATGTTGTTACTGGTGACAATGACGATGAAGAT 3447

NC_006213.1 TGTGTTGCTAGTGAACAGGAGGAGTCTTCTGAAGTCTTAGAGGACACTTTGGATGATGGT 3254

NC_002645.1 CTTTCTGTTCAACA------------AGCTCAACAAGAAGCTACTTTACCTGATATTGCT 3310

JX504050.1 ATTAGTAATGATAGTAATGGTTGTGTTGTTGAAGCGAGCACTGATTTTCATCAATTAGAA 3319

NC_019843.3 CCGTCTGCACCTCAGACTATCCAGCCCGAGGTAAAAGAAGTTGCACCTGTCTATGAGGCT 3539

NC_004718.3 GAACCAGAACCTACACCTGAAGAACCAGTTAATCAGTTTACTGGTTATTTAAAAC----- 3298

EPI_ISL_6640916_omicron ACACCAGTTGTTCAGACTATTGA---AGTGAATAGTTTTAGTGGTTATTTAAAAC----- 3311

MW494315.1 ACACCAGTTGTTCAGACTATTGA---AGTGAATAGTTTTAGTGGTTATTTAAAAC----- 3365

KF686346.1 GTTGTTACTGGTGACAATGACGATGAAGATGTTGTTACTGGTGACAATGACGATGAAGAT 3507

NC_006213.1 CCAAGTGTGGAGACATCTGATTCACAAGTTGAAGAAGATGTAGAAATGTCGGATTTTGTT 3314

NC_002645.1 GAGGATGTTGTTGACCAAGTTGAAGAAGTCAATAGCATTTTTGACATTGAGACAGTGGAT 3370

JX504050.1 TGTATTGTTGATGACTCTGTTAGAGAAGAGGTTGATATAATTGAACAACCTTTTGAAGAA 3379

* *

NC_019843.3 GATACCGAACAGACA--------------------------------------------- 3554

NC_004718.3 ------------------------------------------------------------ 3298

EPI_ISL_6640916_omicron ------------------------------------------------------------ 3311

MW494315.1 ------------------------------------------------------------ 3365

KF686346.1 GTTGTTACTGGTGAC--------------------------------------------- 3522

NC_006213.1 GATCTTGAATCTGTG--------------------------------------------- 3329

NC_002645.1 GTTAAACATGATGTG---------AGTCCTTTTGAAATGCCATTTGAAGAGTTAAATGGT 3421

JX504050.1 GTTGAACATGTGCTCTCAATTAAACAACCTTTTTCTTTTTCTTTTAGAGATGAATTGGGT 3439

NC_019843.3 ------------------------------------------------------------ 3554

NC_004718.3 ------------------------------------------------------------ 3298

EPI_ISL_6640916_omicron ------------------------------------------------------------ 3311

MW494315.1 ------------------------------------------------------------ 3365

KF686346.1 ------------------------------------------------------------ 3522

NC_006213.1 ------------------------------------------------------------ 3329

NC_002645.1 TTAAAGATACTCAAACAATTGGATAACAACTGCTGGGTTAACTCAGTTATGTTACAAATA 3481

JX504050.1 GTTCGTGTTTTAGATCAATCTGATAATAATTGTTGGATTAGTACCACACTTGTACAGTTG 3499

NC_019843.3 ------------------------------------------------------------ 3554

NC_004718.3 ------------------------------------------------------------ 3298

EPI_ISL_6640916_omicron ------------------------------------------------------------ 3311

MW494315.1 ------------------------------------------------------------ 3365

KF686346.1 ---------AATGACGATGAAGACAATAACGATGAAGAGATTGTTACTGGTGACAATGAT 3573

NC_006213.1 ---------ATTCAGGATTATGAAAATGTTTGTTTTGAGTTTTATACTACAGAGCCAGA- 3379

NC_002645.1 CAATTAACTGGTATACTTGATGGTGACTATGCTATGCAGTTTTTTAAAATGGGCCGAGTT 3541

JX504050.1 CAACTTACAAAGCTTTTGGATGATTCTATTGAGATGCAATTGTTTAAAGTTGGTAAAGTT 3559

NC_019843.3 ------------------------------------------------------------ 3554

NC_004718.3 ------------------------------------------------------------ 3298

EPI_ISL_6640916_omicron ------------------------------------------------------------ 3311

MW494315.1 ------------------------------------------------------------ 3365

KF686346.1 GACCAAATTGTTGTTACTGGTGATGATGTAGATGATATTGAAAGTATTTATGACTTTGAT 3633

NC_006213.1 ------------------------------------------------------------ 3379

NC_002645.1 GCCAAGATGATTGAACGCTGCTACACTGCTGAGCAATGTATACGTGGTGCTATGGGTGAT 3601

JX504050.1 GATTCAATTGTCCAAAAGTGTTATGAGTTGTCTCATTTAATTAGTGGTTCACTTGGTGAT 3619

NC_019843.3 ------------------------------------------------------------ 3554

NC_004718.3 ------------------------------------------------------------ 3298

EPI_ISL_6640916_omicron ------------------------------------------------------------ 3311

MW494315.1 ------------------------------------------------------------ 3365

KF686346.1 ACTTATAAAGCTCTTTTAGTTTTTAATGATGTCTATAATGATGCTTTGTTTGTTAGTTAT 3693

NC_006213.1 ------------------------------------------------------------ 3379

NC_002645.1 GTTGGTTTGTGTATGTATAGACTGCTTAAAGACTTACACACTGGTTTTATGGTTATGGAT 3661

JX504050.1 AGTGGTAAACTTCTTAGTGAACTTCTTAAAGAAAAATATACATGTTCTATAACTTTTGAG 3679

NC_019843.3 ------------------------------------------------------------ 3554

NC_004718.3 ------------------------------------------------------------ 3298

EPI_ISL_6640916_omicron ------------------------------------------------------------ 3311

MW494315.1 ------------------------------------------------------------ 3365

KF686346.1 GGTTCTAGTGTTGAAACAGAAACATATTTTAAAGTTAATGGTTTATGGTCACCTACTATT 3753

NC_006213.1 -----------------------ATTTGTTAAAGTTTTGGGTCTGTATGTGCCTAAAGCA 3416

NC_002645.1 TATAAATGTAGTTGTACCAGTGGTAGGCTTGAAGAATCGGGAGCTGTTTTGTTTTG---T 3718

JX504050.1 ATGTCTTGTGATTGT---GGTAAAAAGTTTGATGATCAGGTTGGTTGTTTGTTTTGGATT 3736

NC_019843.3 ------------------------------------------------------------ 3554

NC_004718.3 ------------------------------------------------------------ 3298

EPI_ISL_6640916_omicron ------------------------------------------------------------ 3311

MW494315.1 ------------------------------------------------------------ 3365

KF686346.1 ACACATACTAACTGTTGGTTGCGTTCTGTGTTACTTGTAATGCAGAAATTACCTTTTAAG 3813

NC_006213.1 ACTCGCAACAATTGCTGGTTGCGATCAGTTTTGGCAGTGATGCAGAAATTGCCCTGTCAA 3476

NC_002645.1 ACGCCCACTAAGAAGGCGTTTCCTTATGGTACTTGTCTAAATTGTAACGCACCTCGCATG 3778

JX504050.1 ATGCCTTACACAAAACTTTTTCAAAAAGGTGAGTGTTGTATTTGTCATAAAATGCAGACT 3796

NC_019843.3 ------------------------------------------------------------ 3554

NC_004718.3 ------------------------------------------------------------ 3298

EPI_ISL_6640916_omicron ------------------------------------------------------------ 3311

MW494315.1 ------------------------------------------------------------ 3365

KF686346.1 TTTAAGGATTTAGCTATTGAAAATATGTGGTTATCTTA---TAAGGTGGGTTATAATCAA 3870

NC_006213.1 TTTAAAGATAAAAATTTGCAGGATCTTTGGGTGTTATA---CAAGCAACAGTATAGTCAG 3533

NC_002645.1 TGTACAATTAGGCAGTTACAAGGTACCATAATATTTGTGCAACAAAAACCAGAACCTGTT 3838

JX504050.1 TATAAGCTTGTTAGTATGAAAGGTACTGGTGTGTTTGT---ACAGGATCCAGCACCTATT 3853

NC_019843.3 ---------------------------------------------------------CAG 3557

NC_004718.3 ------------------------------------------------------------ 3298

EPI_ISL_6640916_omicron ------------------------------------------------------------ 3311

MW494315.1 ------------------------------------------------------------ 3365

KF686346.1 AGTTTTGTTGATTATTTACTGACCACTATTCCTAAAGCTATTGTTTTGCCTCAAGGTGGT 3930

NC_006213.1 TTGTTTGTTGATACCTTGGTTAATAAGATACCTGCTAATATTGTACTTCCACAAGGTGGT 3593

NC_002645.1 AATCCTGTTTCTTTTGTTGTTAAACCAGTCTGCTCATCAATTTTTCGTGGTGCTGTGTCT 3898

JX504050.1 GACATTGATGCTTTCCCTGTGAAACCTATATGTTCATCTGTATATTTAGGTGTTAAGGGT 3913

NC_019843.3 AATGTTACTGTTAAACCTAAGAGGTTACGCAAAAAGCGTAATGTTGACCCTTTGT----- 3612

NC_004718.3 ------------------------------------------------------------ 3298

EPI_ISL_6640916_omicron ------------------------------------------------------------ 3311

MW494315.1 ------------------------------------------------------------ 3365

KF686346.1 TTTGTAGCTGATTTTGCTTATTGGTTTTTAAACCAGTTTGATATTAATGCGTATGCTAAT 3990

NC_006213.1 TATGTTGCTGATTTTGCATATTGGTTTTTAACCTTATGTGATTGGCAGTGTGTTGCATAC 3653

NC_002645.1 TGTGGTCATTACCAGACTAACATCTATTCACAAAATTTGTGTGTGGATGGTTTTG----- 3953

JX504050.1 TCTGGTCATTATCAAACAAATTTATACAGTTTTAACAAAGCTATTGATGGTTTTG----- 3968

NC_019843.3 ------------------------------------------------------------ 3612

NC_004718.3 ------------------------------------------------------------ 3298

EPI_ISL_6640916_omicron ------------------------------------------------------------ 3311

MW494315.1 ------------------------------------------------------------ 3365

KF686346.1 TGGTGTTGTTTAAAATGTGGTTTTTCTTTTGATTTAAATGGTTTGGATGCTTTGTTTTTT 4050

NC_006213.1 TGGAAATGCATTAAATGTGATTTAGCTCTTAAGCTTAAAGGCTTGGATGCTATGTTCTTT 3713

NC_002645.1 ------------------------------------------------------------ 3953

JX504050.1 ------------------------------------------------------------ 3968

NC_019843.3 ------------------------------------------------------------ 3612

NC_004718.3 ------------------------------------------------------------ 3298

EPI_ISL_6640916_omicron ------------------------------------------------------------ 3311

MW494315.1 ------------------------------------------------------------ 3365

KF686346.1 TATGGAGATATTGTGTCTCATGTTTGTAAGTGTGGACATAATATGACTCTAATAGCAGCG 4110

NC_006213.1 TATGGTGATGTTGTTTCACATATATGCAAGTGTGGTGAGTCTATGGTACTTATTGATGTT 3773

NC_002645.1 ------------------------------------------------------------ 3953

JX504050.1 ------------------------------------------------------------ 3968

NC_019843.3 ------------------------------------------------------------ 3612

NC_004718.3 ------------------------------------------------------------ 3298

EPI_ISL_6640916_omicron ------------------------------------------------------------ 3311

MW494315.1 ------------------------------------------------------------ 3365

KF686346.1 GACTTACCTTGTACATTACATTTTTCATTATTTGATGACAATTTTTGTGCTTTTTGCACC 4170

NC_006213.1 GATGTGCCATTTACAGCCCACTTTGCTCTTAAAGATAAGTTGTTTTGTGCATTTATTACT 3833

NC_002645.1 ----------------------------------------------GTGTTAACAAGATT 3967

JX504050.1 ----------------------------------------------GTGTCTTTGACATT 3982

NC_019843.3 ------------------------------------------------------------ 3612

NC_004718.3 ------------------------------------------------------------ 3298

EPI_ISL_6640916_omicron ------------------------------------------------------------ 3311

MW494315.1 ------------------------------------------------------------ 3365

KF686346.1 ---------CCTAAAAAAATTTTTATTGCTGCATGTGCTGTGGATGTAAACGTTTGTCAT 4221

NC_006213.1 ---------AAGCGTATTGTGTATAAAGCAGCTTGTGTTGTGGATGTTAATGATAGTCAT 3884

NC_002645.1 CAGCCCTGGACAAATGATGCACTTAATACTATTTGTATTAAGGATGCA---GATTATAAT 4024

JX504050.1 ---------AAAAATAGTAGTGTTAATACTGTTTGTTTTGTTGATGTT---GATTTTCAT 4030

NC_019843.3 ------------------------------------------------------------ 3612

NC_004718.3 ------------------------------------------------------------ 3298

EPI_ISL_6640916_omicron ------------------------------------------------------------ 3311

MW494315.1 ------------------------------------------------------------ 3365

KF686346.1 TCTGTAGCTGTTATAGGTGATGAACAAATAGATGGTAAGTTTGTTACTAAATTTAGTGGT 4281

NC_006213.1 TCTATGGCTGTTGTTGATGGTAAACAAATTGATGATCATCGTATCACTAGTATTACTAGT 3944

NC_002645.1 GCAAAAGTTGAAATATCTGTTACACCAATTAAAAATACAGTTGATACAACACCTAAGGAA 4084

JX504050.1 AGTGTAGAAATAGAAGCTG----------------------------------------- 4049

NC_019843.3 ------------------------------------------------------------ 3612

NC_004718.3 ------------------------------------------------------------ 3298

EPI_ISL_6640916_omicron ------------------------------------------------------------ 3311

MW494315.1 ------------------------------------------------------------ 3365

KF686346.1 GATAAATTTGATTTTATAGTAGGTTATGGAATGTCATTTAGTATGTCTTCTTTTGAGTTA 4341

NC_006213.1 GATAAGTTTGATTTTATTATTGGGCATGGTATGTCATTTTCAATGACTACTTTTGAAATT 4004

NC_002645.1 GA---------------------------------------------ATTTGTTGTTAAA 4099

JX504050.1 ------------------------------------------------------------ 4049

NC_019843.3 CCAATTTTGAACATAAGGTT-ATTACAGAGTGCGTTACCATAGTTTTAGGTGACGCAATT 3671

NC_004718.3 ----------------------TTACTGACAATGTTGCCATTAAATGTGTTGACATCGTT 3336

EPI_ISL_6640916_omicron ----------------------TTACTGACAATGTATACATTAAAAATGCAGACATTGTG 3349

MW494315.1 ----------------------TTACTGACAATGTATACATTAAAAATGCAGACATTGTG 3403

KF686346.1 GCTCAATTGTATGGTTTGTGTATAACACCTAATGTATGTTTTGTTAAAGGTGATATTATA 4401

NC_006213.1 GCCCAATTGTATGGTTCTTGTATAACACCTAATGTGTGTTTTGTTAAAGGTGATATAATT 4064

NC_002645.1 GAGAAGTTGAACGCCTTCCTCGTTCATGACAATGTAGCTTTCTACCAAGGTGATGTTGAT 4159

JX504050.1 GTGAAGTTAAACCTTTTGCT-GTATATAAAAATGTTAAATTTTATTTAGGTGATATTTCA 4108

* ** * * **

NC_019843.3 CAAGTAGCCAAGTGCTATGGGGAGTCTGTGTTAGTTAATGCTGCTAACACACATCTTAAG 3731

NC_004718.3 AAGGAGGCACAAAGTGCTAATCCTATGGTGATTGTAAATGCTGCTAACATACACCTGAAA 3396

EPI_ISL_6640916_omicron GAAGAAGCTAAAAAGGTAAAACCAACAGTGGTTGTTAATGCAGCCAATGTTTACCTTAAA 3409

MW494315.1 GAAGAAGCTAAAAAGGTAAAACCAACAGTGGTTGTTAATGCAGCCAATGTTTACCTTAAA 3463

KF686346.1 AATGTTGCTAGACTTGTTAAAGCTGATGTTATTGTTAATCCTGCTAATGGGCATATGCTC 4461

NC_006213.1 AAAGTATCTAAGCTTGTTAAAGCAGAAGTTGTTGTAAACCCTGCTAATGGCCATATGGCA 4124

NC_002645.1 ACTGTTGTTAATGGTGTTGACTTTGACTTTATTGTAAATGCTGCTAATGAGAACCTTGCT 4219

JX504050.1 CACCTTGTAAACTGTGTTTCTTTTGACTTTGTTGTCAATGCTGCTAATGAAAATCTCTTG 4168

* * ** ** * ** ** * *

NC_019843.3 CATGGCGGTGGTATCGCTGGTGCTATTAATGCGGCTTCAAAAGGGGCTGTCCAAAAAGAG 3791

NC_004718.3 CATGGTGGTGGTGTAGCAGGTGCACTCAACAAGGCAACCAATGGTGCCATGCAAAAGGAG 3456

EPI_ISL_6640916_omicron CATGGAGGAGGTGTTGCAGGAGCCTTAAATAAGGCTACTAACAATGCCATGCAAGTTGAA 3469

MW494315.1 CATGGAGGAGGTGTTGCAGGAGCCTTAAATAAGGCTACTAACAATGCCATGCAAGTTGAA 3523

KF686346.1 CATGGTGGTGGAGTTGCAAAAGCTATAGCTGTAGCTGCAGGTAAAAAATTTTCTAAAGAA 4521

NC_006213.1 CATGGTGGTGGTGTTGCAAAAGCTATTGCAGTAGCAGCTGGACAGCAGTTTGTTAAAGAG 4184

NC_002645.1 CATGGTGGAGGACTTGCCAAAGCTTTAGATGTGTACACTAAAGGTAAACTTCAACGTTTA 4279

JX504050.1 CATGGAGGCGGTGTTGCACGTGCTATTGATATTTTGACTGAAGGTCAACTTCAGTCATTA 4228

***** ** ** * ** ** * * *

NC_019843.3 TCAGATGAGTATATTCTGGCTAAAGGGCCGTTACAAGTAGGAGATTCAGTTCTCTTGCAA 3851

NC_004718.3 AGTGATGATTACATTAAGCTAAATGGCCCTCTTACAGTAGGAGGGTCTTGTTTGCTTTCT 3516

EPI_ISL_6640916_omicron TCTGATGATTACATAGCTACTAATGGACCACTTAAAGTGGGTGGTAGTTGTGTTTTAAGC 3529

MW494315.1 TCTGATGATTACATAGCTACTAATGGACCACTTAAAGTGGGTGGTAGTTGTGTTTTAAGC 3583

KF686346.1 ACTGCTGCTATGGTTAAATCTAAAGGTGTTTGCCAAGTAGGAGATTGTTATGTTTCTACC 4581

NC_006213.1 ACTACCGATATGGTTAAGTCTAAAGGAGTTTGTGCTACTGGAGATTGTTATGTCTCTACA 4244

NC_002645.1 TCTAAAGAACACATTGGATTAGCGGGTAAAGTAAAAGTTGGTACAGGAGTTATGGTTGAG 4339

JX504050.1 TCTAAAGATTACATTAGTAGTAATGGTCCACTTAAGGTTGGAGCAGGTGTTATGTTGGAG 4288

* * ** ** * *

NC_019843.3 GGCCATTCTCTAGCTAAGAATATCCTGCATGTCGTAGGCCCAGATGCCCGCGCTAAAC-- 3909

NC_004718.3 GGACATAATCTTGCTAAGAAGTGTCTGCATGTTGTTGGACCTAACCTAAATGCAGGTG-- 3574

EPI_ISL_6640916_omicron GGACACAATCTTGCTAAACACTGTCTTCATGTTGTCGGCCCAAATGTTAACAAAGGTG-- 3587

MW494315.1 GGACACAATCTTGCTAAACACTGTCTTCATGTTGTCGGCCCAAATGTTAACAAAGGTG-- 3641

KF686346.1 GGTGGTAAATTATGTAAAACAATTCTTAATATTGTAGGCCCTGATGCTAGACAAGATGGA 4641

NC_006213.1 GGGGGCAAATTATGTAAAACTGTGCTTAATGTTGTTGGACCTGATGCGAGAACACAGGGT 4304

NC_002645.1 TGTGATAGCCT------TAGAATTTTTAATGTTGTTGGTCCACGCAAGGGTAAACATG-- 4391

JX504050.1 TGTGAAAAATT------CAACGTATTTAATGTTGTTGGTCCGCGAACTGGTAAACATG-- 4340

* * * ** * ** ** **

NC_019843.3 ----AGGATGTTTCTCTCCTTAGTAAGTGCTATAAGGCTATGAATGCATATCCTCTTGTA 3965

NC_004718.3 ----AGGACATCCAGCTTCTTAAGGCAGCATATGAAAATTTCAATTCACAGGACATCTTA 3630

EPI_ISL_6640916_omicron ----AAGACATTCAACTTCTTAAGAGTGCTTATGAAAATTTTAATCAGCACGAAGTTCTA 3643

MW494315.1 ----AAGACATTCAACTTCTTAAGAGTGCTTATGAAAATTTTAATCAGCACGAAGTTCTA 3697

KF686346.1 AGACAATCTTATGTTTTGTTAGCACGTGCTTATAAGCATCTTAATAATTATGATTGTTGT 4701

NC_006213.1 AAACAAAGTTATGTATTGTTAGAGCGTGTTTATAAACATCTTAACAACTATGACTGTGTT 4364

NC_002645.1 ----AACGTGATTTACTCATAAAAGCTTACAACACTATTAATAATGAACAAGGCACACCT 4447

JX504050.1 ----AGCATTCATTACTTGTTGAAGCTTATAATTCTATTTTATTTGAAAATGGTATTCCA 4396

* * * * * *

NC_019843.3 GTCACTCCTCTTGTTTCAGCAGGCATATTTGGTGTAAAACCAGCTGTGTCTTTTGATTAT 4025

NC_004718.3 CTTGCACCATTGTTGTCAGCAGGCATATTTGGTGCTAAACCACTTCAGTCTTTACAAGTG 3690

EPI_ISL_6640916_omicron CTTGCACCATTATTATCAGCTGGTATTTTTGGTGCTGACCCTATACATTCTTTAAGAGTT 3703

MW494315.1 CTTGCACCATTATTATCAGCTGGTATTTTTGGTGCTGACCCTATACATTCTTTAAGAGTT 3757

KF686346.1 TTGTCTACTCTCATATCGGCTGGTATATTTAGTGTTCCTGCTGATGTGTCATTAACTTAC 4761

NC_006213.1 GTTACAACTTTGATCTCAGCTGGTATATTTAGTGTGCCTTCTGATGTGTCTTTAACATAT 4424

NC_002645.1 TTAACACCAATTTTGAGCTGTGGTATTTTTGGTATCAAACTCGAAACTTCATTAGAAGTT 4507

JX504050.1 CTTATGCCTCTTCTTAGTTGTGGTATTTTTGGTGTAAGGATTGAAAATTCTCTTAAAGCT 4456

* * * * ** ** *** ** ** *

NC_019843.3 CTTATTAGGGAGG---CTAAGACTAGAGTTTTAGTCGTCGTTAATTCCCAAGATGTCTAT 4082

NC_004718.3 TGCGTGCAGACGG---TTCGTACACAGGTTTATATTGCAGTCAATGACAAAGCTCTTTAT 3747

EPI_ISL_6640916_omicron TGTGTAGATACTG---TTCGCACAAATGTCTACTTAGCTGTCTTTGATAAAAATCTCTAT 3760

MW494315.1 TGTGTAGATACTG---TTCGCACAAATGTCTACTTAGCTGTCTTTGATAAAAATCTCTAT 3814

KF686346.1 CTTCTAGGTGTTG---TTGATAAACAAGTTATCCTTGTTAGTAATAATAAAGAAGATTTT 4818

NC_006213.1 CTACTTGGTACTG---CTAAGAAACAAGTTGTTCTTGTTAGCAATAATCAAGAGGATTTT 4481

NC_002645.1 TTGCTTGATGTTTGTAATACAAAAGAAGTTAAAGTTTTTGTTTATACAGACACAGAGGTT 4567

JX504050.1 TTGTTTAGTTGTGACATTAATAAACCATTGCAAGTTTTTGTTTATTCTTCAAATGAAGAA 4516

* * * * * *

NC_019843.3 AAGAGTCTTACCATAGTTGACATTCCAC-------------------------------- 4110

NC_004718.3 GAGCAGGTTGTCATGGATTATCTTGATA-------------------------------- 3775

EPI_ISL_6640916_omicron GACAAACTTGTTTCAAGCTTTTTGGAAA-------------------------------- 3788

MW494315.1 GACAAACTTGTTTCAAGCTTTTTGGAAA-------------------------------- 3842

KF686346.1 GATATTATTCAAAAATGTCAAATTACTT---------------CAGTTGTTGGTACTAAA 4863

NC_006213.1 GATCTTATTTCTAAGTGTCAGATAACTG---------------CTGTTGAGGGCACTAAG 4526

NC_002645.1 TGTAAGGTTAAGGATTTTGTGTCTGGTTTAGTGAATGTTCAAAAAGTTGAGCAACCTAAA 4627

JX504050.1 CAAGCTGTTCTTAAGTTTTTAGATGGTT-------------------------------- 4544

**

NC_019843.3 -AGAGTTTGACTTTTTCATATGATGGGTTACGTGGCGCAATACGTAAAGCTAAAGAT--- 4166

NC_004718.3 -ACCTGAAGCCTAGAGTGGAAGCACCTAAACAAGAGGAGCCACCAAACACAGAAGAT--- 3831

EPI_ISL_6640916_omicron -TGAAGAGTGAAAAGCAAGTTGAACAAAAGATCGCTGAGATTCCTAAAGAGGAAGTTAAG 3847

MW494315.1 -TGAAGAGTGAAAAGCAAGTTGAACAAAAGATCGCTGAGATTCCTAAAGAGGAAGTTAAG 3901

KF686346.1 GCATTGGCTGTTAGATTAACTGCTAATGTAGGCCGTGTTATTAAATTTGAGACAGAT--- 4920

NC_006213.1 AAATTGGCAGCGCGTCTTTCTTTTAATGTTGGACGTTCCATTGTTTACGAAACAGAT--- 4583

NC_002645.1 ATAGAACCAAAACCAGTGTCCGTAATTAAAGTTGCACCCAAGCCTTACAGGGTAGAT--- 4684

JX504050.1 -TAGATTTAACACCAGTCATTGATGATGTTGATGTTGTTAAACCTTTTAGAGTTGAA--- 4600

*

NC_019843.3 ------------------------------------------------------------ 4166

NC_004718.3 ---TCCAAAACTGAGGAGAAATCTGTCGTACAGAAGCCTGTCGATGTGAAGCCAAAAATT 3888

EPI_ISL_6640916_omicron CCATTTATAACTGAAAGTAAACCTTCAGTTGAACAGAGAAAACAAGATGATAAGAAAATC 3907

MW494315.1 CCATTTATAACTGAAAGTAAACCTTCAGTTGAACAGAGAAAACAAGATGATAAGAAAATC 3961

KF686346.1 ------------------------------------------------------------ 4920

NC_006213.1 ------------------------------------------------------------ 4583

NC_002645.1 ------------------------------------------------------------ 4684

JX504050.1 ------------------------------------------------------------ 4600

NC_019843.3 ------------------------------------------------------------ 4166

NC_004718.3 AAGGCCTGCATTGATGAGGTTACCACAACACTGGAAGAAACTAAGTTTCTTACCAATAAG 3948

EPI_ISL_6640916_omicron AAAGCTTGTGTTGAAGAAGTTACAACAACTCTGGAAGAAACTAAGTTCCTCACAGAAAAC 3967

MW494315.1 AAAGCTTGTGTTGAAGAAGTTACAACAACTCTGGAAGAAACTAAGTTCCTCACAGAAAAC 4021

KF686346.1 ------------------------------------------------------------ 4920

NC_006213.1 ------------------------------------------------------------ 4583

NC_002645.1 ------------------------------------------------------------ 4684

JX504050.1 ------------------------------------------------------------ 4600

NC_019843.3 ---------------------------------------TATGGTTTTACTGTTTTTGTG 4187

NC_004718.3 TTACTCTTGTTTGCTGATATCAATGGTAAGCTTTACCATGATTCTCAGAACATGCTTAGA 4008

EPI_ISL_6640916_omicron TTGTTACTTTATATTGACATTAATGGCAATCTTCATCCAGATTCTGCCACTCTTGTTAGT 4027

MW494315.1 TTGTTACTTTATATTGACATTAATGGCAATCTTCATCCAGATTCTGCCACTCTTGTTAGT 4081

KF686346.1 ---------------------------------------GCATACAAACTTTTTTTGAGT 4941

NC_006213.1 ---------------------------------------GCTAATAAGTTGATTTTAATC 4604

NC_002645.1 ---------------------------------------GGTAAATTTAGTTACTTTACA 4705

JX504050.1 ---------------------------------------GGTAATTTTTCATTCTTTGAT 4621

*

NC_019843.3 TGCACAGAC---------------------------------------AACTCTGCTAAC 4208

NC_004718.3 GGTGAAGAT---------------------------------------ATGTCTTTCCTT 4029

EPI_ISL_6640916_omicron GACATTGAC---------------------------------------ATCACTTTCTTA 4048

MW494315.1 GACATTGAC---------------------------------------ATCACTTTCTTA 4102

KF686346.1 GGTGATGAT---------------------------TGTTTTGTTTCAAATTCTTCTGTT 4974

NC_006213.1 AATGACGTT---------------------------GCATTTGTTTCGACATTTAATGTT 4637

NC_002645.1 GAAGACTTGTTGTGTGTCGCTGATGACAAACCCATTGTTTTGTTTACTGACTCTATGCTT 4765

JX504050.1 TGTGGTGTCAATGCCTTGGATGGTGATATTTAC---TTATTATTTACTAACTCTATTTTA 4678

*

NC_019843.3 ACTAAAGTTCTTAGGAACAAGGGTGTTGATTATACTAAGAAGTTTCTTACAGTTG----- 4263

NC_004718.3 GAGAAGGATGCACCTTACATGGTAGGTGATGTTATCACTAGTGGTGATATCACTTGTGTT 4089

EPI_ISL_6640916_omicron AAGAAAGATGCTCCATATATAGTGGGTGATGTTGTTCAAGAGGGTGTTTTAACTGCTGTG 4108

MW494315.1 AAGAAAGATGCTCCATATATAGTGGGTGATGTTGTTCAAGAGGGTGTTTTAACTGCTGTG 4162

KF686346.1 ATACAAGAAGTTTTATTGCTTCGTCATGATATACAATTGAATAATGACGTTCGTG----- 5029

NC_006213.1 TTACAGGATGTTTTATCCTTAAGACATGATATAGCACTTGATGATGATGCACGAA----- 4692

NC_002645.1 ACTTTGGATGACCGTGGTTTAGCTCTAGACAATGCACTTAGTGGTGTGCTTAGTG----- 4820

JX504050.1 ATGTTGGATAAACAAGGACAATTATTGGACACAAAACTTAATGGTATTTTGCAAC----- 4733

* ** *

NC_019843.3 ------------------------------------------------------------ 4263

NC_004718.3 GTAATACCCTCCAAAAAGGCTGGTGGCACTACTGAGATGCTCTCAAGAGCTTTGAAGAAA 4149

EPI_ISL_6640916_omicron GTTATACCTACTAAAAAGGCTGGTGGCACTACTGAAATGCTAGCGAAAGCTTTGAGAAAA 4168

MW494315.1 GTTATACCTACTAAAAAGGCTGGTGGCACTACTGAAATGCTAGCGAAAGCTTTGAGAAAA 4222

KF686346.1 ------------------------------------------------------------ 5029

NC_006213.1 ------------------------------------------------------------ 4692

NC_002645.1 ------------------------------------------------------------ 4820

JX504050.1 ------------------------------------------------------------ 4733

NC_019843.3 ------------------------------------------------------------ 4263

NC_004718.3 GTGCCAGTTGATGAGTATATAACCACGTACCCTGGACAAGGATGTGCTGGTTATACACTT 4209

EPI_ISL_6640916_omicron GTGCCAACAGACAATTATATAACCACTTACCCGGGTCAGGGTTTAAATGGTTACACTGTA 4228

MW494315.1 GTGCCAACAGACAATTATATAACCACTTACCCGGGTCAGGGTTTAAATGGTTACACTGTA 4282

KF686346.1 ------------------------------------------------------------ 5029

NC_006213.1 ------------------------------------------------------------ 4692

NC_002645.1 ------------------------------------------------------------ 4820

JX504050.1 ------------------------------------------------------------ 4733

NC_019843.3 ------------------------------------------------------------ 4263

NC_004718.3 GAGGAAGCTAAGACTGCTCTTAAGAAATGCAAATCTGCATTTTATGTACTACCTTCAGAA 4269

EPI_ISL_6640916_omicron GAGGAGGCAAAGACAGTGCTTAAAAAGTGTAAAAGTGCCTTTTACATTCTACCATCTATT 4288

MW494315.1 GAGGAGGCAAAGACAGTGCTTAAAAAGTGTAAAAGTGCCTTTTACATTCTACCATCTATT 4342

KF686346.1 ------------------------------------------------------------ 5029

NC_006213.1 ------------------------------------------------------------ 4692

NC_002645.1 ------------------------------------------------------------ 4820

JX504050.1 ------------------------------------------------------------ 4733

NC_019843.3 ------------------------------------------------------------ 4263

NC_004718.3 GCACCTAATGCTAAGGAAGAGATTCTAGGAACTGTATCCTGGAATTTGAGAGAAATGCTT 4329

EPI_ISL_6640916_omicron ATCTCTAATGAGAAGCAAGAAATTCTTGGAACTGTTTCTTGGAATTTGCGAGAAATGCTT 4348

MW494315.1 ATCTCTAATGAGAAGCAAGAAATTCTTGGAACTGTTTCTTGGAATTTGCGAGAAATGCTT 4402

KF686346.1 ------------------------------------------------------------ 5029

NC_006213.1 ------------------------------------------------------------ 4692

NC_002645.1 ------------------------------------------------------------ 4820

JX504050.1 ------------------------------------------------------------ 4733

NC_019843.3 ------------------------------------------------------------ 4263

NC_004718.3 GCTCATGCTGAAGAGACAAGAAAATTAATGCCTATATGCATGGATGTTAGAGCCATAATG 4389

EPI_ISL_6640916_omicron GCACATGCAGAAGAAACACGCAAATTAATGCCTGTCTGTGTGGAAACTAAAGCCATAGTT 4408

MW494315.1 GCACATGCAGAAGAAACACGCAAATTAATGCCTGTCTGTGTGGAAACTAAAGCCATAGTT 4462

KF686346.1 ------------------------------------------------------------ 5029

NC_006213.1 ------------------------------------------------------------ 4692

NC_002645.1 ------------------------------------------------------------ 4820

JX504050.1 ------------------------------------------------------------ 4733

NC_019843.3 -------------------------------------------------------ACGGT 4268

NC_004718.3 GCAACCATCCAACGTAAGTATAAAGGAATTAAAATTCAAGAGGGCATCGTTGACTATGGT 4449

EPI_ISL_6640916_omicron TCAACTATACAGCGTAAATATAAGGGTATTAAAATACAAGAGGGTGTGGTTGATTATGGT 4468

MW494315.1 TCAACTATACAGCGTAAATATAAGGGTATTAAAATACAAGAGGGTGTGGTTGATTATGGT 4522

KF686346.1 -------------------------------------------------------ATTAT 5034

NC_006213.1 -------------------------------------------------------CCTTC 4697

NC_002645.1 -------------------------------------------------------CTGCT 4825

JX504050.1 -------------------------------------------------------AGGCA 4738

NC_019843.3 GTGCAATATTATTGCTACACGTCTAAGGACACTTTAGATGATATCTTACAACAGGCTAA- 4327

NC_004718.3 GTCCGATTCTTCTTTTATACTAGTAAAGAGCCTGTAGCTTCTATTATTACGAAGCTGAAC 4509

EPI_ISL_6640916_omicron GCTAGATTTTACTTTTACACCAGTAAAACAACTGTAGCGTCACTTATCAACACACTTAAC 4528

MW494315.1 GCTAGATTTTACTTTTACACCAGTAAAACAACTGTAGCGTCACTTATCAACACACTTAAC 4582

KF686346.1 TTGTTGTCTAAGATGACT---AGTCTTCCTAAAGATTGGCGTCTTATCAATAAATTTGA- 5090

NC_006213.1 GTTCAGAGCAATGTTGAT---GTTGTACCTGAGGGTTGGCGTGTTGTCAATAAGTTTTA- 4753

NC_002645.1 ATTAAGGATTGTGTTGACATAAATAAAGCTATACCTTCTGGTAATCTTATTAAGTTTGA- 4884

JX504050.1 GCTCTTGATTATCTTGCTACAGTTAAAACTGTACCAGCTGGTAATTTGGTTAAACTTGT- 4797

* *

NC_019843.3 --TAAGTCTGTTGGTATTATATCTATGCCTTTGGGATATGTGTCTCATGGTTTAGACTTA 4385

NC_004718.3 TCTCTAAATGAGCCGCTTGTCACAATGCCAATTGGTTATGTGACACATGGTTTTAATCTT 4569

EPI_ISL_6640916_omicron GATCTAAATGAAACTCTTGTTACAATGCCACTTGGCTATGTAACACATGGCTTAAATTTG 4588

MW494315.1 GATCTAAATGAAACTCTTGTTACAATGCCACTTGGCTATGTAACACATGGCTTAAATTTG 4642

KF686346.1 --TGTTATTAACGGTGTTA----------------------------------------- 5107

NC_006213.1 --TCAAATTAATGGTGTTA----------------------------------------- 4770

NC_002645.1 --TATAGGTTCTGTTGTTGTCTACATGTGTGTTGTGCCATCCGAAAAGGACAAACATTTA 4942

JX504050.1 --TGTTGAGAGTTGTACCATTTATATGTGTGTTGTACCATCGATAAATGATCTTTCTTTT 4855

*

NC_019843.3 ATGCAA---GCAGGGAGTGTCGTGCGTAGAGTTAACG------TGCCCTACG-----TGT 4431

NC_004718.3 GAAGAG---GCTGCGCGCTGTATGCGTTCTCTTAAAG------CTCCTGCCGTAGTGTCA 4620

EPI_ISL_6640916_omicron GAAGAA---GCTGCTCGGTATATGAGATCTCTCAAAG------TGCCAGCTACAGTTTCT 4639

MW494315.1 GAAGAA---GCTGCTCGGTATATGAGATCTCTCAAAG------TGCCAGCTACAGTTTCT 4693

KF686346.1 ---------------------------AAACTGTTAAGTATTTTGAGTGTCCTAATTCTA 5140

NC_006213.1 ---------------------------GAACCGTTAAGTATTTTGAGTGTACTGGAGGCA 4803

NC_002645.1 GATAATAATGTTCAACGATGCACACGTAAGTTGAATAGACTTATGTGTGATATAGTTTGT 5002

JX504050.1 GATAAAAATCTTGGTCGTTGTGTGCGTAAACTTAATAGATTGAAAACTTGTGTTATTGCC 4915

NC_019843.3 GTCTCCTAGCTAATAAAGAGCAAGAAGCTATTTTGATGTCTGAAG--ACGTTAAGTTAAA 4489

NC_004718.3 GTATCATCACCAGATGCTGTTACTACATATAATGGATACCTCACT--TCGTCATCAAAGA 4678

EPI_ISL_6640916_omicron GTTTCTTCACCTGATGCTGTTACAGCGTATAATGGTTATCTTACT--TCTTCTTCTAAAA 4697

MW494315.1 GTTTCTTCACCTGATGCTGTTACAGCGTATAATGGTTATCTTACT--TCTTCTTCTAAAA 4751

KF686346.1 TTTATATATGTAGTCAGGGTAAAGACTTTGGTTATGTATGTGATGGTTCTTTTTATAAAG 5200

NC_006213.1 TAGATATATGCAGCCAGGATAAAGTTTTTGGTTATGTACAGCAGGGTATTTTTAATAAGG 4863

NC_002645.1 ACTATACCAGCTGACTACATCTTGCCATTGGTGTTGTCTAGTTTG--ACTTGTAATGTTT 5060

JX504050.1 AATGTTCCTGCTATTGATGTTTTGAAAAAGCTTCTTTCAAGTTTG--ACTTTAACTGTTA 4973

* *

NC_019843.3 C-CCTTCAGAAGATTTTATAAAGCACGTCCGCACTAATGGTGGTTACAATTCTTGG---C 4545

NC_004718.3 CATCTGAGGAGCACTTTGTAGAAACAGTTTCTTTGGCTGGCTCTTACAGAGATTGGTCCT 4738

EPI_ISL_6640916_omicron CACCTGAAGAACATTTTATTGAAACCATCTCACTTGCTGGTTCCTATAAAGATTGGTCCT 4757

MW494315.1 CACCTGAAGAACATTTTATTGAAACCATCTCACTTGCTGGTTCCTATAAAGATTGGTCCT 4811

KF686346.1 CAACTGTTAATCAAGTTTGTGTTTTATTAGCTAAGA------------------AG---- 5238

NC_006213.1 CTACTGTTGCTCAAATTAAAGCCTTGTTTTTGGATA------------------AA---- 4901

NC_002645.1 CTTTTGTAGGTGAACTTAAAGCTGCTGAAGCTAAAG------------------TT---- 5098

JX504050.1 AATTTGTTGTAGAGAGTAATGTTATGGATGTTAACGACTGTTTTAAGAATGATAAT---- 5029

* * *

NC_019843.3 ATTTAGTCGAGGGTGAACTATTGGTGCAAGACTTACGCTTAAATAAGCTCCTGCATTGGT 4605

NC_004718.3 ATTCAGGACAGCGTACAGAGTTAGGTGTTGAAT---------------TTCTTAAGCGTG 4783

EPI_ISL_6640916_omicron ATTCTGGACAATCTACACAACTAGGTATAGAAT---------------TTCTTAAGAGAG 4802

MW494315.1 ATTCTGGACAATCTACACAACTAGGTATAGAAT---------------TTCTTAAGAGAG 4856

KF686346.1 ------------------------------------------------------------ 5238

NC_006213.1 ------------------------------------------------------------ 4901

NC_002645.1 ------------------------------------------------------------ 5098

JX504050.1 ------------------------------------------------------------ 5029

NC_019843.3 CTGATCAAACCATATGCTACAAGG---ATAGTGTGTTTTATGTTGTAAAGAATAGTACAG 4662

NC_004718.3 GTGACAAAATTGTGTACCACACTCTGGAGAGCCCCGTCGAGTTTCATCTTGACGGTGAGG 4843

EPI_ISL_6640916_omicron GTGATAAAAGTGTATATTACACTA---GTAATCCTACCACATTCCACCTAGATGGTGAAG 4859

MW494315.1 GTGATAAAAGTGTATATTACACTA---GTAATCCTACCACATTCCACCTAGATGGTGAAG 4913

KF686346.1 ------------------------------------------------------------ 5238

NC_006213.1 ------------------------------------------------------------ 4901

NC_002645.1 ------------------------------------------------------------ 5098

JX504050.1 ------------------------------------------------------------ 5029

NC_019843.3 CTTTTCCATTTGAAACACTTTCAGCATGTCGTGCGTATTTGGATTCACGCACGACACAGC 4722

NC_004718.3 TTCTTTCACTTGACAAACTAAAGAGTCTCTTATCCCTGCGGGAGGTTAAGACTATAAAAG 4903

EPI_ISL_6640916_omicron TTATCACCTTTGACAATCTTAAGACACTTCTTTCTTTGAGAGAAGTGAGGACTATTAAGG 4919

MW494315.1 TTATCACCTTTGACAATCTTAAGACACTTCTTTCTTTGAGAGAAGTGAGGACTATTAAGG 4973

KF686346.1 ------------------------------------------------------------ 5238

NC_006213.1 ------------------------------------------------------------ 4901

NC_002645.1 ------------------------------------------------------------ 5098

JX504050.1 ------------------------------------------------------------ 5029

NC_019843.3 AGTTAACAATCGAAGTCTTAGTGACTGTCGATGGTGTAAATTTTAGAACAGTCGTTCTAA 4782

NC_004718.3 TGTTCACA---------------ACTGTGGACAACACTAATCTCCACACACAGCTTGTGG 4948

EPI_ISL_6640916_omicron TGTTTACA---------------ACAGTAGACAACATTAACCTCCACACGCAAGTTGTGG 4964

MW494315.1 TGTTTACA---------------ACAGTAGACAACATTAACCTCCACACGCAAGTTGTGG 5018

KF686346.1 --------ATAGATGTTTTGCTTACTGTAGATGGTGTTAATTTTAAATCTATTTCTCTTA 5290

NC_006213.1 --------GTGGACATCTTGCTAACTGTTGATGGTGTTAATTTCACTAATAGGTTTGTGC 4953

NC_002645.1 --------ATAACTATAAAGGTGACAGAGGATGGTGTTAATGTTCATGATGTGACCGTGA 5150

JX504050.1 --------GTAGTTTTGAAAATTACTGAAGATGGTATTAATGTTAAAGATGTTGTTGTTG 5081

** * ** ** * *

NC_019843.3 ATAATAAGAACACTTATAGATCACAGCTTGGATGCGTTTTCTTTAATGGTGCTGATATTT 4842

NC_004718.3 ATATGTCTATGACATATGGACAGCAGTTTGGTCCAACATACTTGGATGGTGCTGATGTTA 5008

EPI_ISL_6640916_omicron ACATGTCAATGACATATGGACAACAGTTTGGTCCAACTTATTTGGATGGAGCTGATGTTA 5024

MW494315.1 ACATGTCAATGACATATGGACAACAGTTTGGTCCAACTTATTTGGATGGAGCTGATGTTA 5078

KF686346.1 CTGTAGGTGAAGTTTTTGGTAAAATACTTGGTAATGTTTTCTGTGATGGCATTGATGTTA 5350

NC_006213.1 CTGTTGGTGAAAGTTTTGGTAAGAGTCTAGGAAATGTGTTTTGTGATGGAGTTAATGTCA 5013

NC_002645.1 CAACAGACAAGTCATTTGAACAACAAGTTGGTGTTATTGCTGATAAGGACAAAGATCTTT 5210

JX504050.1 AGTCTTCTAAGTCACTTGGTAAACAATTGGGTGTTGTGAGTGATGGTGTTGACTCTTTTG 5141

* * ** * * *

NC_019843.3 CTGACACCATTCCTGATGAGAAACAGAATGGTCACAGTTTATATCTAGCAGACAATTTGA 4902

NC_004718.3 CAAAAATTAAACCTCATGTAAATCATGAGGGTAAGACTTTCTTTGTACTACCTAGTGATG 5068

EPI_ISL_6640916_omicron CTAAAATAAAACCTCATAATTCACATGAAGGTAAAACATTTTATGTTTTACCTAATGATG 5084

MW494315.1 CTAAAATAAAACCTCATAATTCACATGAAGGTAAAACATTTTATGTTTTACCTAATGATG 5138

KF686346.1 CTAAGTTAAAGTGTAGTGATTTTTATGCCGATAAAATTTTATATCAGTATGAAAATTTGT 5410

NC_006213.1 CGAAGCATAAGTGTGATATAAATTATAAAGGTAAAGTCTTTTTCCAGTTTGATAATCTTT 5073

NC_002645.1 CTGGTGCAGTACCAAGTGATCTTAACACATCTGAATTGCTTACTAAAGCAATAGATGTTG 5270

JX504050.1 AAGGTGTTTTACCTATTAATACTGATACTGTCTTAT------CTGTAGCTCCAGAAGTTG 5195

* *

NC_019843.3 CTGCTGATGAAACAAAGGCGCTTAAAGAGTTATATGGCCCCGTTGATCCTACTTTCTTAC 4962

NC_004718.3 ACACACTACGTAGTGAAGCTTTC---GAGTACTACCATACTCTTGATGAGAGTTTTCTTG 5125

EPI_ISL_6640916_omicron ACACTCTACGTGTTGAGGCTTTT---GAGTACTACCACACAACTGATCCTAGTTTTCTGG 5141

MW494315.1 ACACTCTACGTGTTGAGGCTTTT---GAGTACTACCACACAACTGATCCTAGTTTTCTGG 5195

KF686346.1 CTTTAGCTGATATTTCTGCTGTA---CAAAGTTCATTTGGGTTTGATCAGCAACAATTGC 5467

NC_006213.1 CTAGTGAAGATTTAAAGGCTGTA---AGAAGTTCCTTTAATTTTGATCAGAAGGAATTGC 5130

NC_002645.1 ATTGGGTCGAATTTTATGGCTTT---AAAGATGCTGTTACTTTTGCAACAGTTGATCATA 5327

JX504050.1 ACTGGGTTGCTTTTTACGGTTTT---GAAAAGGCAGCACTTTTTGCTTCTTTGGATGTAA 5252

* * **

NC_019843.3 ACAGATTCTATTCACTTAAGGCTGCAGTCCATGGGTGGAAGATGGTTGTGTGTGATAAGG 5022

NC_004718.3 GTAGGTACATGTCTGCTTTAAACCACACAAAGAAATGGAAATTTCCTCAAGTTGGTGGTT 5185

EPI_ISL_6640916_omicron GTAGGTACATGTCAGCATTAAATCACACTAAAAAGTGGAAATACCCACAAGTTAATGGTT 5201

MW494315.1 GTAGGTACATGTCAGCATTAAATCACACTAAAAAGTGGAAATACCCACAAGTTAATGGTT 5255

KF686346.1 TTGCTTATTATAAT---TTTTTAACAGTATGTAAATGGTCTGTAGTTGTTAACGGTCCAT 5524

NC_006213.1 TTGCCTATTACAACATGCTTGTTAATTGTTTTAAGTGGCAGGTTGTTGTTAATGGTAAGT 5190

NC_002645.1 GTGCTTTTGCCTAT------------------------GAAAGTGCTGTTGTTAATGGTA 5363

JX504050.1 AGCCATATGGTTAC------------------------CCTAATGATTTTGTTGGTGGTT 5288

* *

NC_019843.3 TACGTTCTCTCAAATTGAGTGATAATAATTGTTATCTTAATGCAGTTATTATGACACTTG 5082

NC_004718.3 TAACTTCAATTAAATGGGCTGATAACAATTGTTATTTGTCTAGTGTTTTATTAGCACTTC 5245

EPI_ISL_6640916_omicron TAACTTCTATTAAATGGGCAGATAACAACTGTTATCTTGCCACTGCATTNTTAACACTCC 5261

MW494315.1 TAACTTCTATTAAATGGGCAGATAACAACTGTTATCTTGCCACTGCATTGTTAACACTCC 5315

KF686346.1 TTTTTTCTTTTGAACAGTCTCATAATAATTGTTATGTGAATGTAGCTTGTCTTATGTTGC 5584

NC_006213.1 ATTTCACTTTTAAGCAAGCTAATAACAATTGTTTTGTTAATGTTTCTTGCTTAATGCTCC 5250

NC_002645.1 TTAGAGTGTTAAAAACTAGTGATAATAATTGTTGGGTGAATGCTGTTTGTATTGCACTAC 5423

JX504050.1 TTAGAGTTCTTGGGACCACCGACAATAATTGTTGGGTTAATGCAACTTGTATAATTTTAC 5348

* * ** ** **** * * *

NC_019843.3 ATTTATTGAAGGACATTAAATTTGTTATACCTGCTCTACAGCATGCATTTATGAAACATA 5142

NC_004718.3 A---ACAGCTTGAAGTCAAATTCAATGCACCAGCACTTCAAGAGGCTTATTATAGAGCCC 5302

EPI_ISL_6640916_omicron A---ACAAATAGAGTTGAAGTTTAATCCACCTGCTCTACAAGATGCTTATTACAGAGCAA 5318

MW494315.1 A---ACAAATAGAGTTGAAGTTTAATCCACCTGCTCTACAAGATGCTTATTACAGAGCAA 5372

KF686346.1 A---GCATATTAATCTTAAATTTAATAAATGGCAGTGGCAGGAAGCATGGTATGAATTTC 5641

NC_006213.1 A---GAGTTTGCATCTGACATTTAAAATTGTTCAATGGCAAGAGGCATGGCTTGAATTTC 5307

NC_002645.1 A---GTATTCGAAACCCCATTTTATTTCACAAGGTCTTGATGCTGCGTGGAATAAATTTG 5480

JX504050.1 A---GTATCTTAAGCCTACTTTTAAATCTAAGGGTTTAAATGTTCTTTGGAACAAATTTG 5405

* * ** * * *

NC_019843.3 AGGGCGGTGATTCAACTGACTTCATAGCCCTCATTATGGCTTATGGCAATTGCACATTTG 5202

NC_004718.3 GTGCTGGTGATGCTGCTAACTTTTGTGCACTCATACTCGCTTACAGTAATAAAACTGTTG 5362

EPI_ISL_6640916_omicron GGGCTGGTGAAGCGGCTAACTTTTGTGCACTTATCTTAGCCTACTGTAATAAGACAGTAG 5378

MW494315.1 GGGCTGGTGAAGCTGCTAACTTTTGTGCACTTATCTTAGCCTACTGTAATAAGACAGTAG 5432

KF686346.1 GTGCTGGCAGACCACATAGATTAGTTGCTCTTGTTTTAGCTAAAGGTCATTTTAAATTTG 5701

NC_006213.1 GTTCTGGCCGCCCTGCTAGATTTGTAGCTTTGGTTTTGGCCAAAGGTGGGTTTAAATTTG 5367

NC_002645.1 TTTTAGGCGATGTTGAAATTTTTGTTGCATTTGTTTACTATGTTGCAAGACTAATGAAAG 5540

JX504050.1 TTACAGGTGATGTTGGACCTTTTGTTAGTTTTATTTATTTTATAACTATGTCTTCAAAGG 5465

** ** * * *

NC_019843.3 GTGCTCCAGATGATGCCTCTCGGTTACTTCATACCGTGCTTGCAAAGGCTGAGTTATGCT 5262

NC_004718.3 GCGAGCTTGGTGATGTCAGAGAAACTATGACCCATCTTCTACAGCATGCTAATTTG---G 5419

EPI_ISL_6640916_omicron GTGAGTTAGGTGATGTTAGAGAAACAATGAGTTACTTGTTTCAACATGCCAATTTA---G 5435

MW494315.1 GTGAGTTAGGTGATGTTAGAGAAACAATGAGTTACTTGTTTCAACATGCCAATTTA---G 5489

KF686346.1 ATGAACCATCAGATGCTACTGATTTTATTCGTGTTGTTTTGAAACAAGCTGATTTA---- 5757

NC_006213.1 GAGATCCTGCTGATTCTAGAGATTTCTTGCGTGTTGTGTTTAGTCAAGTTGATTTG---- 5423

NC_002645.1 GTGACAAGGGTGATGCTGAAGACACTTTGACTAAGTTGTCTAAGTATCTTGCT------- 5593

JX504050.1 GTCAAAAGGGTGATGCTGAAGAGGCATTATCTAAATTGTCAGAGTATTTGATT------- 5518

*** * * *

NC_019843.3 GTTCTGCACGCATGGTTTGGAGAGAGTGGTGCAATGTCTGTGGCATAAAAGATGTTGTTC 5322

NC_004718.3 AATCTGCAAAGCGAGTTCTTAATGTGGTGTGTAAACATTGTGGTCAGAAAACTACTACCT 5479

EPI_ISL_6640916_omicron ATTCTTGCAAAAGAGTCTTGAACGTGGTGTGTAAAACTTGTGGACAACAGCAGACAACCC 5495

MW494315.1 ATTCTTGCAAAAGAGTCTTGAACGTGGTGTGTAAAACTTGTGGACAACAGCAGACAACCC 5549

KF686346.1 --TCAGGTGCAATTTGTGAATTAGAACTTATTTGTGATTGTGGTATTAAACAAGAAAGTC 5815

NC_006213.1 --ACTGGGGCAATATGTGATTTTGAAATTGCATGTAAATGTGGTGTAAAGCAGGAACAGC 5481

NC_002645.1 --AATGAAGCTCAAGTTCAATTAGAACATTATAGTTCTTGTGTTGAATGTGATGCTAAAT 5651

JX504050.1 --AGTGATTCTATTGTTACTCTTGAACAATATTCAACTTGTGACATTTG----------- 5565

* ****

NC_019843.3 TACAAGGCTTAAAAGCTTGTTGTTACGTGGGTGTGCAAACTGTTGAAGATCTGCGTGCTC 5382

NC_004718.3 TAACGGGTGTAGAAGCTGTGATGTATATGGGTACTCTATCTTATGATAATCTTAAGACAG 5539

EPI_ISL_6640916_omicron TTAAGGGTGTAGAAGCTGTTATGTACATGGGCACACTTTCTTATGAACAATTTAAGAAAG 5555

MW494315.1 TTAAGGGTGTAGAAGCTGTTATGTACATGGGCACACTTTCTTATGAACAATTTAAGAAAG 5609

KF686346.1 GTGTTGGTGTTGATGCTGTTATGCATTTTGGTACATTAGCAAAGACTGATCTTTTTAATG 5875

NC_006213.1 GTACTGGTCTGGACGCTGTTATGCATTTTGGTACATTGAGTCGTGAAGATCTTGAGATTG 5541

NC_002645.1 TTAAAAACTCTGTTGCATCTATCAATTCTGCTATAGTTTGTGCTAGTGTCAAACGTGATG 5711

JX504050.1 -TAAAAGTACTGTAGTTGAAGTTAAAAGTGCTATTGTCTGTGCTAGTGTGCTTAAAGATG 5624

* * *

NC_019843.3 GCATGACATATGTATGCCAGTGTGGTGGTGAACGTCATCGGCAATTAGTCGAACACACCA 5442

NC_004718.3 GTGTTTCCATTCCATGTGTGTGTGGTCGTGATGCTACACAATATCTAGTACAACAAGAGT 5599

EPI_ISL_6640916_omicron GTGTTCAGATACCTTGTACGTGTGGTAAACAAGCTACAAAATATCTAGTACAACAGGAGT 5615

MW494315.1 GTGTTCAGATACCTTGTACGTGTGGTAAACAAGCTACAAAATATCTAGTACAACAGGAGT 5669

KF686346.1 GTTATAAGATTGGCTGTAATTGTGCAGGTAGAATTGTCCATTGTACTAAATTGAATGTAC 5935

NC_006213.1 GTTATACCGTGGACTGTTCTTGCGGTAAAAAGCTAATTCATTGTGTACGATTTGATGTAC 5601

NC_002645.1 GTGTGCAAGTTG---GTTATTGTGTCCATGGTATTAAGTACTATTCACG------TGTTA 5762

JX504050.1 GTTGTGATGTTG---GTTTTTGTCCACACAGACATAAATTGCGTTCACG------TGTTA 5675

* * **

NC_019843.3 CCCCCTGGTTGCTGCTCTCAGGCACACCAAATGAAAAATTGGTGACAACCTCCACGGCGC 5502

NC_004718.3 CTTCTTTTGTTATGATGTCTGCACCACCTGCTGAGTA-----TAAATTACAGCAAGGTAC 5654

EPI_ISL_6640916_omicron CACCTTTTGTTATGATGTCAGCACCACCTGCTCAGTA-----TGAACTTAAGCATGGTAC 5670

MW494315.1 CACCTTTTGTTATGATGTCAGCACCACCTGCTCAGTA-----TGAACTTAAGCATGGTAC 5724

KF686346.1 CATTTTTGATTTGTTCTAATACTCCTCTGAGTAAGGA------------------TTTAC 5977

NC_006213.1 CATTTTTAATTTGCAGTAATACACCTGCTAGTGTAAA------------------ATTAC 5643

NC_002645.1 GAAGTGTTAGAGGTAGAGCTATTATAGTCAGTGTCGA------------------ACAGC 5804

JX504050.1 AGTTTGTTAATGGACGTGTTGTTATTACCAATGTTGG------------------TGAAC 5717

* *

NC_019843.3 CTGATTTTGTAGCATTTAATGTCTTTCAGGGCATTGAAACGGCTGTTGGCCATTATGTTC 5562

NC_004718.3 ATTCTTATGTGCGAATGAGTACA---CTGGTAACTATCAGTG----TGGTCATTACACTC 5707

EPI_ISL_6640916_omicron ATTTACTTGTGCTAGTGAGTACA---CTGGTAATTACCAGTG----TGGTCACTATAAAC 5723

MW494315.1 ATTTACTTGTGCTAGTGAGTACA---CTGGTAATTACCAGTG----TGGTCACTATAAAC 5777

KF686346.1 CTGATGATGTTGTTGCAGCTAAC---ATGTTTATGGGTGTAGGTGTAGGCCATTATACAC 6034

NC_006213.1 CTAAGGGTGTAGGAAGTGCAAAT---ATTTTTATAGGTGATAAGGTTGGTCATTATGTTC 5700

NC_002645.1 TTGAACCGTGTGCTCAGTCTAGA---CTTTTGAGTGGTG--------------------- 5840

JX504050.1 CTATAATTTCACAACCTTCTAAG---TTGCTTAATGGTA--------------------- 5753

* *

NC_019843.3 ATGCTCGCCTGAAGGGTGGTCTTATTTTAAAGTTTGACTCTGGCACCGTTAGCAAGACTT 5622

NC_004718.3 ATATAACTGCTAAGGAGACCCTCTATCGTA---TTGACGGAGCTCACCTTACAAAGATGT 5764

EPI_ISL_6640916_omicron ATATAACTTCTAAAGAAACTTTGTATTGCA---TAGACGGTGCTTTACTTACAAAGTCCT 5780

MW494315.1 ATATAACTTCTAAAGAAACTTTGTATTGCA---TAGACGGTGCTTTACTTACAAAGTCCT 5834

KF686346.1 ATTTGAAATGTGGTTCACCTTACCAACATT---ATGATGCTTGTAGTGTTAAAAAATACA 6091

NC_006213.1 ATGTTAAGTGTGAACAATCTTATCAGCTTT---ATGATGCTTCTAATGTTAAGAAGGTTA 5757

NC_002645.1 ------------------------------------------------------------ 5840

JX504050.1 ------------------------------------------------------------ 5753

NC_019843.3 CAGACTGGAAGTGCAAGGTGACAGATGTACTTTTCCCCGGCCA---AAAATACAGTAGCG 5679

NC_004718.3 CAGAGTACAAAGGACCAGTGACTGATGTTTTCTACAAGGAAAC---ATCTTACACTACAA 5821

EPI_ISL_6640916_omicron CAGAATACAAAGGTCCTATTACGGATGTTTTCTACAAAGAAAA---CAGTTACACAACAA 5837

MW494315.1 CAGAATACAAAGGTCCTATTACGGATGTTTTCTACAAAGAAAA---CAGTTACACAACAA 5891

KF686346.1 CAGGTGTTAGTGGTTGTTTAACTGACTGCTTGTATCTTAAAAATTTAACCCAGACTTTTA 6151

NC_006213.1 CAGATGTTACTGGCAAGTTGTCAGATTGTCTGTATCTTAAAAATTTGAAACAAACTTTTA 5817

NC_002645.1 ------------------------------------------------------------ 5840

JX504050.1 ------------------------------------------------------------ 5753

NC_019843.3 ATTGTAATGTCGTACGGTATTCTTTGGACGGTAATTTCAGAACAGAGGTTGATCCCGACC 5739

NC_004718.3 CCATCAAGCCTGTGTCGTATAAACTCGATGGAGTTACTTACACAGAGATTGAACCAAAAT 5881

EPI_ISL_6640916_omicron CCATAAAACCAGTTACTTATAAATTGGATGGTATTGTTTGTACAGAAATTGACCCTAAGT 5897

MW494315.1 CCATAAAACCAGTTACTTATAAATTGGATGGTGTTGTTTGTACAGAAATTGACCCTAAGT 5951

KF686346.1 CATCTATGTTGACTAATTATTTTTTGGATGATGTTGAAATGGTTGCTTATAACCCTGATC 6211

NC_006213.1 AATCGGTGTTAACCACCTATTATTTGGATGATGTTAAGAAAATTGAGTATAAACCTGACT 5877

NC_002645.1 ------------------------------------------------------------ 5840

JX504050.1 ------------------------------------------------------------ 5753

NC_019843.3 TATCTGCTTTCTATGTTAAGGATGGTAAATACTTTACAAGTGAACCACCCGTAACATATT 5799

NC_004718.3 TGGATGGGTATTATAAAAAGGATAATGCTTACTATACA---GAGCAGCCTATAGACCTTG 5938

EPI_ISL_6640916_omicron TGGACAATTATTATAAGAAAGACAATTCTTATTTCACA---GAGCAACCAATTGATCTTG 5954

MW494315.1 TGGACAATTATTATAAGAAAGACAATTCTTATTTCACA---GAGCAACCAATTGATCTTG 6008

KF686346.1 TTTCACAATATTATTGTGATAATGGTAAGTATTATACA------AAACCTATTATAAAGG 6265

NC_006213.1 TGTCACAATATTATTGTGACGGAGGTAAGTATTATACT------CAGCGTATTATTAAAG 5931

NC_002645.1 ------------------------------------------------------------ 5840

JX504050.1 ------------------------------------------------------------ 5753

NC_019843.3 CACCAGCTACAATTTTAGC------TGGTAGTGTCTACACTAATAGCTGCCTTGTATCGT 5853

NC_004718.3 TACCAACTCAACCATTACC------AAATGCGAGTTTTGATAATTTCAAACTCACATGTT 5992

EPI_ISL_6640916_omicron TACCAAACCAACCATATCC------AAACGCAAGCTTCGATAATTTTAAGTTTGTATGTG 6008

MW494315.1 TACCAAACCAACCATATCC------AAACGCAAGCTTCGATAATTTTAAGTTTGTATGTG 6062

KF686346.1 CTCAGTTTAAACCATTTGCTAAAGTTGACGGTGTTTATACTAACTTTAAGTTAGTTGGAC 6325

NC_006213.1 CCCAATTTAAAACATTCGAGAAAGTAGATGGTGTGTATACTAATTTTAAATTGATAGGAC 5991

NC_002645.1 ------------------------------------------------------------ 5840

JX504050.1 ------------------------------------------------------------ 5753

NC_019843.3 CTGATGGACAACCTGGCGGTGATGCTATTAGTTTGAGTTTTAATAACCTTTTAGGGTTTG 5913

NC_004718.3 CTAACACAAAATTTGCTGATGATTTAAATCAAATGAC---------------------AG 6031

EPI_ISL_6640916_omicron ATAATATCAAATTTGCTGATGATTTAAACCAGTTAAC---------------------TG 6047

MW494315.1 ATAATATCAAATTTGCTGATGATTTAAACCAGTTAAC---------------------TG 6101

KF686346.1 ATGATATT---TGTGCTCAATTGAATGATAAGTTAGGTTTTAATGTAGATTTGCCGTTTG 6382

NC_006213.1 ACACCGTC---TGTGACAGTCTTAATGCTAAGTTGGGTTTTGATAGCTCTAAAGAGTTTG 6048

NC_002645.1 ------------------------------------------------------------ 5840

JX504050.1 ------------------------------------------------------------ 5753

NC_019843.3 ATTCTAGTAAACCAGTCACTAAGAAATACACTTACTCCTTCTTGCCTAAAGAAGACGGCG 5973

NC_004718.3 GCTTCACAAAGCCAGCTTCACGAGAGCTATCTGTCACATTCTTCCCAGACTTGAATGGCG 6091

EPI_ISL_6640916_omicron GTTATAAGAAACCTGCTTCAAGAGAGCTTAAAGTTACATTTTTCCCTGACTTAAATGGTG 6107

MW494315.1 GTTATAAGAAACCTGCTTCAAGAGAGCTTAAAGTTACATTTTTCCCTGACTTAAATGGTG 6161

KF686346.1 TT---------------------GAGTACAAAGTAACAGTCTGGCCTGTAGCTACTGGTG 6421

NC_006213.1 TT---------------------GAATATAAGATTACTGAGTGGCCAACAGCTACAGGTG 6087

NC_002645.1 ------------------------------------------------------------ 5840

JX504050.1 ------------------------------------------------------------ 5753

NC_019843.3 ATGTGTTGTTGGCTGAGTTTGACACTTATGACCCTATTTATAAGAATGGTGCCATGTATA 6033

NC_004718.3 ATGTAGTGGCTATTGACTATAGACACTATTCAGCGAGTTTCAAGAAAGGTGCTAAATTAC 6151

EPI_ISL_6640916_omicron ATGTGGTGGCTATTGATTATAAACACTACACACCCTCTTTTAAGAAAGGAGCTAAATTGT 6167

MW494315.1 ATGTGGTGGCTATTGATTATAAACACTACACACCCTCTTTTAAGAAAGGAGCTAAATTGT 6221

KF686346.1 ATGTTGTTTTGGCATCTGATGATTTATATGTGAAACGTTATTTTAAAGGATGTGAAACTT 6481

NC_006213.1 ATGTGGTGTTGGCTACTGATGATTTGTATGTTAAGAGATATGAGAGGGGTTGTATTACTT 6147

NC_002645.1 ------------------------------------------------------------ 5840

JX504050.1 ------------------------------------------------------------ 5753

NC_019843.3 AAGGCAAACCAATTCTTTGGGTCAATAAAGCATCTTATGATACTAATCTTAATAAGTTCA 6093

NC_004718.3 TGCATAAGCCAATTGTTTGGCACATTAACCAGGCTACAACCAAGACAACGTTCAAACCAA 6211

EPI_ISL_6640916_omicron TACATAAACCTATTGTTTGGCATGTTAACAATGCAACTAATAAAGCCACGTATAAACCAA 6227

MW494315.1 TACATAAACCTATTGTTTGGCATGTTAACAATGCAACTAATAAAGCCACGTATAAACCAA 6281

KF686346.1 TTGGTAAGCCTGTTATTTGGTTTTGTCATGATGAAGCATCATTGAATTCTCTTACTTA-- 6539

NC_006213.1 TTGGTAAACCTGTTATATGGTTAAGCCATGAGAAAGCTTCCCTCAATTCTTTAACATA-- 6205

NC_002645.1 ------------------------------------------------------------ 5840

JX504050.1 ------------------------------------------------------------ 5753

NC_019843.3 ATAGAGCTAGTTTGCGTCAAATTTTTGACGTAGCCCCCATTGAACTCGAAAATAAATTCA 6153

NC_004718.3 ACACTTGGTGTTTACGTTGTCTTTGGAGTACAAAGCCAGTAGATACTTCAAATTCATTTG 6271

EPI_ISL_6640916_omicron ATACCTGGTGTATACGTTGTCTTTGGAGCACAAAACCAGTTGAAACATCAAATTCGTTTG 6287

MW494315.1 ATACCTGGTGTATACGTTGTCTTTGGAGCACAAAACCAGTTGAAACATCAAATTCGTTTG 6341

KF686346.1 ----------------------TTTTAATAAACCTAGTTTTAAATCTGAAAATAGATATA 6577

NC_006213.1 ----------------------TTTTAATAGACCTTCATTGGTTGATGATAATAAATTTG 6243

NC_002645.1 ------------------------------------------------------------ 5840

JX504050.1 ------------------------------------------------------------ 5753

NC_019843.3 CACCTTTGAGTGTGGAGTCTACACCAG---------------------------TTGAAC 6186

NC_004718.3 AAGTTCTGGCAGTAGAAGACACACAAGGAATGGACAATCTTGCTTGTGAAAGTCAACAAC 6331

EPI_ISL_6640916_omicron ATGTACTGAAGTCAGAGGACGCGCAGGGAATGGATAATCTTGCCTGCGAAGATCTAAAAC 6347

MW494315.1 ATGTACTGAAGTCAGAGGACGCGCAGGGAATGGATAATCTTGCCTGCGAAGATCTAAAAC 6401

KF686346.1 GTGTTTTGTCTGTTGATTCTGTATCTG------------------AGGAGTCACAAGGTA 6619

NC_006213.1 ATGTTTTAAAAGTGGATGATGTTGACG------------------ATGGTGGTGACAGCT 6285

NC_002645.1 ------------------------------------------------------------ 5840

JX504050.1 ------------------------------------------------------------ 5753

NC_019843.3 CTCCAACTGTAGATGTGGTAGCACTTCAACAGGAAATGACAATTGTCAAATGTAAGGGTT 6246

NC_004718.3 CCACCTCTGAAGAAGTAGTGGAAAATCCTACCATACAGAAGGAAGTCATAGAGTGTGACG 6391

EPI_ISL_6640916_omicron CAGTCTCTGAAGAAGTAGTGGAAAATCCTACCATACAGAAAGACGTTCTTGAGTGTAATG 6407

MW494315.1 CAGTCTCTGAAGAAGTAGTGGAAAATCCTACCATACAGAAAGACGTTCTTGAGTGTAATG 6461

KF686346.1 ATGTGGTTACTCCTGTTATGGAATCGCAGATTAGTACTAAAGAGGTTAAGTTAAAGGGTG 6679

NC_006213.1 CAGAGAGTGGTGCCAAAGAAACCAAAGAAATCAACATTA------TTAAGTTAAGTGGTG 6339

NC_002645.1 ---------------------------------------------TTGCTTATACTGCTT 5855

JX504050.1 ---------------------------------------------TTGCTTATACAACAT 5768

*

NC_019843.3 TAAATAAACCTT---TCGTGAAGGACAATGTCAGTTTCGTTGCTGATGATTCAGGTACTC 6303

NC_004718.3 TGAAAACTACCG---AAGTTGTAGGCAATGTCATACTTAAACCATCAGATGAAGGTGTTA 6448

EPI_ISL_6640916_omicron TGAAAACTACCG---AAGTTGTAGGAGACATTATACTTAAACCAGCAAATAATA---TAA 6461

MW494315.1 TGAAAACTACCG---AAGTTGTAGGAGACATTATACTTAAACCAGCAAATAATAGTTTAA 6518

KF686346.1 TTAGAAAGACTGTTAAAATAGAAGATGCTATTATTGTTAATGATGAAAATAGTTCTATTA 6739

NC_006213.1 TTAAAAAACCATTTAAGGTTGAAGATAGTGTCATTGTTAATGATGATACTAGTGAAACCA 6399

NC_002645.1 TTTCTGGACCTGTTGACAAAGGTCATTATACTGTTTATGATACTGCAAAGAAATCAATGT 5915

JX504050.1 TTTCAGGTTCTTTTGATAACGGTCACTATGTAGTTTATGATGCTGCTAATAATGCTGTCT 5828

* *

NC_019843.3 CCGTTGTTGAGTATCTGTCTAAAGAAGACCTACATACATTGTATGTAGACCCTAAGTATC 6363

NC_004718.3 AAGTAACACAAGAGTTAGGTCATGAGGATCTTATGGCTGCTTATGTGGAAAACA-----C 6503

EPI_ISL_6640916_omicron AAATTACAGAAGAGGTTGGCCACACAGATCTAATGGCTGCTTATGTAGACAATT-----C 6516

MW494315.1 AAATTACAGAAGAGGTTGGCCACACAGATCTAATGGCTGCTTATGTAGACAATT-----C 6573

KF686346.1 AGGTTGTTAAAAGTTTATCTTTAGTTGATGTTTGGGATATGTATTTGACAGGTT------ 6793

NC_006213.1 AATATGTTAAGAGTTTGTCTATTGTTGATGTGTATGATATGTGGCTTACAGGTT------ 6453

NC_002645.1 ATGATGGTGATCGTTTTGTTAAACATGATCTTTCTCTGCTGTCTGTCACATCAG------ 5969

JX504050.1 ATGATGGTGCTCGTTTATTTTCTTCAGATTTGTCTACTTTAGCTGTTACAGCTA------ 5882

* ** * *

NC_019843.3 AAGTCATTGTCTTAAAAGAC--AATGTACTTTCTTCTATGCTTAGATTGCACACCGTTGA 6421

NC_004718.3 AAGCATTACCATTAAGAAACCTAATGAGCTTTCACTAGCCTTAGGTTTAAAAACAATTGC 6563

EPI_ISL_6640916_omicron TAGTCTTACTATTAAGAAACCTAATGAATTATCTAGAGTATTAGGTTTGAAAACCCTTGC 6576

MW494315.1 TAGTCTTACTATTAAGAAACCTAATGAATTATCTAGAGTATTAGGTTTGAAAACCCTTGC 6633

KF686346.1 ------------------------------------------------------------ 6793

NC_006213.1 ------------------------------------------------------------ 6453

NC_002645.1 ------------------------------------------------------------ 5969

JX504050.1 ------------------------------------------------------------ 5882

NC_019843.3 GTCAGGTGATATTAACGTTGTTGCAGCTTCCGGATCTTTGACACGTAAAGTGAAGTTACT 6481

NC_004718.3 CACTCATGGTATTGCTGCAATT-----------AATAGTGTTCCTTGGAGTAAAATTTTG 6612

EPI_ISL_6640916_omicron TACTCATGGTTTAGCTGCTGTT-----------AATAGTGTCCCTTGGGATACTATAGCT 6625

MW494315.1 TACTCATGGTTTAGCTGCTGTT-----------AATAGTGTCCCTTGGGATACTATAGCT 6682

KF686346.1 --------GTGATTATGTTGTTTGGGTTGCTAATGAATTGTCACGCCTAGTTAAATCACC 6845

NC_006213.1 --------GTAAGTATGTTGTTAGAACTGCTAATGCTTTGAGCAGAGCAGTTAACGTACC 6505

NC_002645.1 --------TTGTTATGGTTGGTGGTTATGTTGC--------------------------- 5994

JX504050.1 --------TTGTTGTAGTAGGTGGTTGTGTAAC--------------------------- 5907

* * *

NC_019843.3 ATTTAGGGC--TTCATTTTATTTCAAAGAATTTGCTACCCGCACTTTCACTGCTACCACT 6539

NC_004718.3 GCTTATGTCAAACCATTCTTAGGACAAGCAGCAATTACAACATCAAATTGCGCTAAGAGA 6672

EPI_ISL_6640916_omicron AATTATGCTAAGCCTTTTCTTAACAAAGTTGTTAGTACAACTACTAACATAGTTACACGG 6685

MW494315.1 AATTATGCTAAGCCTTTTCTTAACAAAGTTGTTAGTACAACTACTAACATAGTTACACGG 6742

KF686346.1 CACAGTTAG--GGAATATATACGATATGGTATTAAACCTATTACTATACCTATAGATTTG 6903

NC_006213.1 TACAATACG--TAAGTTTATAAAATTTGGTATGACTCTTGTTAGTATACCAATTGATTTG 6563

NC_002645.1 -----------------------------------------------ACCTGTTAATACA 6007

JX504050.1 -----------------------------------------------ATCTAATGTTCCA 5920

NC_019843.3 GCTGTAGGTAGTTGTATAAAGAGTGTAGTGCGGCATCTAGGTGTTACTAAAGGCATATTG 6599

NC_004718.3 TTAGCACAACGTGTGTTTAACAATTATATGCCTTATGT---------------------- 6710

EPI_ISL_6640916_omicron TGTTTAAACCGTGTTTGTACTAATTATATGCCTTATTT---------------------- 6723

MW494315.1 TGTTTAAACCGTGTTTGTACTAATTATATGCCTTATTT---------------------- 6780

KF686346.1 TTATGTTTAAGAGATGATAATCAAACTCTTTTAGTCCCTAAAATTTTTAAAGCAAGAGCT 6963

NC_006213.1 TTAAATTTAAGAGAGATTAAGCCTGCTGTTAATGTGGTTAAAGCTGTGCGAAATAAAATT 6623

NC_002645.1 GTGA---------AACCTAAACCAGTCATTAATCAACTTGATGAAAAGGCACAGAAGTTC 6058

JX504050.1 ACAATTGTTAGTGAGAAAATTTCTGTTATGGATAAACTTGATACTGGTGCACAAAAATTT 5980

* *

NC_019843.3 ACAGGCTGTTTTAGTTTTGCCAAGATGTTATTTATGCTTCCACTAGCTTACTTTAGTGAT 6659

NC_004718.3 ---------------------------------------------GTTTACATTATTGTT 6725

EPI_ISL_6640916_omicron ---------------------------------------------CTTTACTTTATTGCT 6738

MW494315.1 ---------------------------------------------CTTTACTTTATTGCT 6795

KF686346.1 ATAGAATTTTATGGTTTTTTGAAGTGGTTGTTTATTTATGTT------------------ 7005

NC_006213.1 TCTGTATGCTTTAATTTTATTAAATGGCTTTTTGTCTTATTA------------------ 6665

NC_002645.1 TTTGATTTTGGTGATTTTTTGATTCATAATTTTGTTATTTTT----TTCACATGGTTATT 6114

JX504050.1 TTCCAATTTGGTGATTTTGTTATGAATAACATTGTTCTGTTT----TTAACTTGGTTGCT 6036

NC_019843.3 TCAAAACTCGGCACCACAGAGGTTAAAGTGAGTGCTTTGAAAACAGCCGGCGTTGTGACA 6719

NC_004718.3 CCAATTGT-----GTACTTTTACTAAAAGTACCAATTCTAGAATTAGAGCTTCACTACCT 6780

EPI_ISL_6640916_omicron ACAATTGT-----GTACTTTTACTAGAAGTACAAATTCTAGAATTAAAGCATCTATGCCG 6793

MW494315.1 ACAATTGT-----GTACTTTTACTAGAAGTACAAATTCTAGAATTAAAGCATCTATGCCG 6850

KF686346.1 -------T-----TTAGTTTATTACATTTTACAAATGATAAAACCATTTTTTATACTACA 7053

NC_006213.1 -------T-----TTGGCTGGATTAAAATATCCGCTGATAATAAAGTAATCTACACCACA 6713

NC_002645.1 GAGTATGT-----TTACTTTGTGTAAAACTGCAGTAACTACAGGTGATGTTAAAATAATG 6169

JX504050.1 TAGTATGT-----TTAGTCTTTTACGTACTTCTATTATGAAGCATGATATTAAAGTTATT 6091

* *

NC_019843.3 GGTAATGTTGTAAAACAGTGTTGCACTGCTGCTGTTGATTTAAGTATGGATAAGTT---G 6776

NC_004718.3 ACAACTATTGCTAAAAATAGTGTTAAGAGTGTTGCTAAATTATGTTTGGATGCCG----- 6835

EPI_ISL_6640916_omicron ACTACTATAGCAAAGAATACTGTTAAGAGTGTCGGTAAATTTTGTCTAGAGGCTT----- 6848

MW494315.1 ACTACTATAGCAAAGAATACTGTTAAGAGTGTCGGTAAATTTTGTCTAGAGGCTT----- 6905

KF686346.1 GAAATAGCTTCTAAGTTTACTTTTAATTTGTTTTGTTTGGCTCTTAAAAATGCTTTTCAG 7113

NC_006213.1 GAAATTGCATCAAAGCTTACGTGTAAGCTTGTAGCTTTAGCTTTTAAAAATGCATTTTTG 6773

NC_002645.1 GCCAAAGCACCACAAAGGACGGGTGTTGTTTTAAAACGTAGTCTTAAATATAACTTAAAA 6229

JX504050.1 GCCAAGGCTCCTAAACGTACAGGTGTTATTTTGACACGTAGTTTTAAGTATAACATTAGA 6151

* * * *

NC_019843.3 CGCCGTGTGGATTGGAAATCAACCCTACGGTTGTTACTTATGTTATGCACAACTATGGTA 6836

NC_004718.3 -GCATTAATTATGTGAAGTCACCCAAATTTTCTAAATTGTTCACAATCGCTATGTGGCTA 6894

EPI_ISL_6640916_omicron -CATTTAATTATTTGAAGTCACCTAATTTTTCTAAACTGATAAATATTATAATTTGGTTT 6907

MW494315.1 -CATTTAATTATTTGAAGTCACCTAATTTTTCTAAACTGATAAATATTATAATTTGGTTT 6964

KF686346.1 ACATTTAGATGGAGTATATTTATAAAAGGTTTTCTTGTTGTAGC---CACTGTGTTTTTG 7170

NC_006213.1 ACATTTAAGTGGAGTATGGTTGCTAGAGGTGCTTGCATTATAGC---GACTATATTTCTA 6830

NC_002645.1 GCGTCAGCAGCTGTTCTTAAATCTAAGTGGTGGCTGCTTGCTAAGTTTACGAAACTACTG 6289

JX504050.1 TCTGCTTTGTTTGTTATAAAGCAGAAGTGGTGTGTTATTGTTACTTTGTTTAAGTTCTTA 6211

* *

NC_019843.3 TTGTTGTCTTCTGTGTATCACTTGTATGTCTTCAATCAGGTCTTATCAAGTGATGTTAT- 6895

NC_004718.3 TTGTTGTTAAGTATTTGCTTAGGTTCTCTAATCTGTGTAACTGCTGCTTTTGGTGTACT- 6953

EPI_ISL_6640916_omicron TTACTATTAAGTGTTTGCCTAGGTTCTTTAATCTACTCAACCGCTGCTTTAGGTGTTTT- 6966

MW494315.1 TTACTATTAAGTGTTTGCCTAGGTTCTTTAATCTACTCAACCGCTGCTTTAGGTGTTTT- 7023

KF686346.1 TTTTGGTTTAATTTTTTGTATATAAATGTTATTTTTAGTGACTTTTATCTTCCTAATATT 7230

NC_006213.1 TTGTGGTTTAATTTTATATATGCCAATGTAATTTTTAGTGATTTTTATTTGCCTAAAATC 6890

NC_002645.1 TTACTCATATATACATTGTACTCAGTAGTTTTGCTTTGTGTACGTTTTGGACCGTTTAA- 6348

JX504050.1 TTATTATTATATGCTATTTATGCACTTGTTTTTATGATTGTGCAATTTAGTCCTTTTAAT 6271

** * * *

NC_019843.3 --------------------------------------------------GTTTGAAGAT 6905

NC_004718.3 --------------------------------------------------CTT------- 6956

EPI_ISL_6640916_omicron --------------------------------------------------AAT------- 6969

MW494315.1 --------------------------------------------------AAT------- 7026

KF686346.1 AGTGTTTTTCCTATTTTTGTGGGAAGAATTGTTATGTGGATAAAGGCTACTTT------- 7283

NC_006213.1 GGTTTCTTGCCGACTTTTGTTGGTAAGATTGCACAGTGGATTAAGAACACTTT------- 6943

NC_002645.1 -----TTT------------------------------------------TTG------- 6354

JX504050.1 AGTCTTTT------------------------------------------ATG------- 6282

NC_019843.3 GCCCAAGGTTTGAAAAAGTTCTACAAAGAAGTTAGAGCTTACCTAGGAATCTCTTCTGCT 6965

NC_004718.3 -----------------------------------ATCTAATTTTGGTGCTCCTTCTTAT 6981

EPI_ISL_6640916_omicron -----------------------------------GTCTAATTTAGGCATGCCTTCTTAC 6994

MW494315.1 -----------------------------------GTCTAATTTAGGCATGCCTTCTTAC 7051

KF686346.1 ------------------------------------------------------------ 7283

NC_006213.1 ------------------------------------------------------------ 6943

NC_002645.1 ------------------------------------------------------------ 6354

JX504050.1 ------------------------------------------------------------ 6282

NC_019843.3 TGTGACGGTCTTGCTTCAGCTTATAGGGCGAATTCCTTTGA---------TGTACCTACA 7016

NC_004718.3 TGTAATGGCGTTAGAGAATTGTATCTTAATTCGTCTAACGT---------TACTACTATG 7032

EPI_ISL_6640916_omicron TGTACTGGTTACAGAGAAGGCTATTTGAACTCTACTAATGT---------CACTATTGCA 7045

MW494315.1 TGTACTGGTTACAGAGAAGGCTATTTGAACTCTACTAATGT---------CACTATTGCA 7102

KF686346.1 -----TGGTTTGGTTACAATTTGTGATTTTTATTCTAAGTTAGGTGTAGGTTTTACAAGT 7338

NC_006213.1 -----TAGTCTTGTAACTATTTGTGATCTATATTCCATTCAGGATGTGGGTTTTAAGAAT 6998

NC_002645.1 -----TAGTGAGACTGTTAATGGTTATGCTAAGTCAAACTT---------TGTCAAGGAT 6400

JX504050.1 -----TGGTGACATTGTAAGTGGTTATGAAAAATCCACTTT---------TAATAAGGAT 6328

* * *

NC_019843.3 TTCTGCGCAAACCGTTCTGCAATGTGTAATTGGTGCTTGATTAGCCAAGATTCCATAACT 7076

NC_004718.3 GATTTCTGTGAAGGTTCTTTTCCTTGCAGCATTTGTTTAAGTGGATTAGACTCCCTTGAT 7092

EPI_ISL_6640916_omicron ACCTACTGTACTGGTTCTATACCTTGTAGTGTTTGTCTTAGTGGTTTAGATTCTTTAGAC 7105

MW494315.1 ACCTACTGTACTGGTTCTATACCTTGTAGTGTTTGTCTTAGTGGTTTAGATTCTTTAGAC 7162

KF686346.1 CATTTTTGTAATGGTAGTTTTATATGTGAATTGTGTCATTCTGGTTTTGATATGTTGGAT 7398

NC_006213.1 CAGTATTGTAATGGAAGTATTGCATGTCAGTTCTGCTTGGCAGGATTTGATATGTTAGAT 7058

NC_002645.1 GATTACTGTGATGGTTCATTGGGCTGCAAGATGTGTCTTTTTGGTTACCAAGAGTTAAGT 6460

JX504050.1 ATTTATTGTGGTAATTCTATGGTTTGTAAGATGTGTTTGTTCAGTTATCAAGAGTTTAAT 6388

* ** ** * * *

NC_019843.3 CACTACCCAGCTCTTAAGATGGT------TCAAACACATCTTAGCCACTATGTTCTTAAC 7130

NC_004718.3 TCTTATCCAGCTCTTGAAACCAT------TCAGGTGACGATTTCATCGTACAAGCTAGAC 7146

EPI_ISL_6640916_omicron ACCTATCCTTCTTTAGAAACTAT------ACAAATTACCATTTCATCTTTTAAATGGGAT 7159

MW494315.1 ACCTATCCTTCTTTAGAAACTAT------ACAAATTACCATTTCATCTTTTAAATGGGAT 7216

KF686346.1 ACATATGCAGCTATAGATTTTGTTCAGTATGAAGTAGATAGACGTGTTTTATTTGATTAT 7458

NC_006213.1 AATTATAAAGCCATTGATGTAGTACAGTATGAAGCTGATAGGAGAGCATTTGTTGATTAT 7118

NC_002645.1 CAATTTAGCCATTTGGATGTTGT----GTGGAAGCATATAACAGACCCTTTGT------- 6509

JX504050.1 GATTTGGATCATACTAGTCTTGT----TTGGAAGCACATTCGTGATCCTATAT------- 6437

* * * *

NC_019843.3 ATAGATTGGTTGTGGTTTGCATTTGAGACTGGTTTGGCATACATGCTCTATACCTCGGCC 7190

NC_004718.3 TTGACAATTTTAGGTCTGGCCGCTGAGTGGGTTTTGGCATATATGTTGTTCACAAAATTC 7206

EPI_ISL_6640916_omicron TTAACTGCTTTTGGCTTAGTTGCAGAGTGGTTTTTGGCATATATTCTTTTCACTAGGTTT 7219

MW494315.1 TTAACTGCTTTTGGCTTAGTTGCAGAGTGGTTTTTGGCATATATTCTTTTCACTAGGTTT 7276

KF686346.1 GTTAGTTTAGTCAAATTAATTGTTGAACTCGTTATTGGTTATTCATTATATACAGTATGG 7518

NC_006213.1 ACAGGTGTGTTAAAGATTGTCATTGAATTGATAGTTAGTTACGCCCTGTATACGGCATGG 7178

NC_002645.1 ------------------------------------------------------------ 6509

JX504050.1 ------------------------------------------------------------ 6437

NC_019843.3 TTCAACTGGTTGTTGTTGGCAGGTACATTGCATTATTTCTTTGCA-----CAGACTTCCA 7245

NC_004718.3 TTTTATTTATTAGGTCTTTCAGCTATAATGCAGGTGTTCTTTGGC-----TATTTTGCTA 7261

EPI_ISL_6640916_omicron TTCTATGTACTTGGATTGGCTGCAATCATGCAATTGTTTTTCAGC-----TATTTTGCAG 7274

MW494315.1 TTCTATGTACTTGGATTGGCTGCAATCATGCAATTGTTTTTCAGC-----TATTTTGCAG 7331

KF686346.1 TTTTATCCATTATTTTGTCTTATTGGTTTACAATTATTTACTACATGGTTGCCTGATTTG 7578

NC_006213.1 TTTTATCCATTGTTTGCCCTTATCAGTATTCAGATCTTGACCACTTGGCTGCCTGAGCTT 7238

NC_002645.1 -------------------TTAGTAATATGCAACCTTTCATTGTC---ATGGTTTTGCTG 6547

JX504050.1 -------------------TAATCAGTTTACAACCATTTGTTATA---CTTGTTATTTTG 6475

* ** **

NC_019843.3 TATTTGTAGACTGGCGGTCATACAATTATGCTGTGTCTAGTGCCTTCTGGTTATTCACCC 7305

NC_004718.3 GTCATTT-CATCAGCAATTCTTGGCTCATGTGGTTTATCATTAGTATTGTACAAATGGCA 7320

EPI_ISL_6640916_omicron TACATTT-TATTAGTAATTCTTGGCTTATGTGGTTAATAATTAATCTTGTACAAATGGCC 7333

MW494315.1 TACATTT-TATTAGTAATTCTTGGCTTATGTGGTTAATAATTAATCTTGTACAAATGGCC 7390

KF686346.1 TTTATGTTAGAAACTATGCATTGGTTGATTAGATTTATTGTATTTGTAGCTAATATGTTA 7638

NC_006213.1 TTTATGCTTAGTACATTACATTGGAGTTTTAGGTTGCTGGTGGCTTTAGCTAATATGTTA 7298

NC_002645.1 CTTATATTTGGTGACAATTATTTGAGATGCTTCTTGCTGTATTTTGTTGCTCAGATGATA 6607

JX504050.1 TTAATTTTTGGTAATATGTATTTGCGTTTTGGACTTTTATATTTTGTTGCACAATTTATT 6535

* * * * * *

NC_019843.3 ACATTCCAATGGCGGGTTTGGTACGAATGTATA--------------------------- 7338

NC_004718.3 -----CCCGTTTCTGCAATGGTTAGGATGTACA--------------------------- 7348

EPI_ISL_6640916_omicron -----CCGATTTCAGCTATGGTTAGAATGTACA--------------------------- 7361

MW494315.1 -----CCGATTTCAGCTATGGTTAGAATGTACA--------------------------- 7418

KF686346.1 -----CCTGCTTTTGTCTTGTTGCGGTTTTATA--------------------------- 7666

NC_006213.1 -----CCAGCACATGTGTTTATGAGGTTTTATA--------------------------- 7326

NC_002645.1 -----AGCACAGTTGGTGTTTTTCTAGGTTACAAGGAAACAAATTGGTTCTTGCACTTTA 6662

JX504050.1 -----AGTACTTTTGGTTCTTTCTTAGGCTTTCATCAGAAACAGTGGTTTTTACATTTTG 6590

* * *

NC_019843.3 ------------------------------ATTTGTTAGCATGCCTTTGGCTTTTACGCA 7368

NC_004718.3 ------------------------------TCTTCTTTGCTTCTTTCTACTACATATGGA 7378

EPI_ISL_6640916_omicron ------------------------------TCTTCTTTGCATCATTTTATTATGTATGGA 7391

MW494315.1 ------------------------------TCTTCTTTGCATCATTTTATTATGTATGGA 7448

KF686346.1 ------------------------------TAGTTGTTACTGCTATGTATAAAGTAGTTG 7696

NC_006213.1 ------------------------------TTATTATTGCCTCTTTTATTAAGCTCTTTA 7356

NC_002645.1 TTCCATTTGATGTTATTTGTGATGAACTGCTTGTCACTGTTATTGTTATTAAGGTTATTT 6722

JX504050.1 TGCCGTTTGATGTTTTATGTAATGAGTTTTTAGCTACATTTATTGTCTGCAAAATCGTTT 6650

* *

NC_019843.3 AGTTTTATCAGCATGTAATCAATGGTTGCAAAGATACGGCATGCTTGCTCTGCTATAAGA 7428

NC_004718.3 AGAGCTATGTTCATATCATGGATGGTTGCACCTCTTCGACTTGCATGATGTGCTATAAGC 7438

EPI_ISL_6640916_omicron AAAGTTATGTGCATGTTGTAGACGGTTGTAATTCATCAACTTGTATGATGTGTTACAAAC 7451

MW494315.1 AAAGTTATGTGCATGTTGTAGACGGTTGTAATTCATCAACTTGTATGATGTGTTACAAAC 7508

KF686346.1 GTTTTATTAGGCATATTGTCTATGGTTGTAATAAAGCTGGTTGTTTATTTTGTTATAAAC 7756

NC_006213.1 GCTTGTTTAGGCATGTTGCCTATGGTTGTAGTAAATCTGGTTGTTTGTTTTGTTACAAGA 7416

NC_002645.1 CTTTTGTCAGACATGTGCTTTTTGGTTGTGAAAACCCAGATTGTATTGCGTGTTCTAAGA 6782

JX504050.1 TATTTGTTAGACATATTATTGTTGGCTGTAATAATGCTGACTGTGTAGCTTGTTCTAAAA 6710

*** * ** ** * ** * ** * **

NC_019843.3 GGAACCGACTTACTAGAGTTGAAGCTTCTACCGTTGTCTGTGGTGGAAAACGTACGTTTT 7488

NC_004718.3 GCAATCGTGCCACACGCGTTGAGTGTACAACTATTGTTAATGGCATGAAGAGATCTTTCT 7498

EPI_ISL_6640916_omicron GTAATAGAGCAACAAGAGTCGAATGTACAACTATTGTTAATGGTGTTAGAAGGTCCTTTT 7511

MW494315.1 GTAATAGAGCAACAAGAGTCGAATGTACAACTATTGTTAATGGTGTTAGAAGGTCCTTTT 7568

KF686346.1 GAAATTGTAGTGTTCGTGTTAAGTGTAGTACTATTGTTGGTGGTGTAATTCGTTATTATG 7816

NC_006213.1 GGAATCGTAGTCTACGTGTTAAATGTAGTACTATCGTTGGTGGCATGATACGCTATTACG 7476

NC_002645.1 GTGCTAGACTTAAGAGATTCCCTGTTAACACAATTGTCAATGGTGTGCAACGTTCATTTT 6842

JX504050.1 GTGCTAGACTTAAACGTGTACCACTTCAAACTATTATTAATGGTATGCATAAATCATTCT 6770

* * * * * ** * * *** *

NC_019843.3 ATATCACAGCAAATGGCGGTATTTCATTCTGTCGTAGGCATAATTGGAATTGTGTGGATT 7548

NC_004718.3 ATGTCTATGCAAATGGAGGCCGTGGCTTCTGCAAGACTCACAATTGGAATTGTCTCAATT 7558

EPI_ISL_6640916_omicron ATGTCTATGCTAATGGAGGTAAAGGCTTTTGCAAACTACACAATTGGAATTGTGTTAATT 7571

MW494315.1 ATGTCTATGCTAATGGAGGTAAAGGCTTTTGCAAACTACACAATTGGAATTGTGTTAATT 7628

KF686346.1 ATATTACTGCTAATGGTGGTACTGGTTTTTGTGTTAAACATCAATGGAATTGTTTTAATT 7876

NC_006213.1 ATGTTATGGCTAATGGTGGCACTGGCTTTTGTTCAAAACATCAATGGAATTGCATTGATT 7536

NC_002645.1 ATGTTAATGCAAATGGTGGTAGTAAGTTTTGTAAGAAACATAGATTTTTCTGTGTTGATT 6902

JX504050.1 ATGTTAATGCTAATGGTGGTACTTGTTTCTGTAATAAACATAACTTCTTTTGTGTTAATT 6830

** * ** ***** ** ** ** ** * ** * ***

NC_019843.3 GTGACACTGCAGGTGTGGGGAATACCTTCATCTGTGAAGAAGTCGCAAATGACCTCACTA 7608

NC_004718.3 GTGACACATTTTGCACTGGTAGTACATTCATTAGTGATGAAGTTGCTCGTGATTTGTCAC 7618

EPI_ISL_6640916_omicron GTGATACATTCTGTGCTGGTAGTACATTTATTAGTGATGAAGTTGCGAGAGACTTGTCAC 7631

MW494315.1 GTGATACATTCTGTGCTGGTAGTACATTTATTAGTGATGAAGTTGCGAGAGACTTGTCAC 7688

KF686346.1 GCCATTCTTTTAAACCAGGTAACACTTTTATAACTGTAGAAGCTGCTATAGAACTTTCTA 7936

NC_006213.1 GTGATTCTTATAAACCAGGTAATACTTTTATTACTGTTGAGGCCGCTCTTGATCTATCTA 7596

NC_002645.1 GTGACTCTTATGGTTATGGCAGCACGTTTATAACACCCGAAGTTTCTAGAGAACTTGGTA 6962

JX504050.1 GTGATTCTTTTGGGCCTGGTAATACTTTTATTAATGGTGATATTGCAAGAGAGCTTGGTA 6890

* * * ** * ** ** ** ** * ** *

NC_019843.3 CCGCCCTACGCAGGCCTATTAACGCTACGGATAGATCACATTATTATGTGGATTCCGTTA 7668

NC_004718.3 TCCAGTTTAAAAGACCAATCAACCCTACTGACCAGTCATCGTATATTGTTGATAGTGTTG 7678

EPI_ISL_6640916_omicron TACAGTTTAAAAGACCAATAAATCCTACTGACCAGTCTTCTTACATCGTTGATAGTGTTA 7691

MW494315.1 TACAGTTTAAAAGACCAATAAATCCTACTGACCAGTCTTCTTACATCGTTGATAGTGTTA 7748

KF686346.1 AAGAGCTTAAACGACCTGTAAATCCAACTGATGCTTCACATTATGTAGTTACTGATATTA 7996

NC_006213.1 AGGAATTGAAACGGCCCATTCAGCCTACAGATGTTGCTTATCATACGGTTACTGATGTTA 7656

NC_002645.1 ACATTACCAAAACAAATGTGCAACCAACAGGGCCGGCCTATGTCATGATTGACAAAGTGG 7022

JX504050.1 ATGTTGTTAAAACAGCTGTTCAACCCACAGCTCCTGCATATGTTATTATTGATAAGGTAG 6950

* * * ** * * * *

NC_019843.3 CAGTTAAAGAGACTGTTGTTCAGTTTAATTATCGTAGAGACGGTCAACCATTCTACGAGC 7728

NC_004718.3 CTGTGAAAAATGGCGCGCTTCACCTCTACTTTGACAAGGCTGGTCAAAAGACCTATGAGA 7738

EPI_ISL_6640916_omicron CAGTGAAGAATGGTTCCATCCATCTTTACTTTGATAAAGCTGGTCAAAAGACTTATGAAA 7751

MW494315.1 CAGTGAAGAATGGTTCCATCCATCTTTACTTTGATAAAGCTGGTCAAAAGACTTATGAAA 7808

KF686346.1 AGCAAGTTGGTTGTATGATGCGTTTGTTCTATGATAGAGATGGACAGCGTGTCTACGATG 8056

NC_006213.1 AGCAAGTTGGTTGTTCTATGCGCTTGTTCTATGATCGTGATGGACAGCGCACATATGATG 7716

NC_002645.1 AGTTTGAAAATGGTTTTTACAGATTGTATTCCTGTGAAACATTTTGGCGTTACAACTTTG 7082

JX504050.1 ATTTTGTTAATGGATTTTATCGTCTTTATAGTGGTGACACTTTTTGGCGGTATGACTTTG 7010

* *

NC_019843.3 GGTTTCCCCTCTGCGCTTTTACAAATCTAGATAAGTTGAAGTTCAAAGAGGTCTGTAAAA 7788

NC_004718.3 GACATCCGCTCTCCCATTTTGTCAATTTAGACAATTTGAG------------AGCTAACA 7786

EPI_ISL_6640916_omicron GACATTCTCTCTCTCATTTTGTTAACTTAGACAACCTGAG------------AGCTAATA 7799

MW494315.1 GACATTCTCTCTCTCATTTTGTTAACTTAGACAACCTGAG------------AGCTAATA 7856

KF686346.1 ATGTTGATGCTAGTTTATTTGTAGATATTAATAATCTGTT------------------AC 8098

NC_006213.1 ATGTTAATGCTAGTTTGTTTGTGGATTATAGTAATTTGCT------------------AC 7758

NC_002645.1 ATATAACTGAAAGCAAGTATTCTTGCAAAGAGGTTTTTAA------------------AA 7124

JX504050.1 ACATTACTGAATCTAAGTATAGTTGTAAAGAGGTTCTGAA------------------GA 7052

* * *

NC_019843.3 CTACTACTGGTATACCTGAATACAACTTTATCATCTACGACTCATCAGATCGTGGCCAGG 7848

NC_004718.3 ACACTAAAGGTTCACTGCCTATTAATGTCATAGTTTTTGATGGCAAGTCCAAATGCGACG 7846

EPI_ISL_6640916_omicron ACACTAAAGGTTCATTGCCTATTAATGTTATAGTTTTTGATGGTAAATCAAAATGTGAAG 7859

MW494315.1 ACACTAAAGGTTCATTGCCTATTAATGTTATAGTTTTTGATGGTAAATCAAAATGTGAAG 7916

KF686346.1 ATTCTAAAGTTAAAGTTGTTCCTAATTTGTATGTAGTTGTAGTAGAGAGTGATGCTGATA 8158

NC_006213.1 ATTCTAAGGTTAAGAGTGTGCCTAATATGCATGTTGTGGTAGTGGAAAATGATGCTGATA 7818

NC_002645.1 ATTGTAATGTTTTGGATGATTTCA---------TCGTGTTTAACAATAATGGGACCAATG 7175

JX504050.1 ATTGTAATGTTTTAGAAAATTTTA---------TTGTTTACAATAATAGTGGTAGTAACA 7103

** * * * * *

NC_019843.3 AAAGTTTAGCTAGGTCTGCATGTGTTTATTATTCTCAAGTCTTGTGTAAATCAATTCTTT 7908

NC_004718.3 AGTCTGCTTCTAAGTCTGCTTCTGTGTACTACAGTCAGCTGATGTGCCAACCTATTCTGT 7906

EPI_ISL_6640916_omicron AATCATCTGCAAAATCAGCGTCTGTTTACTACAGTCAGCTTATGTGTCAACCTATACTGT 7919

MW494315.1 AATCATCTGCAAAATCAGCGTCTGTTTACTACAGTCAGCTTATGTGTCAACCTATACTGT 7976

KF686346.1 GAGCTAATTTTCTGAATGCTGTTGTGTTTTATGCACAATCATTGTATAGGCCTATATTAC 8218

NC_006213.1 AAGCCAATTTTCTGAATGCTGCTGTATTTTATGCACAGTCTTTGTTTAGACCTATTTTAA 7878

NC_002645.1 TAACGCAGGTTAAAAATGCTAGTGTTTACTTTTCACAGTTGTTGTGTAGGCCCATTAAAT 7235

JX504050.1 TTACACAGATTAAAAATGCTTGTGTTTATTTTTCTCAATTGTTGTGTGAACCTATAAAGT 7163

** *** * * ** *** * **

NC_019843.3 TGGTTGACTCAAGTTTGGTTACTTCTGTTGGTGATTCTAGTGAAATCGCCACTAAAATGT 7968

NC_004718.3 TGCTTGACCAAGCTCTTGTATCAGACGTTGGAGATAGTACTGAAGTTTCCGTTAAGATGT 7966

EPI_ISL_6640916_omicron TACTAGATCAGGCATTAGTGTCTGATGTTGGTGATAGTGCGGAAGTTGCAGTTAAAATGT 7979

MW494315.1 TACTAGATCAGGCATTAGTGTCTGATGTTGGTGATAGTGCGGAAGTTGCAGTTAAAATGT 8036

KF686346.1 TTGTAGACAAAAAGTTAATTACTACAGCTTGTAATGGTATCTCTGTAACCCAGACTATGT 8278

NC_006213.1 TGGTTGATAAAAATCTGATAACTACTGCTAACACTGGTACGTCTGTTACAGAAACTATGT 7938

NC_002645.1 TAGTTGACAGTGAACTTTTGTCCACTTTGTCAGTTGATTTTAATGGTGTCTTACACAAGG 7295

JX504050.1 TGGTAAATTCAGAGTTGTTGTCAACTTTATCTGTTGATTTTAATGGTGTTTTGCATAAGG 7223

* * * * * * * * * *

NC_019843.3 TTGATTCCTTTGTTAATAGTTTCGTCTCGCTGTATAATGTCACACGCGATAAGTTGGAAA 8028

NC_004718.3 TTGATGCTTATGTCGACACCTTTTCAGCAACTTTTAGTGTTCCTATGGAAAAACTTAAGG 8026

EPI_ISL_6640916_omicron TTGATGCTTACGTTAATACGTTTTCATCAACTTTTAACGTACCAATGGAAAAACTCAAAA 8039

MW494315.1 TTGATGCTTACGTTAATACGTTTTCATCAACTTTTAACGTACCAATGGAAAAACTCAAAA 8096

KF686346.1 TTGATGTTTATGTTGATACTTTTATGTCTCATTTTGATGTTGATAGAAAGAGTTTTAATA 8338

NC_006213.1 TTGATGTTTATGTGGATACATTTTTGTCTATGTTTGATGTGGATAAAAAGAGTCTTAATG 7998

NC_002645.1 CATACATTGATGT----------------------------------------------- 7308

JX504050.1 CATATGTTGATGT----------------------------------------------- 7236

* **

NC_019843.3 AACTTATCTCTACTGCTCGTGATGGCGTAAGGCGAGGCGATAACTTCCATAGTGTCTTAA 8088

NC_004718.3 CACTTGTTGCTACAGCTCACAGCGAGTTAGCAAAGGGTGTAGCTTTAGATGGTGTCCTTT 8086

EPI_ISL_6640916_omicron CACTAGTTGCAACTGCAGAAGCTGAACTTGCAAAGAATGTGTCCTTAGACAATGTCTTAT 8099

MW494315.1 CACTAGTTGCAACTGCAGAAGCTGAACTTGCAAAGAATGTGTCCTTAGACAATGTCTTAT 8156

KF686346.1 ATTTTGTTAATATTGCTCATGCTTCTCTTAGAGAGGGTGTGCAATTAGAAAAGGTTTTAG 8398

NC_006213.1 CTTTAATAGCAACTGCGCATTCTTCTATAAAACAGGGTACGCAGATTTATAAAGTTTTGG 8058

NC_002645.1 -------------------------------ACTACGTAATAGCTTTGGTAAAGATCTTA 7337

JX504050.1 -------------------------------TTTGTGTAATAGTTTTTTTAAGGAGTTAA 7265

* * *

NC_019843.3 CAACATTCATTGACGCAGCACGAGGCCCCGCAGGTGTGGAGTCTGATGTTGAGACCAATG 8148

NC_004718.3 CTACATTCGTGTCAGCTGCCCGA---CAAGGTGTTGTTGATACCGATGTTGACACAAAGG 8143

EPI_ISL_6640916_omicron CTACTTTTATTTCAGCAGCTCGG---CAAGGGTTTGTTGATTCAGATGTAGAAACTAAAG 8156

MW494315.1 CTACTTTTATTTCAGCAGCTCGG---CAAGGGTTTGTTGATTCAGATGTAGAAACTAAAG 8213

KF686346.1 ATACTTTTGTGGGATGTGTACGTAAATGTTGTTCCATTGATTCAGATGTTGAAACAAGAT 8458

NC_006213.1 ATACCTTTTTAAGCTGTGCTCGTAAAAGTTGTTCTATTGATTCAGATGTTGATACTAAGT 8118

NC_002645.1 ATGCTAATATGTCTTTAGCCGAGTGCAAGAGAGCTTTAGGCCTGTCTATTAGTGATCATG 7397

JX504050.1 CTGCTAACATGTCCATGGCTGAATGTAAAGCTACACTTGGTTTGACTGTTTCTGATGATG 7325

* * * * * * *

NC_019843.3 AAATTGTTGACTCTGTGCAGTATGCTCATAAACATGACATACAAATTACTAATGAGAGCT 8208

NC_004718.3 ATGTTATTGAATGTCTCAAACTTTCACATCACTCTGACTTAGAAGTGACAGGTGACAGTT 8203

EPI_ISL_6640916_omicron ATGTTGTTGAATGTCTTAAATTGTCACATCAATCTGACATAGAAGTTACTGGCGATAGTT 8216

MW494315.1 ATGTTGTTGAATGTCTTAAATTGTCACATCAATCTGACATAGAAGTTACTGGCGATAGTT 8273

KF686346.1 TTATTACTAAATCTATGATATCTGCAGTAGCTGCTGGTTTGGAATTTACTGATGAAAATT 8518

NC_006213.1 GTTTAGCTGATTCTGTCATGTCTGCTGTATCGGCAGGTCTTGAATTGACGGATGAAAGTT 8178

NC_002645.1 AATTTACTAGTGCTATTTCTAATGCACATCGTTGTGACGTGTTGTTATCTGATTTGTCAT 7457

JX504050.1 ATTTTGTTTCAGCTGTTGCCAATGCACATAGGTATGACGTTTTGCTTTCAGATTTGTCAT 7385

* * * * * * * * * *

NC_019843.3 ACAATAATTATGTACCCTCATATGTTAAACCTGATAGTGTGTCTACCAG---CGATTTAG 8265

NC_004718.3 GTAACAATTTCATGCTCACCTATAATAAGGTTGAAAACATGACGCCCAG---AGATCTTG 8260

EPI_ISL_6640916_omicron GTAATAACTATATGCTCACCTATAACAAAGTTGAAAACATGACACCCCG---TGACCTTG 8273

MW494315.1 GTAATAACTATATGCTCACCTATAACAAAGTTGAAAACATGACACCCCG---TGACCTTG 8330

KF686346.1 ATAACAATTTGGTACCTACATATTTAAAGAGTGATAATATTGTAGCTGC---TGATTTAG 8575

NC_006213.1 GTAATAACTTGGTGCCAACATATTTGAAGAGTGACAACATTGTGGCAGC---TGATTTAG 8235

NC_002645.1 TTAACAACTTTGTCAGTTCGTATGCTAAACCTGAGGAAAAATTATCAGCTTATGACTTGG 7517

JX504050.1 TTAATAATTTTTTTATTTCTTATGCTAAACCTGAAGATAAGTTGTCCGTTTATGACATTG 7445

** ** * * * *** ** *** * ** * *

NC_019843.3 GTAGTCTCATTGATTGTAATGCGGCTTCAGTTAACCAAATTGTCTTGCGTAATTCTAATG 8325

NC_004718.3 GCGCATGTATTGACTGTAATGCAAGGCATATCAATGCCCAAGTAGCAAAAAGTCACAATG 8320

EPI_ISL_6640916_omicron GTGCTTGTATTGACTGTAGTGCGCGTCATATTAATGCGCAGGTAGCAAAAAGTCACAACA 8333

MW494315.1 GTGCTTGTATTGACTGTAGTGCGCGTCATATTAATGCGCAGGTAGCAAAAAGTCACAACA 8390

KF686346.1 GTGTTCTTATACAGAATGGTGCTAAGCATGTACAGGGTAATGTTGCTAAGGCAGCTAATA 8635

NC_006213.1 GTGTTCTGATTCAAAATTCTGCAAAGCATGTGCAGGGTAATGTTGCTAAAATAGCTGGTG 8295

NC_002645.1 CGTGTTGTATGCGTGCAGGTGCTAAGGTTGTTAATGCCAATGTTCTGACAAAGGACCAAA 7577

JX504050.1 CTTGTTGTATGCGTGCCGGTTCTAAGGTTGTTAACCATAATGTTTTAATTAAAGAGTCAA 7505

** * * * * **

NC_019843.3 GTGCTTGCATTTGGAACGCTGCTGCATATATGAAACTCTCGGATGCACTTAAACGACAGA 8385

NC_004718.3 TTTCACTCATCTGGAATGTAAAAGACTACATGTCTTTATCTGAACAGCTGCGTAAACAAA 8380

EPI_ISL_6640916_omicron TTACTTTGATATGGAACGTTAAAGATTTCATGTCATTGTCTGAACAACTACGAAAACAAA 8393

MW494315.1 TTGCTTTGATATGGAACGTTAAAGATTTCATGTCATTGTCTGAACAACTACGAAAACAAA 8450

KF686346.1 TTTCTTGTATATGGTTTATTGATGCTTTTAATCAACTTACTGCTGATTTACAGCATAAAT 8695

NC_006213.1 TTTCCTGTATATGGTCTGTGGATGCTTTTAATCAGTTTAGTTCTGATTTCCAGCATAAAT 8355

NC_002645.1 CTCCTATTGTTTGGCATGCAAAGGATTTTAACAGTCTTTCTGCTGAAGGTCGCAAGTATA 7637

JX504050.1 TACCTATTGTTTGGGGTGTCAAGGACTTTAATACTCTTTCTCAAGAAGGTAAGAAGTACC 7565

* * *** * * * * *

NC_019843.3 TTCGCATTGCATGCCGTAAGTGTAATTTAGCTTTCCGGTTAACCACCTCAAAGCTACGCG 8445

NC_004718.3 TTCGTAGTGCTGCCAAGAAGAACAACATACCTTTTAGACTAACTTGTGCTACAACTAGAC 8440

EPI_ISL_6640916_omicron TACGTAGTGCTGCTAAAAAGAATAACTTACCTTTTAAGTTGACATGTGCAACTACTAGAC 8453

MW494315.1 TACGTAGTGCTGCTAAAAAGAATAACTTACCTTTTAAGTTGACATGTGCAACTACTAGAC 8510

KF686346.1 TAAAAAAAGCATGTGTTAAAACTGGCTTGAAGTTAAAATTGACTTTTAATAAGCAAGAGG 8755

NC_006213.1 TGAAGAAAGCATGTTGTAAAACTGGTTTGAAACTGAAGCTTACTTATAATAAGCAGATGG 8415

NC_002645.1 TTGTAAAAACTAGCAAAGCTAAGGGTTTGACTTTCTTGTTGACAATTAATGAAAACCAAG 7697

JX504050.1 TTGTTAAAACAACTAAAGCAAAGGGTTTGACTTTTTTATTAACTTTTAATGATAACCAAG 7625

* * * * * * **

NC_019843.3 CTAATGATAATATCTTATCAGTTAGATTCACTGCTAACAAAATTGTTGGTGGTGCTCCTA 8505

NC_004718.3 AGGTTGTCAATGTCATAACTACTAAAATC---TCACTCAAGGGTGGTAAGATTGTTAGTA 8497

EPI_ISL_6640916_omicron AAGTTGTTAATGTTGTAACAACAAAGATA---GCACTTAAGGGTGGTAAAATTGTTAATA 8510

MW494315.1 AAGTTGTTAATGTTGTAACAACAAAGATA---GCACTTAAGGGTGGTAAAATTGTTAATA 8567

KF686346.1 CAAGTGTCCCTATTCTTACAACACCCTTT---TCACTTAAAGGAGGTGTTGTATTGAGTA 8812

NC_006213.1 CTAATGTCTCTGTTTTAACTACACCCTTT---AGTCTTAAAGGGGGTGCAGTTTTTAGTT 8472

NC_002645.1 CTGTCACGCAAATACCTGCAACTAGCATT---GTTGCTAAGCAAGGTGCTGGTGATGCTG 7754

JX504050.1 CAATTACACAAGTTCCTGCTACTAGTATA---GTTGCAAAACAGGGTGCTGGTTTTAAAC 7682

* * * ** * *

NC_019843.3 CATGGT----------TTAATGCGTTGCGTGACTTTACGTTAAAGGGTTATG-TTCTTGC 8554

NC_004718.3 CTTGTT----------TTAAACTTATGCTTAAGGCCACATTATTGTGCGTTC-TTGCTGC 8546

EPI_ISL_6640916_omicron ATTGGT----------TGAAGCAGTTAATTAAAGTTACACTTGTGTTCCTTT-TTGTTGC 8559

MW494315.1 ATTGGT----------TGAAGCAGTTAATTAAAGTTACACTTGTGTTCCTTT-TTGTTGC 8616

KF686346.1 ATTTGT----------TATATATATTATTTTTTGTTAGTTTAATCTGTTTTATATTATTG 8862

NC_006213.1 ATTTTG----------TTTATGTGTGTTTTGTGTTGAGTTTGGTCTGTTTTATTGGACTG 8522

NC_002645.1 GCCATTCATTAACATGGCTGTGGCTACTGTGTGGTCTTGTGTGTTTGATTCA-ATTCTAC 7813

JX504050.1 GTACTT-----ATAATTTTCTGTGGTATGTATGTTTATTTGTTGTTGCATTG-TTTATTG 7736

*

NC_019843.3 TACCATTATTGTGTTTCTGTGTGCTGTACTGATGTATTTGTGTTTACCTACATTTTCTAT 8614

NC_004718.3 ATTGGTTTGT------------------------TATATCGTTATGCCAGTACATACATT 8582

EPI_ISL_6640916_omicron TGCTATTTTC------------------------TATTTAATAACACCTGTTCATGTCAT 8595

MW494315.1 TGCTATTTTC------------------------TATTTAATAACACCTGTTCATGTCAT 8652

KF686346.1 TGGGCTTTAT----------------------------------TGCCTACATATAGTGT 8888

NC_006213.1 TGGTGCTTAA----------------------------------TGCCCACTTACACAGT 8548

NC_002645.1 TTGTGCTTTT----------------------------------TCATGCCCTATTTTAT 7839

JX504050.1 GTGTCTCATT----------------------------------TATTGA-------TTA 7755

NC_019843.3 GGCACCTGTTGAATTTTATGAAGACCGCATCTTGGACTTTAAAGTTCTTGATAATGGTAT 8674

NC_004718.3 GTCAATCCATGATGGTTACACAAATGAAATCATTGGTTACAAAGCCATTCAGGATGGTGT 8642

EPI_ISL_6640916_omicron GTCTAAACATACTGACTTTTCAAGTGAAATCATAGGATACAAGGCTATTGATGGTGGTGT 8655

MW494315.1 GTCTAAACATACTGACTTTTCAAGTGAAATCATAGGATACAAGGCTATTGATGGTGGTGT 8712

KF686346.1 TTATAAGTCTGATATTCATTTGCCTGCTTATGCTAGTTTTAAAGTTATTGATAATGGTGT 8948

NC_006213.1 ACACAAATCAGATTTTCAGCTTCCCGTTTATGCCAGTTATAAAGTTTTAGATAATGGTGT 8608

NC_002645.1 GTACGATATCGTGAGTAGTTTTGAGGGTTATGA---TTTTAAGTATATAGAAAATGGTCA 7896

JX504050.1 TACAACCACTGTAACTAGCTTTCATGGTTATGA---TTTTAAGTACATTGAGAATGGTCA 7812

* ** * * ****

NC_019843.3 CATTAGGGATGTAAATCCTGATGATAAGTGCTTTGCTAATAAGCACCGGTCCTTCACACA 8734

NC_004718.3 CACTCGTGACATCATTTCTACTGATGATTGTTTTGCAAATAAACATGCTGGTTTTGACGC 8702

EPI_ISL_6640916_omicron CACTCGTGACATAGCATCTACAGATACTTGTTTTGCTAACAAACATGCTGATTTTGACAC 8715

MW494315.1 CACTCGTGACATAGCATCTACAGATACTTGTTTTGCTAACAAACATGCTGATTTTGACAC 8772

KF686346.1 TGTTAGAGATATTTCAGTTAATGATTTATGTTTTGCTAATAAATTTTTCCAATTTGATCA 9008

NC_006213.1 TATTAGAGATGTTAGCGTTGAAGATGTTTGTTTCGCTAACAAATTTGAACAATTTGATCA 8668

NC_002645.1 GTTGAAGAATTTTGAAGCGCCACTTAAATGCGTCAGAAACGTTTTTGAAAACTTTGAGGA 7956

JX504050.1 GTTGAAGGTGTTTGAAGCACCTTTACACTGTGTTCGTAATGTTTTTGATAATTTTAATCA 7872

* ** * ** **

NC_019843.3 ATGGTATCATGAGCATGTTGGTGGTGTCTATGACAACTCTATCA---CATGCCCATTGAC 8791

NC_004718.3 ATGGTTTAGCCAGCGTGGTGGTTCA---TACAAAAATGACAAAA---GCTGCCCTGTAGT 8756

EPI_ISL_6640916_omicron ATGGTTTAGCCAGCGTGGTGGTAGT---TATACTAATGACAAAG---CTTGCCCATTGAT 8769

MW494315.1 ATGGTTTAGCCAGCGTGGTGGTAGT---TATACTAATGACAAAG---CTTGCCCATTGAT 8826

KF686346.1 ATGGTATGAGTCCACTTTTGGGTCTGTTTACTATCATAATTCTATGGATTGCCCTATTGT 9068

NC_006213.1 ATGGTATGAGTCTACATTTGGTCTAAGTTATTATAGTAACAGTATGGCTTGTCCCATTGT 8728

NC_002645.1 CTGGCATTATGCTAAGTTTGGCTTCACACCTTTAAACAAGCAAA---GCTGTCCTATTGT 8013

JX504050.1 ATGGCATGAGGCTAAGTTTGGTGTTGTTACTACTAATAGTGATA---AATGTCCTATAGT 7929

*** * *** ** ** *

NC_019843.3 AGTTGCAGTAATTGCTGGAGTTGCTGGTGCTCGCATTCCAGACGTACCTACTACATTGGC 8851

NC_004718.3 AGCTGCTATCATTACAAGAGAGATTGGTTTCATAGTGCCTGGCTTACCGGGTACTGTGCT 8816

EPI_ISL_6640916_omicron TGCTGCAGTCATAACAAGAGAAGTGGGTTTTGTCGTGCCTGGTTTGCCTGGCACGATATT 8829

MW494315.1 TGCTGCAGTCATAACAAGAGAAGTGGGTTTTGTCGTGCCTGGTTTGCCTGGCACGATATT 8886

KF686346.1 AGTGGCAGTTATGGATGAAGATATCGGTTCTACTATGTTTAATGTTCCTACTAAAGTTTT 9128

NC_006213.1 TGTTGCTGTAATAGATCAGGATTTTGGCTCTACAGTGTTTAATGTCCCTACCAAAGTGTT 8788

NC_002645.1 AGTTGGAGT------TTCTGAAATTGTTAATACTGTCGCTGGCATTCCATCTAATGTGTA 8067

JX504050.1 TGTTGGTGT------TTCAGAGCGTATTAATGTTGTTCCTGGTGTTCCAACAAATGTATA 7983

* * * * * * ** * *

NC_019843.3 TTGGGTGAACAATCAGATAATTTTC---TTTGTTTCTCGAGTCTTTGCTAATACAGGCAG 8908

NC_004718.3 GAGAGCAATCAATGGTGACTTCTTGCATTTTCTACCTCGTGTTTTTAGTGCTGTTGGCAA 8876

EPI_ISL_6640916_omicron ACGCACAACTAATGGTGACTTTTTGCATTTCTTACCTAGAGTTTTTAGTGCAGTTGGTAA 8889

MW494315.1 ACGCACAACTAATGGTGACTTTTTGCATTTCTTACCTAGAGTTTTTAGTGCAGTTGGTAA 8946

KF686346.1 GAGACATGGCTTTCATG---TTTTACATTTTTTAACTTATGCATTTGCTAGTGATAGTGT 9185

NC_006213.1 ACGATATGGTTATCATG---TGTTGCACTTTATTACACATGCACTTTCTGCTGATGGAGT 8845

NC_002645.1 TCTTGTTGGTAAAACTT---TAAT---TTTTACACTACAAGCTGCTTTTGGTAATGCTGG 8121

JX504050.1 TTTGGTAGGAAAGACTC---TTGT---TTTTACATTACAGGCTGCTTTTGGAAACACAGG 8037

* * ** * * *

NC_019843.3 TGTTTGCTACACTCCTATAGATGAGATACCCTATAAGAGTTTCTCTGATAGTGGTTGCAT 8968

NC_004718.3 CATTTGCTACACACCTTCCAAACTCATTGAGTATAGTGATTTTGCTACCTCTGCTTGCGT 8936

EPI_ISL_6640916_omicron CATCTGTTACACACCATCAAAACTTATAGAGTACACTGACTTTGCAACATCAGCTTGTGT 8949

MW494315.1 CATCTGTTACACACCATCAAAACTTATAGAGTACACTGACTTTGCAACATCAGCTTGTGT 9006

KF686346.1 TCAGTGCTATACACCACATATTCAGATTTCTTATAATGATTTTTATGCTAGTGGTTGTGT 9245

NC_006213.1 GCAGTGTTATACGCCACATAGTCAAATATCGTATTCTAATTTTTATGCTAGTGGCTGTGT 8905

NC_002645.1 TGTTTGTTATGACATTTTTGGAGTCACAACACCTGAAAA---------------GTGCAT 8166

JX504050.1 TGTTTGTTATGACTTTGATGGTGTTACCACTAGTGATAA---------------GTGTAT 8082

** ** * ** *

NC_019843.3 TCTTCCATCTGAGTGCACTATGTTTAGGGATGCAGAGGGCCGTATGACACCATACTGCCA 9028

NC_004718.3 TCTTGCTGCTGAGTGTACAATTTTTAAGGATGCTATGGGCAAACCTGTGCCATATTGTTA 8996

EPI_ISL_6640916_omicron TTTGGCTGCTGAATGTACAATTTTTAAAGATGCTTCTGGTAAGCCAGTACCATATTGTTA 9009

MW494315.1 TTTGGCTGCTGAATGTACAATTTTTAAAGATGCTTCTGGTAAGCCAGTACCATATTGTTA 9066

KF686346.1 TTTATCATCTTTGTGTACTATGTTTAAAAGAGGTGATGGTACACCACATCCTTATTGTTA 9305

NC_006213.1 GCTTTCCTCTGCTTGCACTATGTTTACAATGGCCGATGGTAGTCCACAACCTTATTGTTA 8965

NC_002645.1 TTTTACTTCTGCTTGTACTAGATTAGAA---GGTTTGGGTGGTAACAATGTTTATTGTTA 8223

JX504050.1 TTTTAATTCTGCTTGTACTAGGTTGGAA---GGTTTGGGTGGTGACAATGTTTATTGTTA 8139

* ** ** ** * ** * ** ** ** *

NC_019843.3 TGATCCTACTGTTTTGCCTGGGGCTTTTGCGTACAGTCAGATGAGGCCTCATGTTCGTTA 9088

NC_004718.3 TGACACTAATTTGCTAGAGGGTTCTATTTCTTATAGTGAGCTTCGTCCAGACACTCGTTA 9056

EPI_ISL_6640916_omicron TGATACCAATGTACTAGAAGGTTCTGTTGCTTATGAAAGTTTACGCCCTGACACACGTTA 9069

MW494315.1 TGATACCAATGTACTAGAAGGTTCTGTTGCTTATGAAAGTTTACGCCCTGACACACGTTA 9126

KF686346.1 TTCAGATGGTGTTATGAAGAATGCTTCTTTGTATACATCTTTGGTTCCACATACACGTTA 9365

NC_006213.1 TACAGAGGGGCTTATGCAAAATGCTTCTCTGTATAGTTCATTGGTACCTCACGTGCGGTA 9025

NC_002645.1 TAACACAGCGCTTATGGAAGGTTCTTTGCCTTACAGTTCAATACAAGCTAATGCATATTA 8283

JX504050.1 CAACACTGGTCTTATTGAAGGTTCTAAACCTTATAGTACTTTACAGCCCAATGCGTATTA 8199

* * ** ** * * * **

NC_019843.3 CGACTTGTATGATGGTAACATGTTTATTAAATTTCCTGAAGTAGTATTTGAAAGTACAC- 9147

NC_004718.3 TGTGCTTATGGATGGTTCCA---TCATACAGTTTCCTAACACTTACCTGGAGGGTTCTG- 9112

EPI_ISL_6640916_omicron TGTGCTCATGGATGGCTCTA---TTATTCAATTTCCTAACACCTACCTTGAAGGTTCTG- 9125

MW494315.1 TGTGCTCATGGATGGCTCTA---TTATTCAATTTCCTAACACCTACCTTGAAGGTTCTG- 9182

KF686346.1 TAGCCTTGCTAATTCTAATGGTTTTATAAGATTTCCTGATGTTATTAGTGAAGGTATTG- 9424

NC_006213.1 TAATCTTGCTAATGCTAAAGGTTTTATCCGTTTTCCAGAAGTGTTGCGAGAAGGGCTTG- 9084

NC_002645.1 TAAATATG---ACAATGGCAATTTTATTAAGTTGCCAGAAGTTATTGCACAAGGCTTTGG 8340

JX504050.1 TAAGTATG---ATGCTAAAAATTATGTACGTTTTCCAGAAATTTTAGCTAGAGGTTTTGG 8256

* * ** ** * *

NC_019843.3 --TTAGGATTACTAGAACTCTGTCAACTCAGTACTGCCGGTTCGGTAGTTGTGAGTATGC 9205

NC_004718.3 --TTAGAGTAGTAACAACTTTTGATGCTGAGTACTGTAGACATGGTACATGCGAAAGGTC 9170

EPI_ISL_6640916_omicron --TTAGAGTGGTAACAACTTTTGATTCTGAGTACTGTAGGCACGGCACTTGTGAAAGATC 9183

MW494315.1 --TTAGAGTGGTAACAACTTTTGATTCTGAGTACTGTAGGCACGGCACTTGTGAAAGATC 9240

KF686346.1 --TACGTATTGTAAGAACGCGCTCTATGACTTATTGTAGAGTGGGTGCATGTGAATACGC 9482

NC_006213.1 --TACGTATCGTGCGTACTCGTTCTATGTCGTATTGCAGAGTTGGATTATGTGAGGAAGC 9142

NC_002645.1 TTTTAGAACAGTGCGTACTATTGCCACCAAATACTGCCGCGTAGGTGAATGTGTTGAATC 8400

JX504050.1 CTTACGTACTATTAGAACTTTGGCTACACGTTATTGTAGAGTTGGTGAATGCCGTGACTC 8316

* * ** ** ** * ** ** *

NC_019843.3 ACAAGAGGGTGTTTGTATTACCACAAATGGCTCGTGGGCCATTTTTAATGACCACCATCT 9265

NC_004718.3 AGAAGTAGGTATTTGCCTATCTACCAGTGGTAGATGGGTTCTTAATAATGAGCATTACAG 9230

EPI_ISL_6640916_omicron AGAAGCTGGTGTTTGTGTATCTACTAGTGGTAGATGGGTACTTAACAATGATTATTACAG 9243

MW494315.1 AGAAGCTGGTGTTTGTGTATCTACTAGTGGTAGATGGGTACTTAACAATGATTATTACAG 9300

KF686346.1 CGAAGAGGGTATATGTTTTAATTTTAATAGTTCCTGGGTTTTGAATAATGATTATTATAG 9542

NC_006213.1 TGATGAGGGTATATGCTTTAATTTTAATGGTTCTTGGGTGCTTAATAATGATTATTATAG 9202

NC_002645.1 CAATGCAGGTGTGTGTTTTGGCTTTGACAAGTGGTTTGTTAACGATGGACGTGTTGCCA- 8459

JX504050.1 ACATAAAGGTGTTTGTTTTGGTTTTGATAAATGGTATGTTAATGATGGACGTGTTGATG- 8375

* *** * ** * * *

NC_019843.3 TAATAGACCTGGTGTCTATTGTGGCTCTGATTTTATTGACATTGTCAGGCGGTTAGCAGT 9325

NC_004718.3 AGCTCTATCAGGAGTTTTCTGTGGTGTTGATGCGATGAATCTCATAGCTAACATCTTTAC 9290

EPI_ISL_6640916_omicron ATCTTTACCAGGAGTTTTCTGTGGTGTAGATGCTGTAAATTTACTTACTAATATGTTTAC 9303

MW494315.1 ATCTTTACCAGGAGTTTTCTGTGGTGTAGATGCTGTAAATTTACTTACTAATATGTTTAC 9360

KF686346.1 AAGTATGCCTGGAACTTTTTGTGGTAGAGATCTTTTTGATTTGTTTTATCAATTTTTTAG 9602

NC_006213.1 ATCATTGCCTGGGACCTTTTGTGGTAGAGATGTTTTTGATTTAATTTATCAGCTATTTAA 9262

NC_002645.1 --------ATGGTTACGTTTGTGGTACTGGTTTGTGGAACCTTGTATTTAACATACTTTC 8511

JX504050.1 --------ACGGTTACATTTGTGGTGATGGTCTTATAGACCTTCTTGTTAATGTACTCTC 8427

** ***** * * * * * *

NC_019843.3 ATCACTGTTCCAGCCTATTACTTATTTCCAATTGACTACCTCATTGGTCTTGGGTATAGG 9385

NC_004718.3 TCCTCTTGTGCAACCTGTGGGTGCTTTAGATGTGTCTGCTTCAGTAGTGGCTGGTGGTAT 9350

EPI_ISL_6640916_omicron ACCACTAATTCAACCTATTGGTGCTTTGGACATATCAGCATCTATAGTAGCTGGTGGTAT 9363

MW494315.1 ACCACTAATTCAACCTATTGGTGCTTTGGACATATCAGCATCTATAGTAGCTGGTGGTAT 9420

KF686346.1 TAGTTTAATTCGTCCTATAGATTTCTTTTCTCTTACTGCTAGTTCTATTTTTGGAGCTAT 9662

NC_006213.1 AGGTTTAGCACAGCCTGTGGATTTTTTGGCATTGACTGCTAGTTCCATTGCTGGTGCTAT 9322

NC_002645.1 CATGTTTTCATCTTCATTCTCTGTTGCTGCAATGTCAGGTCAAATTTTACTTAATTGTGC 8571

JX504050.1 AATCTTTAGTTCATCTTTTAGCGTTGTGGCTATGTCTGGACATATGTTGTTTAATTTTCT 8487

* * * * * *

NC_019843.3 TTTGTGTGCGTTCCTGACTTTGCTCTTCTATTATATTAATAAAGTAAAACGTGCTTTTGC 9445

NC_004718.3 TATTGCCATATTGGTGACTTGTGCTGCCTACTACTTTATGAAATTCAGACGTGTTTTTGG 9410

EPI_ISL_6640916_omicron TGTAGCTATCGTAGTAACATGCCTTGCCTACTATTTTATGAGGTTTAGAAGAGCTTTTGG 9423

MW494315.1 TGTAGCTATCGTAGTAACATGCCTTGCCTACTATTTTATGAGGTTTAGAAGAGCTTTTGG 9480

KF686346.1 ATTGGCTATAGTTGTTGTCTTGGTTTTTTATTATTTAATAAAACTTAAGCGTGCTTTTGG 9722

NC_006213.1 ACTCGCTGTAATTGTTGTTTTGGTGTTTTATTACCTAATAAAGCTTAAACGTGCTTTTGG 9382

NC_002645.1 ATTAGGTGCTTTTGCTATTTTTTGTTGTTTTCTTGTGACAAAGTTTAGACGCATGTTTGG 8631

JX504050.1 TTTTGCAGCATTTATTACATTTTTGTGCTTTTTAGTTACTAAATTTAAACGTGTTTTTGG 8547

* * * * * * * * * * ****

NC_019843.3 AGATTACACCCAGTGTGCTGTAATTGCTGTTGTTGCTGCTGTTCTTAATAGCTTGTGCAT 9505

NC_004718.3 TGAGTACAACCATGTTGTTGCTGCTAATGCACTTTTGTTTTTGATGTCTTTCACTATACT 9470

EPI_ISL_6640916_omicron TGAATACAGTCATGTAGTTGCCTTTAATACTTTACTATTCCTTATGTCATTCACTGTACT 9483

MW494315.1 TGAATACAGTCATGTAGTTGCCTTTAATACTTTACTATTCCTTATGTCATTCACTGTACT 9540

KF686346.1 AGATTATACTAGTGTTGTAGTTATAAATGTTGTTGTTTGGTGTATTAATTTTCTTATGCT 9782

NC_006213.1 TGATTACACCAGTGTTGTTTTTGTTAACGTGATTGTGTGGTGTGTAAATTTTATGATGCT 9442

NC_002645.1 TGACCTTTCTGTAGGTGTTTGCACTGTTGTTGTGGCTGTTTTGCTTAACAATGTCTCTTA 8691

JX504050.1 TGATCTTTCTTATGGTGTTTTTACTGTTGTTTGTGCAACTTTGATTAATAACATTTCTTA 8607

** * *

NC_019843.3 CTGCTTTGTTACCTCTATACCATTGTGTATAGTACCTTACACTGCATTGTACTATTATGC 9565

NC_004718.3 CTGTCTGGTACCAGCTTACAGCTTTCTGCCGGGAGTCTACTCAGTCTTTTACTTGTACTT 9530

EPI_ISL_6640916_omicron CTGTTTAACACCAGTTTACTCATTCTTACCTGGTGTTTATTCTGTTATTTACTTGTACTT 9543

MW494315.1 CTGTTTAACACCAGTTTACTCATTCTTACCTGGTGTTTATTCTGTTATTTACTTGTACTT 9600

KF686346.1 TTTTGTTTTTCAAGTTTATCCTATTTGTGCATGTGTTTATGCTTGTTTTTATTTTTATGT 9842

NC_006213.1 TTTTGTGTTTCAAGTTTACCCCATACTTTCTTGTGTATATGCTATTTGTTATTTTTATGC 9502

NC_002645.1 CATTGTAACTCAGAATTTAG---TAACAATGATTGCTTATGCCATATTGTATTTCTTTGC 8748

JX504050.1 TGTTGTTACTCAAAATTTAT---TTTTTATGTTGCTTTATGCTATTTTGTATTTTGTTTT 8664

* * * ** * ** *

NC_019843.3 TACATTCTATTTTACTAATGAGCCTGCATTTATTATGCATGTTTCTTGGTACATTATGTT 9625

NC_004718.3 GACATTCTATTTCACCAATGATGTTTCATTCTTGGCTCACCTTCAATGGTTTGCCATGTT 9590

EPI_ISL_6640916_omicron GACATTTTATCTTACTAATGATGTTTCTTTTTTAGCACATATTCAGTGGATGGTTATGTT 9603

MW494315.1 GACATTTTATCTTACTAATGATGTTTCTTTTTTAGCACATATTCAGTGGATGGTTATGTT 9660

KF686346.1 AACATTGTATTTTCCTTCTGAAATTAGTGTAATTATGCATTTGCAATGGATTGTTATGTA 9902

NC_006213.1 CACGCTTTATTTCCCTTCGGAGATAAGTGTGATAATGCACTTACAATGGCTAGTTATGTA 9562

NC_002645.1 TACTAGAAGCTTACGCTATG------CATGGATTTGGTGTGCTGCATATTTAATTGCGTA 8802

JX504050.1 TACTAGGACAGTGCGTTATG------CTTGGATTTGGCATATTGCATACATTGTTGCATA 8718

** * * * * *

NC_019843.3 CGGGCCTATCGTTCCCATATGGATGACCTGCGTCTATACAGTTGCAATGTGCTTTAGACA 9685

NC_004718.3 TTCTCCTATTGTGCCTTTTTGGATAACAGCAATCTATGTATTCTGTATTTCTCTGAAGCA 9650

EPI_ISL_6640916_omicron CACACCTTTAGTACCTTTCTGGATAACAATTGCTTATATCATTTGTATTTCCACAAAGCA 9663

MW494315.1 CACACCTTTAGTACCTTTCTGGATAACAATTGCTTATATCATTTGTATTTCCACAAAGCA 9720

KF686346.1 TGGTGCTATAATGCCTTTTTGGTTTTGTGTCACATATGTAGCTATGGTTATTGCAAACCA 9962

NC_006213.1 TGGCACTATTATGCCTTTATGGTTTTGTTTGCTATATATAGCTGTTGTTGTTTCAAATCA 9622

NC_002645.1 TATTTCTTTTGCTCCATGGTGGTTGTGTGCTTGGTACTTTCTTGCTATGTTGACAGG--- 8859

JX504050.1 CTTCTTGTTAATACCATGGTGGCTTCTCACATGGTTTAGTTTTGCTGCATTTTTAGA--- 8775

* ** *** * *

NC_019843.3 CTTCTTCTGGGTTTTAGCTTATTTTAGTAAGAAACATGTAGAAGTTTTTACTGATGGTAA 9745

NC_004718.3 CTGCCATTGGTTCTTTAACAACTATCTTAGGAAAAGAGT---CATGTTTAATGGAGTTAC 9707

EPI_ISL_6640916_omicron TTTCTATTGGTTCTTTAGTAATTACCTAAAGAGACGTGT---AGTCTTTAATGGTGTTTC 9720

MW494315.1 TTTCTATTGGTTCTTTAGTAATTACCTAAAGAGACGTGT---AGTCTTTAATGGTGTTTC 9777

KF686346.1 TGTTTTATGGTTATTTTCATATTGTAGGA--AAATTGGTGTTAATGTATGTAG------- 10013

NC_006213.1 TGCTTTTTGGGTATTTTCTTACTGCAGAA--AGCTTGGTACTTCTGTTCGTAG------- 9673

NC_002645.1 -TTTGTTACCTAGTTTGCTGAAGCTTAAA--GTTTCGACAAATCTTTTCGAAGGTGACAA 8916

JX504050.1 -GCTTTTACCTAATGTTTTTAAGTTAAAA--ATCTCTACTCAATTGTTTGAAGGTGATAA 8832

* * * * * *

NC_019843.3 GCTTAATTGTAGTTTCCAGGACGCTGCCTCTAATATCTTTGTTATTAACAAGGACA---- 9801

NC_004718.3 ATTTA---GTACCTTCGAGGAGGCTGCTTTGTGTACCTTTTTGCTCAACAAGGAAATGTA 9764

EPI_ISL_6640916_omicron CTTTA---GTACTTTTGAAGAAGCTGCGCTGTGCACCTTTTTGTTAAATAAAGAAATGTA 9777

MW494315.1 CTTTA---GTACTTTTGAAGAAGCTGCGCTGTGCACCTTTTTGTTAAATAAAGAAATGTA 9834

KF686346.1 ---TGATAGTACATTTGAAGAAACATCTCTTACTACTTTTATGATTACTAAAGATT---- 10066

NC_006213.1 ---TGATGGTACATTTGAAGAAATGGCTCTCACTACTTTTATGATTACAAAAGATT---- 9726

NC_002645.1 ATTTGTAGGTACATTTGAAAGTGCTGCTGCAGGAACATTTGTCATTGACATGCGTT---- 8972

JX504050.1 GTTTATAGGTACTTTTGAGAGTGCTGCTGCAGGTACATTTGTTCTTGACATGCGTT---- 8888

* *** ** * * * *** * * *

NC_019843.3 -CTTATGCAGCTCTTAGAAACTCTTTAACTAATGATGC-CTATTCACGATTTTTGGGGTT 9859

NC_004718.3 CCTAAAATTGCGTAGCGAGACACTGTTGCCACTTACACAGTATAACAGGTATCTTGCTCT 9824

EPI_ISL_6640916_omicron TCTAAAGTTGCGTAGTGATGTGCTATTACCTCTTACGCAATATAATAGATACTTAGCTCT 9837

MW494315.1 TCTAAAGTTGCGTAGTGATGTGCTATTACCTCTTACGCAATATAATAGATACTTAGCTCT 9894

KF686346.1 -CTTATTGTAGATTAAAGAATTCTGTTTCTGATGTTGC-CTACAATAGATATTTGAGTTT 10124

NC_006213.1 -CTTATTGTAAGCTTAAGAATTCTTTGTCTGATGTTGC-TTTTAATAGATATTTGAGTTT 9784

NC_002645.1 -CTTATGAGAAACTTGCTAATAGCATCTCTCCAGAAAA-GTTGAAAAGTTATGCTGCTAG 9030

JX504050.1 -CTTATGAAAGGCTGATAAATACTATTTCACCTGAGAA-ACTTAAGAATTATGCTGCAAG 8946

** * * * *

NC_019843.3 GTTTAACAAGTATAAGTACTTCTCTGGTGCTATGGAAACAGCCGCTTATCGTGAAGCTGC 9919

NC_004718.3 ATATAACAAGTACAAGTATTTCAGTGGAGCCTTAGATACTACCAGCTATCGTGAAGCAGC 9884

EPI_ISL_6640916_omicron TTATAATAAGTACAAGTATTTTAGTGGAGCAATGGATACAACTAGCTACAGAGAAGCTGC 9897

MW494315.1 TTATAATAAGTACAAGTATTTTAGTGGAGCAATGGATACAACTAGCTACAGAGAAGCTGC 9954

KF686346.1 GTATAATAAGTATCGTTACTATAGTGGTAAAATGGATACTGCTGCCTATAGAGAAGCGGC 10184

NC_006213.1 GTATAATAAATATAGGTATTACAGCGGTAAAATGGATACTGCTGCATATAGGGAGGCTGC 9844

NC_002645.1 CTATAATAGATATAAGTACTATAGTGGTAATGCAAATGAAGCTGATTACCGTTGCGCTTG 9090

JX504050.1 TTATAATAAATATAAATATTATAGTGGTAGTGCTAGTGAGGCTGATTATCGTTGTGCTTG 9006

* *** * ** ** * ** * ** * **

NC_019843.3 AGCATGTCATCTTGCTAAAGCCTTACAAACATACAGCGAGACTGG---TAGTGATCTTCT 9976

NC_004718.3 TTGCTGCCACTTAGCAAAGGCTCTAAATGACTTTAGCAACTCAGG---TGCTGATGTTCT 9941

EPI_ISL_6640916_omicron TTGTTGTCATCTCGCAAAGGCTCTCAATGACTTCAGTAACTCAGG---TTCTGATGTTCT 9954

MW494315.1 TTGTTGTCATCTCGCAAAGGCTCTCAATGACTTCAGTAACTCAGG---TTCTGATGTTCT 10011

KF686346.1 GTGTTCTCAGTTAGCTAAAGCTATGGAAACATTTAATCACAATAATGGTAATGATGTCTT 10244

NC_006213.1 TTGCTCTCAGTTGGCTAAAGCAATGGACACATTTACCAATAATAATGGTAGTGATGTGCT 9904

NC_002645.1 TTATGCCTATTTAGCAAAAGCAATGTTGGACTTTTCGCGTGATCA---TAATGACATCTT 9147

JX504050.1 TTATGCTCATTTAGCCAAGGCTATGTTAGATTATGCAAAAGATCA---TAATGACATGTT 9063

* * ** ** ** * * * *** * *

NC_019843.3 TTACCAACCACCCAACTGTAGCATAACCTCTGGCGTGTTGCAAAGCGGTTTGGTGAAAAT 10036

NC_004718.3 CTACCAACCACCACAGACATCAATCACTTCTGCTGTTCTGCAGAGTGGTTTTAGGAAAAT 10001

EPI_ISL_6640916_omicron TTACCAACCACCACAAATCTCTATCACCTCAGCTGTTTTGCAGAGTGGTTTTAGAAAAAT 10014

MW494315.1 TTACCAACCACCACAAACCTCTATCACCTCAGCTGTTTTGCAGAGTGGTTTTAGAAAAAT 10071

KF686346.1 ATACCAACCTCCTACAGCATCTGTTTCTACATCTTTTTTGCAATCAGGTATTGTAAAGAT 10304

NC_006213.1 TTACCAACCGCCTACTGCTTCCGTCTCAACTTCATTCTTGCAATCTGGTATTGTGAAAAT 9964

NC_002645.1 GTACACACCTCCGACTG---TCAGTTATGGTTCTACATTACAGGCTGGTTTGCGCAAAAT 9204

JX504050.1 ATATTCTCCACCTACTA---TTAGCTACAATTCCACCTTACAATCTGGTCTTAAGAAGAT 9120

** ** ** * ** *** * ** **

NC_019843.3 GTCACATCCCAGTGGAGATGTTGAGGCTTGTATGGTTCAGGTTACCTGCGGTAGCATGAC 10096

NC_004718.3 GGCATTCCCGTCAGGCAAAGTTGAAGGGTGCATGGTACAAGTAACCTGTGGAACTACAAC 10061

EPI_ISL_6640916_omicron GGCATTCCCATCTGGTAAAGTTGAGGGTTGTATGGTACAAGTAACTTGTGGTACAACTAC 10074

MW494315.1 GGCATTCCCATCTGGTAAAGTTGAGGGTTGTATGGTACAAGTAACTTGTGGTACAACTAC 10131

KF686346.1 GGTATCTCCTACGTCAAAAATTGAACCTTGTATTGTTAGTGTTACTTATGGTAGTATGAC 10364

NC_006213.1 GGTAAATCCTACTTCTAAGGTAGAACCATGTGTTGTCAGTGTTACCTATGGTAATATGAC 10024

NC_002645.1 GGCACAACCATCTGGCTTTGTGGAGAAATGTGTTGTCCGTGTCTGCTATGGAAACACTGT 9264

JX504050.1 GGCACAACCATCTGGTTGTGTTGAGAGATGTGTGGTTCGCGTCTGTTATGGTAGTACTGT 9180

* * ** * ** ** * ** ** * ** * *

NC_019843.3 TCTTAATGGTCTTTGGCTTGACAACACAGTCTGGTGCCCACGACACGTAATGTGCCCGGC 10156

NC_004718.3 TCTTAATGGATTGTGGTTGGATGACACAGTATACTGTCCAAGACATGTCATTTGCACAGC 10121

EPI_ISL_6640916_omicron ACTTAACGGTCTTTGGCTTGATGACGTAGTTTACTGTCCAAGACATGTGATCTGCACCTC 10134

MW494315.1 ACTTAACGGTCTTTGGCTTGATGACGTAGTTTACTGTCCAAGACATGTGATCTGCACCTC 10191

KF686346.1 TTTGAATGGTTTATGGTTAGATGACAAAGTTTATTGTCCTCGTCATGTTATATGTTCATC 10424

NC_006213.1 ATTGAATGGTTTATGGTTGGATGACAAGGTCTACTGTCCCAGACATGTAATATGTTCTGC 10084

NC_002645.1 GTTGAATGGGTTGTGGCTTGGTGATATTGTTTATTGCCCACGTCATGTTATCGCATCTAA 9324

JX504050.1 GCTTAATGGAGTTTGGTTAGGTGACACTGTTACTTGTCCTAGACATGTCATAGCACCATC 9240

* ** ** * *** * * * ** ** ** * ** ** ** *

NC_019843.3 TGACCAGTTGTCTGATCCTAATTATGATGCCTTGTTGATTTCTATGACTAATCATAGTTT 10216

NC_004718.3 AGAAGACATGCTTAATCCTAACTATGAAGATCTGCTCATTCGCAAATCCAACCATAGCTT 10181

EPI_ISL_6640916_omicron TGAAGACATGCTTAACCCTAATTATGAAGATTTACTCATTCGTAAGTCTAATCATAATTT 10194

MW494315.1 TGAAGACATGCTTAACCCTAATTATGAAGATTTACTCATTCGTAAGTCTAATCATAATTT 10251

KF686346.1 CTCTAATATGAACGAACCTGATTATTCTGCCTTATTGTGTAGAGTTACTCTAGGTGATTT 10484

NC_006213.1 TTCAGATATGACTAATCCAGATTATACAAATTTGTTGTGTAGAGTAACATCAAGTGATTT 10144

NC_002645.1 CACAACTTCTGCTA---TAGATTATGATCACGAATATAGTATTATGCGGTTGCATAATTT 9381

JX504050.1 AACCACTGTTCTTA---TTGATTATGATCATGCATATAGTACTATGCGTTTGCATAATTT 9297

* *** * * **

NC_019843.3 CAGTGTGCAAAAACACATTGGCGCTCCAGCAAACTTGCGTGTTGTTGGTCATGCCATGCA 10276

NC_004718.3 TCTTGTTCAG---------GCTGGCAATGTTCAACTTCGTGTTATTGGCCATTCTATGCA 10232

EPI_ISL_6640916_omicron CTTGGTACAG---------GCTGGTAATGTTCAACTCAGGGTTATTGGACATTCTATGCA 10245

MW494315.1 CTTGGTACAG---------GCTGGTAATGTTCAACTCAGGGTTATTGGACATTCTATGCA 10302

KF686346.1 TACTATAATG---------TCTGGTCGGATGAGTTTAACAGTTGTGTCTTACCAGATGCA 10535

NC_006213.1 TACTGTATTG---------TTTGATCGTCTAAGCCTTACAGTGATGTCTTATCAAATGCG 10195

NC_002645.1 TTCTATAATA---------TCTGGTACAGCATTTCTTGGTGTTGTAGGTGCTACTATGCA 9432

JX504050.1 TTCAGTGTCT---------CATAATGGTGTCTTCTTGGGAGTTGTCGGTGTTACAATGCA 9348

* * ** * ****

NC_019843.3 AGGCACTCTTTTGAAGTTGACTGTCGATGTTGCTAACCCTAGCACTCCAGCCTACACTTT 10336

NC_004718.3 AAATTGTCTGCTTAGGCTTAAAGTTGATACTTCTAACCCTAAGACACCCAAGTATAAATT 10292

EPI_ISL_6640916_omicron AAATTGTGTACTTAAGCTTAAGGTTGATACAGCCAATCCTAAGACACCTAAGTATAAGTT 10305

MW494315.1 AAATTGTGTACTTAAGCTTAAGGTTGATACAGCCAATCCTAAGACACCTAAGTATAAGTT 10362

KF686346.1 GGGCTGTCAACTTGTTTTGACAGTCTCTTTACAAAATCCTTACACTCCAAAATATACTTT 10595

NC_006213.1 GGGTTGTATGCTTGTTCTTACAGTGACCCTGCAAAATTCTCGTACGCCAAAATATACATT 10255

NC_002645.1 TGGAGTAACTCTTAAAATTAAGGTTTCACAGACTAACATGCACACACCTAGACATTCTTT 9492

JX504050.1 TGGTTCTGTGTTGCGTATTAAGGTTTCACAATCTAATGTACATACACCTAAACATGTTTT 9408

* * * ** ** ** ** * **

NC_019843.3 TACAACAGTGAAACCTGGCGCAGCATTTAGTGTGTTAGCATGCTATAATGGTCGTCCGAC 10396

NC_004718.3 TGTCCGTATCCAACCTGGTCAAACATTTTCAGTTCTAGCATGCTACAATGGTTCACCATC 10352

EPI_ISL_6640916_omicron TGTTCGCATTCAACCAGGACAGACTTTTTCAGTGTTAGCTTGTTACAATGGTTCACCATC 10365

MW494315.1 TGTTCGCATTCAACCAGGACAGACTTTTTCAGTGTTAGCTTGTTACAATGGTTCACCATC 10422

KF686346.1 TGGTAATGTTAAACCTGGTGAAACTTTTACTGTTTTAGCTGCGTATAATGGCCGACCACA 10655

NC_006213.1 TGGTGTGGTTAAACCTGGTGAGACTTTTACTGTTTTAGCTGCTTATAACGGCAAACCACA 10315

NC_002645.1 TAGAACACTAAAATCTGGTGAAGGTTTTAACATCTTAGCATGCTATGATGGTTGTGCTCA 9552

JX504050.1 TAAAACGTTGAAACCTGGTGATTCTTTTAATATTTTAGCATGTTATGAAGGTATTGCATC 9468

* * ** * ** *** * **** ** * ** *

NC_019843.3 TGGTACATTCACTGTTGTAATGCGCCCTAACTACACAATTAAGGGTTCCTTTCTGTGTGG 10456

NC_004718.3 TGGTGTTTATCAGTGTGCCATGAGACCTAATCATACCATTAAAGGTTCTTTCCTTAATGG 10412

EPI_ISL_6640916_omicron TGGTGTTTACCAATGTGCTATGAGGCACAATTTCACTATTAAGGGTTCATTCCTTAATGG 10425

MW494315.1 TGGTGTTTACCAATGTGCTATGAGGCCCAATTTCACTATTAAGGGTTCATTCCTTAATGG 10482

KF686346.1 AGGGGCATTTCATGTTACTATGCGTAGTAGTTATACTATTAAAGGTTCTTTTTTGTGTGG 10715

NC_006213.1 AGGAGCCTTTCATGTAACTATGCGTAGTAGTTATACCATTAAGGGTTCCTTTTTATGCGG 10375

NC_002645.1 AGGTGTTTTTGGTGTGAACATGAGAACTAATTGGACTATCCGTGGTTCATTTATTAATGG 9612

JX504050.1 TGGTGTTTTTGGTGTTAATTTACGTACAAACTTTACTATTAAAGGTTCTTTTATAAATGG 9528

** * * * * ** ** ***** ** * **

NC_019843.3 TTCTTGTGGTAGTGTTGGTTACACCAAGGAGG---GTAGTGTGATCAATTTCTGTTACAT 10513

NC_004718.3 ATCATGTGGTAGTGTTGGTTTTAACATTGATT---ATGATTGCGTGTCTTTCTGCTATAT 10469

EPI_ISL_6640916_omicron TTCATGTGGTAGTGTTGGTTTTAACATAGATT---ATGACTGTGTCTCTTTTTGTTACAT 10482

MW494315.1 TTCATGTGGTAGTGTTGGTTTTAACATAGATT---ATGACTGTGTCTCTTTTTGTTACAT 10539

KF686346.1 GTCATGTGGATCTGTTGGTTATGTATTAACAG---GTGATAGTGTTAAGTTTGTATATAT 10772

NC_006213.1 ATCTTGTGGATCTGTTGGTTATGTAATAATGG---GTGATTGTGTTAAATTTGTTTATAT 10432

NC_002645.1 TGCGTGTGGTTCCCCTGGCTACAATCTTAAAA---ATGGCGAGGTGGAATTTGTTTATAT 9669

JX504050.1 AGCTTGTGGTTCTCCTGGTTATAATGTTAGAAATGATGGTACTGTTGAGTTTTGTTATTT 9588

* ***** *** * * * ** ** *

NC_019843.3 GCATCAAATGGAACTTGCTAATGGTACACATACCGGTTCAGCATTTGATGGTACTATGTA 10573

NC_004718.3 GCATCATATGGAGCTTCCAACAGGAGTACACGCTGGTACTGACTTAGAAGGTAAATTCTA 10529

EPI_ISL_6640916_omicron GCACCATATGGAATTACCAACTGGAGTTCATGCTGGCACAGACTTAGAAGGTAACTTTTA 10542

MW494315.1 GCACCATATGGAATTACCAACTGGAGTTCATGCTGGCACAGACTTAGAAGGTAACTTTTA 10599

KF686346.1 GCATCAATTAGAGCTCAGTACTGGTTGTCACACTGGCACTGATTTTACTGGTAATTTTTA 10832

NC_006213.1 GCATCAATTGGAGCTTAGTACTGGTTGTCATACTGGTACTGACTTCAATGGGGATTTTTA 10492

NC_002645.1 GCATCAAATTGAACTCGGAAGTGGTAGCCATGTAGGTTCTAGCTTTGATGGTGTTATGTA 9729

JX504050.1 ACACCAAATTGAGTTAGGTAGTGGTGCTCATGTTGGTTCTGATTTTACTGGTAGTGTTTA 9648

** ** * ** * * ** ** ** * ** ** * **

NC_019843.3 TGGTGCCTTTATGGATAAACAAGTGCACCAAGTTCAGTTAACAGACAAATACTGCAGTGT 10633

NC_004718.3 TGGTCCATTTGTTGACAGACAAACTGCACAGGCTGCAGGTACAGACACAACCATAACATT 10589

EPI_ISL_6640916_omicron TGGACCTTTTGTTGACAGGCAAACAGCACAAGCAGCTGGTACGGACACAACTATTACAGT 10602

MW494315.1 TGGACCTTTTGTTGACAGGCAAACAGCACAAGCAGCTGGTACGGACACAACTATTACAGT 10659

KF686346.1 TGGTCCATATAGAGATGCTCAAGTTGTACAGTTGCCAGTTAAGGACTATGTCCAGACTGT 10892

NC_006213.1 TGGTCCTTATAAGGATGCTCAGGTTGTTCAGTTGCTCATTCAGGATTATATACAATCTGT 10552

NC_002645.1 TGGTGGTTTTGAAGACCAACCTAATCTTCAAGTTGAATCTGCAAACCAGATGTTAACAGT 9789

JX504050.1 TGGTAATTTTGATGACCAACCTAGTTTGCAAGTTGAGAGTGCCAACCTTATGCTATCAGA 9708

*** * * ** * ** *

NC_019843.3 TAATGTAGTAGCTTGGCTTTACGCAGCAATACTTAATGGTTGCGCTTGGTTTGTAAAACC 10693

NC_004718.3 AAATGTTTTGGCATGGCTGTATGCTGCTGTTATCAATGGTGATAGGTGGTTTCTTAATAG 10649

EPI_ISL_6640916_omicron TAATGTTTTAGCTTGGTTGTACGCTGCTGTTATAAATGGAGACAGGTGGTTTCTCAATCG 10662

MW494315.1 TAATGTTTTAGCTTGGTTGTACGCTGCTGTTATAAATGGAGACAGGTGGTTTCTCAATCG 10719

KF686346.1 TAATGTTATTGCTTGGCTCTATGCAGCTATACTTAATAATTGTGCTTGGTTTGTACAAAA 10952

NC_006213.1 TAATTTTGTAGCATGGCTTTATGCTGCTATACTTAACAATTGTAATTGGTTTGTACAAAG 10612

NC_002645.1 TAATGTGGTTGCATTTCTTTATGCTGCTATATTGAATGGTTGCACATGGTGGCTTAAAGG 9849

JX504050.1 TAATGTTGTTGCCTTTTTGTATGCTGCTTTGTTGAATGGTTGTAGGTGGTGGTTGTGTTC 9768

*** * * ** * * ** ** ** * * ** **** *

NC_019843.3 TAATCGCACTAGTGTTGTTTCTTTTAATGAATGGGCTCTTGCCAACCAATTCACTGAATT 10753

NC_004718.3 ATTCACCACTACTTTGAATGACTTTAACCTTGTGGCAATGAAGTACAACT---ATGAACC 10706

EPI_ISL_6640916_omicron ATTTACCACAACTCTTAATGACTTTAACCTTGTGGCTATGAAGTACAATT---ATGAACC 10719

MW494315.1 ATTTACCACAACTCTTAATGACTTTAACCTTGTGGCTATGAAGTACAATT---ATGAACC 10776

KF686346.1 TGATGTTTGTTCTACTGAAGATTTTAATGTTTGGGCTATGGCAAATGGTTTTAGCCAAGT 11012

NC_006213.1 TGATAAGTGTTCTGTAGAAGATTTTAATGTGTGGGCTCTGTCCAATGGATTTAGCCAAGT 10672

NC_002645.1 TGAAAAATTGTTTGTGGAGCATTATAATGAGTGGGCACAGGCTAATGGTTTCACAGCTAT 9909

JX504050.1 AACTAGAGTTAATGTTGATGGTTTTAATGAATGGGCTATGGCTAATGGTTATACAAGTGT 9828

* * *** *** * *

NC_019843.3 TGTTGGCACTCAATCCGTTGACATGTTAG---------CTGTCAAAACAGGCGTTGCTAT 10804

NC_004718.3 TTTGACACAAGATCATGTTGACATATTGGGACCTCTTTCTGCTCAAACAGGAATTGCCGT 10766

EPI_ISL_6640916_omicron TCTAACACAAGACCATGTTGACATACTAGGACCTCTTTCTGCTCAAACTGGAATTGCCGT 10779

MW494315.1 TCTAACACAAGACCATGTTGACATACTAGGACCTCTTTCTGCTCAAACTGGAATTGCCGT 10836

KF686346.1 AAAAGCAGATCTTGTTTTAGATGCTTTGG---------CTTCAATGACAGGTGTTTCTAT 11063

NC_006213.1 TAAATCTGACCTTGTTATAGATGCTTTAG---------CTTCTATGACTGGTGTGTCTTT 10723

NC_002645.1 GAATGGTGAAGACGCTTTTTCCATTCTTG---------CTGCTAAAACTGGTGTCTGTGT 9960

JX504050.1 TTCTAGTGTTGAGTGCTATTCTATTTTGG---------CAGCAAAAACTGGTGTTAGTGT 9879

* * * ** ** * *

NC_019843.3 TGAACAGCTGCTTTATGCGATCCAACA---ACTGTATACTGGGTTCCAGGGAAAGCAAAT 10861

NC_004718.3 CTTAGATATGTGTGCTGCTTTGAAAGAGCTGCTGCAGAATGGTATGAATGGTCGTACTAT 10826

EPI_ISL_6640916_omicron TTTAGATATGTGTGCTTCATTAAAAGAATTACTGCAAAATGGTATGAATGGACGTACCAT 10839

MW494315.1 TTTAGATATGTGTGCTTCATTAAAAGAATTACTGCAAAATGGTATGAATGGACGTACCAT 10896

KF686346.1 TGAAACCTTATTGGCTGCTATTAAGCG---TCTATATATGGGATTTCAAGGTCGTCAAAT 11120

NC_006213.1 GGAAACACTGTTGGCTGCTATTAAGCG---TCTTAAGAATGGTTTCCAAGGACGTCAGAT 10780

NC_002645.1 GGAAAGATTACTTCATGCTATTCAAGT---TTTGAATAATGGCTTTGGTGGTAAACAAAT 10017

JX504050.1 TGAACAATTGTTAGCTTCCATTCAACA---TCTTCATGAAGGTTTTGGTGGTAAAAACAT 9936

* * * * * * * * ** * ** **

NC_019843.3 CCTTGGCAGTACCATGTTGGAAGATGAATTCACACCTGAGGATGTTAATATGCAGATTAT 10921

NC_004718.3 CCTTGGTAGCACTATTTTAGAAGATGAGTTTACACCATTTGATGTTGTTAGACAATGCTC 10886

EPI_ISL_6640916_omicron ATTGGGTAGTGCTTTATTAGAAGATGAATTTACACCTTTTGATGTTGTTAGACAATGCTC 10899

MW494315.1 ATTGGGTAGTGCTTTATTAGAAGATGAATTTACACCTTTTGATGTTGTTAGACAATGCTC 10956

KF686346.1 ACTAGGAAGTTGTACTTTTGAAGATGAATTGGCACCTTCTGACGTTTATCAACAATTGGC 11180

NC_006213.1 TATGGGTAGTTGCTCTTTTGAGGATGAATTGACACCTAGCGATGTTTATCAACAACTCGC 10840

NC_002645.1 TTTGGGTTATTCTAGTCTCAATGATGAGTTCAGTATTAATGAAGTTGTCAAACAAATGTT 10077

JX504050.1 ACTTGGTTATTCTAGTTTATGTGATGAGTTCACACTAGCTGAAGTTGTGAAGCAGATGTA 9996

* ** * ***** ** ** *** **

NC_019843.3 GGGTGTGGTTATGCAGAGTGGTGTGAGAAAAGTTACATATGGTACTGCGCATTGGTTGTT 10981

NC_004718.3 TGGTGTTACCTTCCAAGGTAAGTTCAAGAAAATTGTTAAGGGCACTCATCATTGGATGCT 10946

EPI_ISL_6640916_omicron AGGTGTTACTTTCCAAAGTGCAGTGAAAAGAACAATCAAGGGTACACACCACTGGTTGTT 10959

MW494315.1 AGGTGTTACTTTCCAAAGTGCAGTGAAAAGAACAATCAAGGGTACACACCACTGGTTGTT 11016

KF686346.1 TGGTGTTAAATTGCAATCTAAAACAAAAAGATTTATTAAAGAAACAATTTATTGGATTTT 11240

NC_006213.1 TGGTATCAAGTTACAATCAAAACGCACTAGATTGTTTAAAGGCACTGTTTGTTGGATTAT 10900

NC_002645.1 TGGTGTTAACCTGCAAAGTGGTAAAACCACTAGTATGTTTAAATCCATAAGCT------- 10130

JX504050.1 TGGTGTTAACTTGCAAAGTGGTAAGGTTATTTTTGGTTTAAAAACAATGTTTT------- 10049

*** * * ** * * *

NC_019843.3 TGCGACCCTTGTCTCAACCTATGTGATAATCTTACAAGCCACTAAATTTACTTTGTGGAA 11041

NC_004718.3 TTTAACTTTCTTGACATCACTATTGATTCTTGTTCAAAGTACACAGTGGTCACTGTTTTT 11006

EPI_ISL_6640916_omicron ACTCACAATTTTGACTTCACTTTTAGTTTTAGTCCAGAGTACTCAATGGTCTTTGTTNNT 11019

MW494315.1 ACTCACAATTTTGACTTCACTTTTAGTTTTAGTCCAGAGTACTCAATGGTCTTTGTTCTT 11076

KF686346.1 GATATCTACATTTTTGTTTAGTTGTATAATTTCTGCATTTGTTAAATGGACTATATTTAT 11300

NC_006213.1 GGCTTCTACATTTTTGTTTAGTTGCATAATTACAGCATTTGTGAAATGGACTATGTTTAT 10960

NC_002645.1 --TATTTGCTGGCTTCTTTGTCATGTTCTGGGCTGAATTATTTGTTTATACCACCACTAT 10188

JX504050.1 --TATTTAGCGTTTTCTTCACAATGTTTTGGGCAGAACTCTTTATTTATACAAACACTAT 10107

* * *

NC_019843.3 CTACTTGTTTGAGACTATTCCCACACAGTTGTTCCCACTCTTATTTGTGACTATGGCCTT 11101

NC_004718.3 CTTTGTTTACGAGAATGCTTTCTTGCCATTTACTCTTGGTATTATGGCAATTGCTGCATG 11066

EPI_ISL_6640916_omicron TTTTTTGTATGAAAATGCCTTTTTACCTTTTGCTATGGGTATTATTGCTATGTCTGCTTT 11079

MW494315.1 TTTTTTGTATGAAAATGCCTTTTTACCTTTTGCTATGGGTATTATTGCTATGTCTGCTTT 11136

KF686346.1 GTATATTAATACACATATGATTGGTGTTACATTATGTGTACTTTGTTTTGT---TAGTTT 11357

NC_006213.1 GTATGTAACTACTAATATGTTTAGTATTACGTTTTGTGCACTTTGTGTTAT---AAGTTT 11017

NC_002645.1 TTGGGTTAACCCTGGTTTTCTTACTCCGTTTATGATTTTGCTTGTTGCTTTGTCACTCTG 10248

JX504050.1 ATGGATAAACCCTGTGATACTTACACCTATATTTTGTCTACTTTTGTTTTTGTCATTAGT 10167

* * *

NC_019843.3 CGTTATGTTGTTGGTTAAACACAAACACACCTTTTTGACACTTTTCTTGTTGCCTGTGGC 11161

NC_004718.3 TGCTATGCTGCTTGTTAAGCATAAGCACGCATTCTTGTGCTTGTTTCTGTTACCTTCTCT 11126

EPI_ISL_6640916_omicron TGCAATGATGTTTGTCAAACATAAGCATGCATTTCTCTGTTTGTTTTTGTTACCTTCTCT 11139

MW494315.1 TGCAATGATGTTTGTCAAACATAAGCATGCATTTCTCTGTTTGTTTTTGTTACCTTCTCT 11196

KF686346.1 TATGATGTTACTAGTTAAACATAAGCATTTTTATTTGACTATGTATATAATTCCTGTACT 11417

NC_006213.1 GGCCATGTTGTTGGTTAAGCATAAGCATCTTTATTTGACTATGTATATAACTCCTGTGCT 11077

NC_002645.1 TCTTACATTTGTTGTTAAACATAAGGTTTTGTTTTTGCAAGTGTTTTTGTTGCCTTCAAT 10308

JX504050.1 TTTAACTATGTTTCTTAAACATAAGTTTTTGTTTTTGCAAGTATTTTTATTACCTACTGT 10227

* * * * ** ** ** * * * * * ***

NC_019843.3 TATTTGTTTGACTTATGCAAAC------ATAGTCTACGAGCCCACT-------------- 11201

NC_004718.3 TGCAACAGTTGCTTACTTTAAT------ATGGTCTACATGCCTGCT-------------- 11166

EPI_ISL_6640916_omicron TGCCACTGTAGCTTATTTTAAT------ATGGTCTATATGCCTGCT-------------- 11179

MW494315.1 TGCCACTGTAGCTTATTTTAAT------ATGGTCTATATGCCTGCT-------------- 11236

KF686346.1 CTGTACCTTGTTTTATGTAAATTATTTAGTTGTTTATAAGGAAGGTTTTAGAGGTTTTAC 11477

NC_006213.1 TTTTACACTGTTGTATAACAACTATTTGGTTGTGTACAAGCATACATTTAGAGGCTATGT 11137

NC_002645.1 TATTGTGGCTGCTATTCAAAACTGTGCTTGGGACTACCATGTTACA---------AAGGT 10359

JX504050.1 TATTGCAACTGCTTTATATAATTGTGTTTTGGATTATTACATAGTA---------AAATT 10278

** * **

NC_019843.3 -----------------------------------ACT---------CCCATTTCGTCAG 11217

NC_004718.3 -----------------------------------AGCTGGGTGATGCGTATCATGACAT 11191

EPI_ISL_6640916_omicron -----------------------------------AGTTGGGTGATGCGTATTATGACAT 11204

MW494315.1 -----------------------------------AGTTGGGTGATGCGTATTATGACAT 11261

KF686346.1 TTATGTCTGGCTCTCATATTTTGTTCCTGCTGTGAATTTTACTTATGTTTATGAAGTATT 11537

NC_006213.1 CTATGCATGGCTATCATATTATGTTCCATCAGTTGAGTACACTTATACTGATGAAGTTAT 11197

NC_002645.1 GTTGGCAGAGAAGTTTGATTATAATGTTTCTGTTATGCAAATGGACATCCAGGGTTTTGT 10419

JX504050.1 TTTGGCTGACCATTTTAACTATAATGTTTCAGTATTACAAATGGATGTTCAGGGTTTAGT 10338

*

NC_019843.3 CGCTGATTGCAGTTGCAAATTGGCTTGCCCCCACTAATGC------------TTATATGC 11265

NC_004718.3 GGCTTGAATTGGCTG-ACACTAGCTTGTCTGGTTATAGGCTTAAGGATTGTGTTATGTAT 11250

EPI_ISL_6640916_omicron GGTTGGATATGGTTG-ATACTA---------GTTTTAAGCTAAAAGACTGTGTTATGTAT 11254

MW494315.1 GGTTGGATATGGTTG-ATACTAGTTTGTCTGGTTTTAAGCTAAAAGACTGTGTTATGTAT 11320

KF686346.1 TTATGGTTGTATTTT-ATGTGTTTTTGCTATTTTTATAACTATGCA---TAGTATTAATC 11593

NC_006213.1 TTATGGCATGTTATT-GCTTGTAGGAATGGTCTTTGTTACATTACG---TAGCATTAACC 11253

NC_002645.1 TAACATTTTTATTTG-TCTTTTTGTTGCACTGTTGCATACTTGGCGCTTTGCTAAAGAGC 10478

JX504050.1 TAATGTTTTGGTCTG-TTTATTTGTTGTATTTTTACACACATGGCGCTTTTCTAAAGAAC 10397

* *

NC_019843.3 GCACTACACATACTGATATTGGTGTCTACATTAGTATGTCACTTGTATTAGTCATTGTAG 11325

NC_004718.3 GCTTCAGCTTTAGTTTTGCTTATTCTCATGACAGCTCGCACTGTTTATGATGATGCTGCT 11310

EPI_ISL_6640916_omicron GCATCAGCTGTAGTGTTACTAATCCTTATGACAGCAAGAACTGTGTATGATGATGGTGCT 11314

MW494315.1 GCATCAGCTGTAGTGTTACTAATCCTTATGACAGCAAGAACTGTGTATGATGATGGTGCT 11380

KF686346.1 ATGACATTTTTTCTTTGATGTTTTTGGTTGGTAGAATAGTTACTTTAATTTCTATGTGGT 11653

NC_006213.1 ATGATTTGTTTTCTTTTATAATGTTTGTTGGTCGTTTGATTTCTGTTTTCTCTTTGTGGT 11313

NC_002645.1 GTTGTACACATTGGTGCACTTATTTGTTCTCACTCATTGCTGTTTTATACACTGCATTGT 10538

JX504050.1 GTTTTACACATTGGTTTACATATGTGTGTTCTCTTATAGCAGTTGCTTACACTTATTTTT 10457

* *

NC_019843.3 TGAAGAGATTGTACAACCCATCACTTTCTAACTTTGCGTTAGCATTGTGCAGTGGTGTAA 11385

NC_004718.3 AGACGTGTTTGGAC-ACTGATGAATGTCATTACACTTGTTTACAAAGTCTACTATGGTAA 11369

EPI_ISL_6640916_omicron AGGAGAGTGTGGAC-ACTTATGAATGTCTTGACACTCGTTTATAAAGTTTATTATGGTAA 11373

MW494315.1 AGGAGAGTGTGGAC-ACTTATGAATGTCTTGACACTCGTTTATAAAGTTTATTATGGTAA 11439

KF686346.1 ATTTTGGTTCGAAT---------------------------------------------- 11667

NC_006213.1 ACAAGGGTTCTAAC---------------------------------------------- 11327

NC_002645.1 ATAGTTATGACTAC---------------------------------------------- 10552

JX504050.1 ATAGTGGTGACTTT---------------------------------------------- 10471

NC_019843.3 TGTGGTTGTACACTTATAGCATTGGAGAAGCCTCAAGCCCCATTGCCTATCTGGTTTTTG 11445

NC_004718.3 TGCTTTAGATCA-------------------------AGCTATTTCCATGTGGGCCTTAG 11404

EPI_ISL_6640916_omicron TGCTTTAGATCA-------------------------AGCCATTTCCATGTGGGCTCTTA 11408

MW494315.1 TGCTTTAGATCA-------------------------AGCCATTTCCATGTGGGCTCTTA 11474

KF686346.1 -----------------------------------------TTAGAAGAGGATGTTTTGT 11686

NC_006213.1 -----------------------------------------TTAGAGGAAGAAATTCTTC 11346

NC_002645.1 -----------------------------------------GTTAGTTTGCTGGTTATGC 10571

JX504050.1 -----------------------------------------TTGAGTTTGCTTGTTATGT 10490

* *

NC_019843.3 TCACTACACTCACTAGTGATTATACGATTACAGTCTTTGTTACTGTCAACCTTGCAAAAG 11505

NC_004718.3 TTATTTCTGTAACCTCTAACTATTCTGGTGTCGTTACGACTATCATGTTTTTAGCTAGAG 11464

EPI_ISL_6640916_omicron TAATCTCTGTTACTTCTAACTACTCAGGTGTAGTTACAACTGTCATGTTTTTGGCCAGAG 11468

MW494315.1 TAATCTCTGTTACTTCTAACTACTCAGGTGTAGTTACAACTGTCATGTTTTTGGCCAGAG 11534

KF686346.1 TATTTATTACAGCCTTTTTAGGTACTTATACATGGACCACTATTTTGTCATTAGCTATAG 11746

NC_006213.1 TTATGTTGGCTTCCCTTTTTGGTACTTACACATGGACAACAGTTTTATCTATGGCTGTAG 11406

NC_002645.1 TACTTTGTGCAATTTCTAATGAATGGTATATTGGTGCTATTATTTTTAGAATT------T 10625

JX504050.1 TTTTATGTGCTATATCTAGTGATTGGTACATTGGTGCCATTGTTTTTAGGTTG------T 10544

* * * *

NC_019843.3 TTTGCACTTATGC---CATCTTTGCTTACTCACCACAGCTTACACTTGTGTTTCCGGAAG 11562

NC_004718.3 CTATAGTGTTTGT---GTGTGTTGAGTATTACCCATTGTTATTTATTACTGGCAACACCT 11521

EPI_ISL_6640916_omicron GTGTTGTTTTTAT---GTGTGTTGAGTATTGCCCTATTTTCTTCATAACTGGTAATACAC 11525

MW494315.1 GTATTGTTTTTAT---GTGTGTTGAGTATTGCCCTATTTTCTTCATAACTGGTAATACAC 11591

KF686346.1 CAAAAATTGTTGCTAATTGGTTGTCTGTTAATATATTTTATTTTACAGATGTACCTTATA 11806

NC_006213.1 CAAAGGTTATTGCTAAGTGGGTTGCTGTGAATGTCTTGTATTTCACAGATATACCTCAAA 11466

NC_002645.1 GTCGTTTTGGTGTTGCATTTTTACCAGTGGAATACGTGTCTTACTTTGATG------GTG 10679

JX504050.1 CACGTTTGATTGTATTTTTTTCACCTGAAAGTGTATTTAGTGTTTTTGGTG------ATG 10598

*

NC_019843.3 TGAAGATGATACTTTTATTATACACATGTTTAGGTTTCATGTGTACTTGCTATTTTGGTG 11622

NC_004718.3 TACAGTGTATCATGCTTGTTTATTGTTTCTTAGGCTATTGTTGCTGCTGCTACTTTGGCC 11581

EPI_ISL_6640916_omicron TTCAGTGTATAATGCTAGTTTATTGTTTCTTAGGCTATTTTTGTACTTGTTACTTTGGCC 11585

MW494315.1 TTCAGTGTATAATGCTAGTTTATTGTTTCTTAGGCTATTTTTGTACTTGTTACTTTGGCC 11651

KF686346.1 TTAAATTGATTCTCTTGAGTTACTTATTTATAGGGTATATTTTATCTTGTTATTGGGGAT 11866

NC_006213.1 TTAAGATAGTGCTTTTGTGCTATTTGTTTATTGGTTATATTATTAGCTGTTATTGGGGCT 11526

NC_002645.1 TTAAAACTGTGCTGTTGTTTTACATGTTGTTAGGCTTTGTTAGCTGTATGTACTATGGTT 10739

JX504050.1 TGAAACTTACTTTAGTTGTTTATTTAATTTGTGGTTATTTAGTTTGTACTTATTGGGGCA 10658

* * * * ** ** * ** * **

NC_019843.3 TCTTCTCTCTTTTGAACCTTAAGCTTAGAGCACCTATGGGTGTCTATGACTTTAAGGTCT 11682

NC_004718.3 TTTTCTGTTTACTCAACCGTTACTTCAGGCTTACTCTTGGTGTTTATGACTACTTGGTCT 11641

EPI_ISL_6640916_omicron TCTTTTGTTTACTCAACCGCTACTTTAGACTGACTCTTGGTGTTTATGATTACTTAGTTT 11645

MW494315.1 TCTTTTGTTTACTCAACCGCTACTTTAGACTGACTCTTGGTGTTTATGATTACTTAGTTT 11711

KF686346.1 TTTTCTCTCTTTTAAACAGTGTTTTTAGAATGCCTATGGGTGTTTATAATTATAAAATTT 11926

NC_006213.1 TGTTTTCCTTGATGAACAGTTTGTTTAGAATGCCTTTGGGTGTTTATAATTATAAAATTT 11586

NC_002645.1 TGTTGTACTGGATTAACAGGTTCTGTAAGTGCACATTAGGTGTTTATGATTTCTGTGTTA 10799

JX504050.1 TTTTGTATTGGTTCAATAGGTTTTTTAAATGTACTATGGGTGTTTATGATTTTAAGGTGA 10718

* ** * * ** * * * ***** *** * * *

NC_019843.3 CAACACAAGAGTTCAGATTCATGACTGCTAACAATCTAACTGCACCTAGAAATTCTTGGG 11742

NC_004718.3 CTACACAAGAATTTAGGTATATGAACTCCCAGGGGCTTTTGCCTCCTAAGAGTAGTATTG 11701

EPI_ISL_6640916_omicron CTACACAGGAGTTTAGATATATGAATTCACAGGGACTACTCCCACCCAAGAATAGCATAG 11705

MW494315.1 CTACACAGGAGTTTAGATATATGAATTCACAGGGACTACTCCCACCCAAGAATAGCATAG 11771

KF686346.1 CTGTTCAAGAATTGCGTTATATGAATGCTAATGGCTTACGTCCACCTCGTAATAGTTTTG 11986

NC_006213.1 CAGTACAGGAATTAAGATATATGAATGCTAATGGATTGCGCCCTCCTAAGAATAGTTTTG 11646

NC_002645.1 GTCCAGCCGAATTTAAGTATATGGTTGCTAATGGTTTGAATGCACCAAATGGCCCTTTTG 10859

JX504050.1 GTGCTGCTGAATTTAAATACATGGTTGCTAATGGACTTCATGCACCACATGGACCTTTTG 10778

** ** * *** * * * * ** *

NC_019843.3 AGGCTATGGCTCTGAACTTTAAGTTAATAGGTATTGGCGGTACACCTTGTATAAAGGTTG 11802

NC_004718.3 ATGCTTTCAAGCTTAACATTAAGTTGTTGGGTATTGGAGGTAAACCATGTATCAAGGTTG 11761

EPI_ISL_6640916_omicron ATGCCTTCAAACTCAACATTAAATTGTTGGGTGTTGGTGGCAAACCTTGTATCAAAGTAG 11765

MW494315.1 ATGCCTTCAAACTCAACATTAAATTGTTGGGTGTTGGTGGCAAACCTTGTATCAAAGTAG 11831

KF686346.1 AGGCTATTTTGTTAAATTTAAAACTGCTTGGAATAGGTGGCGTGCCAGTTATTGAAGTCT 12046

NC_006213.1 AAGCCCTTATGCTTAATTTTAAGCTGTTGGGTATTGGAGGTGTTCCAATCATTGAAGTAT 11706

NC_002645.1 ATGCGCTCTTTCTGTCTTTTAAACTAATGGGTATTGGCGGTCCTAGAACCATTAAAGTTT 10919

JX504050.1 ATGCACTTTGGTTATCATTCAAACTACTTGGTATTGGTGGTGACCGTTGTATAAAAATTT 10838

* ** * * * ** * * ** * ** ** ** * *

NC_019843.3 CTGCTATGCAGTCTAAACTTACAGATCTTAAATGCACATCTGTGGTTCTCCTCTCTGTGC 11862

NC_004718.3 CTACTGTACAGTCTAAAATGTCTGACGTAAAGTGCACATCTGTGGTACTGCTCTCGGTTC 11821

EPI_ISL_6640916_omicron CCACTGTACAGTCTAAAATGTCAGATGTAAAGTGCACATCAGTAGTCTTACTCTCAGTTT 11825

MW494315.1 CCACTGTACAGTCTAAAATGTCAGATGTAAAGTGCACATCAGTAGTCTTACTCTCAGTTT 11891

KF686346.1 CCCAAATTCAATCAAAATTGACTGATGTGAAATGTGCTAATGTTGTTTTGTTAAATTGTT 12106

NC_006213.1 CTCAATTTCAATCAAAATTGACTGATGTCAAATGTGCTAATGTCGTCTTGCTTAATTGCT 11766

NC_002645.1 CTACTGTACAGTCTAAATTGACTGATCTTAAGTGCACAAACGTCGTTCTAATGGGCATTT 10979

JX504050.1 CAACTGTCCAATCCAAACTGACTGATTTGAAGTGTACTAATGTTGTGTTATTGGGTTGTT 10898

* * ** ** *** * * ** * ** ** * ** ** * *

NC_019843.3 TCCAACAGTTACACTTAGAGGCTAATAGTAGGGCCTGGGCTTTCTGTGTTAAATGCCATA 11922

NC_004718.3 TTCAACAACTTAGAGTAGAGTCATCTTCTAAATTGTGGGCACAATGTGTACAACTCCACA 11881

EPI_ISL_6640916_omicron TGCAACAACTCAGAGTAGAATCATCATCTAAATTGTGGGCTCAATGTGTCCAGTTACACA 11885

MW494315.1 TGCAACAACTCAGAGTAGAATCATCATCTAAATTGTGGGCTCAATGTGTCCAGTTACACA 11951

KF686346.1 TACAGCATTTGCATGTTGCTTCTAATTCTAAGTTGTGGCAGTATTGTAGTGTTTTACATA 12166

NC_006213.1 TGCAACATTTGCATGTTGCTTCTAATTCTAAGTTGTGGCATTATTGTAGCACTTTGCACA 11826

NC_002645.1 TGTCTAACATGAACATAGCTTCTAATTCAAAGGAGTGGGCATATTGTGTTGAAATGCACA 11039

JX504050.1 TGTCTAGTATGAACATTGCAGCTAATTCTAGTGAATGGGCTTATTGTGTTGATTTACACA 10958

* * * * * * *** *** ** *

NC_019843.3 ATGATATATTGGCAGCAACAGACCCCAGTGAGGCTTTCGAGAAATTCGTAAGTCTCTTTG 11982

NC_004718.3 ATGATATTCTTCTTGCAAAAGACACAACTGAAGCTTTCGAGAAGATGGTTTCTCTTTTGT 11941

EPI_ISL_6640916_omicron ATGACATTCTCTTAGCTAAAGATACTACTGAAGCCTTTGAAAAAATGGTTTCACTACTTT 11945

MW494315.1 ATGACATTCTCTTAGCTAAAGATACTACTGAAGCCTTTGAAAAAATGGTTTCACTACTTT 12011

KF686346.1 ATGAAATACTATCTACTTCAGATTTGAGTGTAGCTTTTGATAAGCTTGCTCAATTATTGA 12226

NC_006213.1 ATGAAATACTTGCCACTTCGGATCTGAGTGTTGCTTTTGAAAAGCTTGCTCAGTTATTAA 11886

NC_002645.1 ATAAAATAA------------ACTTGTGTGACGACCCTGAAACTGCTCAAGAGTTATTGC 11087

JX504050.1 ATAAGATTA------------ATCTTTGTGATGACCCTGAAAAAGCTCAAGGTATGTTGT 11006

** * ** * ** * ** * * *

NC_019843.3 CTACTTTAATGACTTTTTCTGGTAATGTAGATCTTGATGCGT------------------ 12024

NC_004718.3 CTGTTTTGCTATCCATGCAGGGTGCTGTAGACATTAATAGGT------------------ 11983

EPI_ISL_6640916_omicron CTGTTTTGCTTTCCATGCAGGGTGCTGTAGACATAAACAAGC------------------ 11987

MW494315.1 CTGTTTTGCTTTCCATGCAGGGTGCTGTAGACATAAACAAGC------------------ 12053

KF686346.1 TTGTTTTATTCGCCAATCCTGCTGCAGTTGATACTAAGTGTCTTGCAAGTATAGATGAAG 12286

NC_006213.1 TTGTTTTGTTTGCTAATCCAGCTGCTGTGGATAGCAAGTGCCTGACTAGTATTGAAGAAG 11946

NC_002645.1 TGGCGTTGTTGGCCTTTTTCTTGTCTAAGCATAGTGATTTTG------GTCTTGGTGATC 11141

JX504050.1 TAGCACTCCTTGCGTTCTTTCTAAGTAAACATAGTGATTTTG------GTCTTGATGGCC 11060

* * * * *

NC_019843.3 TAGCTAGTGATATTTTTGACACTCCTAGCGTACTTCAAGCTACTCTTTCTGAGTTTTCAC 12084

NC_004718.3 TGTGCGAGGAAATGCTCGATAACCGTGCTACTCTTCAGGCTATTGCTTCAGAATTTAGTT 12043

EPI_ISL_6640916_omicron TTTGTGAAGAAATGCTGGACAACAGGGCAACCTTACAAGCTATAGCCTCAGAGTTTAGTT 12047

MW494315.1 TTTGTGAAGAAATGCTGGACAACAGGGCAACCTTACAAGCTATAGCCTCAGAGTTTAGTT 12113

KF686346.1 TTAGCGATGATTATGTTCAAGATAGTACCGTTTTGCAGGCTTTGCAAAGTGAGTTTGTAA 12346

NC_006213.1 TTTGCGATGATTACGCAAAGGACAATACTGTTTTGCAGGCTTTACAGAGTGAATTTGTTA 12006

NC_002645.1 TTGTCGATTCTTATTTTGAGAACGACTCCATTTTGCAAAGTGTTGCATCTTCTTTTGTTG 11201

JX504050.1 TTATTGATTCTTATTTTGATAATAGTAGCACCCTTCAGAGTGTTGCTTCATCATTTGTTA 11120

* * * ** * ***

NC_019843.3 ACTTAGCTACCTTTGCTGAGTTGGAAGCTGCGCAGAAAGCCTATCAGGAAGCTATGGACT 12144

NC_004718.3 CTTTACCATCATATGCCGCTTATGCCACTGCCCAGGAGGCCTATGAGCAGGCTGTAGCTA 12103

EPI_ISL_6640916_omicron CCCTTCCATCATATGCAGCTTTTGCTACTGCTCAAGAAGCTTATGAGCAGGCTGTTGCTA 12107

MW494315.1 CCCTTCCATCATATGCAGCTTTTGCTACTGCTCAAGAAGCTTATGAGCAGGCTGTTGCTA 12173

KF686346.1 ATATGGCTAGTTTTGTTGAATATGAAGTCGCAAAGAAAAATTTGGCTGATGCTAAAAATA 12406

NC_006213.1 ATATGGCTAGCTTCGTTGAATATGAAGTTGCTAAGAAAAATCTTGATGAGGCGCGTTTTA 12066

NC_002645.1 GTATGCCATCTTTTGTTGCATATGAAACAGCAAGACAAGAGTATGAAAATGCTGTTGCAA 11261

JX504050.1 GTATGCCATCATATATTGCTTATGAAAATGCTAGACAAGCTTATGAGGATGCTATTGCTA 11180

* * * * * * ** * * **

NC_019843.3 CTGGTGACACCTCACCACAAGTTCTTAAGGCTTTGCAGAAGGCTGTTAATATAGCTAAAA 12204

NC_004718.3 ATGGTGA---TTCTGAAGTCGTTCTCAAAAAGTTAAAGAAATCTTTGAATGTGGCTAAAT 12160

EPI_ISL_6640916_omicron ATGGTGA---TTCTGAAGTTGTTCTTAAAAAGTTGAAGAAGTCTTTGAATGTGGCTAAAT 12164

MW494315.1 ATGGTGA---TTCTGAAGTTGTTCTTAAAAAGTTGAAGAAGTCTTTGAATGTGGCTAAAT 12230

KF686346.1 GTGGTTCTGTTAATCAACAACAGATAAAACAGTTAGAAAAAGCATGTAATATAGCTAAGT 12466

NC_006213.1 GTGGTTCTGCTAATCAACAGCAGTTAAAACAGCTAGAGAAAGCCTGTAATATTGCTAAAT 12126

NC_002645.1 ATGGTTCCTC---ACCACAAATAATCAAACAATTGAAGAAGGCTATGAATGTTGCAAAAG 11318

JX504050.1 ATGGATCTTC---TTCTCAACTTATTAAACAATTGAAGCGTGCCATGAATATCGCAAAGT 11237

*** * ** * * * *** * ** **

NC_019843.3 ACGCCTATGAGAAGGATAAGGCAGTGGCCCGTAAGTTAGAACGTATGGCTGATCAGGCTA 12264

NC_004718.3 CTGAGTTTGACCGTGATGCTGCCATGCAACGCAAGTTGGAAAAGATGGCAGATCAGGCTA 12220

EPI_ISL_6640916_omicron CTGAATTTGACCGTGATGCAGCCATGCAACGTAAGTTGGAAAAGATGGCTGATCAAGCTA 12224

MW494315.1 CTGAATTTGACCGTGATGCAGCCATGCAACGTAAGTTGGAAAAGATGGCTGATCAAGCTA 12290

KF686346.1 CTGTGTATGAACGTGATAAAGCTGTAGCTCGCAAACTCGAACGTATGGCAGACCTAGCAC 12526

NC_006213.1 CTGCTTATGAACGCGACCGTGCTGTAGCAAAAAAGTTGGAGCGTATGGCTGATTTGGCTC 12186

NC_002645.1 CTGAGTTTGACAGGGAATCATCTGTTCAAAAGAAAATTAACAGAATGGCTGAACAAGCTG 11378

JX504050.1 CTGAATTTGATCATGAGATATCTGTTCAGAAGAAAATTAATAGAATGGCTGAACAAGCTG 11297

* * *** ** * * ** * * ***** ** **

NC_019843.3 TGACTTCTATGTATAAGCAAGCACGTGCTGAAGACAAGAAAGCAAAAATTGTCAGTGCTA 12324

NC_004718.3 TGACCCAAATGTACAAACAGGCAAGATCTGAGGACAAGAGGGCAAAAGTAACTAGTGCTA 12280

EPI_ISL_6640916_omicron TGACCCAAATGTATAAACAGGCTAGATCTGAGGACAAGAGGGCAAAAGTTACTAGTGCTA 12284

MW494315.1 TGACCCAAATGTATAAACAGGCTAGATCTGAGGACAAGAGGGCAAAAGTTACTAGTGCTA 12350

KF686346.1 TTACTAACATGTATAAAGAGGCTCGGATTAATGATAAGAAGAGTAAAGTTGTTTCCGCTT 12586

NC_006213.1 TCACTAATATGTATAAAGAAGCTAGAATTAATGATAAGAAGAGTAAGGTTGTTTCTGCCT 12246

NC_002645.1 CTGCAGCTATGTACAAAGAAGCACGTGCTGTTAATAGAAAATCAAAAGTTGTTAGTGCCA 11438

JX504050.1 CTACTCAGATGTATAAAGAAGCACGCTCTGTTAATAGAAAATCTAAAGTTATTAGTGCTA 11357

* ***** ** * ** * * * * * ** * **

NC_019843.3 TGCAAACTATGTTGTTTGGTATGATTAAGAAGCTCGACAACGATGTTCTTAATGGTATCA 12384

NC_004718.3 TGCAAACAATGCTCTTCACTATGCTTAGGAAGCTTGATAATGATGCACTTAACAACATTA 12340

EPI_ISL_6640916_omicron TGCAGACAATGCTTTTCACTATGCTTAGAAAGTTGGATAATGATGCACTCAACAACATTA 12344

MW494315.1 TGCAGACAATGCTTTTCACTATGCTTAGAAAGTTGGATAATGATGCACTCAACAACATTA 12410

KF686346.1 TGCAGACAATGCTTTTTAGCATGGTTCGTAAATTGGATAATCAGGCTTTAAATTCTATTC 12646

NC_006213.1 TGCAAACTATGCTTTTTAGTATGGTGCGTAAGTTAGATAATCAAGCTCTGAATTCAATAT 12306

NC_002645.1 TGCATAGTTTACTCTTTGGCATGCTCCGACGTTTGGACATGTCTAGTGTTGACACTATCC 11498

JX504050.1 TGCACTCTTTACTTTTTGGAATGTTAAGACGTTTGGATATGTCTAGTGTTGAAACTGTTT 11417

**** * * ** *** * * ** * * * *

NC_019843.3 TTTCTAACGCTAGGAATGGTTGTATACCTCTTAGTGTCATCCCACTGTGTGCTTCAAATA 12444

NC_004718.3 TCAACAATGCGCGTGATGGTTGTGTTCCACTCAACATCATACCATTGACTACAGCAGCCA 12400

EPI_ISL_6640916_omicron TCAACAATGCAAGAGATGGTTGTGTTCCCTTGAACATAATACCTCTTACAACAGCAGCCA 12404

MW494315.1 TCAACAATGCAAGAGATGGTTGTGTTCCCTTGAACATAATACCTCTTACAACAGCAGCCA 12470

KF686346.1 TGGATAATGCTGTTAAAGGTTGTGTACCTTTGAGTGCTATTCCAGCATTGGCTGCTAATA 12706

NC_006213.1 TAGATAACGCTGTGAAGGGTTGTGTACCATTGAATGCAATACCTTCATTGGCAGCAAATA 12366

NC_002645.1 TTAATATGGCACGTAATGGTGTTGTCCCTCTTTCCGTTATCCCTGCTACTTCTGCAGCCA 11558

JX504050.1 TGAATTTAGCACGTGATGGTGTTGTGCCATTGTCAGTTATACCTGCAACTTCAGCTTCTA 11477

* ** * *** * * ** * ** ** * * *

NC_019843.3 AACTTCGCGTTGTAATTCCTGACTTCACCGTCTGGAATCAGGTAGTCACATATCCCTCGC 12504

NC_004718.3 AACTCATGGTTGTTGTCCCTGATTATGGTACCTACAAGAACACTTGTGATGGTAACACCT 12460

EPI_ISL_6640916_omicron AACTAATGGTTGTCATACCAGACTATAACACATATAAAAATACGTGTGATGGTACAACAT 12464

MW494315.1 AACTAATGGTTGTCATACCAGACTATAACACATATAAAAATACGTGTGATGGTACAACAT 12530

KF686346.1 CTTTAACTATAATAATACCAGATAAACAAGTTTTTGATAAAGTTGTTGATAATGTTTATG 12766

NC_006213.1 CTCTGAATATAATTGTACCAGATAAAAGTGTTTATGACCAGGTAGTTGATAATGTCTATG 12426

NC_002645.1 GGCTCGTCGTCGTAGTACCAGATCATGATTCATTTGTGAAAATGATGGTAGATGGTTTTG 11618

JX504050.1 AACTAACTATTGTTAGTCCAGATCTTGAATCTTATTCTAAGATTGTTTGTGATGGTTCTG 11537

* * * ** ** * * *

NC_019843.3 TTAACTACGCTGGGGCTTTGTGGGACATTACAGTTATAAACAATGTGGACAATGAAATTG 12564

NC_004718.3 TTACATATGCATCTGCACTCTGGGAAATCCAGCAAGTTGTTGATGCGGATAGCAAGATTG 12520

EPI_ISL_6640916_omicron TTACTTATGCATCAGCATTGTGGGAAATCCAACAGGTTGTAGATGCAGATAGTAAAATTG 12524

MW494315.1 TTACTTATGCATCAGCATTGTGGGAAATCCAACAGGTTGTAGATGCAGATAGTAAAATTG 12590

KF686346.1 TTACATATGCTGGTAGTGTATGGCATATACAGACTGTTCAAGATGCTGATGGTATTAATA 12826

NC_006213.1 TTACCTATGCGGGTAATGTATGGCAGATTCAAACTATCCAGGATTCAGATGGTACAAATA 12486

NC_002645.1 TGCACTACGCTGGTGTTGTTTGGACATTACAGGAAGTTAAGGATAATGATGGTAAGAATG 11678

JX504050.1 TTCATTATGCTGGAGTTGTTTGGACACTTAATGATGTTAAAGACAATGATGGTAGACCTG 11597

* ** ** * *** * * * ** *

NC_019843.3 TTAAGTCTTCAGATGTT---GTAGACAGCAATGAAAATTTAACATGGCCACTTGTTTTAG 12621

NC_004718.3 TTCAACTTAGTGAAATTAACATGGACAATTCACCAAATTTGGCTTGGCCTCTTATTGTTA 12580

EPI_ISL_6640916_omicron TTCAACTTAGTGAAATTAGTATGGACAATTCACCTAATTTAGCATGGCCTCTTATTGTAA 12584

MW494315.1 TTCAACTTAGTGAAATTAGTATGGACAATTCACCTAATTTAGCATGGCCTCTTATTGTAA 12650

KF686346.1 AACAGTTAACTGATATTAGTGTTGATTCTAATTGGCCTCTTGTTATCATTGCGAACAGGT 12886

NC_006213.1 AGCAGTTGAATGAGATATCTGATGATTGTAACTGGCCACTAGTTATTATTGCAAATCGGT 12546

NC_002645.1 TGCATCTTAAAGATGTTACAAAGGAAAACCAGGAAATACTTGTTTGGCCTCTGATTTTGA 11738

JX504050.1 TTCATGTTAAAGAGATTACAAAGGAAAATGTTGAAACTTTGACATGGCCTCTTATCCTTA 11657

* ** * ** *

NC_019843.3 AATGCACTAGGGCATCCACTTCTGCCGTTAA---GTTGCAAAATAATGAGATCAAACCTT 12678

NC_004718.3 CAGCTCTAAGAG---CCAACTCAGCTGTTAA---ACTACAGAATAATGAACTGAGTCCAG 12634

EPI_ISL_6640916_omicron CAGCTTTAAGGG---CCAATTCTGCTGTCAA---ATTACAGAATAATGAGCTTAGTCCTG 12638

MW494315.1 CAGCTTTAAGGG---CCAATTCTGCTGTCAA---ATTACAGAATAATGAGCTTAGTCCTG 12704

KF686346.1 ATAATGAAGTTG------------CTAATGCTGTTATGCAGAATAATGAGTTGATGCCTC 12934

NC_006213.1 ATAATGAGGTAT------------CTGCTACTGTTTTGCAAAATAATGAATTAATGCCTG 12594

NC_002645.1 CTTGTGAACGTG------------TCGTTAA---ATTGCAGAACAATGAAATAATGCCGG 11783

JX504050.1 ATTGTGAACGTG------------TTGTTAA---ACTTCAAAATAATGAAATTATGCCTG 11702

* ** ** ***** * * **

NC_019843.3 CAGGTCTAAAAACCATGGTTGTGTCTGCGGGTCAAGAGCAAACTAACTGTAATACT---A 12735

NC_004718.3 TAGCACTACGACAGATGTCCTGTGCGGCTGGTACCACACAAACAGCTTGTACTGATGACA 12694

EPI_ISL_6640916_omicron TTGCACTACGACAGATGTCTTGTGCTGCCGGTACTACACAAACTGCTTGCACTGATGACA 12698

MW494315.1 TTGCACTACGACAGATGTCTTGTGCTGCCGGTACTACACAAACTGCTTGCACTGATGACA 12764

KF686346.1 ATAAATTAAAAATACAAGTTGTTAATAGTGGTTCTGATATGAATTGTAATATTCCT---- 12990

NC_006213.1 CTAAGTTGAAAATTCAGGTTGTTAATAGTGGTCCAGATCAGACTTGTAATACACCT---- 12650

NC_002645.1 GCAAGATGAAGGTCAAGGCCACCAAAGGTGAAGGTGATGGAGGCATTACTAGTGAA---- 11839

JX504050.1 GTAAACTTAAGCAAAAACCTATGAAAGCTGAGGGTGATGGTGGTGTTTTAGGTGAT---- 11758

* *

NC_019843.3 GTTCCTTAGCTTATTACGAACCTGTGCAGGGTCGTAAAATGCTGATGGCTCTTCTTTCTG 12795

NC_004718.3 ATGCACTTGCCTACTATAACAATTCGAAGGGAGGTAGGTTTGTGCTGGCATTACTATCAG 12754

EPI_ISL_6640916_omicron ATGCGTTAGCTTACTACAACACAACAAAGGGAGGTAGGTTTGTACTTGCACTGTTATCCG 12758

MW494315.1 ATGCGTTAGCTTACTACAACACAACAAAGGGAGGTAGGTTTGTACTTGCACTGTTATCCG 12824

KF686346.1 --ACTCAATGTTATTATAATAATGGTAGTAGTGGTAGAATAGTTTATGCTGTTCTTAGTG 13048

NC_006213.1 --ACTCAATGTTACTATAATAATAGTAACAATGGGAAGATTGTTTATGCTATACTTAGTG 12708

NC_002645.1 --GGTAATGCTCTATACAACAATGAAGGTGGACGTGCATTCATGTATGCATATGTGACTA 11897

JX504050.1 --GGTAATGCCTTGTATAATACTGAGGGTGGTAAAACTTTTATGTACGCTTATATTTCTA 11816

** * * * ** *

NC_019843.3 ATAATGCCTATCTCAAATGGGCGCGT---GTTGAAGGTAAGGACGGATTTGTCAGTGT-- 12850

NC_004718.3 ACCACCAAGATCTCAAATGGGCTAGA---TTCCCTAAGAGTGATGGTACAGGTACAATTT 12811

EPI_ISL_6640916_omicron ATTTACAGGATTTGAAATGGGCTAGA---TTCCCTAAGAGTGATGGAACTGGTACTATCT 12815

MW494315.1 ATTTACAGGATTTGAAATGGGCTAGA---TTCCCTAAGAGTGATGGAACTGGTACTATCT 12881

KF686346.1 ATGTTGATGGTCTTAAGTATACTAAGATAATGAAAGATGATGGAAATTGTGTTGTTTT-- 13106

NC_006213.1 ATGTTGATGGTCTTAAGTATACAAAAATTCTTAAAGATGATGGCAATTTTGTTGTTTT-- 12766

NC_002645.1 CGAAGCCTGGCATGAAGTATGTTAAA---TGGGAACATGACTCTGGTGTGGTTACAGT-- 11952

JX504050.1 ATAAAGCTGACCTTAAATTTGTTAAG---TGGGAGTATGAGGGTGGTTGCAACACAAT-- 11871

* ** * *

NC_019843.3 ----AGAGCTACAACCTCCTTGCAAATTCTTGATTGCGGGACCAAAAGGACCTGAAATCC 12906

NC_004718.3 ACACAGAACTGGAACCACCTTGTAGGTTTGTTACAGACACACCAAAAGGGCCTAAAGTGA 12871

EPI_ISL_6640916_omicron ATACAGAACTGGAACCACCTTGTAGGTTTGTTACAGACACACCTAAAGGTCCTAAAGTGA 12875

MW494315.1 ATACAGAACTGGAACCACCTTGTAGGTTTGTTACAGACACACCTAAAGGTCCTAAAGTGA 12941

KF686346.1 ----AGAGCTTGATCCTCCTTGTAAATTTTCTATACAAGATGTTAAGGGACTTAAAATTA 13162

NC_006213.1 ----GGAGTTAGATCCTCCTTGTAAATTTACTGTTCAAGATGCTAAAGGTCTTAAAATTA 12822

NC_002645.1 ----TGAATTGGAACCACCTTGCAGATTTGTTATAGACACACCTACTGGACCCCAAATTA 12008

JX504050.1 ----CGAGTTAGACTCTCCTTGTCGATTTATGGTCGAAACACCTAATGGTCCTCAAGTGA 11927

** * * * ***** ** * ** * ** *

NC_019843.3 GATATCTCTATTTTGTTAAAAATCTTAACAACCTTCATCGCGGGCAAGTGTTAGGGCACA 12966

NC_004718.3 AATACTTGTACTTCATCAAAGGCTTAAACAACCTAAATAGAGGTATGGTGCTGGGCAGTT 12931

EPI_ISL_6640916_omicron AGTATTTATACTTTATTAAAGGATTAAACAACCTAAATAGAGGTATGGTACTTGGTAGTT 12935

MW494315.1 AGTATTTATACTTTATTAAAGGATTAAACAACCTAAATAGAGGTATGGTACTTGGTAGTT 13001

KF686346.1 AGTATCTTTATTTTATTAAAGGATGTAACACTTTAGCTAGAGGGTGGGTTGTTGGTACTT 13222

NC_006213.1 AGTACCTTTATTTTGTAAAAGGTTGTAACACACTAGCAAGAGGCTGGGTTGTTGGTACAA 12882

NC_002645.1 AGTATCTTTATTTTGTTAAGAATCTTAACAATTTAAGGAGAGGTGCTGTTTTGGGTTACA 12068

JX504050.1 AGTATTTGTATTTTGTTAAAAATTTAAATACCTTACGTAGAGGTGCCGTTCTTGGTTTTA 11987

** * ** ** * ** ** * * * ** ** * **

NC_019843.3 TTGCTGCGACTGTTAGATTGCAAGCTGGTTCTAACACCGAGTTTGCCTCTAATTCCTCGG 13026

NC_004718.3 TAGCTGCTACAGTACGTCTTCAGGCTGGAAATGCTACAGAAGTACCTGCCAATTCAACTG 12991

EPI_ISL_6640916_omicron TAGCTGCCACAGTACGTCTACAAGCTGGTAATGCAACAGAAGTGCCTGCCAATTCAACTG 12995

MW494315.1 TAGCTGCCACAGTACGTCTACAAGCTGGTAATGCAACAGAAGTGCCTGCCAATTCAACTG 13061

KF686346.1 TATCTTCAACAATTAGATTGCAGGCTGGTGTTGCTACTGAGTATGCAGCTAATTCTTCTA 13282

NC_006213.1 TTTCTTCTACAGTTAGATTGCAAGCTGGAACTGCTACTGAATATGCTTCCAACTCATCTA 12942

NC_002645.1 TTGGTGCCACTGTGAGATTGCAAGCTGGCAAACAGACTGAGTTTGTTTCAAACTCCCATT 12128

JX504050.1 TAGGTGCCACAATTCGTCTACAAGCTGGTAAACAAACTGAATTGGCTGTTAATTCTGGAC 12047

* * * ** * * * ** ***** ** ** ** **

NC_019843.3 TGTTGTCACTTGTTAACTTCACCGTTGATCCTCAAAAAGCTTATCTCGATTTCGTCAATG 13086

NC_004718.3 TGCTTTCCTTCTGTGCTTTTGCAGTAGACCCTGCTAAAGCATATAAGGATTACCTAGCAA 13051

EPI_ISL_6640916_omicron TATTATCTTTCTGTGCTTTTGCTGTAGATGCTGCTAAAGCTTACAAAGATTATCTAGCTA 13055

MW494315.1 TATTATCTTTCTGTGCTTTTGCTGTAGATGCTGCTAAAGCTTACAAAGATTATCTAGCTA 13121

KF686346.1 TACTTTCATTATGTGCATTTTCTGTAGATCCTAAGAAAACTTATTTAGATTATATACAAC 13342

NC_006213.1 TATTGTCTTTATGTGCGTTTTCTGTAGATCCTAAGAAAACGTATTTAGATTTTATACAAC 13002

NC_002645.1 TATTAACACATTGTTCTTTTGCTGTTGACCCAGCTGCAGCCTATCTTGATGCTGTTAAAC 12188

JX504050.1 TTTTAACTGCTTGTGCTTTTTCTGTTGATCCAGCAACTACTTACTTGGAAGCTGTTAAAC 12107

* * * * ** * ** ** * * ** ** *

NC_019843.3 CGGGAGGTGCCCCATTGACAAATTGTGTTAAGATGCTTACTCCTAAAACTGGTACAGGTA 13146

NC_004718.3 GTGGAGGACAACCAATCACCAACTGTGTGAAGATGTTGTGTACACACACTGGTACAGGAC 13111

EPI_ISL_6640916_omicron GTGGGGGACAACCAATCACTAATTGTGTTAAGATGTTGTGTACACACACTGGTACTGGTC 13115

MW494315.1 GTGGGGGACAACCAATCACTAATTGTGTTAAGATGTTGTGTACACACACTGGTACTGGTC 13181

KF686346.1 AAGGTGGTGTACCTATAATTAATTGTGTTAAAATGCTCTGTGATCATGCTGGTACTGGTA 13402

NC_006213.1 AAGGAGGAACACCTATTGCCAATTGTGTTAAAATGTTGTGTGACCATGCTGGTACCGGTA 13062

NC_002645.1 AAGGCGCAAAACCTGTTGGCAATTGTGTAAAGATGTTGACTAATGGTTCTGGTAGCGGTC 12248

JX504050.1 ATGGTGCAAAACCTGTAAGTAATTGTATTAAGATGTTATCTAATGGTGCTGGTAATGGTC 12167

** * ** * ** *** * ** *** * * ****** **

NC_019843.3 TAGCTATATCTGTTAAACCAGAGAGTACAGCTGATCAAGAGACTTATGGTGGAGCTTCAG 13206

NC_004718.3 AGGCAATTACTGTAACACCAGAAGCTAACATGGACCAAGAGTCCTTTGGTGGTGCTTCAT 13171

EPI_ISL_6640916_omicron AGGCAATAACAGTCACACCGGAAGCCAATATGGATCAAGAATCCTTTGGTGGTGCATCGT 13175

MW494315.1 AGGCAATAACAGTTACACCGGAAGCCAATATGGATCAAGAATCCTTTGGTGGTGCATCGT 13241

KF686346.1 TGGCCATTACTATTAAACCTGAGGCTACTATTAACCAAGATTCTTATGGTGGTGCCTCTG 13462

NC_006213.1 TGGCCATTACTGTTAAACCCGATGCTACCACTAGTCAGGATTCATATGGTGGTGCGTCTG 13122

NC_002645.1 AGGCTATTACTTGTACCATTGATTCCAACACTACGCAGGACACATATGGTGGCGCGTCTG 12308

JX504050.1 AAGCTATAACAACTAGTGTAGATGCTAACACCAATCAAGATTCTTATGGTGGAGCGTCTA 12227

** ** * * ** * ** ** * * ****** ** **

NC_019843.3 TGTGTCTCTATTGCCGTGCGCATATAGAACATCCTGATGTCTCTGGTGTTTGTAAATATA 13266

NC_004718.3 GTTGTCTGTATTGTAGATGCCACATTGACCATCCAAATCCTAAAGGATTCTGTGACTTGA 13231

EPI_ISL_6640916_omicron GTTGTCTGTACTGCCGTTGCCACATAGATCATCCAAATCCTAAAGGATTTTGTGACTTAA 13235

MW494315.1 GTTGTCTGTACTGCCGTTGCCACATAGATCATCCAAATCCTAAAGGATTTTGTGACTTAA 13301

KF686346.1 TTTGTATTTATTGCCGTGCACGTGTAGAGCATCCAGATGTAGATGGTATATGTAAATTAC 13522

NC_006213.1 TTTGTATATATTGCCGCGCACGAGTTGAACACCCAGATGTTGATGGGTTGTGCAAATTAC 13182

NC_002645.1 TTTGTATTTATTGCAGAGCACATGTTGCACATCCAACCATGGACGGTTTTTGTCAGTACA 12368

JX504050.1 TTTGTTTGTATTGTCGGGCCCACGTTCCTCACCCTAGTATGGATGGTTACTGTAAGTTTA 12287

*** * ** ** * * * ** ** ** ** * *

NC_019843.3 AGGGTAAGTTTGTCCAAATCCCTGCTCAGTGTGTCCGTGACCCTGTGGGATTTTGTTTGT 13326

NC_004718.3 AAGGTAAGTACGTCCAAATACCTACCACTTGTGCTAATGACCCAGTGGGTTTTACACTTA 13291

EPI_ISL_6640916_omicron AAGGTAAGTATGTACAAATACCTACAACTTGTGCTAATGACCCTGTGGGTTTTACACTTA 13295

MW494315.1 AAGGTAAGTATGTACAAATACCTACAACTTGTGCTAATGACCCTGTGGGTTTTACACTTA 13361

KF686346.1 GTGGTAAATTTGTACAAGTCCCTTTGG---GTATAAAAGACCCTATTCTTTATGTGTTAA 13579

NC_006213.1 GCGGCAAGTTTGTACAAGTGCCTGTAG---GTATAAAAGATCCTGTGTCTTATGTTTTGA 13239

NC_002645.1 AAGGCAAGTGGGTACAAGTGCCTATAG---GTACAAATGACCCTATAAGATTTTGTCTTG 12425

JX504050.1 AGGGTAAATGTGTTCAGGTTCCTATTG---GTTGTTTGGATCCTATTAGGTTTTGTTTAG 12344

** ** * ** ** * *** ** ** ** * * * *

NC_019843.3 CAAATACCCCCTGTAATGTCTGTCAATATTGGATTGGATATGGGTGCAATTGTGACTCGC 13386

NC_004718.3 GAAACACAGTCTGTACCGTCTGCGGAATGTGGAAAGGTTATGGCTGTAGTTGTGACCAAC 13351

EPI_ISL_6640916_omicron AAAACACAGTCTGTACCGTCTGCGGTATGTGGAAAGGTTATGGCTGTAGTTGTGATCAAC 13355

MW494315.1 AAAACACAGTCTGTACCGTCTGCGGTATGTGGAAAGGTTATGGCTGTAGTTGTGATCAAC 13421

KF686346.1 CACATGATGTTTGTCAAGTCTGTGGTTTTTGGAGAGATGGCAGTTGTTCCTGTGTAGGTT 13639

NC_006213.1 CACATGATGTTTGTCGAGTTTGTGGATTTTGGCGGGATGGAAGTTGTTCATGTGTTAGCA 13299

NC_002645.1 AAAATACTGTTTGTAAAGTTTGTGGTTGTTGGCTTAATCATGGCTGTACATGTGACCGGA 12485

JX504050.1 AAAATAATGTGTGTAATGTTTGTGGTTGTTGGTTGGGACACGGGTGTGCTTGTGACCGTA 12404

* * *** ** ** *** * ** ****

NC_019843.3 TTAGGCAAGCAGCACTGCCCCAATCTAAAGATTCCAATTTTTTAAACGAGTCCGGGGTTC 13446

NC_004718.3 TCCGCGAACCCTTGATGCAGTCTGCGGATGCATCAACGTTTTTAAACGGGTTTGCGGTGT 13411

EPI_ISL_6640916_omicron TCCGCGAACCCATGCTTCAGTCAGCTGATGCACAATCGTTTTTAAACGGGTTTGCGGTGT 13415

MW494315.1 TCCGCGAACCCATGCTTCAGTCAGCTGATGCACAATCGTTTTTAAACGGGTTTGCGGTGT 13481

KF686346.1 CAAGTGTCGCTGTTC------AATCTAAAGATTTAAATTTTTTAAACGGGTTCGGGGTAC 13693

NC_006213.1 CTGACACTACTGTTC------AATCAAAAGATACTAATTTTTTAAACGGGTTCGGGGTAC 13353

NC_002645.1 CTG------CTATCC------AAAGTTTTGATAACAGTTATTTAAACGAGTCCGGGGCTC 12533

JX504050.1 CAA------CTATTC------AAAGTGTTGACATTTCTTATTTAAACGAGCAAGGGGTTC 12452

* * * ******** * * **

NC_019843.3 TATTGTAAATGCCCGAATAGAACCCTGTTCAAGTGGTTTGTCCACTGATGTCGTCTTTAG 13506

NC_004718.3 AAGTGCA---GCCCGTCTTACACCGTGCGGCACAGGCACTAGTACTGATGTCGTCTACAG 13468

EPI_ISL_6640916_omicron AAGTGCA---GCCCGTCTTACACCGTGCGGCACAGGCACTAGTACTGATGTCGTATACAG 13472

MW494315.1 AAGTGCA---GCCCGTCTTACACCGTGCGGCACAGGCACTAGTACTGATGTCGTATACAG 13538

KF686346.1 TAGTGTGAATGCCCGGCTAGTACCCTGTGCTAGTGGTTTATCTACTGATGTTCAATTAAG 13753

NC_006213.1 GAGTGTAGATGCCCGTCTCGTACCCTGCGCCAGTGGTTTATCTACTGATGTACAATTAAG 13413

NC_002645.1 TAGTGCC---GCTCGACTAGAGCCCTG---TAATGGTACAGACATAGATTACTGTGTCCG 12587

JX504050.1 TAGTGCA---GCTCGACTAGAACCCTG---TAATGGCACGGACATCGATAAGTGTGTTCG 12506

* ** ** ** * ** ** * ** * *** *

NC_019843.3 GGCATTTGACATCTGCAACTATAAGGCTAAGGTTGCTGGTATTGGAAAATACTACAAGAC 13566

NC_004718.3 GGCTTTTGATATTTACAAC------GAAAAAGTTGCTGGTTTTGCAAAGTTCCTAAAAAC 13522

EPI_ISL_6640916_omicron GGCTTTTGACATCTACAAT------GATAAAGTAGCTGGTTTTGCTAAATTCCTAAAAAC 13526

MW494315.1 GGCTTTTGACATCTACAAT------GATAAAGTAGCTGGTTTTGCTAAATTCCTAAAAAC 13592

KF686346.1 GGCATTTGATATTTGTAAT------ACCAATAGAGCTGGTATAGGTTTATATTATAAAGT 13807

NC_006213.1 GGCATTTGATATTTACAAT------GCTAGTGTTGCTGGCATTGGTTTACATTTAAAAGT 13467

NC_002645.1 TGCATTTGACGTTTACAAT------AAAGATGCGTCTTTTATCGGAAAAAATCTGAAGTC 12641

JX504050.1 TGCTTTTGACATTTATAAT------AAAAATGTTTCATTCTTGGGTAAGTGTTTGAAGAT 12560

** ***** * * ** * * * **

NC_019843.3 TAATACTTGTAGGTTTGTAGAATTAGATGACCAAGGGCATCATTTAGACTCCTATTTTGT 13626

NC_004718.3 TAATTGCTGTCGCTTCCAGGAGAAGGATGAGGAAGGCAATTTATTAGACTCTTACTTTGT 13582

EPI_ISL_6640916_omicron TAATTGTTGTCGCTTCCAAGAAAAGGACGAAGATGACAATTTAATTGATTCTTACTTTGT 13586

MW494315.1 TAATTGTTGTCGCTTCCAAGAAAAGGACGAAGATGACAATTTAATTGATTCTTACTTTGT 13652

KF686346.1 GAATTGTTGCCGTTTTCAGCGTATAGATGACGACGGTAATAAATTGGATAAGTTCTTTGT 13867

NC_006213.1 TAATTGTTGCCGTTTTCAGCGTGTTGATGAGAACGGTGATAAATTAGATCAGTTCTTTGT 13527

NC_002645.1 CAATTGTGTGCGCTTCAAGAATGTAGATAAGGA------------TGACGCGTTCTATAT 12689

JX504050.1 GAACTGTGTTCGTTTTAAAAATGCTGATCTTAA------------GGATGGTTATTTTGT 12608

** * ** ** * ** * * * *

NC_019843.3 CGTTAAGAGGCATACTATGGAGAATTATGAACTAGAGAAGCACTGTTACGACTTGTTACG 13686

NC_004718.3 AGTTAAGAGGCATACTATGTCTAACTACCAACATGAAGAGACTATTTATAACTTGGTTAA 13642

EPI_ISL_6640916_omicron AGTTAAGAGACACACTTTCTCTAACTACCAACATGAAGAAACAATTTATAATTTACTTAA 13646

MW494315.1 AGTTAAGAGACACACTTTCTCTAACTACCAACATGAAGAAACAATTTATAATTTACTTAA 13712

KF686346.1 TGTCAAAAGAACTAATTTAGAAGTTTATAATAAAGAGAAAACTTATTATGAGTTGACTAA 13927

NC_006213.1 TGTTAAGAGGACAGATCTGACTATATATAATAGAGAGATGAAATGCTATGAGCGTGTAAA 13587

NC_002645.1 TGTTAAACGTTGCATTAAGTCAGTTATGGACCACGAGCAGTCCATGTATAACTTACTTAA 12749

JX504050.1 TATAAAGAGGTGTACTAAGTCGGTTATGGAACACGAGCAATCCATGTATAACCTACTTAA 12668

* ** * * * ** ** *

NC_019843.3 TGACTGTGATGCTGTAGCTCCCCATGATTTCTTCATCTTTGATGTAGACAAAGTTAAAAC 13746

NC_004718.3 AGATTGTCCAGCGGTTGCTGTCCATGACTTTTTCAAGTTTAGAGTAGATGGTGACATGGT 13702

EPI_ISL_6640916_omicron GGATTGTCCAGCTGTTGCTAAACATGACTTCTTTAAGTTTAGAATAGACGGTGACATGGT 13706

MW494315.1 GGATTGTCCAGCTGTTGCTAAACATGACTTCTTTAAGTTTAGAATAGACGGTGACATGGT 13772

KF686346.1 AAGTTGTGGTGTTGTGGCTGAACATGATTTCTTTACATTTGATATTGATGGTAGTCGCGT 13987

NC_006213.1 AGATTGTAAGTTTGTGGCTGAACACGATTTCTTTACATTTGATGTAGAAGGTAGTCGTGT 13647

NC_002645.1 AGGCTGTAATGCTGTTGCTAAGCATGATTTCTTTACTTGGCATGAGGGCAGAACCATTTA 12809

JX504050.1 CTTTTCTGGTGCTTTGGCTGAGCATGATTTCTTTACTTGGAAAGATGGCAGAGTCATTTA 12728

* * * *** ** ** ** ** * * *

NC_019843.3 ACCTCATATTGTACGTCAGCGTTTAACTGAGTACACTATGATGGATCTTGTATATGCCCT 13806

NC_004718.3 ACCACATATATCACGTCAGCGTCTAACTAAATACACAATGGCTGATTTAGTCTATGCTCT 13762

EPI_ISL_6640916_omicron ACCACATATATCACGTCAACGTCTTACTAAATACACAATGGCAGACCTCGTCTATGCTTT 13766

MW494315.1 ACCACATATATCACGTCAACGTCTTACTAAATACACAATGGCAGACCTCGTCTATGCTTT 13832

KF686346.1 GCCACATATAGTTCGTAGGAATCTTTCAAAGTATACTATGTTAGATCTTTGCTATGCATT 14047

NC_006213.1 GCCACACATTGTACGCAAGGATTTAACAAAGTATACTATGTTGGATCTTTGCTATGCATT 13707

NC_002645.1 TGGTAATGTTAGTAGACAGGATCTTACTAAATACACCATGATGGATTTGTGCTTCGCTCT 12869

JX504050.1 TGGTAATGTTAGTAGACATAATCTTACTAAATATACTATGATGGACTTGGTCTATGCTAT 12788

* * * * * * * ** ** *** ** * * ** *

NC_019843.3 GAGGCACTTTGATCAA---AATAGCGAAGTGCTTAAGGCTATCTTAGTGAAGTATGGTTG 13863

NC_004718.3 ACGTCATTTTGATGAGGGTAATTGTGATACATTAAAAGAAATACTCGTCACATACAATTG 13822

EPI_ISL_6640916_omicron AAGGCATTTTGATGAAGGTAATTGTGACACATTAAAAGAAATACTTGTCACATACAATTG 13826

MW494315.1 AAGGCATTTTGATGAAGGTAATTGTGACACATTAAAAGAAATACTTGTCACATACAATTG 13892

KF686346.1 GCGTCATTTTGATCGTAATGATTGTTCAATATTGTGTGAAATTCTTTGTGAGTATGCTGA 14107

NC_006213.1 GCGACATTTTGATCGCAATGATTGCATGCTGCTTTGTGACATTCTCTCTATATATGCTGG 13767

NC_002645.1 GCGTAACTTTGATGAAAAAGACTGTGAAGTTTTTAAGGAGATATTGGTTCTTACTGGTTG 12929

JX504050.1 GCGTAACTTTGATGAACAAAATTGTGATGTTCTAAAAGAAGTATTAGTTTTAACTGGTTG 12848

* * ****** * * * * * * *

NC_019843.3 CTGTGATGTTACCTACTTTGAAAATAAACTCTGGTTTGATTTTGTTGAAAATCCCAGTGT 13923

NC_004718.3 CTGTGATGATGATTATTTCAATAAGAAGGATTGGTATGACTTCGTAGAGAATCCTGACAT 13882

EPI_ISL_6640916_omicron TTGTGATGATGATTATTTCAATAAAAAGGACTGGTATGATTTTGTAGAAAACCCAGATAT 13886

MW494315.1 TTGTGATGATGATTATTTCAATAAAAAGGACTGGTATGATTTTGTAGAAAACCCAGATAT 13952

KF686346.1 TTGTAAAGAATCCTACTTTTCTAAGAAAGATTGGTATGATTTTGTTGAAAATCCTGATAT 14167

NC_006213.1 TTGTGAACAATCCTACTTTACTAAGAAGGATTGGTATGATTTTGTTGAAAATCCTGATAT 13827

NC_002645.1 TTGTAGTACTGATTACTTTGAAATGAAGAATTGGTTTGACCCCATAGAAAATGAGGACAT 12989

JX504050.1 TTGTGACAATTCTTATTTTGATAGTAAGGGTTGGTATGACCCAGTTGAAAATGAAGATAT 12908

*** ** ** * ** **** *** * ** ** *

NC_019843.3 TATTGGTGTTTATCATAAACTTGGAGAACGTGTACGCCAAGCTATCTTAAACACTGTTAA 13983

NC_004718.3 CTTACGCGTATATGCTAACTTAGGTGAGCGTGTACGCCAATCATTATTAAAGACTGTACA 13942

EPI_ISL_6640916_omicron ATTACGCGTATACGCCAACTTAGGTGAACGTGTACGCCAAGCTTTGTTAAAAACAGTACA 13946

MW494315.1 ATTACGCGTATACGCCAACTTAGGTGAACGTGTACGCCAAGCTTTGTTAAAAACAGTACA 14012

KF686346.1 TATTAATATATATAAAAAATTAGGCCCTATTTTTAATAGAGCTTTACTTAATACTGTCAT 14227

NC_006213.1 TATTAATGTGTATAAAAAGCTAGGACCTATTTTTAATAGAGCCCTAGTTAGCGCTACTGA 13887

NC_002645.1 ACACCGTGTGTATGCTGCTTTAGGTAAGGTAGTTGCAAATGCAATGCTTAAGTGTGTTGC 13049

JX504050.1 ACATAGAGTTTATGCATCTCTTGGCAAAATTGTAGCTAGAGCTATGCTTAAATGCGTTGC 12968

* ** * ** * * * * *

NC_019843.3 ATTTTGTGACCACATGGTCAAGGCTGGTTTAGTCGGTGTGCTCACACTAGACAACCAGGA 14043

NC_004718.3 ATTCTGCGATGCTATGCGTGATGCAGGCATTGTAGGCGTACTGACATTAGATAATCAGGA 14002

EPI_ISL_6640916_omicron ATTCTGTGATGCCATGCGAAATGCTGGTATTGTTGGTGTACTGACATTAGATAATCAAGA 14006

MW494315.1 ATTCTGTGATGCCATGCGAAATGCTGGTATTGTTGGTGTACTGACATTAGATAATCAAGA 14072

KF686346.1 TTTTGCAGACACCTTAGTTGAAGTAGGTTTAGTTGGTGTTTTAACTTTAGATAACCAAGA 14287

NC_006213.1 GTTTGCGGACAAATTGGTGGAGGTAGGCTTAGTAGGCGTTTTAACACTTGATAATCAAGA 13947

NC_002645.1 TTTTTGCGACGAAATGGTGCTCAAAGGAGTTGTTGGTGTTTTGACCTTAGACAACCAAGA 13109

JX504050.1 TCTATGCGATGCGATGGTTGCTAAAGGTGTTGTTGGTGTTTTAACATTAGATAACCAAGA 13028

* ** * ** * ** ** ** * ** * ** ** ** **

NC_019843.3 CCTTAATGGCAAGTGGTATGATTTTGGTGACTTCGTAATCACTCAACCTGGTTCAGGAGT 14103

NC_004718.3 TCTTAATGGGAACTGGTACGATTTCGGTGATTTCGTACAAGTAGCACCAGGCTGCGGAGT 14062

EPI_ISL_6640916_omicron TCTCAATGGTAACTGGTATGATTTCGGTGATTTCATACAAACCACGCCAGGTAGTGGAGT 14066

MW494315.1 TCTCAATGGTAACTGGTATGATTTCGGTGATTTCATACAAACCACGCCAGGTAGTGGAGT 14132

KF686346.1 TTTGTATGGTCAATGGTATGATTTTGGTGATTTTATACAAACAGCCCCAGGATTTGGTGT 14347

NC_006213.1 TTTAAATGGTAAATGGTATGATTTTGGTGACTATGTTATTGCAGCCCCAGGATGTGGTGT 14007

NC_002645.1 TCTTAATGGGAATTTCTATGACTTCGGTGACTTTGTATTGTGTCCTCCTGGAATGGGAAT 13169

JX504050.1 TCTTAATGGTAACTTTTATGATTTTGGTGATTTTGTTGTTAGCTTACCTAATATGGGTGT 13088

* **** * * ** ** ** ***** * * ** ** *

NC_019843.3 AGCTATAGTTGATAGCTACTATTCTTATTTGATGCCTGTGCTCTCAATGACCGATTGTCT 14163

NC_004718.3 TCCTATTGTGGATTCATATTACTCATTGCTGATGCCCATCCTCACTTTGACTAGGGCATT 14122

EPI_ISL_6640916_omicron TCCTGTTGTAGATTCTTATTATTCATTGTTAATGCCTATATTAACCTTGACCAGGGCTTT 14126

MW494315.1 TCCTGTTGTAGATTCTTATTATTCATTGTTAATGCCTATATTAACCTTGACCAGGGCTTT 14192

KF686346.1 GGCAGTCGCAGATTCTTACTATTCTTATATGATGCCTATGTTGACTATGTGTCATGTATT 14407

NC_006213.1 TGCTATAGCAGATTCTTATTATTCTTATATCATGCCTATGCTGACCATGTGTCATGCATT 14067

NC_002645.1 ACCCTACTGCACGTCATACTATTCTTATATGATGCCTGTTATGGGTATGACTAATTGTTT 13229

JX504050.1 TCCCTGTTGTACATCATATTATTCTTATATGATGCCTATTATGGGTTTAACTAATTGTTT 13148

* ** ** ** * * ***** * * * *

NC_019843.3 GGCCGCTGAGACACATAGGGATTGTGA---TTTTAATAAACCACTCATTGAGTGGCCACT 14220

NC_004718.3 GGCTGCTGAGTCCCATATGGATGCTGA---TCTCGCAAAACCACTTATTAAGTGGGATTT 14179

EPI_ISL_6640916_omicron AACTGCAGAGTCACATGTTGACACTGA---CTTAACAAAGCCTTACATTAAGTGGGATTT 14183

MW494315.1 AACTGCAGAGTCACATGTTGACACTGA---CTTAACAAAGCCTTACATTAAGTGGGATTT 14249

KF686346.1 AGATTGTGAATTATTTGTTAA---------------TGATAGTTATAGACAATTCGATCT 14452

NC_006213.1 GGATTGCGAATTGTATGTGAA---------------TAATGCTTATAGACTATTTGATCT 14112

NC_002645.1 AGCTAGTGAGTGCTTTATGAAAAGTGACATCTTTGGTCAAGACTTCAAAACTTTTGATTT 13289

JX504050.1 AGCTAGTGAGTGTTTTGTCAAGAGTGATATTTTTGGTAGTGATTTTAAAACTTTTGATTT 13208

** * * * * *

NC_019843.3 TACTGAGTATGATTTTACTGATTATAAGGTACAACTCTTTGAGAAGTACTTTAAATATTG 14280

NC_004718.3 GCTGAAATATGATTTTACGGAAGAGAGACTTTGTCTCTTCGACCGTTATTTTAAATATTG 14239

EPI_ISL_6640916_omicron GTTAAAATATGACTTCACGGAAGAGAGGTTAAAACTCTTTGACCGTTATTTTAAATATTG 14243

MW494315.1 GTTAAAATATGACTTCACGGAAGAGAGGTTAAAACTCTTTGACCGTTATTTTAAATATTG 14309

KF686346.1 TGTGCAGTATGATTTTACTGATTACAAGTTAGAGTTGTTTAATAAGTATTTTAAGTATTG 14512

NC_006213.1 TGTACAGTATGATTTTACTGATTACAAGCTTGAATTGTTTAATAAGTATTTTAAGCACTG 14172

NC_002645.1 GTTGAAATATGATTTCACAGAACATAAGGAGGTTTTGTTTAACAAGTACTTTAAGTATTG 13349

JX504050.1 GCTTAAGTATGATTTCACTGAACATAAAGAAAATTTATTCAATAAGTACTTTAAGCATTG 13268

* ***** ** ** ** * * * ** * ** ***** * **

NC_019843.3 GGATCAGACGTATCACGCAAATTGCGTTAATTGTACTGATGACCGTTGTGTGTTACATTG 14340

NC_004718.3 GGACCAGACATACCATCCCAATTGTATTAACTGTTTGGATGATAGGTGTATCCTTCATTG 14299

EPI_ISL_6640916_omicron GGATCAGACATACCACCCAAATTGTGTTAACTGTTTGGATGACAGATGCATTCTGCATTG 14303

MW494315.1 GGATCAGACATACCACCCAAATTGTGTTAACTGTTTGGATGACAGATGCATTCTGCATTG 14369

KF686346.1 GGGTATGAAGTATCATCCTAATACTGTGGATTGTGATAATGATAGGTGTATTATTCATTG 14572

NC_006213.1 GAGTATGCCATATCATCCTAACACTGTTGATTGTCAGGATGATCGGTGTATTATACATTG 14232

NC_002645.1 GGGACAGGATTATCATCCTGATTGTGTTGATTGCCATGACGAGATGTGTATTTTGCATTG 13409

JX504050.1 GAGTTTTGATTATCATCCTAATTGTAGTGACTGTTATGATGATATGTGTGTTATACATTG 13328

* ** ** * * * ** * ** ** * * *****

NC_019843.3 TGCTAATTTCAATGTATTGTTTGCTATGACCATGCCTAAGACTTGTTTCGGACCCATAGT 14400

NC_004718.3 TGCAAACTTTAATGTGTTATTTTCTACTGTGTTTCCACCTACAAGTTTTGGACCACTAGT 14359

EPI_ISL_6640916_omicron TGCAAACTTTAATGTTTTATTCTCTACAGTGTTCCCACTTACAAGTTTTGGACCACTAGT 14363

MW494315.1 TGCAAACTTTAATGTTTTATTCTCTACAGTGTTCCCACCTACAAGTTTTGGACCACTAGT 14429

KF686346.1 TGCTAATTTTAATATACTATTTAGTATGGTTTTACCTAATACTTGTTTTGGTCCCCTTGT 14632

NC_006213.1 TGCTAATTTTAACATACTTTTTAGTATGGTTTTACCTAATACATGTTTTGGGCCTCTTGT 14292

NC_002645.1 TTCAAATTTTAACACACTCTTCGCAACCACAATTCCAAACACGGCTTTTGGACCTCTATG 13469

JX504050.1 TGCTAATTTTAATACACTATTTGCCACAACTATACCAGGTACTGCTTTTGGTCCACTATG 13388

* * ** ** ** * ** * * ** ** *** ** ** *

NC_019843.3 CCGAAAGATCTTTGTTGATGGCGTGCCATTTGTAGTATCTTGTGGTTATCACTACAAAGA 14460

NC_004718.3 AAGAAAAATATTTGTAGATGGTGTTCCTTTTGTTGTTTCAACTGGATACCATTTTCGTGA 14419

EPI_ISL_6640916_omicron GAGAAAAATATTTGTTGATGGTGTTCCATTTGTAGTTTCAACTGGATACCACTTCAGAGA 14423

MW494315.1 GAGAAAAATATTTGTTGATGGTGTTCCATTTGTAGTTTCAACTGGATACCACTTCAGAGA 14489

KF686346.1 TAGACAAATTTTTGTAGATGGTGTACCGTTTGTTGTTTCTATTGGTTACCATTACAAAGA 14692

NC_006213.1 TAGGCAAATTTTTGTGGATGGTGTGCCTTTTGTTGTTTCAATTGGCTACCATTATAAAGA 14352

NC_002645.1 CAGAAAAGTGTTTATTGATGGTGTACCCGTAGTTGCTACTGCTGGTTACCACTTTAAACA 13529

JX504050.1 TCGTAAAGTTTTTATAGATGGTGTTCCACTTGTTACAACTGCTGGTTATCATTTTAAGCA 13448

* * * *** * ***** ** ** * ** * *** ** ** * *

NC_019843.3 ATTAGGTTTAGTCATGAATATGGATGTTAGTCTCCATAGACATAGGCTCTCTCTTAAGGA 14520

NC_004718.3 GTTAGGAGTCGTACATAATCAGGATGTAAACTTACATAGCTCGCGTCTCAGTTTCAAGGA 14479

EPI_ISL_6640916_omicron GCTAGGTGTTGTACATAATCAGGATGTAAACTTACATAGCTCTAGACTTAGTTTTAAGGA 14483

MW494315.1 GCTAGGTGTTGTACATAATCAGGATGTAAACTTACATAGCTCTAGACTTAGTTTTAAGGA 14549

KF686346.1 GTTAGGTGTAGTTATGAACTTAGATGTTGACACACACCGTTATCGTTTGTCTCTTAAAGA 14752

NC_006213.1 ACTTGGTATTGTGATGAATATGGATGTGGATACACATCGTTATCGCTTGTCTTTAAAAGA 14412

NC_002645.1 ATTAGGACTTGTGTGGAACAAAGATGTTAACACTCATTCTACCAGACTTACTATTACTGA 13589

JX504050.1 ATTAGGTTTGGTTTGGAATAAAGATGTTAACACACACTCAGTTAGGTTGACAATTACTGA 13508

* ** * ** ** ***** ** * * * * **

NC_019843.3 GTTGATGATGTATGCCGCTGATCCAGCCATGCACATTGCCTCCTCTAACGCTTTTCTTGA 14580

NC_004718.3 ACTTTTAGTGTATGCTGCTGATCCAGCTATGCATGCAGCTTCTGGCAATTTATTGCTAGA 14539

EPI_ISL_6640916_omicron ATTACTTGTGTATGCTGCTGACCCTGCTATGCACGCTGCTTCTGGTAATCTATTACTAGA 14543

MW494315.1 ATTACTTGTGTATGCTGCTGACCCTGCTATGCACGCTGCTTCTGGTAATCTATTACTAGA 14609

KF686346.1 TTTACTTCTTTATGCAGCAGATCCTGCTATGCATGTTGCATCTGCTAGTGCTCTGCTTGA 14812

NC_006213.1 CTTGCTTTTATATGCTGCTGATCCAGCTTTGCATGTAGCTTCTGCTAGTGCATTGTATGA 14472

NC_002645.1 ACTCTTACAGTTTGTGACAGATCCAACGCTTATAGTTGCGTCATCGCCTGCCTTGGTGGA 13649

JX504050.1 ACTTTTGCAATTTGTCACCGACCCTTCCTTGATAATAGCTTCTTCCCCAGCACTCGTTGA 13568

* * * ** * ** ** * * ** ** * **

NC_019843.3 TTTGAGGACATCATGTTTTAGTGTCGCTGCACTTACAACTGGTTTGACTTTTCAAACTGT 14640

NC_004718.3 TAAACGCACTACATGCTTTTCAGTAGCTGCACTAACAAACAATGTTGCTTTTCAAACTGT 14599

EPI_ISL_6640916_omicron TAAACGCACTACGTGCTTTTCAGTAGCTGCACTTACTAACAATGTTGCTTTTCAAACTGT 14603

MW494315.1 TAAACGCACTACGTGCTTTTCAGTAGCTGCACTTACTAACAATGTTGCTTTTCAAACTGT 14669

KF686346.1 TTTACGAACTTGTTGTTTTAGTGTAGCTGCCATTACAAGTGGTATAAAATTTCAAACTGT 14872

NC_006213.1 TTTACGCACTTGCTGTTTTAGTGTTGCCGCTATAACAAGCGGTGTAAAATTTCAAACAGT 14532

NC_002645.1 TAAACGCACTGTTTGTTTTTCTGTCGCTGCTTTGAGTACAGGATTAACATCCCAAACAGT 13709

JX504050.1 TCAACGCACTATTTGTTTTTCTGTTGCAGCATTGAGTACTGGTTTGACAAATCAAGTTGT 13628

* * ** ** *** ** ** ** * * * * *** **

NC_019843.3 GCGGCCTGGCAATTTTAACCAAGACTTCTATGATTTCGTGGTATCTAAAGGTTTCTTTAA 14700

NC_004718.3 CAAACCCGGTAATTTTAATAAAGACTTTTATGACTTTGCTGTGTCTAAAGGTTTCTTTAA 14659

EPI_ISL_6640916_omicron CAAACCCGGTAATTTTAACAAAGACTTCTATGACTTTGCTGTGTCTAAGGGTTTCTTTAA 14663

MW494315.1 CAAACCCGGTAATTTTAACAAAGACTTCTATGACTTTGCTGTGTCTAAGGGTTTCTTTAA 14729

KF686346.1 AAAACCAGGTAACTTTAACCAAGACTTTTACGAGTTTGTTAAAAGTAAAGGCTTGTTTAA 14932

NC_006213.1 TAAACCTGGTAATTTTAATCAGGATTTTTATGATTTTGTTTTAAGTAAAGGCCTGCTTAA 14592

NC_002645.1 AAAACCTGGCCATTTTAATAAGGAGTTTTATGACTTCTTACGTTCTCAGGGGTTTTTCGA 13769

JX504050.1 TAAGCCAGGTCATTTTAATGAAGAGTTTTATAACTTTCTTCGTTTAAGAGGTTTCTTTGA 13688

** ** * ***** * ** ** ** * ** ** * * *

NC_019843.3 GGAGGGCTCTTCAGTGACGCTCAAACATTTTTTCTTTGCTCAAGATGGTAATGCTGCTAT 14760

NC_004718.3 GGAAGGAAGTTCTGTTGAACTAAAACACTTCTTCTTTGCTCAGGATGGCAACGCTGCTAT 14719

EPI_ISL_6640916_omicron GGAAGGAAGTTCTGTTGAATTAAAACACTTCTTCTTTGCTCAGGATGGTAATGCTGCTAT 14723

MW494315.1 GGAAGGAAGTTCTGTTGAATTAAAACACTTCTTCTTTGCTCAGGATGGTAATGCTGCTAT 14789

KF686346.1 AGAGGGTAGTACAGTTGATTTGAAACATTTTTTCTTTACTCAAGATGGTAATGCTGCAAT 14992

NC_006213.1 AGAGGGTAGCTCAGTTGATCTGAAGCACTTTTTCTTTACACAGGATGGTAATGCTGCTAT 14652

NC_002645.1 TGAGGGTTCAGAATTAACATTGAAGCATTTCTTTTTTACACAAAAGGGTGATGCTGCAAT 13829

JX504050.1 TGAAGGTTCTGAACTTACATTAAAACATTTCTTCTTCGCACAGAATGGTGATGCTGCTGT 13748

** ** * * ** ** ** ** ** * ** * ** * ***** *

NC_019843.3 TACAGATTATAATTACTATTCTTATAATCTGCCTACTATGTGTGACATCAAACAAATGTT 14820

NC_004718.3 CAGTGATTATGACTATTATCGTTATAATCTGCCAACAATGTGTGATATCAGACAACTCCT 14779

EPI_ISL_6640916_omicron CAGCGATTATGACTACTATCGTTATAATCTACCAACAATGTGTGATATCAGACAACTACT 14783

MW494315.1 CAGCGATTATGACTACTATCGTTATAATCTACCAACAATGTGTGATATCAGACAACTACT 14849

KF686346.1 TACTGATTATAATTATTATAAGTATAATTTACCTACTATGGTTGATATTAAGCAGTTATT 15052

NC_006213.1 TACTGATTATAATTATTATAAGTATAATTTGCCCACCATGGTGGACATTAAGCAGTTGTT 14712

NC_002645.1 TAAAGATTTTGATTATTATCGTTACAACAGACCTACTATGCTGGATATTGGACAAGCTCG 13889

JX504050.1 TAAAGATTTTGACTTTTACCGTTATAATAAGCCTACCATTTTAGATATTTGTCAAGCTAG 13808

* **** * * * ** ** ** ** ** ** ** ** **

NC_019843.3 GTTCTGCATGGAAGTTGTAAACAAGTACTTCGAAATCTATGACGGTGGTTGTCTTAATGC 14880

NC_004718.3 ATTCGTAGTTGAAGTTGTTGATAAATACTTTGATTGTTACGATGGTGGCTGTATTAATGC 14839

EPI_ISL_6640916_omicron ATTTGTAGTTGAAGTTGTTGATAAGTACTTTGATTGTTACGATGGTGGCTGTATTAATGC 14843

MW494315.1 ATTTGTAGTTGAAGTTGTTGATAAGTACTTTGATTGTTACGATGGTGGCTGTATTAATGC 14909

KF686346.1 GTTTGTATTAGAAGTTGTTTATAAATATTTTGAAATTTATGATGGTGGTTGTATACCAGC 15112

NC_006213.1 GTTTGTTTTGGAAGTTGTTTATAAGTATTTTGAGATTTATGATGGTGGGTGTATACCGGC 14772

NC_002645.1 CGTAGCATATCAAGTGGCAGCTCGCTATTTTGACTGTTACGAGGGTGGCTGTATTACATC 13949

JX504050.1 AGTTACATATAAGATAGTCTCTCGTTATTTTGACATTTATGAAGGTGGCTGTATTAAGGC 13868

* * * * ** ** ** ** ** ***** *** * *

NC_019843.3 TTCTGAAGTGGTTGTTAATAATTTAGACAAGAGTGCTGGCCATCCTTTTAATAAGTTTGG 14940

NC_004718.3 CAACCAAGTAATCGTTAACAATCTGGATAAATCAGCTGGTTTCCCATTTAATAAATGGGG 14899

EPI_ISL_6640916_omicron TAACCAAGTCATCGTCAACAACCTAGACAAATCAGCTGGTTTTCCATTTAATAAATGGGG 14903

MW494315.1 TAACCAAGTCATCGTCAACAACCTAGACAAATCAGCTGGTTTTCCATTTAATAAATGGGG 14969

KF686346.1 ATCACAAGTTATTGTTAATAATTATGATAAAAGTGCTGGTTATCCATTTAATAAATTTGG 15172

NC_006213.1 ATCACAAGTCATTGTTAATAATTATGATAAGAGTGCTGGCTATCCATTTAACAAATTTGG 14832

NC_002645.1 TAGAGAGGTTGTTGTTACAAACCTTAATAAAAGCGCTGGTTGGCCCCTTAATAAGTTTGG 14009

JX504050.1 ATGTGAAGTTGTTGTAACAAATCTTAATAAGAGTGCTGGTTGGCCATTAAATAAGTTTGG 13928

* ** * ** * ** * ** ***** ** * ** ** * **

NC_019843.3 CAAAGCTCGTGTCTATTATGAGAGCATGTCTTACCAGGAGCAAGATGAACTTTTTGCCAT 15000

NC_004718.3 TAAGGCTAGACTTTATTATGACTCAATGAGTTATGAGGATCAAGATGCACTTTTCGCGTA 14959

EPI_ISL_6640916_omicron TAAGGCTAGACTTTATTATGATTCAATGAGTTATGAGGATCAAGATGCACTTTTCGCATA 14963

MW494315.1 TAAGGCTAGACTTTATTATGATTCAATGAGTTATGAGGATCAAGATGCACTTTTCGCATA 15029

KF686346.1 TAAAGCCAGACTTTATTATGAGGCATTATCATTTGAAGAACAGAATGAAATTTATGCATA 15232

NC_006213.1 AAAAGCCAGGCTCTATTATGAAGCATTATCATTTGAGGAACAGGATGAAATTTACGCTTA 14892

NC_002645.1 TAAAGCTGGTTTATATTATGAGTCTATTAGTTATGAGGAACAAGATGCTATTTTTTCATT 14069

JX504050.1 TAAAGCTAGTTTGTATTATGAATCTATATCTTATGAAGAACAGGATGCTTTGTTTGCTTT 13988

** ** * * ******** * * * ** ** *** * * *

NC_019843.3 GACAAAGCGTAACGTCATTCCTACCATGACTCAAATGAATCTAAAATATGCTATTAGTGC 15060

NC_004718.3 TACTAAGCGTAATGTCATCCCTACTATAACTCAAATGAATCTTAAGTATGCCATTAGTGC 15019

EPI_ISL_6640916_omicron TACAAAACGTAATGTCATCCCTACTATAACTCAAATGAATCTTAAGTATGCCATTAGTGC 15023

MW494315.1 TACAAAACGTAATGTCATCCCTACTATAACTCAAATGAATCTTAAGTATGCCATTAGTGC 15089

KF686346.1 TACTAAACGTAATGTTCTGCCCACCTTAACTCAAATGAATTTAAAATATGCTATCAGTGC 15292

NC_006213.1 TACTAAGCGTAATGTCCTGCCAACACTTACTCAAATGAATTTGAAATATGCTATTAGTGC 14952

NC_002645.1 AACAAAGCGTAATATTCTCCCTACTATGACTCAGTTAAATCTTAAATACGCCATATCTGG 14129

JX504050.1 GACAAAGCGTAATGTCCTCCCTACTATGACACAGCTGAATCTTAAGTATGCTATTAGTGG 14048

** ** ***** * * ** ** * ** ** * *** * ** ** ** ** **

NC_019843.3 TAAGAATAGAGCTCGCACTGTTGCAGGCGTGTCCATACTTAGCACAATGACTAATCGCCA 15120

NC_004718.3 AAAGAATAGAGCTCGCACCGTAGCTGGTGTCTCTATCTGTAGTACTATGACAAATAGACA 15079

EPI_ISL_6640916_omicron AAAGAATAGAGCTCGCACCGTAGCTGGTGTCTCTATCTGTAGTACTATGACCAATAGACA 15083

MW494315.1 AAAGAATAGAGCTCGCACCGTAGCTGGTGTCTCTATCTGTAGTACTATGACCAATAGACA 15149

KF686346.1 TAAGAATAGAGCTCGCACTGTAGCAGGTGTTTCTATTCTTAGTACTATGACAGGCCGAAT 15352

NC_006213.1 TAAGAATAGAGCCCGCACTGTTGCTGGTGTTTCCATACTTAGTACTATGACTGGCAGAAT 15012

NC_002645.1 TAAGGAACGCGCACGTACAGTGGGTGGCGTCTCTTTATTAGCTACTATGACTACAAGACA 14189

JX504050.1 TAAAGAACGTGCTAGAACTGTTGGTGGTGTTTCTCTGTTGTCTACAATGACCACAAGACA 14108

** * * ** * ** ** * ** ** ** * ** ***** *

NC_019843.3 GTACCATCAGAAAATGCTTAAGTCCATGGCTGCAACTCGTGGAGCGACTTGCGTCATTGG 15180

NC_004718.3 GTTTCATCAGAAATTATTGAAGTCAATAGCCGCCACTAGAGGAGCTACTGTGGTAATTGG 15139

EPI_ISL_6640916_omicron GTTTCATCAAAAATTATTGAAATCAATAGCCGCCACTAGAGGAGCTACTGTAGTAATTGG 15143

MW494315.1 GTTTCATCAAAAATTATTGAAATCAATAGCCGCCACTAGAGGAGCTACTGTAGTAATTGG 15209

KF686346.1 GTTCCATCAAAAATGTTTGAAGAGTATAGCAGCTACCCGAGGTGTTCCTGTTGTTATAGG 15412

NC_006213.1 GTTTCATCAAAAATGTTTGAAAAGTATAGCAGCTACACGTGGTGTTCCTGTAGTTATAGG 15072

NC_002645.1 GTTTCATCAGAAATGTCTGAAATCCATAGTAGCTACCAGAAATGCCACCGTTGTTATCGG 14249

JX504050.1 ATACCATCAAAAACATCTTAAATCCATTGTTAATACACGCAATGCCACTGTTGTTATTGG 14168

* ***** *** * ** ** * ** * * * ** ** **

NC_019843.3 TACTACAAAGTTCTACGGTGGCTGGGATTTCATGCTTAAAACATTGTACAAAGATGTTGA 15240

NC_004718.3 AACAAGCAAGTTTTACGGTGGCTGGCATAATATGTTAAAAACTGTTTACAGTGATGTAGA 15199

EPI_ISL_6640916_omicron AACAAGCAAATTCTATGGTGGTTGGCACAATATGTTAAAAACTGTTTATAGTGATGTAGA 15203

MW494315.1 AACAAGCAAATTCTATGGTGGTTGGCACAACATGTTAAAAACTGTTTATAGTGATGTAGA 15269

KF686346.1 AACCACTAAATTTTATGGTGGTTGGGACGATATGTTACGTCATCTTATAAAGGATGTTGA 15472

NC_006213.1 CACCACTAAATTTTATGGTGGCTGGGATGATATGTTACGCCGCCTTATTAAAGATGTTGA 15132

NC_002645.1 CACTACCAAGTTTTATGGCGGGTGGGATAATATGTTAAAGAACCTGATGGCCGATGTTGA 14309

JX504050.1 TACTACCAAATTTTATGGTGGTTGGAATAATATGTTGCGTACTTTAATTGATGGTGTTGA 14228

** * ** ** ** ** ** *** * *** * * * *** **

NC_019843.3 TAATCCGCATCTTATGGGTTGGGATTACCCTAAGTGTGATAGAGCTATGCCTAATATGTG 15300

NC_004718.3 AACTCCACACCTTATGGGTTGGGATTATCCAAAATGTGACAGAGCCATGCCTAACATGCT 15259

EPI_ISL_6640916_omicron AAACCCTCACCTTATGGGTTGGGATTATCCTAAATGTGATAGAGCCATGCCTAACATGCT 15263

MW494315.1 AAACCCTCACCTTATGGGTTGGGATTATCCTAAATGTGATAGAGCCATGCCTAACATGCT 15329

KF686346.1 CAACCCTGTTCTTATGGGTTGGGATTATCCTAAATGTGATCGTGCTATGCCAAATATTTT 15532

NC_006213.1 CAATCCTGTACTTATGGGTTGGGATTATCCTAAGTGTGATCGTGCTATGCCAAACCTACT 15192

NC_002645.1 TGATCCTAAATTGATGGGATGGGACTATCCTAAGTGTGATAGAGCTATGCCCTCAATGAT 14369

JX504050.1 AAACCCTATGCTTATGGGTTGGGATTATCCCAAATGTGATAGAGCTTTGCCTAACATGAT 14288

** * ***** ***** ** ** ** ***** * ** **** *

NC_019843.3 TAGAATCTTCGCTTCACTCATATTAGCTCGTAAACATGGCACTTGTTGTACTACAAGGGA 15360

NC_004718.3 TAGGATAATGGCCTCTCTTGTTCTTGCTCGCAAACATAACACTTGCTGTAACTTATCACA 15319

EPI_ISL_6640916_omicron TAGAATTATGGCCTCACTTGTTCTTGCTCGCAAACATACAACGTGTTGTAGCTTGTCACA 15323

MW494315.1 TAGAATTATGGCCTCACTTGTTCTTGCTCGCAAACATACAACGTGTTGTAGCTTGTCACA 15389

KF686346.1 GCGTATTGTTAGTAGTTTAGTTTTGGCCCGCAAACATGAATTTTGTTGTTCACATGGTGA 15592

NC_006213.1 ACGTATTGTTAGTAGTTTGGTATTAGCCCGAAAACATGAGACATGTTGTTCGCAAAGCGA 15252

NC_002645.1 TCGTATGTTGTCGGCTATGATCTTAGGTTCTAAGCATGTCACATGTTGTACGGCTAGTGA 14429

JX504050.1 ACGTATGATTTCAGCCATGGTGTTGGGCTCTAAGCATGTTAATTGTTGTACTGCAACAGA 14348

* ** * * * * * ** *** ** *** *

NC_019843.3 CAGATTTTATCGCTTGGCAAATGAGTGTGCTCAGGTGCTAAGCGAATATGTTCTATGTGG 15420

NC_004718.3 CCGTTTCTACAGGTTAGCTAACGAGTGTGCGCAAGTATTAAGTGAGATGGTCATGTGTGG 15379

EPI_ISL_6640916_omicron CCGTTTCTATAGATTAGCTAATGAGTGTGCTCAAGTATTGAGTGAAATGGTCATGTGTGG 15383

MW494315.1 CCGTTTCTATAGATTAGCTAATGAGTGTGCTCAAGTATTGAGTGAAATGGTCATGTGTGG 15449

KF686346.1 TAGATTTTATCGCCTTGCGAATGAATGTGCTCAAGTTTTGAGTGAAATAGTTATGTGTGG 15652

NC_006213.1 TAGGTTTTATCGACTTGCGAATGAATGCGCACAAGTTTTGAGTGAAATTGTTATGTGTGG 15312

NC_002645.1 TAAATTTTATAGACTTAGTAATGAGCTTGCTCAAGTTTTGACCGAGGTTGTTTATTCAAA 14489

JX504050.1 TAGGTTTTATAGGCTTGGTAATGAGTTGGCACAAGTTTTAACAGAAGTTGTTTATTCTAA 14408

** ** * * ** ** ** ** ** * * ** ** *

NC_019843.3 TGGTGGTTACTACGTCAAACCTGGAGGTACCAGTAGCGGAGATGCCACCACTGCATATGC 15480

NC_004718.3 CGGCTCACTATATGTTAAACCAGGTGGAACATCATCCGGTGATGCTACAACTGCTTATGC 15439

EPI_ISL_6640916_omicron CGGTTCACTATATGTTAAACCAGGTGGAACCTCATCAGGAGATGCCACAACTGCTTATGC 15443

MW494315.1 CGGTTCACTATATGTTAAACCAGGTGGAACCTCATCAGGAGATGCCACAACTGCTTATGC 15509

KF686346.1 CGGTTGCTATTATGTTAAGCCTGGTGGTACTAGCAGTGGTGATGCAACTACTGCTTTTGC 15712

NC_006213.1 TGGCTGTTATTATGTTAAGCCTGGTGGCACTAGTAGTGGTGATGCAACTACTGCTTTTGC 15372

NC_002645.1 TGGTGGGTTTTATTTTAAACCTGGTGGTACAACTTCTGGTGATGCAACTACAGCCTACGC 14549

JX504050.1 TGGTGGTTTTTATTTTAAGCCAGGTGGTACGACTTCTGGTGACGCTAGTACAGCTTATGC 14468

** ** * ** ** ** ** ** ** ** ** * ** ** * **

NC_019843.3 CAATAGTGTCTTTAACATTTTGCAGGCGACAACTGCTAATGTCAGTGCACTTATGGGTGC 15540

NC_004718.3 TAATAGTGTCTTTAACATTTGTCAAGCTGTTACAGCCAATGTAAATGCACTTCTTTCAAC 15499

EPI_ISL_6640916_omicron TAATAGTGTTTTTAACATTTGTCAAGCTGTCACGGCCAATGTTAATGCACTTTTATCTAC 15503

MW494315.1 TAATAGTGTTTTTAACATTTGTCAAGCTGTCACGGCCAATGTTAATGCACTTTTATCTAC 15569

KF686346.1 TAATTCTGTTTTTAATATATGTCAGGCTGTTACTGCTAACGTTTGTTCTCTTATGGCCTG 15772

NC_006213.1 TAATTCAGTCTTTAACATATGTCAAGCTGTTTCAGCCAATGTATGTGCCTTAATGTCATG 15432

NC_002645.1 CAATTCTGTCTTTAATATATTTCAGGCTGTAAGTTCTAACATTAATTGCGTTTTGAGCGT 14609

JX504050.1 TAATTCTATTTTTAACATTTTTCAAGCCGTGAGTTCTAACATTAACAGGTTGCTTAGTGT 14528

*** * ***** ** * ** ** * ** * * *

NC_019843.3 TAATGGCAACAAGATTGTTGACAAAGAAGTTAAAGACATGCAGTTTGATTTGTATGTCAA 15600

NC_004718.3 TGATGGTAATAAGATAGCTGACAAGTATGTCCGCAATCTACAACACAGGCTCTATGAGTG 15559

EPI_ISL_6640916_omicron TGATGGTAACAAAATTGCCGATAAGTATGTCCGCAATTTACAACACAGACTTTATGAGTG 15563

MW494315.1 TGATGGTAACAAAATTGCCGATAAGTATGTCCGCAATTTACAACACAGACTTTATGAGTG 15629

KF686346.1 TAATGGCCATAAGATTGAAGATTTAAGTATACGCAATTTACAAAAACGCTTATACTCTAA 15832

NC_006213.1 CAATGGCAATAAGATTGAAGATCTTAGTATACGTGCTCTTCAGAAGCGCTTATACTCACA 15492

NC_002645.1 TAACTCGTCAAATTGCAATAATTTTAATGTTAAGAAGTTACAGAGACAACTTTATGATAA 14669

JX504050.1 CCCATCAGATTCATGTAATAATGTTAATGTTAGGGATTTACAACGACGTCTGTATGATAA 14588

* * * ** * **

NC_019843.3 TGTTTACAGGAGCACTAGCCCAGACCCCAAATTTGTTGATAAATACTATGCTTTTCTTAA 15660

NC_004718.3 TCTCTATAGAAATAGGGATGTTGATCATGAATTCGTGGATGAGTTTTACGCTTACCTGCG 15619

EPI_ISL_6640916_omicron TCTCTATAGAAATAGAGATGTTGACACAGACTTTGTGAATGAGTTTTACGCATATTTGCG 15623

MW494315.1 TCTCTATAGAAATAGAGATGTTGACACAGACTTTGTGAATGAGTTTTACGCATATTTGCG 15689

KF686346.1 TGTTTATCGTACAGATTATGTTGATTATACATTTGTTAATGAGTATTATGAATTTTTATG 15892

NC_006213.1 TGTGTATAGAAGTGATAAGGTTGATTCAACCTTTGTCACAGAATATTATGAATTTTTAAA 15552

NC_002645.1 TTGCTATAGAAATAGTAATGTTGATGAATCTTTTGTGGATGACTTTTATGGTTATTTGCA 14729

JX504050.1 TTGTTATAGGTTAACTAGTGTTGAAGAGTCATTCATTGATGATTATTATGGTTATCTTAG 14648

* ** * ** ** * * * ** * * *

NC_019843.3 TAAGCACTTTTCTATGATGATACTGTCTGATGACGGTGTCGTTTGCTATAATAGTGATTA 15720

NC_004718.3 TAAACATTTCTCCATGATGATTCTTTCTGATGATGCCGTTGTGTGCTATAACAGTAACTA 15679

EPI_ISL_6640916_omicron TAAACATTTCTCAATGATGATACTCTCTGACGATGCTGTTGTGTGTTTCAATAGCACTTA 15683

MW494315.1 TAAACATTTCTCAATGATGATACTCTCTGACGATGCTGTTGTGTGTTTCAATAGCACTTA 15749

KF686346.1 TAAGCATTTTAGTATGATGATTTTGAGTGATGATGGTGTTGTCTGTTATAACTCTGATTA 15952

NC_006213.1 TAAGCATTTTAGTATGATGATTTTGAGTGATGATGGGGTTGTGTGTTATAATTCTGATTA 15612

NC_002645.1 AAAGCATTTTTCTATGATGATTCTTTCTGATGATAGTGTTGTGTGCTATAATAAAACTTA 14789

JX504050.1 GAAACATTTTTCAATGATGATTCTCTCTGATGACGGTGTTGTCTGTTATAACAAGGATTA 14708

** ** ** ******** * *** ** ** ** ** * ** **

NC_019843.3 TGCAGCTAAGGGTTACATTGCTGGAATACAGAATTTTAAGGAAACGCTGTATTATCAGAA 15780

NC_004718.3 TGCGGCTCAAGGTTTAGTAGCTAGCATTAAGAACTTTAAGGCAGTTCTTTATTATCAAAA 15739

EPI_ISL_6640916_omicron TGCATCTCAAGGTCTAGTGGCTAGCATAAAGAACTTTAAGTCAGTTCTTTATTATCAAAA 15743

MW494315.1 TGCATCTCAAGGTCTAGTGGCTAGCATAAAGAACTTTAAGTCAGTTCTTTATTATCAAAA 15809

KF686346.1 TGCTAGTAAGGGTTATATAGCTAATATAAGTGTTTTTCAACAAGTTTTGTACTATCAGAA 16012

NC_006213.1 TGCGTCCAAAGGGTATATTGCTAATATAAGTGCCTTTCAACAGGTATTATATTATCAAAA 15672

NC_002645.1 TGCTGGACTTGGTTACATTGCTGATATTAGTGCTTTTAAAGCCACTTTGTATTATCAGAA 14849

JX504050.1 TGCTGAGTTAGGTTATATAGCAGACATTAGTGCTTTTAAAGCCACTTTGTATTACCAGAA 14768

*** ** * ** ** *** * * ** ** ** **

NC_019843.3 CAATGTCTTTATGTCTGAAGCTAAATGCTGGGTGGAAACCGATCTGAAGAAAGGGCCACA 15840

NC_004718.3 TAATGTGTTCATGTCTGAGGCAAAATGTTGGACTGAGACTGACCTTACTAAAGGACCTCA 15799

EPI_ISL_6640916_omicron CAATGTTTTTATGTCTGAAGCAAAATGTTGGACTGAGACTGACCTTACTAAAGGACCTCA 15803

MW494315.1 CAATGTTTTTATGTCTGAAGCAAAATGTTGGACTGAGACTGACCTTACTAAAGGACCTCA 15869

KF686346.1 TAATGTTTTTATGTCTGAATCTAAATGTTGGGTTGAAAATGATATTACTAATGGTCCTCA 16072

NC_006213.1 TAACGTTTTTATGTCAGAATCCAAATGTTGGGTTGAACATGACATAAATAATGGACCTCA 15732

NC_002645.1 TGGTGTGTTTATGAGTACAGCTAAGTGTTGGACTGAGGAAGATCTTTCTATAGGACCTCA 14909

JX504050.1 TAATGTCTTTATGAGTACTTCTAAATGTTGGGTTGAAGAAGATTTAACTAAGGGACCACA 14828

** ** *** * ** ** *** ** ** * * ** ** **

NC_019843.3 TGAATTCTGTTCACAGCATACGCTTTATATTAAGGATGGCGACGATGGTTACTTCCTTCC 15900

NC_004718.3 CGAATTTTGCTCACAGCATACAATGCTAGTTAAACAAGGAGATGATTACGTGTACCTGCC 15859

EPI_ISL_6640916_omicron TGAATTTTGCTCTCAACATACAATGCTAGTTAAACAGGGTGATGATTATGTGTACCTTCC 15863

MW494315.1 TGAATTTTGCTCTCAACATACAATGCTAGTTAAACAGGGTGATGATTATGTGTACCTTCC 15929

KF686346.1 TGAATTTTGTTCCCAACATACTATGTTGGTTAAGATAGATGGTGATTATGTTTATTTACC 16132

NC_006213.1 TGAATTCTGTTCACAACACACAATGCTTGTAAAGATGGATGGTGACGATGTCTACCTTCC 15792

NC_002645.1 TGAATTTTGCTCACAGCACACTATGCAGATTGTAGATGAAAATGGTAAGTATTATCTACC 14969

JX504050.1 TGAGTTTTGTTCCCAGCATACTATGCAAATAGTTGACAAAGATGGTACCTATTATTTGCC 14888

** ** ** ** ** ** ** * * * * * **

NC_019843.3 TTATCCAGACCCTTCAAGAATTTTGTCTGCCGGTTGCTTTGTAGATGATATCGTTAAGAC 15960

NC_004718.3 TTACCCAGATCCATCAAGAATATTAGGCGCAGGCTGTTTTGTCGATGATATTGTCAAAAC 15919

EPI_ISL_6640916_omicron TTACCCAGATCCATCAAGAATCCTAGGGGCCGGCTGTTTTGTAGATGATATCGTAAAAAC 15923

MW494315.1 TTACCCAGATCCATCAAGAATCCTAGGGGCCGGCTGTTTTGTAGATGATATCGTAAAAAC 15989

KF686346.1 ATATCCAGATCCTTCTAGAATTCTAGGAGCTGGTTGTTTTGTTGATGATTTATTGAAGAC 16192

NC_006213.1 ATATCCTAATCCTAGTCGTATATTAGGAGCTGGATGTTTTGTAGATGATTTGTTAAAGAC 15852

NC_002645.1 ATATCCAGATCCTAGCCGTATTATTTCTGCTGGTGTTTTTGTGGATGACATCACTAAGAC 15029

JX504050.1 TTACCCAGATCCTAGTAGGATCTTGTCAGCTGGTGTTTTTGTTGATGATGTTGTTAAGAC 14948

** ** * ** * ** * ** ** ***** ***** * ** **

NC_019843.3 TGACGGTACACTCATGGTAGAGCGGTTTGTGTCTTTGGCTATAGATGCTTACCCTCTCAC 16020

NC_004718.3 AGATGGTACACTTATGATTGAAAGGTTCGTGTCACTGGCTATTGATGCTTACCCACTTAC 15979

EPI_ISL_6640916_omicron AGATGGTACACTTATGATTGAACGGTTCGTGTCTTTAGCTATAGATGCTTACCCACTTAC 15983

MW494315.1 AGATGGTACACTTATGATTGAACGGTTCGTGTCTTTAGCTATAGATGCTTACCCACTTAC 16049

KF686346.1 TGACAGTGTTCTTTTGATAGAGCGCTTTGTAAGTCTAGCTATAGATGCTTACCCTTTAGT 16252

NC_006213.1 TGATAGTGTTCTTTTAATAGAACGATTTGTAAGTCTTGCAATAGATGCTTATCCACTTGT 15912

NC_002645.1 TGATGCTGTCATTCTTTTGGAACGCTATGTTTCTCTGGCTATAGATGCCTACCCATTGTC 15089

JX504050.1 AGATGCTGTTGTTTTGTTAGAACGTTATGTGTCTTTAGCTATTGATGCATACCCTCTTTC 15008

** * * * * ** * * ** * ** ** ***** ** ** *

NC_019843.3 AAAGCATGAAGATATAGAATACCAGAATGTATTCTGGGTCTACTTACAGTATATAGAAAA 16080

NC_004718.3 AAAACATCCTAATCAGGAGTATGCTGATGTCTTTCACTTGTATTTACAATACATTAGAAA 16039

EPI_ISL_6640916_omicron TAAACATCCTAATCAGGAGTATGCTGATGTCTTTCATTTGTACTTACAATACATAAGAAA 16043

MW494315.1 TAAACATCCTAATCAGGAGTATGCTGATGTCTTTCATTTGTACTTACAATACATAAGAAA 16109

KF686346.1 ATATCATGAAAATGAAGAATACCAAAAAGTCTTTCGTGTATATTTAGAATATATAAAAAA 16312

NC_006213.1 GTATCATGAAAATGAAGAATACCAAAAGGTTTTTCGTGTTTATTTGGCGTATATAAAGAA 15972

NC_002645.1 TAAGCATCCTAAACCTGAGTACAGGAAGGTGTTTTACGCATTGTTAGACTGGGTCAAACA 15149

JX504050.1 AAAACACCCTAATTCCGAATATCGTAAGGTTTTTTACGTATTACTTGATTGGGTTAAGCA 15068

* ** * ** ** * ** ** * * * * *

NC_019843.3 ACTGTATAAAGACCTTACAGGACACATGCTTGACAGTTATTCTGTCATGCTATGTGGTGA 16140

NC_004718.3 GTTACATGATGAGCTTACTGGCCACATGTTGGACATGTATTCCGTAATGCTAACTAATGA 16099

EPI_ISL_6640916_omicron GCTACATGATGAGTTAACAGGACACATGTTAGACATGTATTCTGTTATGCTTACTAATGA 16103

MW494315.1 GCTACATGATGAGTTAACAGGACACATGTTAGACATGTATTCTGTTATGCTTACTAATGA 16169

KF686346.1 ACTGTATAATGATCTTGGTACTCAGATCTTAGATAGTTATAGTGTTATTTTAAGTACTTG 16372

NC_006213.1 GTTGTACAATGACCTGGGTAATCAGATCTTGGATAGCTACAGTGTTATTTTAAGTACTTG 16032

NC_002645.1 TCTCAACAAGACTCTTAACGAAGGTGTTTTGGAGTCTTTTTCTGTTACACTTTTAGATGA 15209

JX504050.1 TCTTAACAAAAATTTGAATGAGGGTGTTCTTGAATCTTTTTCTGTTACACTTCTTGATAA 15128

* * * * * * ** * ** * * *

NC_019843.3 TAATTCTGCTAAGTTTTGGGAAGAGGCATTCTATAGAGATCTCTATAGTTCGCCTACCAC 16200

NC_004718.3 TAACACCTCACGGTACTGGGAACCTGAGTTTTATGAGGCTATGTACACACCACATACAGT 16159

EPI_ISL_6640916_omicron TAACACTTCAAGGTATTGGGAACCTGAGTTTTATGAGGCTATGTACACACCGCATACAGT 16163

MW494315.1 TAACACTTCAAGGTATTGGGAACCTGAGTTTTATGAGGCTATGTACACACCGCATACAGT 16229

KF686346.1 TGATGGTTTAAAGTTTACTGAAGAATCATTTTACAAGAATATGTATTTAAAAAGTGCCGT 16432

NC_006213.1 TGATGGACAAAAGTTCACTGATGAGTCCTTTTACAAGAACATGTATTTAAGAAGTGCAGT 16092

NC_002645.1 ACATGAGTCTAAGTTTTGGGATGAAAGCTTTTATGCTAGTATGTATGAGAAGTCTACAGT 15269

JX504050.1 TCAAGAAGATAAGTTTTGGTGTGAAGATTTTTATGCTAGTATGTATGAAAATTCTACAAT 15188

* ** ** ** * ** * *

NC_019843.3 TTTGCAGGCTGTCGGTTCATGCGTTGTATGCCATTCACAGACTTCCCTACGCTGTGGGAC 16260

NC_004718.3 CTTGCAGGCTGTAGGTGCTTGTGTATTGTGCAATTCACAGACTTCACTTCGTTGCGGTGC 16219

EPI_ISL_6640916_omicron CTTACAGGCTGTTGGGGCTTGTGTTCTTTGCAATTCACAGACTTCATTAAGATGTGGTGC 16223

MW494315.1 CTTACAGGCTGTTGGGGCTTGTGTTCTTTGCAATTCACAGACTTCATTAAGATGTGGTGC 16289

KF686346.1 GATGCAGAGTGTAGGTGCATGTGTTGTTTGTTCATCACAAACTTCTTTGCGTTGTGGCAG 16492

NC_006213.1 TATGCAGAGTGTTGGAGCTTGCGTGGTCTGCTCTTCTCAAACATCATTACGTTGTGGCAG 16152

NC_002645.1 ATTACAAGCTGCTGGTCTTTGTGTAGTATGTGGTTCTCAAACAGTTCTAAGATGCGGTGA 15329

JX504050.1 ATTGCAAGCTGCTGGTTTATGTGTTGTTTGTGGTTCACAAACTGTACTTCGTTGTGGTGA 15248

* ** ** ** ** ** * ** ** ** ** * * ** **

NC_019843.3 ATGCATCCGTAGACCATTTCTCTGCTGTAAATGCTGCTATGATCATGTTATAGCAACTCC 16320

NC_004718.3 CTGTATTAGGAGACCATTCCTATGTTGCAAGTGCTGCTATGACCATGTCATTTCAACATC 16279

EPI_ISL_6640916_omicron TTGCATACGTAGACCATTCTTATGTTGTAAATGCTGTTACGACCATGTCATATCAACATC 16283

MW494315.1 TTGCATACGTAGACCATTCTTATGTTGTAAATGCTGTTACGACCATGTCATATCAACATC 16349

KF686346.1 TTGTATACGTAAGCCTTTGTTATGTTGTAAATGTTGTTATGACCATGTTATGGCAACTAA 16552

NC_006213.1 TTGCATCAGAAAGCCTCTTCTTTGCTGCAAGTGTTGTTATGATCATGTTATGGCGACTGA 16212

NC_002645.1 TTGTTTACGCAGACCGATGTTGTGCACTAAGTGCGCCTATGATCATGTGTTTGGCACTGA 15389

JX504050.1 TTGTCTGCGTAAGCCTATGTTGTGCACTAAATGCGCATATGATCATGTATTTGGTACCGA 15308

** * * * ** * * ** ** ** ** ** ***** * **

NC_019843.3 ACATAAGATGGTTTTGTCTGTTTCTCCTTACGTTTGTAATGCCCCTGGTTGTGGCGTTTC 16380

NC_004718.3 ACACAAATTAGTGTTGTCTGTTAATCCCTATGTTTGCAATGCCCCAGGTTGTGATGTCAC 16339

EPI_ISL_6640916_omicron ACATAAATTAGTCTTGTCTGTTAATCCGTATGTTTGCAATGCTCCAGGTTGTGATGTCAC 16343

MW494315.1 ACATAAATTAGTCTTGTCTGTTAATCCGTATGTTTGCAATGCTCCAGGTTGTGATGTCAC 16409

KF686346.1 TCATAAATATGTTTTGAGTGTCTCACCTTACGTTTGTAATGCACCTAACTGTGATGTGAG 16612

NC_006213.1 TCATAAATATGTCTTGAGTGTTTCACCATATGTGTGTAATGCACCAGGATGTGATGTAAA 16272

NC_002645.1 TCATAAGTTCATTTTAGCTATTACACCATATGTGTGTAACACATCTGGCTGCAATGTAAA 15449

JX504050.1 CCACAAGTTTATTTTGGCTATAACACCGTATGTATGTAATGCATCAGGTTGTGGTGTTAG 15368

** ** * ** * * ** ** ** ** ** * * ** **

NC_019843.3 AGACGTTACTAAGCTATATTTAGGTGGTATGAGCTACTTTTGTGTAGATCATAGACCTGT 16440

NC_004718.3 TGATGTGACACAACTGTATCTAGGAGGTATGAGCTATTATTGCAAGTCACATAAGCCTCC 16399

EPI_ISL_6640916_omicron AGATGTGACTCAACTTTACTTAGGAGGTATGAGCTATTATTGTAAATCACATAAACCACC 16403

MW494315.1 AGATGTGACTCAACTTTACTTAGGAGGTATGAGCTATTATTGTAAATCACATAAACCACC 16469

KF686346.1 TGATGTCACCAAATTATATTTGGGTGGTATGTCTTACTATTGTGAAAACCATAAACCCCA 16672

NC_006213.1 TGATGTTACCAAATTGTATCTAGGTGGTATGTCATATTATTGTGAAGACCATAAGCCACA 16332

NC_002645.1 TGACGTTACAAAACTGTATCTTGGAGGTTTGAATTATTACTGTGTAGACCACAAACCACA 15509

JX504050.1 TGATGTCAAAAAATTGTATCTTGGTGGTTTGAATTACTATTGTACAAATCATAAACCACA 15428

** ** * * * ** * ** *** ** ** * ** ** * **

NC_019843.3 GTGTAGTTTTCCACTTTGCGCTAATGGTCTTGTATTCGGCTTATACAAGAATATGTGCAC 16500

NC_004718.3 CATTAGTTTTCCATTATGTGCTAATGGTCAGGTTTTTGGTTTATACAAAAACACATGTGT 16459

EPI_ISL_6640916_omicron CATTAGTTTTCCATTGTGTGCTAATGGACAAGTTTTTGGTTTATATAAAAATACATGTGT 16463

MW494315.1 CATTAGTTTTCCATTGTGTGCTAATGGACAAGTTTTTGGTTTATATAAAAATACATGTGT 16529

KF686346.1 TTATTCATTTAAGTTAGTTATGAATGGTATGGTCTTTGGTTTGTATAAACAATCTTGTAC 16732

NC_006213.1 ATATTCATTCAAGTTGGTAATGAATGGTCTGGTTTTTGGTCTATATAAACAATCTTGTAC 16392

NC_002645.1 TCTTTCATTCCCACTGTGTTCAGCTGGTAATGTCTTTGGTTTGTACAAAAGTTCTGCTTT 15569

JX504050.1 GTTGTCTTTTCCATTATGTTCAGCTGGTAATATATTTGGTTTATATAAAAATTCAGCAAC 15488

** * *** * ** ** * ** **

NC_019843.3 AGGTAGTCCTTCTATAGTTGAATTTAATAGGTTGGCTACCTGTGACTGGACTGAAAGTGG 16560

NC_004718.3 AGGCAGTGACAATGTCACTGACTTCAATGCGATAGCAACATGTGATTGGACTAATGCTGG 16519

EPI_ISL_6640916_omicron TGGTAGCGATAATGTTACTGACTTTAATGCAATTGCAACATGTGACTGGACAAATGCTGG 16523

MW494315.1 TGGTAGCGATAATGTTACTGACTTTAATGCAATTGCAACATGTGACTGGACAAATGCTGG 16589

KF686346.1 GGGTTCACCTTATATAGATGATTTTAATAAGATAGCTAGTTGTAAATGGACAGAAGTTGA 16792

NC_006213.1 AGGATCTCCGTACATAGACGATTTTAATCGTATAGCTAGTTGTAAATGGACCGATGTGGA 16452

NC_002645.1 GGGTTCCATGGACATTGATGTCTTTAACAAACTTTCTACCTCTGATTGGTCTGACATTCG 15629

JX504050.1 TGGTTCCTTAGATGTTGAAGTTTTTAATAGGCTTGCAACGTCTGATTGGACTGATGTTAG 15548

** * * ** ** * * * * * * *** * *

NC_019843.3 TGATTACACCCTTGCCAATACTACAACAGAACCACTCAAACTTTTTGCTGCTGAGACTTT 16620

NC_004718.3 CGATTACATACTTGCCAACACTTGTACTGAGAGACTCAAGCTTTTCGCAGCAGAAACGCT 16579

EPI_ISL_6640916_omicron TGATTACATTTTAGCTAACACCTGTACTGAAAGACTCAAGCTTTTTGCAGCAGAAACGCT 16583

MW494315.1 TGATTACATTTTAGCTAACACCTGTACTGAAAGACTCAAGCTTTTTGCAGCAGAAACGCT 16649

KF686346.1 TGATTATGTTCTGGCAAATGAGTGTATTGAACGTTTAAAGTTATTTGCTGCAGAAACTCA 16852

NC_006213.1 TGATTACATACTAGCTAATGAATGTACAGAGCGCTTGAAATTGTTTGCTGCAGAAACGCA 16512

NC_002645.1 CGACTACAAGCTTGCTAATGATGCAAAAGAGTCACTAAGGTTGTTTGCAGCTGAAACGGT 15689

JX504050.1 GGACTATAAACTTGCTAATGATGTTAAAGATACACTTAGACTCTTTGCGGCTGAAACTAT 15608

** ** * ** ** * ** * * * ** ** ** ** **

NC_019843.3 ACGTGCCACTGAAGAGGCGTCTAAGCAGTCTTATGCTATTGCCACCATCAAAGAAATTGT 16680

NC_004718.3 CAAAGCCACTGAGGAAACATTTAAGCTGTCATATGGTATTGCCACTGTACGCGAAGTACT 16639

EPI_ISL_6640916_omicron CAAAGCTACTGAGGAGACATTTAAACTGTCTTATGGTATTGCTACTGTACGTGAAGTGCT 16643

MW494315.1 CAAAGCTACTGAGGAGACATTTAAACTGTCTTATGGTATTGCTACTGTACGTGAAGTGCT 16709

KF686346.1 AAAGGCAACTGAAGAGGCTTTTAAACAAAGCTATGCTTCTGCTACCATTCAAGAGATTGT 16912

NC_006213.1 AAAGGCAACCGAGGAAGCCTTTAAGCAGAGTTATGCATCAGCAACAATACAAGAGATTGT 16572

NC_002645.1 CAAGGCTAAAGAGGAAAGTGTTAAGTCATCATACGCTTATGCTACCCTAAAGGAGATTGT 15749

JX504050.1 TAAAGCTAAAGAAGAGAGTGTTAAGTCTTCTTATGCTTTTGCAACTCTTAAAGAGGTTGT 15668

** * ** ** *** ** * ** ** * ** * *

NC_019843.3 TGGTGAGCGCCAACTATTACTTGTGTGGGAGGCTGGCAAGTCCAAACCACCACTCAATCG 16740

NC_004718.3 CTCTGACAGAGAATTGCATCTTTCATGGGAGGTTGGAAAACCTAGACCACCATTGAACAG 16699

EPI_ISL_6640916_omicron GTCTGACAGAGAATTACATCTTTCATGGGAAGTTGGTAAACCTAGACCACCACTTAACCG 16703

MW494315.1 GTCTGACAGAGAATTACATCTTTCATGGGAAGTTGGTAAACCTAGACCACCACTTAACCG 16769

KF686346.1 TAGTGATAGAGAAGTTATTTTGTGTTGGGAGACAGGTAAAGTTAAACCACCACTTAATAA 16972

NC_006213.1 TAGTGAGCGCGAATTGATTCTCTCTTGGGAGATTGGAAAAGTTAAGCCACCACTTAATAA 16632

NC_002645.1 AGGTCCTAAGGAACTTTTGCTCTTATGGGAAAGTGGAAAAGCCAAACCACCGTTAAACCG 15809

JX504050.1 TGGACCTAAAGAATTGCTTCTTAGTTGGGAAAGTGGTAAAGTTAAACCACCTTTGAATCG 15728

** * * ***** ** ** * ***** * **

NC_019843.3 TAATTATGTTTTTACTGGTTATCATATAACCAAAAATAGTAAAGTGCAGCTCGGTGAGTA 16800

NC_004718.3 AAACTATGTCTTTACTGGTTACCGTGTAACTAAAAATAGTAAAGTACAGATTGGAGAGTA 16759

EPI_ISL_6640916_omicron AAATTATGTCTTTACTGGTTATCGTGTAACTAAAAACAGTAAAGTACAAATAGGAGAGTA 16763

MW494315.1 AAATTATGTCTTTACTGGTTATCGTGTAACTAAAAACAGTAAAGTACAAATAGGAGAGTA 16829

KF686346.1 AAATTATGTTTTCACAGGCTACCATTTTACTAGTACTGGTAAGACAGTTTTAGGTGAGTA 17032

NC_006213.1 AAATTATGTTTTTACTGGCTACCATTTTACTAAAAATGGTAAGACAGTTTTAGGTGAGTA 16692

NC_002645.1 TAATTCTGTTTTTACATGCTTCCAAATTACAAAAGACTCCAAGTTTCAAGTTGGTGAGTT 15869

JX504050.1 TAATTCTGTTTTCACTTGTTTTCAAATAAGTAAGGACTCAAAATTCCAAATAGGTGAGTT 15788

** * *** ** ** * * * * * * ** * ** ****

NC_019843.3 CATTTTCGAGCGCATTGATTATA---GTGATGCTGTATCCTACAAGTCTAGTACAACGTA 16857

NC_004718.3 CACCTTTGAAAAAGGTGACTATG---GTGATGCTGTTGTGTACAGAGGTACTACGACATA 16816

EPI_ISL_6640916_omicron CACCTTTGAAAAAGGTGACTATG---GTGATGCTGTTGTTTACCGAGGTACAACAACTTA 16820

MW494315.1 CACCTTTGAAAAAGGTGACTATG---GTGATGCTGTTGTTTACCGAGGTACAACAACTTA 16886

KF686346.1 TGTTTTTGATAAAAGTGAATTAA---CTAACGGTGTGTATTACCGCGCTACAACTACTTA 17089

NC_006213.1 TGTTTTTGATAAGAGTGAGTTGA---CTAATGGTGTGTATTATCGCGCCACAACCACTTA 16749

NC_002645.1 TGTGTTTGAGAAAGTAGATTACGGTTCTGATACGGTTACTTACAAATCCACTGCTACTAC 15929

JX504050.1 CATCTTTGAGAAGGTTGAATATGGTTCTGATACTGTTACGTATAAGTCTACTGTAACTAC 15848

** ** ** * * * ** ** * **

NC_019843.3 TAAACTGACTGTAGGTGACATCTTCGTACTTACCTCTCACTCTGTGGCTACCTTGACGGC 16917

NC_004718.3 CAAGTTGAATGTTGGTGATTACTTTGTGTTGACATCTCACACTGTAATGCCACTTAGTGC 16876

EPI_ISL_6640916_omicron CAAATTAAATGTTGGTGATTATTTTGTGCTGACATCACATACAGTAATGCCATTAAGTGC 16880

MW494315.1 CAAATTAAATGTTGGTGATTATTTTGTGCTGACATCACATACAGTAATGCCATTAAGTGC 16946

KF686346.1 TAAACTTTCTATAGGTGATGTTTTTGTTTTAACATCACATTCTGTAGCTAGTTTAAGTGC 17149

NC_006213.1 TAAGCTATCTGTAGGAGATGTTTTTGTTTTAACCTCTCATTCAGTAGCTAATTTAAGTGC 16809

NC_002645.1 TAAGTTAGTACCAGGTATGTTGTTTATTTTGACTTCTCATAATGTTGCTCCACTTAGAGC 15989

JX504050.1 TAAGTTAGTTCCTGGTATGATTTTTGTCTTAACATCTCACAATGTCCAACCTTTACGTGC 15908

** * ** ** * * ** ** ** ** * **

NC_019843.3 GCCCACAATTGTGAATCAAGAGAGGTATGTTAAAATTACTGGGTTGTACCCAACCATTAC 16977

NC_004718.3 ACCTACTCTAGTGCCACAAGAGCACTATGTGAGAATTACTGGCTTGTACCCAACACTCAA 16936

EPI_ISL_6640916_omicron ACCTACACTAGTGCCACAAGAGCACTATGTTAGAATTACTGGCTTATACCCAACACTCAA 16940

MW494315.1 ACCTACACTAGTGCCACAAGAGCACTATGTTAGAATTACTGGCTTATACCCAACACTCAA 17006

KF686346.1 ACCTACACTTGTCCCACAAGAGAACTATGCTAGTATA---AGATTTTCTAGTGTTTATAG 17206

NC_006213.1 TCCTACGCTTGTTCCGCAGGAGAATTATAGTAGTATT---AGATTTGCTAGTGTTTATAG 16866

NC_002645.1 GCCAACAATGGCAAACCAGGAGAAATATTCTACCATTTACAAGTTGCACCCATCATTTAA 16049

JX504050.1 ACCAACTATTGCAAACCAAGAGAAGTATTCTAGCATTTATAAATTGCACCCTGCTTTTAA 15968

** ** * * ** *** *** * ** ** *

NC_019843.3 GGTACCTGAAGAGTTCGCAAGTCATGTTGCCAACTTCCAAAAATCAGGTTATAGTAAATA 17037

NC_004718.3 CATCTCAGATGAGTTTTCTAGCAATGTTGCAAATTATCAAAAGGTCGGCATGCAAAAGTA 16996

EPI_ISL_6640916_omicron TATCTCAGATGAGTTTTCTAGCAATGTTGCAAATTATCAAAAGGTTGGTATGCAAAAGTA 17000

MW494315.1 TATCTCAGATGAGTTTTCTAGCAATGTTGCAAATTATCAAAAGGTTGGTATGCAAAAGTA 17066

KF686346.1 TGTTCCATTGGTGTTTCAAAATAATGTTGCTAATTATCAGCACATTGGAATGAAACGTTA 17266

NC_006213.1 TGTGCTTGAGACGTTTCAGAACAATGTTGTTAATTATCAACACATTGGTATGAAACGTTA 16926

NC_002645.1 TGTTAGTGATGCTTATGCAAATCTTGTACCTTATTACCAACTTATTGGCAAACAGCGTAT 16109

JX504050.1 TGTCAGTGATGCATATGCTAATTTGGTTCCATATTACCAACTTATTGGTAAACAAAAGAT 16028

* * * ** * * ** **

NC_019843.3 TGTCACTGTTCAGGGACCACCTGGCACTGGCAAAAGTCATTTTGCTATAGGGTTAGCGAT 17097

NC_004718.3 CTCTACACTCCAAGGACCACCTGGTACTGGTAAGAGTCATTTTGCCATCGGACTTGCTCT 17056

EPI_ISL_6640916_omicron TTCTACACTCCAGGGACCACCTGGTACTGGTAAGAGTCATTTTGCTATTGGCCTAGCTCT 17060

MW494315.1 TTCTACACTCCAGGGACCACCTGGTACTGGTAAGAGTCATTTTGCTATTGGCCTAGCTCT 17126

KF686346.1 TTGCACTGTTCAAGGTCCCCCTGGTACGGGAAAGTCTCATCTTGCTATAGGTCTAGCTGT 17326

NC_006213.1 CTGCACCGTGCAAGGACCTCCTGGTACAGGGAAGTCACATCTTGCTATTGGTCTTGCTGT 16986

NC_002645.1 AACCACAATACAGGGTCCTCCTGGTAGTGGAAAATCGCATTGTTCTATTGGTATTGGTGT 16169

JX504050.1 AACTACAATACAGGGTCCTCCTGGTAGTGGTAAGTCACATTGTTCCATTGGACTTGGATT 16088

** * ** ** ** ***** * ** ** *** * * ** ** * * *

NC_019843.3 TTACTACCCTACAGCACGTGTTGTTTATACAGCATGTTCACACGCAGCTGTTGATGCTTT 17157

NC_004718.3 CTATTACCCATCTGCTCGCATAGTGTATACGGCATGCTCTCATGCAGCTGTTGATGCCCT 17116

EPI_ISL_6640916_omicron CTACTACCCTTCTGCTCGCATAGTGTATACAGCTTGCTCTCATGCCGCTGTTGATGCACT 17120

MW494315.1 CTACTACCCTTCTGCTCGCATAGTGTATACAGCTTGCTCTCATGCCGCTGTTGATGCACT 17186

KF686346.1 TTATTACTACACAGCACGTGTAGTTTATACTGCTGCTAGTCATGCTGCTGTAGATGCATT 17386

NC_006213.1 ATTCTATTGTACAGCACGTGTTGTATACACAGCGGCCAGCCATGCAGCTGTTGACGCATT 17046

NC_002645.1 GTATTACCCTGGAGCGAGGATCGTGTTCACCGCTTGTTCTCACGCTGCTGTTGATTCGCT 16229

JX504050.1 GTACTACCCAGGTGCGCGTATTGTTTTTGTTGCTTGTGCCCATGCTGCTGTTGATTCCTT 16148

* ** ** * * ** * ** ** ** ***** ** * *

NC_019843.3 GTGTGAAAAAGCTTTTAAATATTTGAACATTGCTAAATGTTCCCGTATCATTCCTGCAAA 17217

NC_004718.3 ATGTGAAAAGGCATTAAAATATTTGCCCATAGATAAATGTAGTAGAATCATACCTGCGCG 17176

EPI_ISL_6640916_omicron ATGTGAGAAGGCATTAAAATATTTGCCTATAGATAAATGTAGTAGAATTATACCTGCACG 17180

MW494315.1 ATGTGAGAAGGCATTAAAATATTTGCCTATAGATAAATGTAGTAGAATTATACCTGCACG 17246

KF686346.1 GTGTGAAAAAGCTTATAAGTTTTTAAATATTAACGATTGTACACGTATTATTCCTGCTAA 17446

NC_006213.1 GTGTGAAAAAGCATATAAATTTTTGAATATAAATGATTGCACTCGTATTGTTCCGGCCAA 17106

NC_002645.1 CTGTGCAAAAGCTGTCACAGCCTATAGTGTTGATAAGTGTACACGTATTATTCCTGCACG 16289

JX504050.1 ATGTGCAAAAGCTATGACTGTTTATAGCATTGATAAGTGTACTAGGATTATACCTGCAAG 16208

**** ** ** * * * * ** * ** * ** **

NC_019843.3 GGCACGTGTTGAGTGCTATGACAGGTTTAAAGTTAATGAGACAAATTCTCAATATTTGTT 17277

NC_004718.3 TGCGCGCGTAGAGTGTTTTGATAAATTCAAAGTGAATTCAACACTAGAACAGTATGTTTT 17236

EPI_ISL_6640916_omicron TGCTCGTGTAGAGTGTTTTGATAAATTCAAAGTGAATTCAACATTAGAACAGTATGTCTT 17240

MW494315.1 TGCTCGTGTAGAGTGTTTTGATAAATTCAAAGTGAATTCAACATTAGAACAGTATGTCTT 17306

KF686346.1 AGTTCGTGTAGATTGTTATGATAAGTTTAAAATTAATGATACCACTTGTAAGTATGTTTT 17506

NC_006213.1 GGTCAGGGTGGAGTGCTATGATAAGTTTAAAATTAATGACACCACTCGTAAGTATGTGTT 17166

NC_002645.1 TGCCAGAGTTGAGTGTTATAGTGGTTTTAAACCTAACAATAATAGTGCACAATACGTGTT 16349

JX504050.1 AGCTCGGGTTGAGTGTTATAGTGGCTTTAAACCAAATAACACTAGTGCACAATACATATT 16268

* * ** ** ** * * ** *** ** * * ** * **

NC_019843.3 TAGTACTATTAATGCTCTACCAGAAACTTCTGCCGATATTCTGGTGGTTGATGAGGTTAG 17337

NC_004718.3 CTGCACTGTAAATGCATTGCCAGAAACAACTGCTGACATTGTAGTCTTTGATGAAATCTC 17296

EPI_ISL_6640916_omicron TTGTACTGTAAATGCATTGCCTGAGACGACAGCAGATATAGTTGTCTTTGATGAAATTTC 17300

MW494315.1 TTGTACTGTAAATGCATTGCCTGAGACGACAGCAGATATAGTTGTCTTTGATGAAATTTC 17366

KF686346.1 TACCACAATAAATGCATTACCAGAGTTGGTTACAGATATTGTTGTTGTTGATGAAGTTAG 17566

NC_006213.1 TACTACCATAAATGCATTACCTGAGATGGTGACTGATATTGTTGTTGTAGATGAAGTTAG 17226

NC_002645.1 TAGTACTGTTAATGCGTTACCTGAAGTTAATGCAGACATTGTTGTCGTGGATGAGGTGTC 16409

JX504050.1 TAGCACTGTTAACGCATTACCTGAGTGTAATGCTGATATCGTTGTTGTAGATGAAGTTTC 16328

** * ** ** * ** ** * ** ** * ** * ***** *

NC_019843.3 TATGTGCACTAATTATGATCTTTCAATTATTAATGCACGTATTAAAGCTAAGCACATTGT 17397

NC_004718.3 TATGGCTACTAATTATGACTTGAGTGTTGTCAATGCTAGACTTCGTGCAAAACACTACGT 17356

EPI_ISL_6640916_omicron AATGGCCACAAATTATGATTTGAGTGTTGTCAATGCCAGATTACGTGCTAAGCACTATGT 17360

MW494315.1 AATGGCCACAAATTATGATTTGAGTGTTGTCAATGCCAGATTACGTGCTAAGCACTATGT 17426

KF686346.1 TATGCTTACTAATTATGAATTGTCTGTTATAAATGCTCGTATTAAAGCTAAACATTATGT 17626

NC_006213.1 TATGCTTACCAATTATGAGCTTTCTGTTATTAATGCTCGTATTCGCGCTAAGCATTATGT 17286

NC_002645.1 TATGTGCACTAACTATGACTTGTCTGTGATTAACCAGCGTATATCATATAAACACATTGT 16469

JX504050.1 AATGTGTACAAATTATGACCTTTCTGTTATTAACCAGCGTTTATCATATAAACATATTGT 16388

*** ** ** ***** * * * ** * * ** ** **

NC_019843.3 CTATGTAGGAGATCCAGCACAGTTGCCAGCTCCTAGGACTTTGTTGACTAGAGGCACATT 17457

NC_004718.3 CTATATTGGCGATCCTGCTCAATTACCAGCCCCCCGCACATTGCTGACTAAAGGCACACT 17416

EPI_ISL_6640916_omicron GTACATTGGCGACCCTGCTCAATTACCTGCACCACGCACATTGCTAACTAAGGGCACACT 17420

MW494315.1 GTACATTGGCGACCCTGCTCAATTACCTGCACCACGCACATTGCTAACTAAGGGCACACT 17486

KF686346.1 ATATATTGGAGATCCTGCTCAATTACCTGCACCACGTGTGCTGTTGAGCAAGGGTTCTTT 17686

NC_006213.1 TTATATTGGTGATCCTGCTCAATTGCCAGCACCACGTGTGTTATTGAGCAAGGGTACACT 17346

NC_002645.1 ATATGTTGGTGATCCTCAACAGCTTCCAGCTCCTAGAGTTCTTATCTCTAAAGGTGTTAT 16529

JX504050.1 TTATGTTGGTGATCCACAACAACTTCCTGCACCTAGAGTAATGATTACTAAAGGTGTTAT 16448

** * ** ** ** ** * ** ** ** * * * * ** *

NC_019843.3 GGAACCAGAAAATTTCAATAGTGTCACTAGATTGATGTGTAACTTAGGTCCTGACATATT 17517

NC_004718.3 AGAACCAGAATATTTTAATTCAGTGTGCAGACTTATGAAAACAATAGGTCCAGACATGTT 17476

EPI_ISL_6640916_omicron AGAACCAGAATATTTCAATTCAGTGTGTAGACTTATGAAAACTATAGGTCCAGACATGTT 17480

MW494315.1 AGAACCAGAATATTTCAATTCAGTGTGTAGACTTATGAAAACTATAGGTCCAGACATGTT 17546

KF686346.1 AGAACCTAGGCACTTCAATTCTATTACTAAAATAATGTGTTGTTTAGGTCCTGATATCTT 17746

NC_006213.1 TGAACCTAAATATTTTAACACTGTTACTAAGCTCATGTGTTGCTTAGGGCCAGACATTTT 17406

NC_002645.1 GGAACCAATTGACTATAATGTTGTGACACAACGTATGTGTGCTATAGGACCCGATGTCTT 16589

JX504050.1 GGAGCCTGTTGATTATAACGTTGTTACTCAACGTATGTGTGCTATAGGCCCTGATGTTTT 16508

** ** * * ** * *** **** ** ** * **

NC_019843.3 TTTAAGTATGTGCTACAGGTGTCCTAAGGAAATAGTAAGCACTGTGAGCGCTCTTGTCTA 17577

NC_004718.3 CCTTGGAACTTGTCGCCGTTGTCCTGCTGAAATTGTTGACACTGTGAGTGCTTTAGTTTA 17536

EPI_ISL_6640916_omicron CCTCGGAACTTGTCGGCGTTGTCCTGCTGAAATTGTTGACACTGTGAGTGCTTTGGTTTA 17540

MW494315.1 CCTCGGAACTTGTCGGCGTTGTCCTGCTGAAATTGTTGACACTGTGAGTGCTTTGGTTTA 17606

KF686346.1 TTTGGGAAATTGTTATAGGTGTCCTAAAGAAATTGTAGAAACTGTTTCAGCATTGGTTTA 17806

NC_006213.1 TCTTGGTACATGTTATAGATGTCCTAAGGAAATCGTTGATACAGTGTCCGCCTTGGTTTA 17466

NC_002645.1 TTTACACAAGTGTTACAGATGTCCTGCTGAAATAGTTAACACTGTTTCAGAGCTTGTTTA 16649

JX504050.1 TCTTCATAAATGTTATAGATGTCCTGCTGAAATAGTTAATACAGTTTCTGAACTTGTTTA 16568

* * ** * ****** ***** ** ** ** * * ** **

NC_019843.3 CAATAATAAATTGTTAGCCAAGAAGGAGCTTTCAGGCCAGTGCTTTAAAATACTCTATAA 17637

NC_004718.3 TGACAATAAGCTAAAAGCACACAAGGATAAGTCAGCTCAATGCTTCAAAATGTTCTACAA 17596

EPI_ISL_6640916_omicron TGATAATAAGCTTAAAGCACATAAAGACAAATCAGCTCAATGCTTTAAAATGTTTTATAA 17600

MW494315.1 TGATAATAAGCTTAAAGCACATAAAGACAAATCAGCTCAATGCTTTAAAATGTTTTATAA 17666

KF686346.1 TGATAATAAACTCAAGGCTAAAAATGATAATAGTTCATTATGTTTTAAAGTATATTTTAA 17866

NC_006213.1 TGAAAATAAGCTTAAGGCTAAGAATGAGAGTAGTTCATTGTGTTTTAAGGTCTATTATAA 17526

NC_002645.1 TGAAAACAAGTTTGTACCTGTCAAAGAAGCTAGTAAGCAGTGCTTCAAAATCTTTGAACG 16709

JX504050.1 TGAGAACAAGTTTGTCCCTGTTAAACCTGCTAGTAAACAGTGTTTTAAAGTCTTTTTTAA 16628

* ** ** * * ** ** ** ** *

NC_019843.3 GGGCAATGTGACGCATGATGCTAGCTCTGCCATTAATAGACCACAACTCACATTTGTGAA 17697

NC_004718.3 AGGTGTTATTACACATGATGTTTCATCTGCAATCAACAGACCTCAAATAGGCGTTGTAAG 17656

EPI_ISL_6640916_omicron GGGTGTTATCACGCATGATGTTTCATCTGCAATTAACAGGCCACAAATAGGCGTGGTAAG 17660

MW494315.1 GGGTGTTATCACGCATGATGTTTCATCTGCAATTAACAGGCCACAAATAGGCGTGGTAAG 17726

KF686346.1 GGGACAGACAACACATGAGAGTTCAAGTGCTGTAAATATTCAACAGATATATCTAATTAG 17926

NC_006213.1 GGGCGTTACAACACATGAAAGTTCTAGTGCTGTAAATATGCAGCAGATTTATTTGATTAA 17586

NC_002645.1 CGGTAGTGTTCAGGTAGACAATGGCTCCAGTATAAATAGGCGTCAACTTGATGTTGTTAA 16769

JX504050.1 GGGTAATGTACAGGTTGACAATGGTTCTAGTATTAACAGAAAGCAGCTTGAAATAGTTAA 16688

** ** * * ** * ** * * * *

NC_019843.3 GAATTTTATTACTGCCAATCCGGCATGGAGTAAGGCAGTCTTTATTTCGCCTTACAATTC 17757

NC_004718.3 AGAATTTCTTACACGCAATCCTGCTTGGAGAAAAGCTGTTTTTATCTCACCTTATAATTC 17716

EPI_ISL_6640916_omicron AGAATTCCTTACACGTAACCCTGCTTGGAGAAAAGCTGTCTTTATTTCACCTTATAATTC 17720

MW494315.1 AGAATTCCTTACACGTAACCCTGCTTGGAGAAAAGCTGTCTTTATTTCACCTTATAATTC 17786

KF686346.1 TAAATTTTTAAAAGCTAATCCAGTTTGGAATAGTGCTGTTTTTATTAGTCCTTATAATAG 17986

NC_006213.1 TAAGTTTTTGAAGGCTAACCCTTTGTGGCATAAAGCTGTTTTTATTAGCCCATATAATAG 17646

NC_002645.1 GCGATTTATACATAAAAACTCCACATGGAGCAAGGCTGTGTTTATCTCACCTTACAATAG 16829

JX504050.1 GCTGTTTTTAGTTAAAAATCCAAGTTGGAGTAAGGCTGTGTTTATTTCTCCTTATAATAG 16748

** * ** * *** * ** ** ***** ** ** ***

NC_019843.3 ACAGAATGCTGTGTCTCGTTCAATGCTGGGTCTTACCACTCAGACTGTTGATTCCTCACA 17817

NC_004718.3 ACAGAACGCTGTAGCTTCAAAAATCTTAGGATTGCCTACGCAGACTGTTGATTCATCACA 17776

EPI_ISL_6640916_omicron ACAGAATGCTGTAGCCTCAAAGATTTTGGGACTACCAACTCAAACTGTTGATTCATCACA 17780

MW494315.1 ACAGAATGCTGTAGCCTCAAAGATTTTGGGACTACCAACTCAAACTGTTGATTCATCACA 17846

KF686346.1 TCAGAATTATGTTGCTAAGCGTGTTTTAGGTGTTCAAACACAAACTGTAGATTCTGCTCA 18046

NC_006213.1 TCAGAACTTTGCAGCTAAGCGTGTTTTGGGTTTACAAACCCAAACCGTGGATTCTGCTCA 17706

NC_002645.1 TCAAAATTATGTAGCTGCCAGGCTTTTAGGCTTACAAACTCAGACAGTGGATTCTGCTCA 16889

JX504050.1 TCAGAATTATGTTGCTAGTAGATTTTTAGGACTTCAAATTCAAACTGTTGATTCTTCTCA 16808

** ** ** * * * ** * * ** ** ** ***** * **

NC_019843.3 GGGTTCAGAATACCAGTACGTTATCTTCTGTCAAACAGCAGATACGGCACATGCTAACAA 17877

NC_004718.3 GGGTTCTGAATATGACTATGTCATATTCACACAAACTACTGAAACAGCACACTCTTGTAA 17836

EPI_ISL_6640916_omicron GGGCTCAGAATATGACTATGTCATATTCACTCAAACCACTGAAACAGCTCACTCTTGTAA 17840

MW494315.1 GGGCTCAGAATATGACTATGTCATATTCACTCAAACCACTGAAACAGCTCACTCTTGTAA 17906

KF686346.1 AGGTTCGGAATATGATTACGTTATATATTCACAAACAGCAGAAACAGCCCATTCTGTTAA 18106

NC_006213.1 AGGTTCTGAATATGATTATGTTATATATTCACAGACTGCAGAAACAGCGCATTCTGTAAA 17766

NC_002645.1 AGGTAGTGAATATGACTATGTTATATTCGCACAGACATCAGATACTGCTCATGCCTGTAA 16949

JX504050.1 AGGTAGTGAGTATGATTATGTAATCTATGCACAAACTTCTGACACTGCACATGCTTGCAA 16868

** ** ** * ** ** ** * ** ** * ** ** ** ** * **

NC_019843.3 CATTAACAGATTTAATGTTGCAATCACTCGTGCCCAAAAAGGTATTCTTTGTGTTATGAC 17937

NC_004718.3 TGTCAACCGCTTCAATGTGGCTATCACAAGGGCAAAAATTGGCATTTTGTGCATAATGTC 17896

EPI_ISL_6640916_omicron TGTAAACAGATTTAATGTTGCTATTACCAGAGCAAAAGTAGGCATACTTTGCATAATGTC 17900

MW494315.1 TGTAAACAGATTTAATGTTGCTATTACCAGAGCAAAAGTAGGCATACTTTGCATAATGTC 17966

KF686346.1 TGTTAATCGATTTAATGTTGCCATAACTAGAGCCAAGAAGGGCATTTTTTGTGTTATGAG 18166

NC_006213.1 TGTTAATCGCTTCAATGTTGCTATTACTCGAGCCAAGAAAGGTATTCTTTGTGTTATGAG 17826

NC_002645.1 TGCCAATCGTTTTAACGTTGCCATTACTAGAGCAAAGAAAGGTATTTTCTGTATTATGTC 17009

JX504050.1 TGTAAACCGTTTTAATGTTGCTATAACACGTGCTAAGAAGGGTATATTTTGTGTAATGTG 16928

** * ** ** ** ** ** ** * ** * ** ** * ** * ***

NC_019843.3 ATCTCAGGCACTCTTTGAGTCCTTAGAGTTTACTGAATTGTCTTTTACTAATTACAAGCT 17997

NC_004718.3 TGATAGAGATCTTTATGACAAACTGCAATTTACAAGTCTAGAAATACCACGTCGCAATGT 17956

EPI_ISL_6640916_omicron TGATAGAGACCTTTATGACAAGTTGCAATTTACAAGTCTTGAAATTCCACGTAGGAATGT 17960

MW494315.1 TGATAGAGACCTTTATGACAAGTTGCAATTTACAAGTCTTGAAATTCCACGTAGGAATGT 18026

KF686346.1 TAATATGCAATTATTTGAATCTCTTAATTTTATTACTCTACCTTTAGATAAAATTCAAAA 18226

NC_006213.1 TAATATGCAGTTGTTTGAAGCATTACAGTTTACTACATTGACCTTAGATAAAGTGCCACA 17886

NC_002645.1 TGACAGAACTTTGTTTGATGCACTTAAGTTCTT---------------TGAAATCACTAT 17054

JX504050.1 TGATAAAACTTTGTTTGATTCACTTAAGTTTTT---------------TGAGATTAAACA 16973

* * *** * * **

NC_019843.3 C------------CAGTCTCAGATTGTAACTGGCCTTTTTAAAGATTGCTCTAGAGAAAC 18045

NC_004718.3 GGCTACATTA---CAAGCAGAAAATGTAACTGGACTTTTTAAGGACTGTAGTAAGATCAT 18013

EPI_ISL_6640916_omicron GGCAACTTTA---CAAGCTGAAAATGTAACAGGACTCTTTAAAGATTGTAGTAAGGTAAT 18017

MW494315.1 GGCAACTTTA---CAAGCTGAAAATGTAACAGGACTCTTTAAAGATTGTAGTAAGGTAAT 18083

KF686346.1 TCAAACTTTACCTCGTTTGCATTGCACAACTAATCTTTTTAAAGATTGTAGTAAAAGTTG 18286

NC_006213.1 GGCCGTCGAAACTAAAGTTCAATGTAGTACTAATTTATTTAAAGATTGTAGCAAGAGTTA 17946

NC_002645.1 GACAGATTTA---CAGTCTGAAAGTAGTTGTGGTTTGTTTAAGGATTGTGCACGTAACCC 17111

JX504050.1 TGCAGATTTA---CACTCTAGCCAGGTTTGTGGCTTGTTTAAAAATTGTACACGCACTCC 17030

* ***** * **

NC_019843.3 TTCTGGCCTCTCACCTGCTTATGCACCAACATATGTTAGTGTTGATGACAAGTATAAGAC 18105

NC_004718.3 TACTGGTCTTCATCCTACACAGGCACCTACACACCTCAGCGTTGATATAAAGTTCAAGAC 18073

EPI_ISL_6640916_omicron CACTGGGTTACATCCTACACAGGCACCTACACACCTCAGTGTTGACACTAAATTCAAAAC 18077

MW494315.1 CACTGGGTTACATCCTACACAGGCACCTACACACCTCAGTGTTGACACTAAATTCAAAAC 18143

KF686346.1 CTTAGGTTATCATCCAGCGCATGCCCCCTCATTTTTAGCAGTTGATGATAAATATAAGGT 18346

NC_006213.1 TAGCGGTTATCACCCAGCTCATGCTCCTTCATTTTTGGCAGTAGATGACAAATATAAGGC 18006

NC_002645.1 TATTGATTTACCACCAAGTCATGCCACTACTTATTTGTCATTGTCTGATAGATTTAAGAC 17171

JX504050.1 TCTTAATTTACCACCAACTCATGCACACACTTTCTTGTCGTTGTCAGATCAGTTTAAGAC 17090

** * ** * * * * **

NC_019843.3 GAGTGATGAGCTTTGCGTGAATCTTAATTTACCCGC---AAATGTCCCATACTCTCGTGT 18162

NC_004718.3 TGAAGGATTATGTGTTGACATACCAGGCATACCAAA---GGACATGACCTACCGTAGACT 18130

EPI_ISL_6640916_omicron TGAAGGTTTATGTGTTGACGTACCTGGCATACCTAA---GGACATGACCTATAGAAGACT 18134

MW494315.1 TGAAGGTTTATGTGTTGACATACCTGGCATACCTAA---GGACATGACCTATAGAAGACT 18200

KF686346.1 TAATGAAAATTTGGCTGTAAATTTAAATATTTGTGAACCTGTTTTAACATATTCTCGTTT 18406

NC_006213.1 AACTGGCGATTTAGCCGTGTGTCTTGGTATTGGTGATTCTGCTGTTACATATTCAAGATT 18066

NC_002645.1 TAGTGGTGACTTGGCTGTTCAAATAGGTAACAACAA---TGTTTGTACCTATGAACATGT 17228

JX504050.1 TACAGGTGATTTAGCTGTTCAAATAGGTTCAAATAA---CGTTTGTACTTATGAACATGT 17147

* * * ** *

NC_019843.3 TATTTCCAGGATGGGCTTTAAACTCGATGCAACAGTTCCTGGATATCCTAAGCTTTTCAT 18222

NC_004718.3 CATCTCTATGATGGGTTTCAAAATGAATTACCAAGTCAATGGTTACCCTAATATGTTTAT 18190

EPI_ISL_6640916_omicron CATCTCTATGATGGGTTTTAAAATGAATTATCAAGTTAATGGTTACCCTAACATGTTTAT 18194

MW494315.1 CATCTCTATGATGGGTTTTAAAATGAATTATCAAGTTAATGGTTACCCTAACATGTTTAT 18260

KF686346.1 AATATCTCTTATGGGTTTTAAATTAGATTTGACTCTTGATGGTTATTCTAAATTGTTTAT 18466

NC_006213.1 AATATCACTCATGGGTTTTAAATTGGATGTTACCCTTGATGGGTATTGTAAGCTTTTTAT 18126

NC_002645.1 GATTTCATATATGGGTTTCAGGTTTGATGTTAGCATGCCTGGTAGTCATAGTTTGTTCTG 17288

JX504050.1 TATATCATTTATGGGTTTTAGGTTTGATATTAGTATTCCTGGTAGTCATAGTTTGTTTTG 17207

** ** ***** ** * * ** * *** ** * **

NC_019843.3 TACTCGTGAAGAGGCTGTAAGGCAAGTTCGAAGCTGGATAGGCTTCGATGTTGAGGGTGC 18282

NC_004718.3 CACCCGCGAAGAAGCTATTCGTCACGTTCGTGCGTGGATTGGCTTTGATGTAGAGGGCTG 18250

EPI_ISL_6640916_omicron CACCCGCGAAGAAGCTATAAGACATGTACGTGCATGGATTGGCTTCGATGTCGAGGGGTG 18254

MW494315.1 CACCCGCGAAGAAGCTATAAGACATGTACGTGCATGGATTGGCTTCGATGTCGAGGGGTG 18320

KF686346.1 TACTAAAGATGAAGCCATTAAACGTGTTAGAGGTTGGGTTGGTTTTGATGTTGAAGGCGC 18526

NC_006213.1 AACTAAAGAAGAAGCTGTTAAACGCGTGCGTGCCTGGGTTGGCTTTGATGCTGAAGGTGC 18186

NC_002645.1 TACTAGAGACTTTGCCATGCGTCATGTCAGAGGTTGGTTAGGAATGGATGTGGAAGGTGC 17348

JX504050.1 TACACGTGACTTTGCTATTCGTAATGTGCGTGGTTGGTTGGGTATGGATGTTGAAAGTGC 17267

** ** ** * ** * *** * ** * **** ** *

NC_019843.3 TCATGCTTCCCGTAATGCATGTGGCACCAATGTGCCTCTACAATTAGGATTTTCAACTGG 18342

NC_004718.3 TCATGCAACTAGAGATGCTGTGGGTACTAACCTACCTCTCCAGCTAGGATTTTCTACAGG 18310

EPI_ISL_6640916_omicron TCATGCTACTAGAGAAGCTGTTGGTACCAATTTACCTTTACAGCTAGGTTTTTCTACAGG 18314

MW494315.1 TCATGCTACTAGAGAAGCTGTTGGTACCAATTTACCTTTACAGCTAGGTTTTTCTACAGG 18380

KF686346.1 TCATGCTACTCGCGAAAACATTGGAACAAACTTTCCACTGCAAATAGGTTTTTCAACTGG 18586

NC_006213.1 TCATGCCACGCGTGATAGCATTGGGACAAATTTCCCACTTCAATTAGGATTTTCCACAGG 18246

NC_002645.1 ACATGTCACAGGTGACAATGTTGGCACTAATGTACCTCTACAAGTTGGTTTTTCCAATGG 17408

JX504050.1 TCATGTTTGTGGCGATAACATAGGTACTAATGTTCCTTTACAGGTTGGTTTTTCAAATGG 17327

**** * * ** ** ** * ** * ** * ** ***** * **

NC_019843.3 TGTGAACTTTGTTGTTCAGCCAGTTGGTGTTGTAGACACTGAGTGGGGTAACATGTTAAC 18402

NC_004718.3 TGTTAACTTAGTAGCTGTACCGACTGGTTATGTTGACACTGAAAATAACACAGAATTCAC 18370

EPI_ISL_6640916_omicron TGTTAACCTAGTTGCTGTACCTACAGGTTATGTTGATACACCTAATAATACAGATTTTTC 18374

MW494315.1 TGTTAACCTAGTTGCTGTACCTACAGGTTATGTTGATACACCTAATAATACAGATTTTTC 18440

KF686346.1 TGTGGATTTTGTAGTTGAAGCTACTGGCTTATTTGCTGAGAGAGATTGTTATACTTTTAA 18646

NC_006213.1 AATTGATTTTGTTGTGGAAGCCACTGGTTTGTTTGCTGATAGAGATGGTTACAGCTTTAA 18306

NC_002645.1 TGTTGATTTTGTAGCTCAACCTGAAGGTTGTGTTCTAACAAACACTGGCAGTGTTGTAAA 17468

JX504050.1 TGTTAATTTTGTTGTGCAAACTGAAGGTTGTGTGTCTACCAATTTTGGTGATGTTATTAA 17387

* * * ** * * ** * *

NC_019843.3 GGGCATTGCTGCACGTCCTCCACCAGGTGAACAGTTTAAGCACCTCGTGCCTCTTATGCA 18462

NC_004718.3 CAGAGTTAATGCAAAACCTCCACCAGGTGACCAGTTTAAACATCTTATACCACTCATGTA 18430

EPI_ISL_6640916_omicron CAGAGTTAGTGCTAAACCACCGCCTGGAGATCAATTTAAACACCTCATACCACTTATGTA 18434

MW494315.1 CAGAGTTAGTGCTAAACCACCGCCTGGAGATCAATTTAAACACCTCATACCACTTATGTA 18500

KF686346.1 AAAAACTGTAGCTAAAGCTCCTCCTGGTGAAAAATTTAAACATTTAATACCCCTTATGTC 18706

NC_006213.1 AAAGGCTGTGGCGAAAGCTCCTCCTGGTGAACAATTTAAGCACCTCATCCCTTTGATGAC 18366

NC_002645.1 ACCTGTTCGTGCTCGTGCACCACCTGGAGAACAATTCACTCACATTGTACCTCTGTTACG 17528

JX504050.1 ACCTGTTTGTGCAAAATCTCCACCAGGTGAACAATTTAGACACCTTATTCCTCTTTTACG 17447

* ** * ** ** ** ** * ** * ** * * ** * *

NC_019843.3 TAAGGGGGCTGCGTGGCCTATTGTTAGACGACGTATAGTGCAAATGTTGTCAGACACTTT 18522

NC_004718.3 TAAAGGCTTGCCCTGGAATGTAGTGCGTATTAAGATAGTACAAATGCTCAGTGATACACT 18490

EPI_ISL_6640916_omicron CAAAGGACTTCCTTGGAATGTAGTGCGTATAAAGATTGTACAAATGTTAAGTGACACACT 18494

MW494315.1 CAAAGGACTTCCTTGGAATGTAGTGCGTATAAAGATTGTACAAATGTTAAGTGACACACT 18560

KF686346.1 AAAAGGTCAAAAGTGGGATATTGTTAGAATTAGAATTGTTCAAATGTTATCTGATTATCT 18766

NC_006213.1 GAGAGGTCATCGCTGGGATGTTGTTAGACCTAGAATAGTACAAATGTTTGCAGATCATTT 18426

NC_002645.1 CAAGGGACAACCTTGGAGTGTGTTGAGAAAACGTATTGTTCAAATGATAGCAGATTTTCT 17588

JX504050.1 TAAAGGACAACCTTGGTTAATTGTTCGTAGACGCATTGTGCAAATGATATCTGATTATTT 17507

* ** *** * * * ** ** ****** * ** *

NC_019843.3 AGACAAATTGTCTGATTACTGTACGTTTGTTTGTTGGGCTCATGGCTTTGAATTAACGTC 18582

NC_004718.3 GAAAGGATTGTCAGACAGAGTCGTGTTCGTCCTTTGGGCGCATGGCTTTGAGCTTACATC 18550

EPI_ISL_6640916_omicron TAAAAATCTCTCTGACAGAGTCGTATTTGTCTTATGGGCACATGGCTTTGAGTTGACATC 18554

MW494315.1 TAAAAATCTCTCTGACAGAGTCGTATTTGTCTTATGGGCACATGGCTTTGAGTTGACATC 18620

KF686346.1 TTTAGACCTTTCTGATAGTGTAGTATTTATTACTTGGTCTGCCAGTTTTGAACTTACTTG 18826

NC_006213.1 AATTGATCTGTCTGATTGTGTTGTGCTAGTTACATGGGCAGCCAACTTTGAGCTCACTTG 18486

NC_002645.1 TGCTGGCTCATCTGATGTACTGGTGTTTGTACTTTGGGCTGGCGGTTTAGAGTTGACCAC 17648

JX504050.1 GTCCAATTTGTCTGACATTCTTGTCTTTGTTTTGTGGGCAGGTAGTTTGGAATTAACTAC 17567

** ** * * *** * ** ** * **

NC_019843.3 TGCATCATACTTTTGCAAGATAGGTAAGGAACAGAAGTGTTGCATGTGCAATAGACGCGC 18642

NC_004718.3 AATGAAGTACTTTGTCAAGATTGGACCTGAAAGAACGTGTTGTCTGTGTGACAAACGTGC 18610

EPI_ISL_6640916_omicron TATGAAGTATTTTGTGAAAATAGGACCTGAGCGCACCTGTTGTCTATGTGATAGACGTGC 18614

MW494315.1 TATGAAGTATTTTGTGAAAATAGGACCTGAGCGCACCTGTTGTCTATGTGATAGACGTGC 18680

KF686346.1 TTTAAGGTATTTTGCTAAATTAGGCAGAGAGCTTAATTGTAATGTGTGTTCTAATCGTGC 18886

NC_006213.1 TCTCCGCTACTTTGCAAAAGTAGGGCGTGAGATTTCTTGTAATGTATGCACTAAACGTGC 18546

NC_002645.1 TATGCGTTATTTTGTTAAGATTGGAGCTGTTAAACATTGCCAA---TGTGGTACTGTTGC 17705

JX504050.1 AATGCGTTACTTTGTAAAAATAGGGCCAATTAAATATTGTTAT---TGTGGTAATTCTGC 17624

** *** ** * ** ** ** * **

NC_019843.3 TGCAGCGTACTCTTCACCTCTGCAATCTTATGCCTGCTGGACTCATTCCTGCGGTTATGA 18702

NC_004718.3 AACTTGCTTTTCTACTTCATCAGATACTTATGCCTGCTGGAATCATTCTGTGGGTTTTGA 18670

EPI_ISL_6640916_omicron CACATGCTTTTCCACTGCTTCAGACACTTATGCCTGTTGGCATCATTCTATTGGATTTGA 18674

MW494315.1 CACATGCTTTTCCACTGCTTCAGACACTTATGCCTGTTGGCATCATTCTATTGGATTTGA 18740

KF686346.1 TACATGCTACAATTCTAGAACTGGTTATTATGGTTGTTGGCGCCATAGTTATACTTGTGA 18946

NC_006213.1 CACAGTTTACAATTCTAGAACTGGTTACTATGGTTGTTGGCGCCATAGTGTTACATGTGA 18606

NC_002645.1 AACATGCTACAATTCTGTTAGTAATGACTATTGTTGCTTTAAACATGCATTGGGCTGTGA 17765

JX504050.1 CACTTGTTATAATTCAGTTAGTAATGAATATTGTTGTTTTAAACATGCATTGGGTTGTGA 17684

* * * *** ** * *** * ***

NC_019843.3 TTATGTCTACAACCCTTTCTTTGTCGATGTTCAACAGTGGGGTTATGTAGGCAATCTTGC 18762

NC_004718.3 CTATGTCTATAACCCATTTATGATTGATGTTCAGCAGTGGGGCTTTACGGGTAACCTTCA 18730

EPI_ISL_6640916_omicron TTACGTCTATAATCCGTTTATGATTGATGTTCAACAATGGGGTTTTACAGGTAACCTACA 18734

MW494315.1 TTACGTCTATAATCCGTTTATGATTGATGTTCAACAATGGGGTTTTACAGGTAACCTACA 18800

KF686346.1 TTATGTGTATAATCCACTTATTGTAGATATACAACAGTGGGGTTATACAGGTTCTTTAAC 19006

NC_006213.1 TTACTTGTATAATCCACTTATTGTTGATATTCAACAGTGGGGATATATTGGTTCTTTATC 18666

NC_002645.1 CTATGTTTATAATCCATATGTCATAGATATTCAACAATGGGGTTATGTTGGTTCACTCTC 17825

JX504050.1 TTATGTTTACAATCCGTATGCTTTTGATATACAACAGTGGGGTTATGTTGGTTCCTTGAG 17744

** * ** ** ** * *** * ** ** ***** * * ** *

NC_019843.3 TACTAATCACGATCGTTATTGCTCTGTCCATCAAGGAGCTCATGTGGCTTCTAATGATGC 18822

NC_004718.3 GAGTAACCATGACCAACATTGCCAGGTACATGGAAATGCACATGTGGCTAGTTGTGATGC 18790

EPI_ISL_6640916_omicron AAGCAACCATGATCTGTATTGTCAAGTCCATGGTAATGCACATGTAGCTAGTTGTGATGC 18794

MW494315.1 AAGCAACCATGATCTGTATTGTCAAGTCCATGGTAATGCACATGTAGCTAGTTGTGATGC 18860

KF686346.1 TAGTAATCACGATATAATTTGTAATGTACATAAAGGTGCACATGTTGCGTCAGCTGATGC 19066

NC_006213.1 AAGTAATCATGATTTATATTGTAGTGTCCATAAAGGAGCACATGTTGCTTCCTCTGATGC 18726

NC_002645.1 CACTAATCACCATGCAATTTGTAATGTTCATAGAAATGAGCATGTTGCTTCTGGTGATGC 17885

JX504050.1 CCAAAACCACCACACATTCTGTAACATTCATAGAAACGAGCATGATGCCTCTGGTGATGC 17804

** ** * ** * *** * **** ** ******

NC_019843.3 AATAATGACTCGTTGTTTAGCTATTCATTCTTGTTTTATAGAACGTGTGGATTGGGATAT 18882

NC_004718.3 TATCATGACTAGATGTTTAGCAGTCCATGAGTGCTTTGTTAAGCGCGTTGATTGGTCTGT 18850

EPI_ISL_6640916_omicron AATCATGACTAGGTGTCTAGCTGTCCACGAGTGCTTTGTTAAGCGTGTTGACTGGACTAT 18854

MW494315.1 AATCATGACTAGGTGTCTAGCTGTCCACGAGTGCTTTGTTAAGCGTGTTGACTGGACTAT 18920

KF686346.1 AATTATGACTCGTTGTTTAGCAATCTATGATTGTTTTTGTAAATCTGTTAATTGGAATTT 19126

NC_006213.1 TATAATGACACGGTGTTTGGCCGTTTATGATTGCTTTTGCAATAATATTAATTGGAATGT 18786

NC_002645.1 TATTATGACTAGATGTTTGGCTGTGTATGACTGCTTTGTTAAGAATGTGGATTGGTCAAT 17945

JX504050.1 TGTTATGACACGTTGTTTGGCAGTACATGATTGTTTTGTCAAAAATGTTGATTGGACTGT 17864

* ***** * *** * ** * * ** *** * * * *** *

NC_019843.3 AGAGTATCCTTATATCTCACATGAAAAGAAATTGAATTCCTGTTGTAGAATCGTTGAGCG 18942

NC_004718.3 TGAATACCCTATTATAGGAGATGAACTGAGGGTTAATTCTGCTTGCAGAAAAGTACAACA 18910

EPI_ISL_6640916_omicron TGAATATCCTATAATTGGTGATGAACTGAAGATTAATGCGGCTTGTAGAAAGGTTCAACA 18914

MW494315.1 TGAATATCCTATAATTGGTGATGAACTGAAGATTAATGCGGCTTGTAGAAAGGTTCAACA 18980

KF686346.1 AGAGTATCCAATAATTTCTAATGAGGTCAGTATAAATACATCTTGTAGGTTATTGCAGCG 19186

NC_006213.1 GGAGTATCCCATCATTTCAAATGAGTTAAGTATTAATACCTCTTGTAGGGTCTTGCAGCG 18846

NC_002645.1 TACCTACCCTATGATAGCTAATGAAAATGCCATAAACAAGGGCGGTCGCACTGTGCAGAG 18005

JX504050.1 AACGTACCCCTTTATTGCAAATGAGAAATTTATCAATGGCTGTGGGCGTAATGTCCAGGG 17924

** ** ** **** * ** * * * *

NC_019843.3 CAACGTCGTACGTGCTGCTCTTCTTGCCGGTTCATTTGACAAAGTCTATGATATTGGCAA 19002

NC_004718.3 CATGGTTGTGAAGTCTGCATTGCTTGCTGATAAGTTTCCAGTTCTTCATGACATTGGAAA 18970

EPI_ISL_6640916_omicron CATGGTTGTTAAAGCTGCATTATTAGCAGACAAATTCCCAGTTCTTCACGACATTGGTAA 18974

MW494315.1 CATGGTTGTTAAAGCTGCATTATTAGCAGACAAATTCCCAGTTCTTCACGACATTGGTAA 19040

KF686346.1 TGTCATGCTTAAAGCTGCCATGCTATGTAATAGATACAACTTATGTTATGACATAGGCAA 19246

NC_006213.1 TGTGATTCTTAAAGCTGCCATGCTCTGCAACAGATATACTTTGTGTTATGATATTGGCAA 18906

NC_002645.1 TCATATTATGCGTGCTGCTATTAAATTGTACAACCCTAAAGCAATCCATGACATTGGTAA 18065

JX504050.1 ACATGTTGTTCGTGCAGCCTTGAAATTGTATAAACCTAGTGTTATTCATGACATTGGTAA 17984

* * * ** * * ** ** ** **

NC_019843.3 TCCTAAAGGAATTCCTATTGTTGATGACCCTGTGGTTGATTGGCATTATTTTGATGCACA 19062

NC_004718.3 TCCAAAGGCTATCAAGTGTGTGCCTCAGGCTGAAGTAGAATGGAAGTTCTACGATGCTCA 19030

EPI_ISL_6640916_omicron CCCTAAAGCTATTAAGTGTGTACCTCAAGCTGATGTAGAATGGAAGTTCTATGATGCACA 19034

MW494315.1 CCCTAAAGCTATTAAGTGTGTACCTCAAGCTGATGTAGAATGGAAGTTCTATGATGCACA 19100

KF686346.1 TCCTAAAGGTTTAGCTTG------TGTCAAAGATTATGAATTTAAATTTTATGATGCTTT 19300

NC_006213.1 CCCAAAAGCGATTGCCTG------TGTCAAAGATTTTGATTTTAAGTTCTATGATGCCCA 18960

NC_002645.1 TCCTAAGGGTATTCGTTGTG---CTGTAACTGATGCCAAGTGGTATTGTTATGACAAGAA 18122

JX504050.1 TCCTAAAGGTGTACGTTGTG---CTGTTACTGATGCCAAATGGTACTGTTATGACAAGCA 18041

** ** * * * * * * * * * **

NC_019843.3 GCCCTTGACCAGGAAGG---------TACAACAGCTTTTCTATACAGAGGACATGG---C 19110

NC_004718.3 GCCATGTAGTGACAAAGCTTACAAAATAGAGGAACTCTTCTATTCTTATGCTACACATCA 19090

EPI_ISL_6640916_omicron GCCTTGTAGTGACAAAGCTTATAAAATAGAAGAATTATTCTATTCTTATGCCACACATTC 19094

MW494315.1 GCCTTGTAGTGACAAAGCTTATAAAATAGAAGAATTATTCTATTCTTATGCCACACATTC 19160

KF686346.1 TCCTGTAGCCAAGTTTG---------TTAAACAGTTATTTTATGTCTATGATGTGCATAA 19351

NC_006213.1 ACCAATTGTTAAGTCTG---------TTAAGACTCTTTTGTATTCTTTTGAGGCACATAA 19011

NC_002645.1 CCCTATTAATTCTAATG---------TGAAAACATTGGAGTATGATTACATGACACATGG 18173

JX504050.1 ACCTGTTAATAGTAATG---------TCAAGTTGTTGGATTATGATTATGCAACCCATGG 18092

** * * * * ***

NC_019843.3 CTCAAGATTTGCTGATGGGCTCTGCTTATTTTGGAACTGTAATGTACCAAAATATCCTAA 19170

NC_004718.3 CGATAAATTCACTGATGGTGTTTGTTTGTTTTGGAATTGTAACGTTGATCGTTACCCAGC 19150

EPI_ISL_6640916_omicron TGACAAATTCACAGATGGTGTATGCCTATTTTGGAATTGCAATGTCGATAGATATCCTGC 19154

MW494315.1 TGACAAATTCACAGATGGTGTATGCCTATTTTGGAATTGCAATGTCGATAGATATCCTGC 19220

KF686346.1 AGATAATTTTAAAGATGGTTTATGTATGTTTTGGAATTGTAATGTTGATAAATATCCATC 19411

NC_006213.1 GGACTCTTTTAAAGACGGTTTGTGTATGTTTTGGAACTGTAATGTGGATAAGTATCCACC 19071

NC_002645.1 CCAAATG------GATGGCTTGTGTTTGTTTTGGAATTGTAATGTGGATATGTACCCTGA 18227

JX504050.1 TCAACTT------GATGGTCTTTGTTTATTCTGGAATTGTAATGTTGATATGTATCCAGA 18146

** ** * ** * ** ***** ** ** ** ** **

NC_019843.3 TAATGCAATTGTATGCAGGTTTGACACACGTGTGCATTCTGAGTTCAATTTGCCAGGTTG 19230

NC_004718.3 CAATGCAATTGTGTGTAGGTTTGACACAAGAGTCTTGTCAAACTTGAACTTACCAGGCTG 19210

EPI_ISL_6640916_omicron TAATTCCATTGTTTGTAGATTTGACACTAGAGTGCTATCTAACCTTAACTTGCCTGGTTG 19214

MW494315.1 TAATTCCATTGTTTGTAGATTTGACACTAGAGTGCTATCTAACCTTAACTTGCCTGGTTG 19280

KF686346.1 TAATTCAATTGTTTGTAGATTTGACACTCGAGTTTTAAATAAATTAAACCTTCCTGGATG 19471

NC_006213.1 GAATGCAGTTGTATGTAGATTTGACACTAGAGTGTTGAATAATTTAAATCTTCCTGGCTG 19131

NC_002645.1 ATTCTCAATTGTTTGCAGGTTTGACACACGTACACGATCTACATTGAACCTTGAAGGTGT 18287

JX504050.1 ATTTTCAATTGTGTGTCGTTTTGACACACGTACTCGTTCTGTTTTTAATTTAGAAGGTGT 18206

* **** ** * ******** * * ** * **

NC_019843.3 TGATGGCGGTAGTTTGTATGTTAACAAGCACGCTTTTCATACACCAGCATATGATGTGAG 19290

NC_004718.3 TGATGGTGGTAGTTTGTATGTGAATAAGCATGCATTCCACACTCCAGCTTTCGATAAAAG 19270

EPI_ISL_6640916_omicron TGATGGTGGCAGTTTGTATGTAAATAAACATGCATTCCACACACCAGCTTTTGATAAAAG 19274

MW494315.1 TGATGGTGGCAGTTTGTATGTAAATAAACATGCATTCCACACACCAGCTTTTGATAAAAG 19340

KF686346.1 TAATGGTGGTAGTTTGTATGTTAATAAACATGCATTCCATACTAATCCTTTTACTAGAAC 19531

NC_006213.1 TAATGGAGGTAGTTTGTATGTTAATAAACATGCATTCCACACTAAACCCTTTGCTAGGGC 19191

NC_002645.1 AAATGGTGGGTCATTGTATGTCAATAATCATGCATTTCACACTCCTGCTTATGATAAACG 18347

JX504050.1 TAATGGTGGTTCTCTTTATGTTAACAAACATGCGTTTCATACACCAGCATATGATAAACG 18266

**** ** * ***** ** ** ** ** ** ** ** * * *

NC_019843.3 TGCATTCCGTGATCTGAAACCTTTACCATTCTTTTATTATTCTACTACACCATGTGAAGT 19350

NC_004718.3 TGCATTTACTAATTTAAAGCAATTGCCTTTCTTTTACTATTCTGATAGTCCTTGTGAGTC 19330

EPI_ISL_6640916_omicron TGCTTTTGTTAATTTAAAACAATTACCATTTTTCTATTACTCTGACAGTCCATGTGAGTC 19334

MW494315.1 TGCTTTTGTTAATTTAAAACAATTACCATTTTTCTATTACTCTGACAGTCCATGTGAGTC 19400

KF686346.1 TGTTTTTGAAAATCTTAAGCCTATGCCTTTTTTCTATTATTCAGATACGCCTTGTGTGTA 19591

NC_006213.1 AGCCTTTGAGCATTTGAAGCCTATGCCATTCTTCTATTATTCAGATACGCCTTGTGTGTA 19251

NC_002645.1 TGCTATGGCTAAATTGAAACCAGCACCGTTTTTCTACTATGACGACGGTTCATGTGAGGT 18407

JX504050.1 TGCTTTTGTTAAATTAAAACCTATGCCCTTTTTTTACTTTGATGACAGTGATTGTGATGT 18326

* * * * ** * ** ** ** ** * ****

NC_019843.3 GCATGGTAATGGTAGTATGATAGAGGATATTGATTATGTACCCCTAAAATCTGCAGTCTG 19410

NC_004718.3 TCATGGCAAACAAGTAGTG---TCGGATATTGATTATGTTCCACTCAAATCTGCTACGTG 19387

EPI_ISL_6640916_omicron TCATGGAAAACAAGTAGTG---TCAGATATAGATTATGTACCACTAAAGTCTGCTACGTG 19391

MW494315.1 TCATGGAAAACAAGTAGTG---TCAGATATAGATTATGTACCACTAAAGTCTGCTACGTG 19457

KF686346.1 CGTAGATGGTTTAGAATCT---AAACAAGTTGATTACGTTCCTTTAAGAAGCGCCACTTG 19648

NC_006213.1 TATGGATGGCATGGATGCT---AAGCAGGTTGATTATGTACCTTTGAAATCTGCCACGTG 19308

NC_002645.1 TGTTCACG---------------ATCAAGTTAACTATGTTCCTTTGAGAGCCACTAATTG 18452

JX504050.1 TGTGCAAG---------------AACAAGTTAATTATGTACCCCTTCGCGCTAGTAGTTG 18371

* * * ** ** ** * **

NC_019843.3 TATTACAGCTTGTAATTTAGGGGGCGCTGTTTGTAGGAAGCATGCTACAGAGTACAGAGA 19470

NC_004718.3 TATTACACGATGCAATTTAGGTGGTGCTGTTTGCAGACACCATGCAAATGAGTACCGACA 19447

EPI_ISL_6640916_omicron TATAACACGTTGCAATTTAGGTGGTGCTGTCTGTAGACATCATGCTAATGAGTACAGATT 19451

MW494315.1 TATAACACGTTGCAATTTAGGTGGTGCTGTCTGTAGACATCATGCTAATGAGTACAGATT 19517

KF686346.1 TATCACACGGTGTAATCTAGGTGGAGCTGTTTGTTCAAAGCATGCTGAAGAATATTGTAA 19708

NC_006213.1 CATCACAAGATGCAATTTAGGTGGTGCAGTTTGTTTAAAACATGCTGAAGAGTATCGTGA 19368

NC_002645.1 CATTACCAAGTGTAATATTGGTGGTGCTGTATGTTCTAAGCACGCTAATCTCTATAGAGC 18512

JX504050.1 TGTTACTCGTTGTAATATAGGTGGTGCTGTTTGTTCAAAACATGCAAATTTGTATCAAAA 18431

* ** ** *** * ** ** ** ** ** * ** ** **

NC_019843.3 GTATATGGAAGCATATAATCTTGTCTCTGCATCAGGTTTCCGCCTTTGGTGTTATAAGAC 19530

NC_004718.3 GTACTTGGATGCATATAATATGATGATTTCTGCTGGATTTAGCCTATGGATTTACAAACA 19507

EPI_ISL_6640916_omicron GTATCTCGATGCTTATAACATGATGATCTCAGCTGGCTTTAGCTTGTGGGTTTACAAACA 19511

MW494315.1 GTATCTCGATGCTTATAACATGATGATCTCAGCTGGCTTTAGCTTGTGGGTTTACAAACA 19577

KF686346.1 CTACCTTGAGTCTTATAATATAGTTACTACAGCAGGCTTTACTTTTTGGGTTTATAAGAA 19768

NC_006213.1 GTACTTAGAGTCTTACAATACAGCTACTACAGCAGGTTTTACTTTTTGGGTCTATAAGAC 19428

NC_002645.1 ATATGTTGAGTCATATAACATTTTTACTCAAGCTGGTTTTAATATTTGGGTTCCTACCAC 18572

JX504050.1 ATATGTTGAGGCATATAATACATTTACACAGGCAGGTTTTAACATTTGGGTACCACATAG 18491

** * ** * ** ** * ** ** * ***

NC_019843.3 CTTTGATATTTATAATCTCTGGTCTACTTTTA------CAAAAGTTCAAGGTTTGGAAAA 19584

NC_004718.3 ATTTGATACTTATAACCTGTGGAATACATTTA------CCAGGTTACAGAGTTTAGAAAA 19561

EPI_ISL_6640916_omicron ATTTGATACTTATAACCTCTGGAACACTTTTA------CAAGACTTCAGAGTTTAGAAAA 19565

MW494315.1 ATTTGATACTTATAACCTCTGGAACACTTTTA------CAAGACTTCAGAGTTTAGAAAA 19631

KF686346.1 TTTTGATTTTTATAATTTATGGAACACTTTTA------CTACGTTACAGAGTTTAGAAAA 19822

NC_006213.1 ATTTGATTTTTATAATTTGTGGAATACGTTCA------CCAAGCTACAAAGCTTGGAGAA 19482

NC_002645.1 GTTTGATTGTTATAATTTGTGGCAGACATTCACAGAGGTCAATTTACAAGGTTTAGAGAA 18632

JX504050.1 TTTTGATGTTTATAATTTGTGGCAAATTTTTATTGAAACTAATTTACAAAGTCTTGAAAA 18551

****** ****** * *** * ** * * * ** * * ** **

NC_019843.3 CATTGCTTTTAATGTTGTTAAACAAGGCCATTTTATTGGTGTTGAGGGTGAACTACCTGT 19644

NC_004718.3 TGTGGCTTATAATGTTGTTAATAAAGGACACTTTGATGGACACGCCGGCGAAGCACCTGT 19621

EPI_ISL_6640916_omicron TGTGGCTTTTAATGTTGTAAATAAGGGACACTTTGATGGACAACAGGGTGAAGTACCAGT 19625

MW494315.1 TGTGGCTTTTAATGTTGTAAATAAGGGACACTTTGATGGACAACAGGGTGAAGTACCAGT 19691

KF686346.1 CGTAATATATAACTTGGTTAATGTTGGTCATTATGATGGACGTACAGGTGAATTACCTTG 19882

NC_006213.1 TGTTGTATATAATTTAGTCAAGACTGGTCATTATACAGGACAGGCTGGTGAAATGCCTTG 19542

NC_002645.1 CATTGCTTTTAACGTTGTTAATAAAGGTTCATTTGTTGGTGCTGATGGTGAATTACCAGT 18692

JX504050.1 TATAGCATTTAATGTTGTAAAAAAAGGGTGTTTTACTGGTGTTGATGGTGAGTTACCTGT 18611

* * *** * ** ** ** * * ** ** ** **

NC_019843.3 AGCTGTAGTCAATGATAAGATCTTCACCAAGAGTGGCGTTAATGACATTTGTATGTTTGA 19704

NC_004718.3 TTCCATCATTAATAATGCTGTTTACACAAAGGTAGATGGTATTGATGTGGAGATCTTTGA 19681

EPI_ISL_6640916_omicron TTCTATCATTAATAACACTGTTTACACAAAAGTTGATGGTGTTGATGTAGAATTGTTTGA 19685

MW494315.1 TTCTATCATTAATAACACTGTTTACACAAAAGTTGATGGTGTTGATGTAGAATTGTTTGA 19751

KF686346.1 TGCTATTATTAATGACAAAGTTGTTGTTAAGATTAATAATGTAGATACTGTTATTTTTAA 19942

NC_006213.1 TGCCATTATAAATGATAAAGTTGTGGCTAAGATCGATAAGGAGGATGTTGTCATTTTTAT 19602

NC_002645.1 AGCCATTAGTGGTGATAAAGTGTTCGTACGTGATGGTAACACTGATAATTTAGTCTTTGT 18752

JX504050.1 TGCAGTTGTTAACGACAAAGTTTTTGTTCGCTATGGCGATGTTGACAACTTGGTTTTTAC 18671

* * * * ** * ***

NC_019843.3 GAATAAAACCACTTTGCCTACTAATATAGCTTTTGAACTCTATGCTAAGCGTGCTGTACG 19764

NC_004718.3 AAATAAGACAACACTTCCTGTTAATGTTGCATTTGAGCTTTGGGCTAAGCGTAACATTAA 19741

EPI_ISL_6640916_omicron AAATAAAACAACATTACCTGTTAATGTAGCATTTGAGCTTTGGGCTAAGCGCAACATTAA 19745

MW494315.1 AAATAAAACAACATTACCTGTTAATGTAGCATTTGAGCTTTGGGCTAAGCGCAACATTAA 19811

KF686346.1 AAATAATACATCATTTCCTACTAATATAGCTGTTGAATTGTTTACAAAACGTAGTATCCG 20002

NC_006213.1 TAATAATACAACATACCCTACTAATGTGGCCGTTGAATTATTTGCCAAGCGCAGTGTTCG 19662

NC_002645.1 TAACAAAACATCACTGCCTACAAACATAGCATTTGAACTTTTTGCTAAGAGGAAGGTTGG 18812

JX504050.1 AAATAAAACAACATTGCCTACTAATGTTGCTTTTGAATTGTTTGCAAAACGAAAAATGGG 18731

** ** ** * *** ** * ** **** * * * ** * *

NC_019843.3 CTCGCATCCCGATTTCAAATTGCTACACAATTTACAAGCAGACATTTGCTACAAGTTCGT 19824

NC_004718.3 ACCAGTGCCAGAGATTAAGATACTCAATAATTTGGGTGTTGATATCGCTGCTAATACTGT 19801

EPI_ISL_6640916_omicron ACCAGTACCAGAGGTGAAAATACTCAATAATTTGGGTGTGGACATTGCTGCTAATACTGT 19805

MW494315.1 ACCAGTACCAGAGGTGAAAATACTCAATAATTTGGGTGTGGACATTGCTGCTAATACTGT 19871

KF686346.1 GCACCACCCTGAACTTAAGATTCTTAGAAATTTGAACATTGATATTTGTTGGAAGCATGT 20062

NC_006213.1 ACACCACCCAGAGCTTAAGCTCTTTAGAAATTTAAATATAGACGTGTGTTGGAAGCACGT 19722

NC_002645.1 TTTAACACCACCTCTCAGTATTCTCAAAAACCTTGGTGTTGTCGCCACATATAAGTTTGT 18872

JX504050.1 TTTAACACCACCATTGTCTATTCTCAAAAATCTCGGTGTTGTTGCTACATATAAATTTGT 18791

** * * * ** * * ** **

NC_019843.3 CCTTTGGGATTATGAACGTAGCAATATTTATGGTACTGCTACTATTGGTGTATGTAAGTA 19884

NC_004718.3 AATCTGGGACTACAAAAGAGAAGCCCCAGCACATGTATCTACAATAGGTGTCTGCACAAT 19861

EPI_ISL_6640916_omicron GATCTGGGACTACAAAAGAGATGCTCCAGCACATATATCTACTATTGGTGTTTGTTCTAT 19865

MW494315.1 GATCTGGGACTACAAAAGAGATGCTCCAGCACATATATCTACTATTGGTGTTTGTTCTAT 19931

KF686346.1 CCTGTGGGATTATGTTAAAGATAGTTTGTTTTGTAGTTCCACTTATGGTGTTTGTAAATA 20122

NC_006213.1 CATTTGGGATTATGCTAGAGAAAGTATATTTTGCAGTAATACCTATGGTGTCTGCATGTA 19782

NC_002645.1 CTTGTGGGATTATGAAGCTGAGCGTCCCTTGACAAGCTTTACTAAGTCTGTTTGTGGTTA 18932

JX504050.1 TTTATGGGATTATGAAGCTGAAAGACCTTTTACCTCATATACTAAGAGTGTATGTAAATA 18851

* ***** ** ** *** **

NC_019843.3 CACTGATATTGA------------------TGTTAATTCAGCTTTGAATATATGTTTTGA 19926

NC_004718.3 GACTGACATTGCCAAGAAACCTACTGAGAGTGCTTGTTCTTCACTTACTGTCTTGTTTGA 19921

EPI_ISL_6640916_omicron GACTGACATAGCCAAGAAACCAACTGAAACGATTTGTGCACCACTCACTGTNNTTTTTGA 19925

MW494315.1 GACTGACATAGCCAAGAAACCAACTGAAACGATTTGTGCACCACTCACTGTCTTTTTTGA 19991

KF686346.1 CACAGATTTGAA------------------GTTCATCGAAAATTTGAATATACTTTTTGA 20164

NC_006213.1 TACAGATTTAAA------------------GTTCATTGATAAATTGAATGTCCTTTTTGA 19824

NC_002645.1 TACAGACTTTGC------------------------AGAGGATGTTTGTACTTGTTACGA 18968

JX504050.1 CACTGATTTTAA------------------------TGAGGATGTTTGTGTTTGTTTTGA 18887

** ** * * * * **

NC_019843.3 CATACGCGATAATTGTTCATTGGAGAAGTTCATGTCTACTCCCAATGCCATCTTTATTTC 19986

NC_004718.3 TGGTAGAGTGGAAGGACAGGTAGACCTTTTTAGAAACGCCCGTAATGGTGTTTTAATAAC 19981

EPI_ISL_6640916_omicron TGGTAGAGTTGATGGTCAAGTAGACTTATTTAGAAATGCCCGTAATGGTGTTCTTATTAC 19985

MW494315.1 TGGTAGAGTTGATGGTCAAGTAGACTTATTTAGAAATGCCCGTAATGGTGTTCTTATTAC 20051

KF686346.1 TGGTCGTGACACTGGCGCTTTAGAAGCTTTTAGAAAAGCAAGAAATGGTGTTTTTATTAG 20224

NC_006213.1 TGGTCGTGATAATGGTGCTCTTGAAGCTTTTAAACGTTCTAATAATGGCGTTTACATTTC 19884

NC_002645.1 TAATAGTATACAAGGTTCATACGAACGTTTTACTCTGTCAACTAATGCTGTGTTATTCTC 19028

JX504050.1 CAATAGTATTCAGGGTTCGTATGAGCGTTTTACGCTTACTACGAACGCTGTTTTATTTTC 18947

* * ** ** * * ** * * *

NC_019843.3 TGATAGAAAAATCAAGAA---------------------------------ATACCCTTG 20013

NC_004718.3 AGAAGGTTCAGTCAAAGG---------------------------------TCTAACACC 20008

EPI_ISL_6640916_omicron AGAAGGTAGTGTTAAAGG---------------------------------TTTACAACC 20012

MW494315.1 AGAAGGTAGTGTTAAAGG---------------------------------TTTACAACC 20078

KF686346.1 TACTGAAAAATTAAGTAG---------------------------------GTTATCAAT 20251

NC_006213.1 CACGACAAAAGTTAAGAG---------------------------------TCTTTCGAT 19911

NC_002645.1 TGCTACTGCTGTGAAAACAGGTGGTAAGAGTTTGCCGGCTATTAAATTGAATTTTGGAAT 19088

JX504050.1 TACTGTTGTCATTAAAAA------------TTTAACACCTATAAAGTTGAATTTTGGTAT 18995

* *

NC_019843.3 TATGGTAGGTCCTGATTATGCTTACTTCAATGGTGCTATCATCCGTGATAGTGATGTTGT 20073

NC_004718.3 TTCAAAGGGACCAGCACAAGCTAGCGTCAATGGAGTCACATTAATTG---GAGAATCAGT 20065

EPI_ISL_6640916_omicron ATCTGTAGGTCCCAAACAAGCTAGTCTTAATGGAGTCACATTAATTG---GAGAAGCCGT 20069

MW494315.1 ATCTGTAGGTCCCAAACAAGCTAGTCTTAATGGAGTCACATTAATTG---GAGAAGCCGT 20135

KF686346.1 GATTAAAGGTCCGCAACGAGCTGATTTAAATGGTGTGATTGTGGATA------AAGTTGG 20305

NC_006213.1 GATAAGAGGTCCACCGCGTGCTGAATTAAATGGCGTAGTGGTGGACA------AGGTTGG 19965

NC_002645.1 GCTTAATGGTAATGCAATTGCTACTGTCAAATCA---------GAAG------ATGGTAA 19133

JX504050.1 GTTGAATGGTATGCCAGTTTCTTCTATTAAGGGT---------GATA------AAGGTGT 19040

** ** * ** *

NC_019843.3 TAAACAACCAGTGAAGTTCTACTTGTATAAGAAAGTCAATAATGA--------------- 20118

NC_004718.3 AAAAACAC------AGTTTAACTACTTTAAGAAAGTAGACGGCAT--------------- 20104

EPI_ISL_6640916_omicron AAAAACAC------AGTTCAATTATTATAAGAAAGTTGATGGTGT--------------- 20108

MW494315.1 AAAAACAC------AGTTCAATTATTATAAGAAAGTTGATGGTGT--------------- 20174

KF686346.1 AGAACTCAAAGTTGAGTTTTGGTTCGCTATGAGAAAAGATGGTGACGATGTTATCTTCAG 20365

NC_006213.1 AGACACTGATTGTGTGTTTTATTTTGCTGTGCGTAAAGAAGGTCAGGATGTCATCTTCAG 20025

NC_002645.1 CATAAAAAATATTAACTGGTTTGTTTACGTACGCAAAGATGGCAA--------------- 19178

JX504050.1 TGAAAAATTAGTTAATTGGTACATATATGTTCGTAAAAATGGTCA--------------- 19085

* *

NC_019843.3 ------------------------------------GTTTATTGATCCTACTGAGTGTAT 20142

NC_004718.3 ------------------------------------TATTCAACAGTTGCCTGAAACCTA 20128

EPI_ISL_6640916_omicron ------------------------------------TGTCCAACAATTACCTGAAACTTA 20132

MW494315.1 ------------------------------------TGTCCAACAATTACCTGAAACTTA 20198

KF686346.1 CCGAACAGACAGCCTATGCTCAAGCCATTACTGGAGCCCACAAGGTAATCTAGGTGGTAA 20425

NC_006213.1 CCAATTCGACAGCCTGGGAGTCAGCTCTAACCAGAGCCCACAAGGTAATCTGGGGAGTAA 20085

NC_002645.1 ------------------------------------ACCTGTTGATCATTATGATGGTTT 19202

JX504050.1 ------------------------------------ATTTCAAGATCACTATGATGGTTT 19109

*

NC_019843.3 TTA---------------------------------------------------CACTCA 20151

NC_004718.3 CTT---------------------------------------------------TACTCA 20137

EPI_ISL_6640916_omicron CTT---------------------------------------------------TACTCA 20141

MW494315.1 CTT---------------------------------------------------TACTCA 20207

KF686346.1 TTG---CGCGGGTAATGTCATTGGTAATGATGCTCTAACACGTTTTACTATCTTTACTCA 20482

NC_006213.1 TGGTAAACCCGGTAATGTCGGTGGTAATGATGCTCTGTCAATCTCTACTATCTTTACACA 20145

NC_002645.1 TTA---------------------------------------------------TACCCA 19211

JX504050.1 TTA---------------------------------------------------CACTCA 19118

** **

NC_019843.3 GAGTCGCTCTTGTAGTGACTTCCTACCCCTTTCTGACATGGAGAAAGACTTTCTATCTTT 20211

NC_004718.3 GAGCAGAGACTTAGAGGATTTTAAGCCCAGATCACAAATGGAAACTGACTTTCTCGAGCT 20197

EPI_ISL_6640916_omicron GAGTAGAAATTTACAAGAATTTAAACCCAGGAGTCAAATGGAAATTGATTTCTTAGAATT 20201

MW494315.1 GAGTAGAAATTTACAAGAATTTAAACCCAGGAGTCAAATGGAAATTGATTTCTTAGAATT 20267

KF686346.1 GAGTCGTGTATTGTCAAGTTTTGAACCTCGCTCAGATTTAGAACGGGATTTTATTGATAT 20542

NC_006213.1 AAGCCGTGTTATTAGCTCTTTTACATGTCGTACTGATATGGAAAAAGATTTTATAGCTTT 20205

NC_002645.1 AGGTCGTAATTTACAAGACTTTTTGCCTCGCAGCACAATGGAAGAAGACTTTTTGAACAT 19271

JX504050.1 AGGTAGGAATTTATCAGACTTTACACCAAGAAGTGATATGGAGTATGATTTTCTTAACAT 19178

* * ** * ** ** ** * *

NC_019843.3 TGATAGTGATGTTTTCATTAAGAAGTATGGCTTGGAAAACTATGCTTTTGAGCACGTAGT 20271

NC_004718.3 CGCTATGGATGAATTCATACAGCGATATAAGCTCGAGGGCTATGCCTTCGAACACATCGT 20257

EPI_ISL_6640916_omicron AGCTATGGATGAATTCATTGAACGGTATAAATTAGAAGGCTATGCCTTCGAACATATCGT 20261

MW494315.1 AGCTATGGATGAATTCATTGAACGGTATAAATTAGAAGGCTATGCCTTCGAACATATCGT 20327

KF686346.1 GGATGATAATCTGTTTATTGCTAAATATGGTTTAGAAGACTATGCATTTGATCATATAGT 20602

NC_006213.1 AGATCAAGATGTGTTTATTCAGAAGTATGGTTTGGAGGACTATGCCTTTGAACACATTGT 20265

NC_002645.1 GGATATAGGCGTGTTTATTCAAAAGTATGGTCTAGAGGATTTCAACTTCGAGCACGTTGT 19331

JX504050.1 GGATATGGGTGTTTTTATTAATAAATATGGTCTTGAGGATTTTAATTTTGAACATGTTGT 19238

* * ** ** *** * ** * ** ** ** * **

NC_019843.3 CTATGGAGACTTCTCTCATACTACGTTAGGCGGTCTTCACTTGCTTATTGGTTTATACAA 20331

NC_004718.3 TTATGGAGATTTCAGTCATGGACAACTTGGCGGTCTTCATTTAATGATAGGCTTAGCCAA 20317

EPI_ISL_6640916_omicron TTATGGAGATTTTAGTCATAGTCAGTTAGGTGGTTTACATCTACTGATTGGACTAGCTAA 20321

MW494315.1 TTATGGAGATTTTAGTCATAGTCAGTTAGGTGGTTTACATCTACTGATTGGACTAGCTAA 20387

KF686346.1 TTATGGTAGTTTTAACCATAAAGTTATAGGAGGTTTGCATTTGCTTATAGGCTTATTTCG 20662

NC_006213.1 TTATGGTAACTTCAACCAGAAGATTATTGGTGGTTTGCATTTGTTAATAGGCTTGTACCG 20325

NC_002645.1 GTATGGTGATGTTTCAAAAACTACTCTAGGCGGTTTACACTTGTTGATTTCACAAGTACG 19391

JX504050.1 ATATGGTGATGTTTCAAAAACTACATTAGGAGGTCTTCATTTGTTGATATCACAGTTTAG 19298

***** * * * ** *** * ** * * **

NC_019843.3 GAAGCAACAGGAAGGTCATATTATTATGGAAGAAATGCT---AAAAGGTAGCTCAACTAT 20388

NC_004718.3 GCGCTCACAAGATTCACCACTTAAATTAGAGGATTTTAT---CCCTATGGACAGCACAGT 20374

EPI_ISL_6640916_omicron ACGTTTTAAGGAATCACCTTTTGAATTAGAAGATTTTAT---TCCTATGGACAGTACAGT 20378

MW494315.1 ACGTTTTAAGGAATCACCTTTTGAATTAGAAGATTTTAT---TCCTATGGACAGTACAGT 20444

KF686346.1 TAGGCTAAAAAAATCTAATTTGTTAATTCAAGAGTTTTT---ACAGTATGATTCTAGTAT 20719

NC_006213.1 AAGACAGCAAACTTCCAATCTGGTTGTTCAGGAGTTTGT---TTCATATGACTCCAGCAT 20382

NC_002645.1 TCTGAGTAAAATGGGCATCTTAAAGGCAGAGGAGTTTGTGGCAGCATCTGACATAACACT 19451

JX504050.1 GCTTAGTAAAATGGGTGTTTTGAAAGCTGATGATTTTGTCACTGCTTCTGACACAACTTT 19358

* * * ** * * * *

NC_019843.3 TCATAACTATTTTATTACTGAGACTAACACAGCGGCTTTTAAGGCGGTGTGTTCTGTTAT 20448

NC_004718.3 GAAAAATTACTTCATAACAGATGCGCAAACAGGTTCATCAAAATGTGTGTGTTCTGTGAT 20434

EPI_ISL_6640916_omicron TAAAAACTATTTCATAACAGATGCGCAAACAGGTTCATCTAAGTGTGTGTGTTCTGTTAT 20438

MW494315.1 TAAAAACTATTTCATAACAGATGCGCAAACAGGTTCATCTAAGTGTGTGTGTTCTGTTAT 20504

KF686346.1 TCATTCATATTTTATTACTGATCAGGAGTGTGGTAGTAGTAAGAGTGTTTGTACAGTTAT 20779

NC_006213.1 ACACTCTTATTTTATCACTGACGAGAAGAGTGGTGGTAGTAAGAGTGTTTGCACTGTTAT 20442

NC_002645.1 CAAATGTTGTACTGTGACTTATCTTAATGATCCTAGTTCTAAGACTGTTTGTACTTACAT 19511

JX504050.1 GAGGTGCTGTACTGTTACTTATCTTAATGAACTTAGTTCAAAAGTTGTTTGTACTTATAT 19418

* * ** * * ** ** ** * **

NC_019843.3 AGATTTAAAGCTTGACGACTTTGTTATGATTTTAAAGAGTCAAGACCTTGGCGTAGTATC 20508

NC_004718.3 TGATCTTTTACTTGATGACTTTGTCGAGATAATAAAGTCACAAGATTTGTCAGTGATTTC 20494

EPI_ISL_6640916_omicron TGATTTATTACTTGATGATTTTGTTGAAATAATAAAATCCCAAGATTTATCTGTAGTTTC 20498

MW494315.1 TGATTTATTACTTGATGATTTTGTTGAAATAATAAAATCCCAAGATTTATCTGTAGTTTC 20564

KF686346.1 TGATTTATTATTAGATGATTTTGTTTCTATTGTTAAGTCATTAAATTTGAGTTGTGTTAG 20839

NC_006213.1 AGATATTTTGTTGGATGATTTTGTGGCTCTTGTTAAGTCACTTAATCTTAATTGTGTGAG 20502

NC_002645.1 GGATTTGTTGTTGGATGATTTTGTTTCTGTATTGAAGTCTTTGGATTTGACTGTTGTATC 19571

JX504050.1 GGATTTGTTGTTGGACGACTTTGTTACTATACTAAAGAGTTTAGATCTTGGTGTAATATC 19478

*** * * ** ** ***** * * ** * * *

NC_019843.3 CAAGGTTGTCAAGGTTCCTATTGACTTAACAATGATTGAGTTTATGTTATGGTGTAAGGA 20568

NC_004718.3 AAAAGTGGTCAAGGTTACAATTGACTATGCTGAAATTTCATTCATGCTTTGGTGTAAGGA 20554

EPI_ISL_6640916_omicron TAAGGTTGTCAAAGTGACTATTGACTATACAGAAATTTCATTTATGCTTTGGTGTAAAGA 20558

MW494315.1 TAAGGTTGTCAAAGTGACTATTGACTATACAGAAATTTCATTTATGCTTTGGTGTAAAGA 20624

KF686346.1 TAAAGTTGTTAATATTAATGTTGATTTTAAGGATTTTCAATTTATGTTGTGGTGTAATGA 20899

NC_006213.1 TAAGGTTGTTAATGTTAATGTTGATTTTAAAGATTTTCAGTTTATGCTTTGGTGTAACGA 20562

NC_002645.1 CAAGGTTCATGAGGTCATAATTGACAACAAACCATGGAGATGGATGCTATGGTGTAAAGA 19631

JX504050.1 TAAAGTTCATGAAGTTATTATAGATAATAAACCTTATAGGTGGATGTTGTGGTGTAAAGA 19538

** ** * * * ** * *** * ******** **

NC_019843.3 TGGACAGGTTCAAACCTTCTACCCTCGACTCCAGGCTTCTGCAGATTGGAAACCTGGTCA 20628

NC_004718.3 TGGACATGTTGAAACCTTCTACCCAAAACTACAAGCAAGTCAAGCGTGGCAACCAGGTGT 20614

EPI_ISL_6640916_omicron TGGCCATGTAGAAACATTTTACCCAAAATTACAATCTAGTCAAGCGTGGCAACCGGGTGT 20618

MW494315.1 TGGCCATGTAGAAACATTTTACCCAAAATTACAATCTAGTCAAGCGTGGCAACCGGGTGT 20684

KF686346.1 TAATAAAATTATGACTTTTTATCCTAAAATGCAAGCCACTAATGATTGGAAACCTGGCTA 20959

NC_006213.1 TGAGAAAGTTATGACTTTCTATCCTCGTTTGCAAGCTGCATCTGACTGGAAGCCTGGTTA 20622

NC_002645.1 TAATGCCGTTGCTACATTCTATCCTCAGTTGCAGAGTGCA---GAATGGAAATGCGGGTA 19688

JX504050.1 TAACCACTTGTCCACTTTTTATCCACAGTTGCAGTCTGCT---GAATGGAAGTGTGGTTA 19595

* * ** ** ** ** * ** * *** * **

NC_019843.3 TGCAATGCCATCCCTCTTTAAAGTTCAAAATGTAAACCTTGAACGTTGTGAGCTTGCTAA 20688

NC_004718.3 TGCGATGCCTAACTTGTACAAGATGCAAAGAATGCTTCTTGAAAAGTGTGACCTTCAGAA 20674

EPI_ISL_6640916_omicron TGCTATGCCTAATCTTTACAAAATGCAAAGAATGCTATTAGAAAAGTGTGACCTTCAAAA 20678

MW494315.1 TGCTATGCCTAATCTTTACAAAATGCAAAGAATGCTATTAGAAAAGTGTGACCTTCAAAA 20744

KF686346.1 TTCTATGCCTGTTTTGTATAAGTATTTGAATGTTCCATTAGAGAGAGTCTCTTTATGGAA 21019

NC_006213.1 TTCTATGCCTGTATTATATAAGTATTTGAATTCTCCAATGGAAAGAGTTAGTCTCTGGAA 20682

NC_002645.1 TTCTATGCCTGGTATTTATAAGACACAACGTATGTGCTTAGAACCATGTAATTTGTATAA 19748

JX504050.1 TGCTATGCCACAAATTTATAAGCTTCAACGTATGTGTTTGGAACCTTGTAATTTATATAA 19655

* * ***** * * ** * ** * **

NC_019843.3 TTACAAGCAATCTATTCCTATGCCTCGCGGTGTGCACATGAACATCGCTAAATATATGCA 20748

NC_004718.3 TTATGGTGAAAATGCTGTTATACCAAAAGGAATAATGATGAATGTCGCAAAGTATACTCA 20734

EPI_ISL_6640916_omicron TTATGGTGATAGTGCAACATTACCTAAAGGCATAATGATGAATGTCGCAAAATATACTCA 20738

MW494315.1 TTATGGTGATAGTGCAACATTACCTAAAGGCATAATGATGAATGTCGCAAAATATACTCA 20804

KF686346.1 TTATGGTAAACCTATTAATTTGCCTACAGGCTGTATGATGAATGTTGCTAAGTACACTCA 21079

NC_006213.1 TTATGGGAAGCCAGTTACTTTGCCTACAGGCTGTATGATGAATGTTGCTAAGTATACTCA 20742

NC_002645.1 TTATGGTGCAGGTTTGAAGTTGCCCAGTGGCATTATGTTCAATGTTGTTAAATACACTCA 19808

JX504050.1 TTATGGTGCTGGTATTAAGTTGCCTAGTGGTATAATGTTAAATGTTGTTAAATACACTCA 19715

*** * ** ** * ** * * ** ** * **

NC_019843.3 ATTGTGCCAGTATTTAAATACTTGCACATTAGCCGTGCCTGCCAATATGCGTGTTATACA 20808

NC_004718.3 ACTGTGTCAATACTTAAATACACTTACTTTAGCTGTACCCTACAACATGAGAGTTATTCA 20794

EPI_ISL_6640916_omicron ACTGTGTCAATATTTAAACACATTAACATTAGCTGTACCCTATAATATGAGAGTTATACA 20798

MW494315.1 ACTGTGTCAATATTTAAACACATTAACATTAGCTGTACCCTATAATATGAGAGTTATACA 20864

KF686346.1 ATTATGTCAGTATTTGAATACTACAACATTAGCTGTTCCTGTTAATATGCGTGTTTTACA 21139

NC_006213.1 GTTATGTCAATATCTGAATACTACAACATTAGCTGTACCTGTTAATATGCGAGTTTTGCA 20802

NC_002645.1 ATTGTGTCAATATTTTAACAGTACCACGTTATGTGTTCCTCATAATATGAGAGTGTTACA 19868

JX504050.1 GCTTTGTCAATACCTAAATAGCACTACAATGTGCGTACCTCATAATATGCGTGTTTTGCA 19775

* ** ** ** * ** * ** * ** ** ** *** * ** * **

NC_019843.3 TTTTGGCGCTGGTTCTGATAAAGGTATCGCTCCTGGTACCTCAGTTTTACGACAGTGGCT 20868

NC_004718.3 CTTTGGTGCTGGCTCTGATAAAGGAGTTGCACCAGGTACAGCTGTGCTCAGACAATGGTT 20854

EPI_ISL_6640916_omicron TTTTGGTGCTGGTTCTGATAAAGGAGTTGCACCAGGTACAGCTGTTTTAAGACAGTGGTT 20858

MW494315.1 TTTTGGTGCTGGTTCTGATAAAGGAGTTGCACCAGGTACAGCTGTTTTAAGACAGTGGTT 20924

KF686346.1 TTTAGGTGCAGGGTCTGATAAAGAAGTAGCTCCAGGTTCTGCTGTTTTAAGACAGTGGTT 21199

NC_006213.1 TTTAGGTGCAGGTTCAGAAAAAGGAGTAGCACCGGGTTCTGCAGTTCTTAGGCAGTGGTT 20862

NC_002645.1 CTTGGGTGCTGGCTCTGATTATGGTGTTGCACCAGGAACTGCTGTTCTTAAAAGGTGGTT 19928

JX504050.1 CTATGGTGCTGGTTCTGACAAAGGTGTGGCACCTGGTACAACTGTTTTAAAACGTTGGCT 19835

* ** ** ** ** ** * * * ** ** ** * * ** * *** *

NC_019843.3 TCCTACAGATGCCATTATTATAGATAATGATTTAAATGAGTTCGTGTCAGATGCTGACAT 20928

NC_004718.3 GCCAACTGGCACACTACTTGTCGATTCAGATCTTAATGACTTCGTCTCCGACGCAGATTC 20914

EPI_ISL_6640916_omicron GCCTACGGGTACGCTGCTTGTCGATTCAGATCTTAATGACTTTGTCTCTGATGCAGATTC 20918

MW494315.1 GCCTACGGGTACGCTGCTTGTCGATTCAGATCTTAATGACTTTGTCTCTGATGCAGATTC 20984

KF686346.1 ACCATCTGGTAGTATTCTTGTAGATAATGATCTAAACCCATTTGTTAGCGATAGTTTAGT 21259

NC_006213.1 GCCTGCTGGTACTATTCTTGTAGATAACGATTTATACCCATTTGTTAGTGACAGTGTCGC 20922

NC_002645.1 GCCGCACGACGCAATTGTTGTTGACAACGATGTTGTTGACTATGTGAGTGACGCTGATTT 19988

JX504050.1 ACCACCCGATGCAATAATCATTGATAATGATATCAATGATTATGTTAGTGATGCAGATTT 19895

** * * * * ** *** * * ** **

NC_019843.3 AACTTTATTTGGAGATTGTGTAACTGTACGTGTCGGCCAACAAGTGGATCTTGTTATTTC 20988

NC_004718.3 TACTTTAATTGGAGACTGTGCAACAGTACATACGGCTAATAAATGGGACCTTATTATTAG 20974

EPI_ISL_6640916_omicron AACTTTGATTGGTGATTGTGCAACTGTACATACAGCTAATAAATGGGATCTCATTATTAG 20978

MW494315.1 AACTTTGATTGGTGATTGTGCAACTGTACATACAGCTAATAAATGGGATCTCATTATTAG 21044

KF686346.1 TACTTATTTTGGAGATTGTATGACTTTACCATTTGATTGTCATTGGGATTTGATAATATC 21319

NC_006213.1 TACATATTTTGGGGATTGTATAACTTTACCCTTTGATTGTCAATGGGATTTGATAATTTC 20982

NC_002645.1 TAGTGTTACTGGTGATTGTGCAACCGTTTATTTGGAAGACAAGTTTGACTTGTTAATCTC 20048

JX504050.1 TAGCATTACAGGTGATTGTGCTACTGTTTATCTTGAAGATAAGTTTGACTTACTTATTTC 19955

* ** ** *** ** * * * ** * * **

NC_019843.3 CGACATGTATGATCCTACTACTAAGAATGTAACAGGTAGTAATGAGTCAAAGGCTTTATT 21048

NC_004718.3 CGATATGTATGACCCTAGGACCAAACATGTGACAAAAGAGAATGACTCTAAAGAAGGGTT 21034

EPI_ISL_6640916_omicron TGATATGTACGACCCTAAGACTAAAAATGTTACAAAAGAAAATGACTCTAAAGAGGGTTT 21038

MW494315.1 TGATATGTACGACCCTAAGACTAAAAATGTTACAAAAGAAAATGACTCTAAAGAGGGTTT 21104

KF686346.1 TGATATGTATGATCCTCTTACTAAAAATATTGGTGATTATAATGTGAGTAAGGATGGTTT 21379

NC_006213.1 TGATATGTATGACCCTATTACTAAGAACATAGGGGAGTACAATGTGAGTAAAGATGGTTT 21042

NC_002645.1 TGATATGTACGATGGTAGGACAAAGGCAATTGATGGTGAAAATGTTTCGAAAGAAGGATT 20108

JX504050.1 TGATATGTATGATGGTAGAATTAAATTTTGTGATGGTGAAAATGTCTCTAAAGATGGGTT 20015

** ***** ** * * ** **** ** * **

NC_019843.3 CTTTACTTACCTGTGTAACCTCATTAATAATAATCTTGCTCTTGGTGGGTCTGTTGCTAT 21108

NC_004718.3 TTTCACTTATCTGTGTGGATTTATAAAGCAAAAACTAGCCCTGGGTGGTTCTATAGCTGT 21094

EPI_ISL_6640916_omicron TTTCACTTACATTTGTGGGTTTATACAACAAAAGCTAGCTCTTGGAGGTTCCGTGGCTAT 21098

MW494315.1 TTTCACTTACATTTGTGGGTTTATACAACAAAAGCTAGCTCTTGGAGGTTCCGTGGCTAT 21164

KF686346.1 TTTTACTTACATTTGTCATTTAATTCGTGATAAATTATCTTTGGGTGGTAGTGTAGCTAT 21439

NC_006213.1 CTTTACATACATTTGTCATATGATTCGAGACAAGTTAGCTCTGGGTGGCAGTGTTGCTAT 21102

NC_002645.1 TTTCACTTACATCAATGGTTTCATTTGTGAAAAACTTGCCATCGGAGGTTCGATTGCTAT 20168

JX504050.1 TTTTACTTATCTTAATGGTGTTATTAGAGAAAAATTAGCTATTGGTGGTAGTGTTGCCAT 20075

** ** ** * * * ** * ** * * * ** ** * ** *

NC_019843.3 TAAAATAACAGAACACTCTTGGAGCGTTGAACTTTATGAACTTATGGGAAAATTTGCTTG 21168

NC_004718.3 AAAGATAACAGAGCATTCTTGGAATGCTGACCTTTACAAGCTTATGGGCCATTTCTCATG 21154

EPI_ISL_6640916_omicron AAAGATAACAGAACATTCTTGGAATGCTGATCTTTATAAGCTCATGGGACACTTCGCATG 21158

MW494315.1 AAAGATAACAGAACATTCTTGGAATGCTGATCTTTATAAGCTCATGGGACACTTCGCATG 21224

KF686346.1 AAAAATTACAGAGTTTTCTTGGAATGCTGATTTATATAAATTAATGAGTTGTTTTGCATT 21499

NC_006213.1 AAAAATAACAGAGTTTTCTTGGAATGCAGAATTATATAAGTTAATGGGGTATTTTGCATT 21162

NC_002645.1 TAAAGTAACAGAGTATAGCTGGAATAAGAAATTGTATGAACTTGTACAAAGATTTTCTTT 20228

JX504050.1 TAAGATTACAGAATATAGTTGGAATAAGTATCTTTATGAATTAATACAAAGATTTGCTTT 20135

** * ***** **** * * ** * * * ** * *

NC_019843.3 GTGGACTGTTTTCTGCACCAATGCAAATGCATCCTCATCTGAAGGATTCCTCTTAGGTAT 21228

NC_004718.3 GTGGACAGCTTTTGTTACAAATGTAAATGCATCATCATCGGAAGCATTTTTAATTGGGGC 21214

EPI_ISL_6640916_omicron GTGGACAGCCTTTGTTACTAATGTGAATGCGTCATCATCTGAAGCATTTTTAATTGGATG 21218

MW494315.1 GTGGACAGCCTTTGTTACTAATGTGAATGCGTCATCATCTGAAGCATTTTTAATTGGATG 21284

KF686346.1 TTGGACAGTTTTTTGTACTAATGTAAATGCTTCTTCTAGTGAAGGGTTTTTAATAGGTAT 21559

NC_006213.1 TTGGACTGTGTTTTGCACAAATGCAAATGCTTCTTCTAGTGAAGGATTTTTAATTGGCAT 21222

NC_002645.1 TTGGACTATGTTTTGCACTTCTGTTAATACGTCATCATCAGAAGCCTTTGTTGTCGGAAT 20288

JX504050.1 TTGGACTTTGTTTTGCACGTCTGTTAATACATCCTCTTCAGAAGCTTTTCTTATTGGTAT 20195

***** ** ** ** *** * ** ** **** ** * * **

NC_019843.3 TAATTACTTGGGTACTATTAAAGAAAAT------ATAGATGGTGGTGCTATGCACGCCAA 21282

NC_004718.3 TAACTATCTTGGCAAGCCGAAGGAACAA------ATTGATGGCTATACCATGCATGCTAA 21268

EPI_ISL_6640916_omicron TAATTATCTTGGCAAACCACGCGAACAA------ATAGATGGTTATGTCATGCATGCAAA 21272

MW494315.1 TAATTATCTTGGCAAACCACGCGAACAA------ATAGATGGTTATGTCATGCATGCAAA 21338

KF686346.1 AAATTACCTGGGTAAATCTTCTTTTGAA------ATAGATGGCAATGTTATGCATGCTAA 21613

NC_006213.1 AAATTATTTGTGTAAGCCCAAGGTTGAG------ATAGATGGAAATGTTATGCATGCCAA 21276

NC_002645.1 TAACTATCTTGGTGATTTCGCACAAGGACCTTTTATAGATGGTAACATAATACACGCAAA 20348

JX504050.1 TAATTATTTAGGTGACTTTATTCAAGGTCCTTTTATAGCTGGTAACACTGTTCATGCTAA 20255

** ** * * ** * *** * ** ** **

NC_019843.3 CTATATATTTTGGAGAAATTCCACTCCTATGAATCTGAGTACTTACTCACTTTTTGATTT 21342

NC_004718.3 CTACATTTTCTGGAGGAACACAAATCCTATCCAGTTGTCTTCCTATTCACTCTTTGACAT 21328

EPI_ISL_6640916_omicron TTACATATTTTGGAGGAATACAAATCCAATTCAGTTGTCTTCCTATTCTTTATTTGACAT 21332

MW494315.1 TTACATATTTTGGAGGAATACAAATCCAATTCAGTTGTCTTCCTATTCTTTATTTGACAT 21398

KF686346.1 CTATTTGTTTTGGAGAAATAGTACAACATGGAATGGCGGTGCTTATAGTTTATTTGATAT 21673

NC_006213.1 TTATTTGTTTTGGAGAAATTCCACAGTTTGGAACGGGGGTGCTTATAGCCTGTTTGATAT 21336

NC_002645.1 TTATGTATTTTGGCGTAACTCCACTGTTATGAGTTTGTCCTACAACTCTGTTTTAGACCT 20408

JX504050.1 TTATATATTTTGGCGTAATTCTACTATTATGTCTTTGTCATACAATTCAGTTTTAGATTT 20315

** * ** *** * ** * * * ** ** *

NC_019843.3 ATCCAAGTTTCAATTAAAATTAAAAGGAACACCAGTTCTTCAATTAAAGGAGAGTCAAAT 21402

NC_004718.3 GAGCAAATTTCCTCTTAAATTAAGAGGAACTGCTGTAATGTCTCTTAAGGAGAATCAAAT 21388

EPI_ISL_6640916_omicron GAGTAAATTTCCCCTTAAATTAAGGGGTACTGCTGTTATGTCTTTAAAAGAAGGTCAAAT 21392

MW494315.1 GAGTAAATTTCCCCTTAAATTAAGGGGTACTGCTGTTATGTCTTTAAAAGAAGGTCAAAT 21458

KF686346.1 GACTAAATTTTCTTTGAAATTGGCTGGCACTGCTGTTGTTAATTTAAGACCAGATCAATT 21733

NC_006213.1 GGCTAAATTCCCGCTTAAGTTGGCTGGTACTGCCGTAATAAATTTAAGAGCAGACCAGAT 21396

NC_002645.1 GAGTAAATTTAATTGCAAACACAAAGCGACTGTTGTTGTGCAATTAAAGGATAGTGATAT 20468

JX504050.1 AAGTAAGTTTGAATGTAAACATAAAGCCACTGTTGTTGTTACACTTAAAGATAGTGATGT 20375

** ** ** * ** ** * * * * *

NC_019843.3 TAACGAACTCGTAATATCTCTCCTGTCGCAGGGTAAGTTACTTATCCGTGACAATGATAC 21462

NC_004718.3 CAATGATATGATTTATTCTCTTCTGGAAAAAGGTAGGCTTATCATTAGAGAAAACAACAG 21448

EPI_ISL_6640916_omicron CAATGATATGATTTTATCTCTTCTTAGTAAAGGTAGACTTATAATTAGAGAAAACAACAG 21452

MW494315.1 CAATGATATGATTTTATCTCTTCTTAGTAAAGGTAGACTTATAATTAGAGAAAACAACAG 21518

KF686346.1 AAATGATTTAGTTTATTCTCTTATTGAAAGAGGTAAATTATTAGTTCGCGATACGCGTAA 21793

NC_006213.1 TAATGATATGGTTTATTCCCTTCTTGAAAAGGGTAAACTACTTATTAGAGATACAAATAA 21456

NC_002645.1 TAATGAAATGGTGCTTAGTCTTGTTAGGAGTGGTAAGTTGCTTGTAAGGGGTAATGGCAA 20528

JX504050.1 AAATGATATGGTTTTGAGTTTGATTAAGAGTGGTAGGTTGTTGTTACGCAATAATGGTCG 20435

** ** * * * * **** * * * * *

NC_019843.3 ACTCAGTGTTTCTACTGATGTTCTTGTTAACACCTACAGAAAG----------------- 21505

NC_004718.3 AGTTGTGGTTTCAAGTGATATTCTTGTTAACAACTAAACGAAC-ATGTTTATTTTCTTAT 21507

EPI_ISL_6640916_omicron AGTTGTTATTTCTAGTGATGTTCTTGTTAACAACTAAACGAACAATGTTTGTTTTTCTTG 21512

MW494315.1 AGTTGTTATTTCTAGTGATGTTCTTGTTAACAACTAAACGAACAATGTTTGTTTTTCTTG 21578

KF686346.1 AGAGATTTTTGTTGGTGATAGTCTTGT-AAATACTTGTTAGATCTCATTAA--------- 21843

NC_006213.1 AGAAGTTTTCGTTGGTGACAGTTTGGT-TAATGTAATCTAAACTTTAAAAATGGCTGTCG 21515

NC_002645.1 GTGTTTGAGTTTTAGTAATCATTTAGTCTCAACTAAATAAAATGTTTGTTTTGCTTGTTG 20588

JX504050.1 TTTTGGTGGTTTTAGTAATCATTTAGTCTCAACTAAATGAAACTTTTCTTGATTTTGCTT 20495

* * * * ** *

NC_019843.3 ------------------------------------------------------------ 21505

NC_004718.3 TATTTCTTACTCTCACTAGTGGTAGTGACCTTGACCGGTGCACCACTTTTGATGATGTTC 21567

EPI_ISL_6640916_omicron TTTTATTGCCACTAGTCTCTAGTCAGTGTGTTAATCTTACAACC---------------- 21556

MW494315.1 TTTTATTGCCACTAGTCTCTAGTCAGTGTGTTAATCTTACAACC---------------- 21622

KF686346.1 ---ATCTAAACTATGTTAATTATTTTTTTATTTTTTTATTT------------------- 21881

NC_006213.1 CTTATGCAGACAAGCCTAATCATTTTATCAATTTTCCACTTACCCATTTTCAGGGTTTTG 21575

NC_002645.1 CATATGCCTTGTTGCATATTGCTGGTTGTCA----------------------------- 20619

JX504050.1 GTTTTGCC-CCTGGCCTCTTGCTTTTTCACATGTAATAGTA------------------- 20535

NC_019843.3 ---------------------------------------------------TTA------ 21508

NC_004718.3 AAGCTCCTAATTACACTCAACATAC-----TTCATCTATGAGGGGGGTTTACTA------ 21616

EPI_ISL_6640916_omicron -AGAACTCAATTACCCCCTGCATACACTAATTCTTTCACACGTGGTGTTTATTA------ 21609

MW494315.1 -AGAACTCAATTACCCCCTGCATACACTAATTCTTTCACACGTGGTGTTTATTA------ 21675

KF686346.1 ------------------------------------------------------------ 21881

NC_006213.1 TGTTAAATTATAAAGGTTTACAATTTCAAATTCTCGATGAAGGAGTGGATTGTAAAATAC 21635

NC_002645.1 ------------------------------------------------------------ 20619

JX504050.1 ------------------------------------------------------------ 20535

NC_019843.3 ------------------------------------------------------------ 21508

NC_004718.3 ------------------------------------------------------------ 21616

EPI_ISL_6640916_omicron ------------------------------------------------------------ 21609

MW494315.1 ------------------------------------------------------------ 21675

KF686346.1 ------------------------------------------------------------ 21881

NC_006213.1 AAACAGCGCCACACATTAGTCTTACTATGCTGGACATACAGCCTGAAGACTATAAAAGTG 21695

NC_002645.1 ------------------------------------------------------------ 20619

JX504050.1 ------------------------------------------------------------ 20535

NC_019843.3 ------------------------------------------------------------ 21508

NC_004718.3 ------------------------------------------------------------ 21616

EPI_ISL_6640916_omicron ------------------------------------------------------------ 21609

MW494315.1 ------------------------------------------------------------ 21675

KF686346.1 ------------------------------------------------------------ 21881

NC_006213.1 TTGATGTCGCTATTCAAGAAGTTATTGATGATATGCATTGGGGTGATGGTTTTCAGATTA 21755

NC_002645.1 ------------------------------------------------------------ 20619

JX504050.1 ------------------------------------------------------------ 20535

NC_019843.3 ------------------------------------------------------------ 21508

NC_004718.3 ------------------------------------------------------------ 21616

EPI_ISL_6640916_omicron ------------------------------------------------------------ 21609

MW494315.1 ------------------------------------------------------------ 21675

KF686346.1 ------------------------------------------------------------ 21881

NC_006213.1 AATTTGAGAATCCTCACATCCTAGGAAGATGCATAGTTTTAGATGTTAAAGGTGTAGAAG 21815

NC_002645.1 ------------------------------------------------------------ 20619

JX504050.1 ------------------------------------------------------------ 20535

NC_019843.3 ------------------------------------------------------------ 21508

NC_004718.3 ------------------------------------------------------------ 21616

EPI_ISL_6640916_omicron ------------------------------------------------------------ 21609

MW494315.1 ------------------------------------------------------------ 21675

KF686346.1 ------------------------------------------------------------ 21881

NC_006213.1 AATTGCATGACGATTTAGTTAATTACATTCGTGATAAAGGTTGTGTTGCTGACCAATCCA 21875

NC_002645.1 ------------------------------------------------------------ 20619

JX504050.1 ------------------------------------------------------------ 20535

NC_019843.3 ------------------------------------------------------------ 21508

NC_004718.3 ------------------------------------------------------------ 21616

EPI_ISL_6640916_omicron ------------------------------------------------------------ 21609

MW494315.1 ------------------------------------------------------------ 21675

KF686346.1 ------------------------------------------------------------ 21881

NC_006213.1 GGAAATGGATTGGCCATTGCACCATAGCTCAACTCACGGATGCAGCACTGTCCATTAAGG 21935

NC_002645.1 ------------------------------------------------------------ 20619

JX504050.1 ------------------------------------------------------------ 20535

NC_019843.3 ------------------------------------------------------------ 21508

NC_004718.3 ------------------------------------------------------------ 21616

EPI_ISL_6640916_omicron ------------------------------------------------------------ 21609

MW494315.1 ------------------------------------------------------------ 21675

KF686346.1 ------------------------------------------------------------ 21881

NC_006213.1 AAAATGTTGATTTTATAAACAGCATGCAATTCAATTATAAAATCACCATCAACCCCTCAT 21995

NC_002645.1 ------------------------------------------------------------ 20619

JX504050.1 ------------------------------------------------------------ 20535

NC_019843.3 ------------------------------------------------------------ 21508

NC_004718.3 ------------------------------------------------------------ 21616

EPI_ISL_6640916_omicron ------------------------------------------------------------ 21609

MW494315.1 ------------------------------------------------------------ 21675

KF686346.1 ------------------------------------------------------------ 21881

NC_006213.1 CACCGGCTAGACTTGAAATAGTTAAGCTCGGTGCTGAAAAGAAAGATGGTTTTTATGAAA 22055

NC_002645.1 ------------------------------------------------------------ 20619

JX504050.1 ------------------------------------------------------------ 20535

NC_019843.3 ------------------------------------------------------------ 21508

NC_004718.3 ------------------------------------------------------------ 21616

EPI_ISL_6640916_omicron ------------------------------------------------------------ 21609

MW494315.1 ------------------------------------------------------------ 21675

KF686346.1 ------------------------------------------------------------ 21881

NC_006213.1 CCATAGTTAGTCACTGGATGGGAATTCGTTTTGAATACACATCACCCACTGATAAGCTAG 22115

NC_002645.1 ------------------------------------------------------------ 20619

JX504050.1 ------------------------------------------------------------ 20535

NC_019843.3 ------------------------------------------------------------ 21508

NC_004718.3 ------------------------------------------------------------ 21616

EPI_ISL_6640916_omicron ------------------------------------------------------------ 21609

MW494315.1 ------------------------------------------------------------ 21675

KF686346.1 ------------------------------------------------------------ 21881

NC_006213.1 CTATGATTATGGGTTATTGTTGTTTAGATGTGGTACGTAAAGAGCTAGAAGAAGGCGATC 22175

NC_002645.1 ------------------------------------------------------------ 20619

JX504050.1 ------------------------------------------------------------ 20535

NC_019843.3 ------------------------------------------------------------ 21508

NC_004718.3 ------------------------------------------------------------ 21616

EPI_ISL_6640916_omicron ------------------------------------------------------------ 21609

MW494315.1 ------------------------------------------------------------ 21675

KF686346.1 ------------------------------------------------------------ 21881

NC_006213.1 TTCCCGAGAATGATGATGATGCTTGGTTTAAGCTATCGTACCATTATGAAAACAATTCTT 22235

NC_002645.1 ------------------------------------------------------------ 20619

JX504050.1 ------------------------------------------------------------ 20535

NC_019843.3 ------------------------------------------------------------ 21508

NC_004718.3 ------------------------------------------------------------ 21616

EPI_ISL_6640916_omicron ------------------------------------------------------------ 21609

MW494315.1 ------------------------------------------------------------ 21675

KF686346.1 ------------------------------------------------------------ 21881

NC_006213.1 GGTTCTTCCGACATGTCTACAGGAAAAGTTTTCATTTCCGTAAGGCTTGTCAAAATTTAG 22295

NC_002645.1 ------------------------------------------------------------ 20619

JX504050.1 ------------------------------------------------------------ 20535

NC_019843.3 ------------------------------------------------------------ 21508

NC_004718.3 ------------------------------------------------------------ 21616

EPI_ISL_6640916_omicron ------------------------------------------------------------ 21609

MW494315.1 ------------------------------------------------------------ 21675

KF686346.1 ------------------------------------------------------------ 21881

NC_006213.1 ATTGTAATTGTTTGGGGTTTTATGAATCTTCAGTTGAAGAATATTAAACTCAGTGAAAAT 22355

NC_002645.1 ------------------------------------------------------------ 20619

JX504050.1 ------------------------------------------------------------ 20535

NC_019843.3 ------------------------------------------------------------ 21508

NC_004718.3 ------------------------------------------------------------ 21616

EPI_ISL_6640916_omicron ------------------------------------------------------------ 21609

MW494315.1 ------------------------------------------------------------ 21675

KF686346.1 ---------------------------------------------CTGTTATGGTTTTAA 21896

NC_006213.1 GTTTTTGCTTCCTAGATTTATTCTAGTTAGCTGCATAATTGGTAGCTTAGGTTTTTACAA 22415

NC_002645.1 ------------------------------------------------------------ 20619

JX504050.1 ------------------------------------------------------------ 20535

NC_019843.3 ------------------------------------------------------------ 21508

NC_004718.3 ------------------------------------------------------------ 21616

EPI_ISL_6640916_omicron ------------------------------------------------------------ 21609

MW494315.1 ------------------------------------------------------------ 21675

KF686346.1 TGAACCTCTTAATGTTGTGTCTCATTTAAACCATGACTGGTTTTTATTTGGTGATAGTCG 21956

NC_006213.1 CCCTCCTACCAATGTTGTTTCGCATGTAAATGGAGATTGGTTTTTATTTGGTGACAGTCG 22475

NC_002645.1 ------------------------------------------------------------ 20619

JX504050.1 ------------------------------------------------------------ 20535

NC_019843.3 ------------------------------------------------------------ 21508

NC_004718.3 ------------------------------------------------------------ 21616

EPI_ISL_6640916_omicron ------------------------------------------------------------ 21609

MW494315.1 ------------------------------------------------------------ 21675

KF686346.1 TTCTGATTGTAACCATATTAATAATTTAAAAATTAAAAATTTTGATTATTTGGATATTCA 22016

NC_006213.1 TTCAGATTGTAATCATATTGTTAATATCAACCCCCATAATTATTCTTATATGGACCTTAA 22535

NC_002645.1 ------------------------------------------------------------ 20619

JX504050.1 ------------------------------------------------------------ 20535

NC_019843.3 ------------------------------------------------------------ 21508

NC_004718.3 ------------------------------------------------------------ 21616

EPI_ISL_6640916_omicron ------------------------------------------------------------ 21609

MW494315.1 ------------------------------------------------------------ 21675

KF686346.1 CCCTAGTTTGTGCAACAATGGTAAGATTTCATCTAGTGCCGGTGATTCTATTTTTAAGAG 22076

NC_006213.1 TCCTGTTCTGTGTGATTCTGGTAAAATATCATCTAAAGCTGGCAACTCCATTTTTAGGAG 22595

NC_002645.1 ------------------------------------------------------------ 20619

JX504050.1 ------------------------------------------------------------ 20535

NC_019843.3 ------------------------------------------------------------ 21508

NC_004718.3 ------------------------------------------------------------ 21616

EPI_ISL_6640916_omicron ------------------------------------------------------------ 21609

MW494315.1 ------------------------------------------------------------ 21675

KF686346.1 TTTTCATTTCACTCGATTTTATAATTACACTGGCGAAGGTGATCAAATTATTTTTTATGA 22136

NC_006213.1 TTTTCACTTTACCGATTTTTATAATTACACAGGCGAAGGTCAACAAATTATTTTTTATGA 22655

NC_002645.1 ------------------------------------------------------------ 20619

JX504050.1 ------------------------------------------------------------ 20535

NC_019843.3 ------------------------------------------------------------ 21508

NC_004718.3 ------------------------------------------------------------ 21616

EPI_ISL_6640916_omicron ------------------------------------------------------------ 21609

MW494315.1 ------------------------------------------------------------ 21675

KF686346.1 GGGTGTTAATTTTAATCCTTATCATAGATTTAAGTGTTTTCCTAATGGTAGTAATGATGT 22196

NC_006213.1 GGGTGTTAATTTTACGCCTTATCATGCCTTTAAATGCAACCGTTCTGGTAGTAATGATAT 22715

NC_002645.1 ------------------------------------------------------------ 20619

JX504050.1 ------------------------------------------------------------ 20535

NC_019843.3 ------CGTTGATGTAGGGCCAGATTCTGTTAAGTCTGCTTGTATTGAGGTTGATATAC- 21561

NC_004718.3 ------TCCTGATGAAATTTTTAGATCAGACACTCTTTATTTAACTCAGGATTTATTTC- 21669

EPI_ISL_6640916_omicron ------CCCTGACAAAGTTTTCAGATCCTCAGTTTTACATTCAACTCAGGACTTGTTCT- 21662

MW494315.1 ------CCCTGACAAAGTTTTCAGATCCTCAGTTTTACATTCAACTCAGGACTTGTTCT- 21728

KF686346.1 ATGGCTTCTTAACAAGGTAAGATTTTATCGTGCCTTATATTCTAATATGGCCTTTTTTCG 22256

NC_006213.1 TTGGATGCAGAATAAAGGCTTGTTTTATACTCAGGTTTATAAGAATATGGCTGTGTATCG 22775

NC_002645.1 ------------------------------------------------------------ 20619

JX504050.1 ------------------------------------------------------------ 20535

NC_019843.3 --AACAGACTTTCTTTGATAAAACTTGGCCTAGGCCAATTGATGTTTCTAAGGCTGACGG 21619

NC_004718.3 --TTCCATTTTATTCTAATGTTACAGGGTTTCATACTAT--------------------- 21706

EPI_ISL_6640916_omicron --TACCTTTCTTTTCCAATGTTACTTGGTTCCATGTTA------TCTCTGGGACCAATGG 21714

MW494315.1 --TACCTTTCTTTTCCAATGTTACTTGGTTCCATGCTATACATGTCTCTGGGACCAATGG 21786

KF686346.1 TTATCTTACTTTTGTTGATATTCC------TTATAATGTTTCTCTTTCTAAGTTTAATTC 22310

NC_006213.1 CAGCCTTACTTTTGTTAATGTACCATATGTTTATAATGGCTCCGCACAAGCTACAGCTCT 22835

NC_002645.1 ------------------------------------------------------------ 20619

JX504050.1 --------------------------------------------------ATGCTAATCT 20545

NC_019843.3 TATTATATACCCTCAAGGCCGTACATATTCTAACATAACTATCACTTATCAAGGTCTTTT 21679

NC_004718.3 TA---------ATCATACGTTTGGCAACCCTGTCATACCTTTTAAGGATGGTATTTATTT 21757

EPI_ISL_6640916_omicron TA---------CTAAGAGGTTTGATAACCCTGTCCTACCATTTAATGATGGTGTTTATTT 21765

MW494315.1 TA---------CTAAGAGGTTTGATAACCCTGTCCTACCATTTAATGATGGTGTTTATTT 21837

KF686346.1 TTGTAAAAGTGATATTTTATCACTTAACAATCCTATTTTTAT------------------ 22352

NC_006213.1 TTGTAAATCTGGTAGTTTAGTCCTTAATAACCCTGCATATATAGCTCCTCAAGCTAACTC 22895

NC_002645.1 ------------------------------------------------------------ 20619

JX504050.1 CTCTATGTTACAATTAGGTGTTCCTGACAATTCTTCAACTAT------------------ 20587

NC_019843.3 TCCCTATCAGGGAGACCATGGTGATATGTATGTTTACTCTGCAGGACATGCTACAGGCAC 21739

NC_004718.3 TGCTGCCACAGAGAAATCAAATGTTGTCCGTGGTTGGGTTTTTGGTTCTACCATGAACAA 21817

EPI_ISL_6640916_omicron TGCTTCCATTGAGAAGTCTAACATAATAAGAGGCTGGATTTTTGGTACTACTTTAGATTC 21825

MW494315.1 TGCTTCCACTGAGAAGTCTAACATAATAAGAGGCTGGATTTTTGGTACTACTTTAGATTC 21897

KF686346.1 ---------TAATTATTCTAAGGAAGTTTATTTTACTTTATTAGGTTGTTCTCTTTATTT 22403

NC_006213.1 TGGGGATTATTATTATAAGGTTGAAGCTGATTTTTATTTGTCAGGTTGTGACGAGTATAT 22955

NC_002645.1 ------------------------------------------------------------ 20619

JX504050.1 ------------------------------------------------------------ 20587

NC_019843.3 AACTCCACAAAAGTTGTTTGTAGCTAACTATTCTCAGGACGTCAAACAGTTTGCTAATGG 21799

NC_004718.3 CAAGTCACAGTCGGTGATTATTATTAACAATTCTA------------------------- 21852

EPI_ISL_6640916_omicron GAAGACCCAGTCCCTACTTATTGTTAATAACGCTA------------------------- 21860

MW494315.1 GAAGACCCAGTCCCTACTTATTGTTAATAACGCTA------------------------- 21932

KF686346.1 AGTACCGCTTTGCCTTTTTAAATCTAACTT---------------------------TAG 22436

NC_006213.1 CGTACCACTTTGTATTTTTAACGGCAAGTTTTTGTCGAATACAAAGTATTATGATGATAG 23015

NC_002645.1 ------------------------------------------------------------ 20619

JX504050.1 ------------------------------------------------------------ 20587

NC_019843.3 ---GTTTGTCGTCCGTATAGGAGCAGCTGCCAATTCCACTGGCACTGTTATTATTAGCCC 21856

NC_004718.3 ------------------------------------------------------------ 21852

EPI_ISL_6640916_omicron ------------------------------------------------------------ 21860

MW494315.1 ------------------------------------------------------------ 21932

KF686346.1 TCAGTACTATTATAACATAGATACTGGCTCTGTTTATGGTTTT------------TCTAA 22484

NC_006213.1 TCAATATTATTTTAATAAAGACACTGGTGTTATTTATGGTCTCAATTCTACAGAAACCAT 23075

NC_002645.1 ------------------------------------------------------------ 20619

JX504050.1 ------------------------------------------------------------ 20587

NC_019843.3 ATCTACCAGCGCTACTATACGAAAAATTTACCCTGCTTTTATGCTGGGTTCTTCAGTTGG 21916

NC_004718.3 ------------------------------------------------------------ 21852

EPI_ISL_6640916_omicron ------------------------------------------------------------ 21860

MW494315.1 ------------------------------------------------------------ 21932

KF686346.1 TGTTGTTTATCCTGATTTAGACTGTATTTATATTTCTCTTAAACCAGGTTCTT-ATAAAG 22543

NC_006213.1 TACCACTGGTTTTGATCTTAATTGTTATTATTTAGTTTTACCCTCTGGTAATT-ATTTAG 23134

NC_002645.1 ------------------------------------------------------------ 20619

JX504050.1 ------------------------------------------------------------ 20587

NC_019843.3 TAATTTCTCAGATGGTAAAATGGGCCGCTTCTTCAATCATACTCTAGTTCTTTTGCCCGA 21976

NC_004718.3 ------------------------------------------------------------ 21852

EPI_ISL_6640916_omicron ------------------------------------------------------------ 21860

MW494315.1 ------------------------------------------------------------ 21932

KF686346.1 TTTCCACCACTGCACCTTTTTTATCCTTACCTACTAAAGCTCTCTGTTTTGATAAATCTA 22603

NC_006213.1 CCATTTCAAATGAGCTATTGTTAACTGTTCCTACGAAAGCAATCTGTCTTAATAAGCGTA 23194

NC_002645.1 ------------------------------------------------------------ 20619

JX504050.1 ------------------------------------------------------------ 20587

NC_019843.3 TGGATGTGGCACTTT----ACTTAGAGCTTTTTATTGTATTCTAGAGCCTCGCTCTGGAA 22032

NC_004718.3 ------------------------------------------------------------ 21852

EPI_ISL_6640916_omicron ------------------------------------------------------------ 21860

MW494315.1 ------------------------------------------------------------ 21932

KF686346.1 AACAATTTGTACCTGTACAGGTTGTTGATTCTAGATGGAACAACGAGCGTGCCTCAGATA 22663

NC_006213.1 AGGATTTTACGCCTGTACAGGTTGTTGATTCGCGGTGGAACAATGCCAGGCAGTCTGATA 23254

NC_002645.1 ------------------------------------------------------------ 20619

JX504050.1 ----TGTTACGGGTTTATTGCCAACTCATTGGTTTTGTGCTAATCAGAGTACATCTGTTT 20643

NC_019843.3 ATCATTGTCCTGCTGGC------AATTCCTATACTTCTTTTGCCACTTATCACACTCCTG 22086

NC_004718.3 ------CTAATGTTGTT------ATACGAGCATGTAACTTTGAATTGTGTGACAACCCTT 21900

EPI_ISL_6640916_omicron ------CTAATGTTGTT------ATTAAAGTCTGTGAATTTCAATTTTGTAATGATCCAT 21908

MW494315.1 ------CTAATGTTGTT------ATTAAAGTCTGTGAATTTCAATTTTGTAATGATCCAT 21980

KF686346.1 TTTCTTTATCTGTTGCATGTCAATTGCCATATTGTTATTTTCGCAATTCTTCTGCTAATT 22723

NC_006213.1 ACATGACGGCGGTTGCTTGTCAACCTCCGTACTGTTATTTTCGTAATTCTACTACCAACT 23314

NC_002645.1 ------------------------------------------------------------ 20619

JX504050.1 ACTCAGCCAATGGTTTCTTTTATATTGATGTTGGTAATCACCGTAGTGCTTTTGCGCTCC 20703

NC_019843.3 CAACAGATTGTTCTGATGGCAATTACAATCGTAATGCCAGTCT----------------- 22129

NC_004718.3 TCTTTGCTGTTTCTAAACCCA--------------TGGGTACA----------------- 21929

EPI_ISL_6640916_omicron TTTTGG---------ACCACAAAAACAACAAAAGTTGGATGGA----------------- 21942

MW494315.1 TTTTGGGTGTTTATTACCACAAAAACAACAAAAGTTGGATGGA----------------- 22023

KF686346.1 ATGTTGGCAAGTATGATATTAACCACGGTGATAGTGGTTTTATTTCTATTTTATCTGGTC 22783

NC_006213.1 ATGTTGGTGTTTATGATATTAATCATGGAGATGCTGGTTTTACTAGCATACTTAGTGGTT 23374

NC_002645.1 ------------------------------------------------------------ 20619

JX504050.1 ATACTGGTTATTATGATGCTAATCAGTATTATATTTATGTTAC----------------- 20746

NC_019843.3 ------------------------------------------------------------ 22129

NC_004718.3 ------------------------------------------------------------ 21929

EPI_ISL_6640916_omicron ------------------------------------------------------------ 21942

MW494315.1 ------------------------------------------------------------ 22023

KF686346.1 TTTTATATAATGTTTCTTGTATTTCATATTATGGTGTATTTCTATATGATAATTTTACAT 22843

NC_006213.1 TGTTATATAATTCACCTTGTTTTTCGCAGCAAGGCGTTTTTAGGTATGATAATGTTAGCA 23434

NC_002645.1 ------------------------------------------------------------ 20619

JX504050.1 -------TAATGAAATAGGCTTAAATGCTTCTGTTACTCTTAAGATTTGTAAGTTTAGTA 20799

NC_019843.3 ------------------------------------------------------------ 22129

NC_004718.3 ------------------------------------------------------------ 21929

EPI_ISL_6640916_omicron ------------------------------------------------------------ 21942

MW494315.1 ------------------------------------------------------------ 22023

KF686346.1 CCATTTGGCCCTATTATTCTTTTGGTAGGTGTCCTACATCTTCTATTATTAAACAT---- 22899

NC_006213.1 GTGTCTGGCCTCTCTACCCCTATGGCAGATGTCCCACTGCTGCTGATATTAATATCCCTG 23494

NC_002645.1 ------------------------------------------------------------ 20619

JX504050.1 GAAACACTACTTTTGATTTTTTAAGTAATGCTTCTAGTTCTTTTGACTGTATAGTT---- 20855

NC_019843.3 ------------------------------------------------------------ 22129

NC_004718.3 ------------------------------------------------------------ 21929

EPI_ISL_6640916_omicron ------------------------------------------------------------ 21942

MW494315.1 ------------------------------------------------------------ 22023

KF686346.1 -----CCAATTTGTGTTTATGATTTTTTGCCTATTATTTTACAAGGTATTTTATTATGTT 22954

NC_006213.1 ATTTACCCATTTGTGTGTATGATCCGCTACCAGTTATTTTGCTTGGCATTCTTTTGGGCG 23554

NC_002645.1 ------------------------------------------------------------ 20619

JX504050.1 --------------------AATTTGTTATTTACAGAACAGTTAGGTGCGCCTTTGGGCA 20895

NC_019843.3 ------------------------------------------------------------ 22129

NC_004718.3 ------------------------------------------------------------ 21929

EPI_ISL_6640916_omicron ------------------------------------------------------------ 21942

MW494315.1 ------------------------------------------------------------ 22023

KF686346.1 TAGCTTTACTTTTTGTTGTTTTTCTATTATTCTTGTTATATAACGATAGATCTCATTA-- 23012

NC_006213.1 TTGCGATTGTAATTATTGTAGTTTTGTTGTTATATTTTATGGTGGATAATGTTACTAGGC 23614

NC_002645.1 ------------------------------------------------------------ 20619

JX504050.1 TAACTATATCTGGTGAAACTGTGCGTCTGCATTTATATAATGTAACTCGTACTTTTTATG 20955

NC_019843.3 ------------------------------------------------------------ 22129

NC_004718.3 ------------------------------------------------------------ 21929

EPI_ISL_6640916_omicron ------------------------------------------------------------ 21942

MW494315.1 ------------------------------------------------------------ 22023

KF686346.1 -------------------AATCTAAACATGTTATTAATTATTTTTATT---TTGCCTAC 23050

NC_006213.1 TGCATGATGCTTAGACCATAATCTAAACATGTTTTTGATACTTTTAATTTCCTTACCAAC 23674

NC_002645.1 ------------------------------------------------------------ 20619

JX504050.1 TGCCAGCAGCTTAT--------------------------------------AAACTTAC 20977

NC_019843.3 ------------------------------------------------------------ 22129

NC_004718.3 ------------------------------------------------------------ 21929

EPI_ISL_6640916_omicron ------------------------------------------------------------ 21942

MW494315.1 ------------------------------------------------------------ 22023

KF686346.1 AACATTAGCTGTTATAGGTGATTTTAATTGTACTAATTTTGCTATTAATGATAAAAACAC 23110

NC_006213.1 GGCTTTTGCTGTTATAGGAGATTTAAAGTGTACTTCAGATAATATTAATGATAAAGACAC 23734

NC_002645.1 ------------------------------------------------------------ 20619

JX504050.1 TAAACTTAGTGTTAAATGTTACTTTAACTATTCCTGTGTTTTTAGTGTTGTCAACGCCAC 21037

NC_019843.3 ------------------GAACTCTTTTAAGGAGTATTT--TAATTTACGTAACTGCACC 22169

NC_004718.3 ------------------CAGACACATACTATGATATTCGATAATGCATTTAATTGCACT 21971

EPI_ISL_6640916_omicron ------------------AAGTGAGTTCAGAGTTTATTC--TAGTGCGAATAATTGCACT 21982

MW494315.1 ------------------AAGTGAGTTCAGAGTTTATTC--TAGTGCGAATAATTGCACT 22063

KF686346.1 CACAGTTCCTCGCATAAG------------------------------------------ 23128

NC_006213.1 CGGTCCTCCTCCTATAAG------------------------------------------ 23752

NC_002645.1 ------------------AACTACAAATGGGCTGAACAC-----------TAGTTACTCT 20650

JX504050.1 CGTTACTGTGAATGTCACCACACATAATGGCCGTGTAGT-----------TAACTACACT 21086

NC_019843.3 TTTATGTACACTTATAACATTACCGAAGATGAGATTTTAGAGTGGTTTGGCATTACACAA 22229

NC_004718.3 TTCGAGTACATATCTGATGCCTTTTCGCTTGATGTTTCAGAAAAGTCAGGTAATTTTAAA 22031

EPI_ISL_6640916_omicron TTTGAATATGTCTCTCAGCCTTTTCTTATGGACCTTGAAGGAAAACAGGGTAATTTCAAA 22042

MW494315.1 TTTGAATATGTCTCTCAGCCTTTTCTTATGGACCTTGAAGGAAAACAGGGTAATTTCAAA 22123

KF686346.1 -----------------TGAGTATGTTGTGGATGTTTCTTATGGTTTGGGTACATATTAT 23171

NC_006213.1 -----------------TACTGATACTGTTGATGTTACTAATGGTTTGGGTACTTATTAT 23795

NC_002645.1 GTTTGCAACGGCTGTGTTGGTTATTCAGAAAATGTATTTGCTGTTGAGAGTGGTGGTTAT 20710

JX504050.1 GTTTGTGATGATTGTAATGGTTATACTGATAACATATTTTCTGTTCAACAGGATGGCCGC 21146

* *

NC_019843.3 ACTGCTCAAGGTGTTCACCTCTTCT----------------------------------- 22254

NC_004718.3 CACTTACGAGAGTTTGTGTTTAAAAATAAAGATGGGTT---------------------- 22069

EPI_ISL_6640916_omicron AATCTTAGGGAATTTGTGTTTAAGAATATTGATGGTTA---------------------- 22080

MW494315.1 AATCTTAGGGAATTTGTGTTTAAGAATATTGATGGTTA---------------------- 22161

KF686346.1 ATACTTGATCGTGTTTATTTAAATACTACTATATTATT---TACTGGTTATTTCCCTAAA 23228

NC_006213.1 GTTTTAGATCGTGTGTATTTAAATACTACGTTGTTTCT---TAATGGTTATTACCCTACT 23852

NC_002645.1 ATACCCTCCGACTTTGCATTCAATAATTGGTTCCTTCTAACTAATACCTCATCTGTTGTA 20770

JX504050.1 ATTCCTAATGGTTTCCCTTTTAATAATTGGTTTTTGTTAACTAATGGTTCCACACTAGTA 21206

* *

NC_019843.3 ------------------------------------------------------------ 22254

NC_004718.3 ------------------------------------------------------------ 22069

EPI_ISL_6640916_omicron ------------------------------------------------------------ 22080

MW494315.1 ------------------------------------------------------------ 22161

KF686346.1 TCTGGTGCCAATTTTAGGGATCTATCTTTAAAAGGTACTACATATTTGAGTACTCTTTGG 23288

NC_006213.1 TCAGGTTCCACATATCGTAATATGGCACTGAAGGGAAGTGTACTATTGAGCAGACTATGG 23912

NC_002645.1 GATGGTGTTGTGAGGAGTTTTCAGCCTTTGTTGCTTAATTGCTTATGGTCTGTTTCTGGC 20830

JX504050.1 GACGGGGTCTCTAGACTTTATCAACCACTCCGTTTAACTTGTTTATGGCCTGTACCTGGT 21266

NC_019843.3 ------------------------------------------------------------ 22254

NC_004718.3 ------------------------------------------------------------ 22069

EPI_ISL_6640916_omicron ------------------------------------------------------------ 22080

MW494315.1 ------------------------------------------------------------ 22161

KF686346.1 TATCAGAAACCCTTTTTATCTGATTTTAATAATGGTATTTTTTCTAGAGTTAAGAATACT 23348

NC_006213.1 TTTAAACCACCATTTCTTTCTGATTTTATTAATGGTATTTTTGCTAAGGTCAAAAATACC 23972

NC_002645.1 TTGCGGTTTACTACTGGTTTTGTCTATTTTAATGGTACTGGGAGAGGTG---A------- 20880

JX504050.1 CTTAAATCTTCAACTGGTTTTGTTTATTTTAATGCCACTGGTTCTGATGTTAA------- 21319

NC_019843.3 ------------------------------------------------------------ 22254

NC_004718.3 ------------------------------------------------------------ 22069

EPI_ISL_6640916_omicron ------------------------------------------------------------ 22080

MW494315.1 ------------------------------------------------------------ 22161

KF686346.1 AAGTTGTATGTTAATAAAACTTTGTATAGTGAGTTTAGTACTATAGTTATAGGTAGTGTT 23408

NC_006213.1 AAGGTTATTAAAGATCGTGTAATGTATAGTGAGTTCCCTGCTATAACTATAGGTAGTACT 24032

NC_002645.1 ------------------------------------------------------------ 20880

JX504050.1 ------------------------------------------------------------ 21319

NC_019843.3 ------------------------------------------------------------ 22254

NC_004718.3 ------------------------------------------------------------ 22069

EPI_ISL_6640916_omicron ------------------------------------------------------------ 22080

MW494315.1 ------------------------------------------------------------ 22161

KF686346.1 TTTATTAACAACTCTTATACTATTGTTGTTCAACCTCATA-------------------- 23448

NC_006213.1 TTTGTAAATACATCCTATAGTGTGGTAGTACAACCACGTACAATCAATTCAACACAGGAT 24092

NC_002645.1 ------------------------------------------------------------ 20880

JX504050.1 ------------------------------------------------------------ 21319

NC_019843.3 ------------------------------------------------------------ 22254

NC_004718.3 ------------------------------------------------------------ 22069

EPI_ISL_6640916_omicron ------------------------------------------------------------ 22080

MW494315.1 ------------------------------------------------------------ 22161

KF686346.1 ----------------ATGGTGTTTTGGAGATTACAGCTTGTCAATACACTATGTGTGAG 23492

NC_006213.1 GGTGATAATAAATTACAAGGTCTTTTAGAGGTCTCTGTTTGCCAGTATAATATGTGCGAG 24152

NC_002645.1 ------------------------------------------------------------ 20880

JX504050.1 ------------------------------------------------------------ 21319

NC_019843.3 ------------------------------------------------------------ 22254

NC_004718.3 ------------------------------------------------------------ 22069

EPI_ISL_6640916_omicron ------------------------------------------------------------ 22080

MW494315.1 ------------------------------------------------------------ 22161

KF686346.1 TATCCTCATACTATTTGTAAATCTAAA---GGTAGTTCTCGTAATGAATCTTGGCATTTT 23549

NC_006213.1 TACCCACAAACGATTTGTCATCCTAACCTGGGTAATCATCGCAAAGAACTATGGCATTTG 24212

NC_002645.1 ------------------------------------------------------------ 20880

JX504050.1 ------------------------------------------------------------ 21319

NC_019843.3 ------------------------------------------------------------ 22254

NC_004718.3 -----------------------------------------TCTCTATGTTTATAAGGGC 22088

EPI_ISL_6640916_omicron -----------------------------------------TTTTAAAATATATTCTAAG 22099

MW494315.1 -----------------------------------------TTTTAAAATATATTCTAAG 22180

KF686346.1 GATAAATCTGAACCTTTGTGTCTGTTCAAGAAAAATTTTACTTATAATGTTTCTACAGAT 23609

NC_006213.1 GATACAGGTGTTGTTTCCTGTTTATATAAGCGTAATTTCACATATGATGTGAATGCTGAT 24272

NC_002645.1 -----------------------------------------TTGTAAAGGTTTTTCCTCA 20899

JX504050.1 -----------------------------------------TTGTAACGGCTATCAACAT 21338

NC_019843.3 ---------------------------------------------CATCTCGGTATGTTG 22269

NC_004718.3 TATCAACCTATAGATGTAGTTCGT---------------------GATCTACCTTCTGGT 22127

EPI_ISL_6640916_omicron CACACGCCTATTA---TAGTGCGTGAGCCAGA------------AGATCTCCCTCAGGGT 22144

MW494315.1 CACACGCCTATTAATTTAGTGCGT---------------------GATCTCCCTCAGGGT 22219

KF686346.1 TTCTTGTATTTTCATTTTTATCAAGAACGTGGCACTTTTTATGCTTATTATGCTGACTCT 23669

NC_006213.1 TATTTGTATTTTCATTTTTATCAAGAAGGTGGTACTTTTTATGCATATTTTACAGACACT 24332

NC_002645.1 GATGTTTTGTCTGATGTCATACGTTACAACCT------------CAATTTTGAAGAAAAC 20947

JX504050.1 AATTCTGTTGTTGATGTTATGCGTTACAATCTTAACTTCAGTGCTAATTCTTTGGACAAT 21398

**

NC_019843.3 ATTTGTACGGCGGCAATATGTTTCAATTTGCCACCTTGCCTGTTTATGATACTATTAAGT 22329

NC_004718.3 TTTAACACTTTGAAACCTATTTTTAAGTTGCCTCTTGGTATTAACATTACAAATTTTAGA 22187

EPI_ISL_6640916_omicron TTTTCGGCTTTAGAACCATTGGTAGATTTGCCAATAGGTATTAACATCACTAGGTTTCAA 22204

MW494315.1 TTTTCGGCTTTAGAACCATTGGTAGATTTGCCAATAGGTATTAACATCACTAGGTTTCAA 22279

KF686346.1 GGCATGCCTACTACTTTTTTATTTAGTTTGTATCTTGGTACTCTTTTATCTCATTATTAT 23729

NC_006213.1 GGTGTTGTTACTAAGTTTTTGTTTAATGTTTATTTAGGCATGGCGCTTTCACACTATTAT 24392

NC_002645.1 CTTAGACGTGGAACCATTTTGTTTAAAACATCTTATGGTGTTGTTGTGTTTTATTGTAC- 21006

JX504050.1 CTCAAGAGTGGTGTTATAGTTTTTAAAACTTTACAGTACGATGTTTTGTTTTATTGTAG- 21457

* * *

NC_019843.3 ATTATTCTATC------------ATTCCTCACA-GTATTCGTTCTATCCAAAGTGATAGA 22376

NC_004718.3 GCCATTCTTAC------------AGCCTTTTCACCTGCTCAAGACATTTGGGGCACGTCA 22235

EPI_ISL_6640916_omicron ACTTTACTTGCTTTACATAGAAGTTATTTGACTCCTGGTGATTCTTCTTCAGGTTGGACA 22264

MW494315.1 ACTTTACTTGCTTTACATAGAAGTTATTTGACTCCTGGTGATTCTTCTTCAGGTTGGACA 22339

KF686346.1 GTTTTGCCTTT------------------GACTTGTAATGCTATATCTTCTAATACTGAT 23771

NC_006213.1 GTCATGCCTCT------------------GACTTGTAATAG------------------T 24416

NC_002645.1 -----------------------------------------------------------C 21007

JX504050.1 -----------------------------------------------------------T 21458

NC_019843.3 AAAGCTTGGGCTGCCTTCTACGTATATAAACTTCAACCGTTAACTTTCCTGTTGGATTTT 22436

NC_004718.3 ------GCTGCAGCCTATTTTGTTGGCTATTTAAAGCCAACTACATTTATGCTCAAGTAT 22289

EPI_ISL_6640916_omicron GCTGGTGCTGCAGCTTATTATGTGGGTTATCTTCAACCTAGGACTTTTCTATTAAAATAT 22324

MW494315.1 GCTGGTGCTGCAGCTTATTATGTGGGTTATCTTCAACCTAGGACTTTTCTATTAAAATAT 22399

KF686346.1 AATGAGACTTTACAATATTGGGTCACACCTTTGTCTAAACGCCAATATCTTCTTAAATTT 23831

NC_006213.1 AAGCTTACTTTAGAATATTGGGTTACACCTCTCACTTCTAGACAATATTTACTCGCTTTC 24476

NC_002645.1 AACAACACTTTAGTTTCAGGTGATGCTCACATACCATTTGGTACAGTTTTGGGCAATTTT 21067

JX504050.1 AATTCTTCCTCAGGTGTTCTTGACACCACAATACCTTTTGGCCCGTCCTCTCAACCTTAT 21518

* * *

NC_019843.3 TCTGTTGATGGTTATATACGCAGAGCTATAGACTGTGGTTTTAATGATTTGTCACAACTC 22496

NC_004718.3 GATGAAAATGGTACAATCACAGATGCTGTTGATTGTTCTCAAAATCCACTTGCTGAACTC 22349

EPI_ISL_6640916_omicron AATGAAAATGGAACCATTACAGATGCTGTAGACTGTGCACTTGACCCTCTCTCAGAAACA 22384

MW494315.1 AATGAAAATGGAACCATTACAGATGCTGTAGACTGTGCACTTGACCCTCTCTCAGAAACA 22459

KF686346.1 GACAACCGTGGTGTTATTACTAATGCTGTTGATTGTTCTAGTAGTTTCTTTAGTGAGATT 23891

NC_006213.1 AATCAAGATGGTATTATTTTTAATGCTGTTGATTGTATGAGTGATTTTATGAGTGAGATT 24536

NC_002645.1 TATTGCTTTGTAAATACTACTATTGGCAATGAAACTACGTCTGCTTTTGTGGGTGCACTA 21127

JX504050.1 TACTGTTTTATAAACAGCACTATCAACACTACTCATGTTAGCACTTTTGTGGGTATTTTA 21578

* * * *

NC_019843.3 CACTGCTCATATG---AATCCTTCGATGTTGAATCTGGAGTTTATTCAGTTTCGTCTTTC 22553

NC_004718.3 AAATGCTCTGTTA---AGAGCTTTGAGATTGACAAAGGAATTTACCAGACCTCTAATTTC 22406

EPI_ISL_6640916_omicron AAGTGTACGTTGA---AATCCTTCACTGTAGAAAAAGGAATCTATCAAACTTCTAACTTT 22441

MW494315.1 AAGTGTACGTTGA---AATCCTTCACTGTAGAAAAAGGAATCTATCAAACTTCTAACTTT 22516

KF686346.1 CAATGTAAAACTA---AATCTTTATTACCTAATACTGGTGTTTATGACTTATCTGGTTTT 23948

NC_006213.1 AAGTGTAAAACAC---AATCTATAGCACCACCTACTGGTGTTTATGAATTAAACGGTTAC 24593

NC_002645.1 CCTAAGACAGTTCGTGAGTTTGTTATTTCACGCACAGGACATTTTTATATTAATGGCTAT 21187

JX504050.1 CCACCCACTGTGCGTGAAATTGTTGTTGCTAGAACTGGTCAGTTTTATATTAATGGTTTT 21638

* * ** * *

NC_019843.3 GAAGCAAAACCTTCTGGCTCAGTTGTGGAACAGGCTGAAGGTGTTGAATGTGATTTTT-- 22611

NC_004718.3 AGGGTTGTTCCCTCAGGAGATGTTGTGAGATTCCCTAATATTACAAACTTGTGTCCTTTT 22466

EPI_ISL_6640916_omicron AGAGTCCAACCAACAGAATCTATTGTTAGATTTCCTAATATTACAAACTTGTGCCCTTTT 22501

MW494315.1 AGAGTCCAACCAACAGAATCTATTGTTAGATTTCCTAATATTACAAACTTGTGCCCTTTT 22576

KF686346.1 ACTGTTAAGCCTGTTGCAACTGTACATCGTCGTATTCCTGATTTACCTGATTGTGACATT 24008

NC_006213.1 ACTGTTCAGCCAATCGCAGATGTTTACCGACGTAAACCTAATCTTCCCAATTGCAATATA 24653

NC_002645.1 CGCTATTTCACTTTAGGTAATGTAGAAGCCGTTAATTTCAATGTCACTACTGCAGAAACC 21247

JX504050.1 AAGTATTTCGATTTGGGTTTCATAGAAGCTGTCAATTTTAATGTCACGACTGCTAGTGCC 21698

* * *

NC_019843.3 ----CACCTCTTCTGTCTGGCACACCTCCTCAGGTTTATAATTTCAAGCGTTTGGTTTTT 22667

NC_004718.3 GGAGAGGTTTTTAATGCTACTAAATTCCCTTCTGTCTATGCATGGGAGAGAAAAAAAATT 22526

EPI_ISL_6640916_omicron GATGAAGTTTTTAACGCCACCAGATTTGCATCTGTTTATGCTTGGAACAGGAAGAGAATC 22561

MW494315.1 GGTGAAGTTTTTAACGCCACCAGATTTGCATCTGTTTATGCTTGGAACAGGAAGAGAATC 22636

KF686346.1 GATAAATGGCTTAACAATTTTAATGTACCCTCACCTCTTAATTGGGAACGTAAAATTTTT 24068

NC_006213.1 GAAGCTTGGCTTAATGATAAGTCGGTGCCCTCTCCATTAAATTGGGAACGTAAGACATTT 24713

NC_002645.1 ACTGATT----------------------------------------------------- 21254

JX504050.1 ACAGATT----------------------------------------------------- 21705

NC_019843.3 ACCAATTGCAATTATAATCTTACCAAATTGCTTTCACTTTTTTCTGTGAATGATTTTACT 22727

NC_004718.3 TCTAATTGTGTTGCTGATTACTCTGTGCTCTACAACTCAACATTTTTTTCAACCTTTAAG 22586

EPI_ISL_6640916_omicron AGCAACTGTGTTGCTGATTATTCTGTCCTATATAATCTCGCACCATTTTTCACTTTTAAG 22621

MW494315.1 AGCAACTGTGTTGCTGATTATTCTGTCCTATATAATTCCGCATCATTTTCCACTTTTAAG 22696

KF686346.1 TCTAATTGCAACTTTAATTTGAGTACTTTGCTTCGTTTAGTTCATACTGATTCTTTTTCT 24128

NC_006213.1 TCAAATTGTAATTTTAATATGAGCAGCCTGATGTCTTTTATTCAGGCAGACTCATTTACT 24773

NC_002645.1 ----------------------------------------TTTGTACTGTTGCGTTAGCT 21274

JX504050.1 ----------------------------------------TTTGGACGGTTGCATTTGCT 21725

**

NC_019843.3 TGTAGTCAAATATCTCCAGCAGCAATTGCTAGCAACTGTTATTCTTCACTGATTTTGGAT 22787

NC_004718.3 TGCTATGGCGTTTCTGC--CACTAAGTTGAATGATCTTTGCTTCTCCAATGTCTATGCAG 22644

EPI_ISL_6640916_omicron TGTTATGGAGTGTCTCC--TACTAAATTAAATGATCTCTGCTTTACTAATGTCTATGCAG 22679

MW494315.1 TGTTATGGAGTGTCTCC--TACTAAATTAAATGATCTCTGCTTTACTAATGTCTATGCAG 22754

KF686346.1 TGTAATAATTTTGATGAATCTAAGATATATGGTAGTTGTTTTAAGAGTATTGTTTTAGAT 24188

NC_006213.1 TGTAATAATATTGATGCTGCTAAGATATATGGTATGTGTTTTTCCAGCATAACTATAGAT 24833

NC_002645.1 TCTTATGCTGACGTTTTGGTTAATGTGTCACAAACCTCTATTGCTAATATAATTTATTGC 21334

JX504050.1 ACTTTTGTTGATGTTTTGGTTAATGTTAGTGCAACTAACATTCAAAACTTACTTTATTGC 21785

* * * * * *

NC_019843.3 TACTTTTCATACCCACTTAGTATGAAATCCGAT--CTCAGTGTTAGTTCTGCTGGTCCAA 22845

NC_004718.3 ATTCTTTTGTAGTCAAGGGAGATGATGTAAGACAAATAGCGCCAGGACAAACTGGTGTTA 22704

EPI_ISL_6640916_omicron ATTCATTTGTAATTAGAGGTGATGAAGTCAGACAAATCGCTCCAGGGCAAACTGGAAATA 22739

MW494315.1 ATTCATTTGTAATTAGAGGTGATGAAGTCAGACAAATCGCTCCAGGGCAAACTGGAAAGA 22814

KF686346.1 AAATTTGCCATACCCAACTCCAGACGATCTGAT--TTGCAGTTGGGCAGTTCTGGTTTTC 24246

NC_006213.1 AAGTTTGCTATACCCAATGGCAGGAAGGTTGAC--CTACAATTGGGTAATTTGGGCTATT 24891

NC_002645.1 AACTCTGTTATTAACAGACTGAGATGTGACCAG--TTGTCCTTTGATGTACCAGATGGTT 21392

JX504050.1 GATTCTCCATTTGAAAAGTTGCAGTGTGAGCAC--TTGCAGTTTGGATTGCAGGATGGTT 21843

* * * *

NC_019843.3 TATCCCAGTTTAATTATAAACAGTCCTTTT------------------------------ 22875

NC_004718.3 TTGCTGATTATAATTATAAATTGCCAGATG------------------------------ 22734

EPI_ISL_6640916_omicron TTGCTGATTATAATTATAAATTACCAGATG------------------------------ 22769

MW494315.1 TTGCTGATTATAATTATAAATTACCAGATG------------------------------ 22844

KF686346.1 TGCAATCTTCTAATTATAAAATTGACACTA------------------------------ 24276

NC_006213.1 TGCAGTCATTTAACTATAGAATTGATACTA------------------------------ 24921

NC_002645.1 TTTATTCTACAAGCCCTATTCAATCCGTTGAGCTACCTGTGTCTATTGTGTCGCTACCTG 21452

JX504050.1 TTTATTCTGCAAATTTTCTTGATGATAATGTTTTGCCTGAGACTTATGTTGCACTCCCCA 21903

* * * *

NC_019843.3 ---------------------------------------------CTAATCCCACATGTT 22890

NC_004718.3 ---------------------------------------------ATTTCATGGGTTGTG 22749

EPI_ISL_6640916_omicron ---------------------------------------------ATTTTACAGGCTGCG 22784

MW494315.1 ---------------------------------------------ATTTTACAGGCTGCG 22859

KF686346.1 ---------------------------------------------CTTCTAGTTCTTGTC 24291

NC_006213.1 ---------------------------------------------CTGCAACAAGTTGTC 24936

NC_002645.1 TTTATCATAAACATACGTTTATTGTGTTGTACGTTGACTTCAAACCTCAGAGTGGCGGTG 21512

JX504050.1 TTTATTATCAACACACGGACAT-------AAATTTTACTGCAA--CTGCATCTTTTGGTG 21954

* *

NC_019843.3 TGATTTTAGCGACTGTTCCTCATAACCTTACTACTATTAC-------------------- 22930

NC_004718.3 TCCTTGCTTGGAATACTAGGAACATTGATGCTACTTCAAC-------------------- 22789

EPI_ISL_6640916_omicron TTATAGCTTGGAATTCTAACAAGCTTGATTCTAAGGTTAG-------------------- 22824

MW494315.1 TTATAGCTTGGAATTCTAACAATCTTGATTCTAAGGTTGG-------------------- 22899

KF686346.1 AATTGTATTATAGTTTGCCTGCAATTAATGTTACTATTAA---------------TAATT 24336

NC_006213.1 AGTTGTATTATAATTTACCTGCTGCTAATGTTTCTGTTAG---------------CAGGT 24981

NC_002645.1 GCAAGTGCTTTAACTGTTATCCTGCTGGTGTTAATATTACACTGGCCAATTTTAATGAAA 21572

JX504050.1 GTTCTTGTTATGTTTGTAAACCACACCAGGTTAATATATCTCT------------TAATG 22002

*

NC_019843.3 ------------------------------------------------------------ 22930

NC_004718.3 ------------------------------------------------------------ 22789

EPI_ISL_6640916_omicron ------------------------------------------------------------ 22824

MW494315.1 ------------------------------------------------------------ 22899

KF686346.1 ATAATCCTTCTTCTTGGAATAGAAGGTATGGTTTTA------------------------ 24372

NC_006213.1 TTAATCCTTCTACTTGGAATAAGAGATTTGGTTTTATAGAAGATTCTGTTT--------- 25032

NC_002645.1 CTAAAGGGCCTTTGTGTGTTGACACATCACACTTCACTACCAAATACGTT---------- 21622

JX504050.1 GTAACACTTCAGTGTGTGTTAGAACATCTCATTTTTCAATTAGGTATATTTATAACCGCG 22062

NC_019843.3 ------------------------------------------------------------ 22930

NC_004718.3 ------------------------------------------------------------ 22789

EPI_ISL_6640916_omicron ------------------------------------------------------------ 22824

MW494315.1 ------------------------------------------------------------ 22899

KF686346.1 ------------ATAATTTTAATTTGAGCTCTCATAGTGTTGTTTACTCACGTTATTGTT 24420

NC_006213.1 TTAAGCCTCGACCTGCAGGTGTTCTTACTAATCATGATGTAGTTTATGCACAACACTGTT 25092

NC_002645.1 -----GCTGTTTATGCCAATGTTGGTAGGTGGAGTGCTAGTATTAACACGGGAAATTGCC 21677

JX504050.1 TTAAGAGTGGTTCACCAGGTGACTCTTCATGGCACATTTATTTAAAGAGTGGCACTTGTC 22122

NC_019843.3 -------------------------------------------------------TAAGC 22935

NC_004718.3 -------------------------------------------------------TGGTA 22794

EPI_ISL_6640916_omicron -------------------------------------------------------TGGTA 22829

MW494315.1 -------------------------------------------------------TGGTA 22904

KF686346.1 TTTCTGTTAATAATACTTTTTGTCCTTGTGC---TAAACCTTCTTTTGCTTCAAGTTGCA 24477

NC_006213.1 TCAAAGCTCCTAAAAATTTCTGTCCGTGTAAATTGAATGGTTCGTGTGTAGGTAGTGGTC 25152

NC_002645.1 CTTTTTCTTTTGGCAAAGTTAATAACTTTGT---TAAATTTGGCAGTGTATGTTTTTCGC 21734

JX504050.1 CATTTTCTTTTTCTAAGTTAAATAATTTTCA---AAAGTTCAAGACTATTTGTTTCTCAA 22179

NC_019843.3 CTCTTAAGTACAGCTATATTAACAAGTGCTCTCGTCTTCTTTCTGATGATCGTACTGAAG 22995

NC_004718.3 ATTATAATTATAAATATAGGTATCTTAGACATGGCAAGCTTAGGCCCTTTGAGAGAGACA 22854

EPI_ISL_6640916_omicron ATTATAATTACCTGTATAGATTGTTTAGGAAGTCTAATCTCAAACCTTTTGAGAGAGATA 22889

MW494315.1 ATTATAATTACCTGTATAGATTGTTTAGGAAGTCTAATCTCAAACCTTTTGAGAGAGATA 22964

KF686346.1 AGAGTCATAAACCACCTTCTGCTTCCTGTCCTATTGGTACTAATTATCGTTCTTGTGAGA 24537

NC_006213.1 CTGGTAAAAATAATGGTATAGGCACTTGTCCTGCAGGTACTAATTATTTAACTTGTGATA 25212

NC_002645.1 TAAAGGATATACCCGGTGGTTGCGCAATGCCTATAGTGGCTAATT--------------- 21779

JX504050.1 CCGTCGAAGTGCCTGGTAGTTGTAATTTTCCGCTTGAAGCCACCT--------------- 22224

* *

NC_019843.3 TACCTCAGTTAGTGAACGCTAATCAATACTCACCCTGTGTATCCATTGT----------- 23044

NC_004718.3 TATCTAATGTGCCTTTCTCCCCTGATGGCAAACCTTGCACCCCACCTGC---TCTTAATT 22911

EPI_ISL_6640916_omicron TTTCAACTGAAATCTATCAGGCCGGTAACAAACCTTGTAATGGTGTTGCAGGTTTTAATT 22949

MW494315.1 TTTCAACTGAAATCTATCAGGCCGGTAGCACACCTTGTAATGGTGTTGAAGGTTTTAATT 23024

KF686346.1 GTACTACTGTACTCGACCACACTGACTGGTGTAGGTGTTCTTGTTTACC----------- 24586

NC_006213.1 ------------------------------------ATTTGTGCACTCC----------- 25225

NC_002645.1 ------------------------------------GGGCTTATAGTAA----------- 21792

JX504050.1 ------------------------------------GGCATTACACTTC----------- 22237

NC_019843.3 ----------------------------CCCATCCACTGTGTGGGAAGACGGTGATTATT 23076

NC_004718.3 GTTATTGGCCATTAAATGATTATGGTTTTTACACCACTACTGGCATTGGCTACCAACCTT 22971

EPI_ISL_6640916_omicron GTTACTTTCCTTTACGATCATATAGTTTCCGACCCACTTATGGTGTTGGTCACCAACCAT 23009

MW494315.1 GTTACTTTCCTTTACAATCATATGGTTTCCAACCCACTAATGGTGTTGGTTACCAACCAT 23084

KF686346.1 ----------------------------TGATCCTATAACTGCTTATGACCCTAGGTCTT 24618

NC_006213.1 ----------------------------TGATCCTATTACATTTACAGGTACTTATAAGT 25257

NC_002645.1 ----------------------------GTACTATACTATAGGCTCATTGTATGTTTCTT 21824

JX504050.1 ----------------------------TTATACTATTGTTGGTGCTTTGTATGTTACTT 22269

* *

NC_019843.3 ATAGGAAACAACTATCTCCACTTGAAGGTGGTGGCTGGCTTGTTGCTAGTGGCTCAACTG 23136

NC_004718.3 ACAGAGTTGTAGTACTTTCTTTTGAA---------------------------------- 22997

EPI_ISL_6640916_omicron ACAGAGTAGTAGTACTTTCTTTTGAA---------------------------------- 23035

MW494315.1 ACAGAGTAGTAGTACTTTCTTTTGAA---------------------------------- 23110

KF686346.1 GTTCTCAAAAAAAGTCTTTGGTTGGTGTTGGTGAACATTGTGCAGGGTTCGGTGTTGATG 24678

NC_006213.1 GCCCCCAAACTAAATCTTTAGTTGGCATAGGTGAGCACTGTTCGGGTCTTGCTGTTAAAA 25317

NC_002645.1 GGAGTGATGGTGATGGAATTACTGGCGTCCCACAACCTGTTGAGGGTGTTAGTTCCTTTA 21884

JX504050.1 GGTCTGAAGGTAATTCTATTACTGGTGTACCTTATCCTGTCTCTGGTATTCGTGAGTTTA 22329

**

NC_019843.3 TTGCCATGACTGAGCAATTACAGATGGGCTTTGGTATTACAGTTCAATATGGTACAGACA 23196

NC_004718.3 ------------------------------------------------------------ 22997

EPI_ISL_6640916_omicron ------------------------------------------------------------ 23035

MW494315.1 ------------------------------------------------------------ 23110

KF686346.1 AAGAAAAGTGTGGTGTATTGGATGGATCATATAATGTTTCTTGTCTTTGTAGTACTGATG 24738

NC_006213.1 GTGATTATTGTGGAGGC------------------AATTCTTGTACTTGCCGACCACAAG 25359

NC_002645.1 TGAATGTTACATTGGACAAATGTACTAAATATAATATTTATGATGTATCTGGTGTGGGTG 21944

JX504050.1 GTAATTTAGTTTTAAATAATTGTACCAAATATAATATTTATGATTATGTTGGTACTGGAA 22389

NC_019843.3 CCAATAGTGTTTGCCCCAAGCTTGAATTTGCTAATGACACAAAAA--------------- 23241

NC_004718.3 --------CTTTTAAATGCACCGGCCACGGTTTGTGGACCAAAAT--------------- 23034

EPI_ISL_6640916_omicron --------CTTCTACATGCACCAGCAACTGTTTGTGGACCTAAAA--------------- 23072

MW494315.1 --------CTTCTACATGCACCAGCAACTGTTTGTGGACCTAAAA--------------- 23147

KF686346.1 CCTTTCTAGGTTGGTCTTATGACACTTGCGTCAGTAACAACCGTTGTAATATTTTTTCTA 24798

NC_006213.1 CATTTTTGGGTTGGTCTGCAGACTCTTGTTTACAAGGAGACAAGTGTAATATTTTTGCTA 25419

NC_002645.1 ---TTATTCGCGTTAGCAATGACACCTTTCTTAATGGAATTACGT---ACACATCAACTT 21998

JX504050.1 ---TTATACGTTCTTCAAACCAGTCACTTGCTGGTGGTATTACAT---ATGTTTCTAACT 22443

NC_019843.3 ---------------------------------------------------------TTG 23244

NC_004718.3 ---------------------------------------------------------TAT 23037

EPI_ISL_6640916_omicron ---------------------------------------------------------AGT 23075

MW494315.1 ---------------------------------------------------------AGT 23150

KF686346.1 ATTTTATTTTAAATGGTATCAATAGTGGTACCACTTGTTCTAATGATTTATTGCAGCCTA 24858

NC_006213.1 ATTTTATTTTGCATGATGTTAATAGTGGTCTTACTTGTTCTACTGATTTACAAAAAGCTA 25479

NC_002645.1 CAGGTAACCTTCTGGGTTTTAAAGATGTTACTAAGGGCACCATCTACTCTATCACTCCTT 22058

JX504050.1 CTGGTAATTTACTTGGTTTTAAAAATGTTTCCACTGGTAACATTTTTATTGTGACACCAT 22503

NC_019843.3 CCTCTCAATTAG------GCAATTGCGTGGAATATTCCCTCTATGGTGTTTCGGGCCGTG 23298

NC_004718.3 CCACTGACCTTATTAAGAACCAGTGTGTCAATTTTAATTTTAATGGACTCACTGGTACTG 23097

EPI_ISL_6640916_omicron CTACTAATTTGGTTAAAAACAAATGTGTCAATTTCAACTTCAATGGTTTAAAAGGCACAG 23135

MW494315.1 CTACTAATTTGGTTAAAAACAAATGTGTCAATTTCAACTTCAATGGTTTAACAGGCACAG 23210

KF686346.1 ATACTGAAGTTTATACTGATGTTTGTGTTGATTACGACCTTTATGGTATTACAGGACAAG 24918

NC_006213.1 ACACAGACATAATTCTTGGTGTTTGTGTTAATTATGACCTCTATGGTATTTTAGGCCAAG 25539

NC_002645.1 GTAACCCACCA------------------------GATCAGCTTGTTGTTTATCAGCAAG 22094

JX504050.1 GTAACCAACCA------------------------GACCAAGTAGCTGTTTATCAACAAA 22539

* *

NC_019843.3 GTGTTTTTCAGAA---------------------------TTGCACAGC------TGTAG 23325

NC_004718.3 GTGTGTTAACTCC---------------------------TTCTTCAAAGAGATTTCAAC 23130

EPI_ISL_6640916_omicron GTGTTCTTACTGA---------------------------GTCTAACAAAAAGTTTCTGC 23168

MW494315.1 GTGTTCTTACTGA---------------------------GTCTAACAAAAAGTTTCTGC 23243

KF686346.1 GTATTTTTAAAGAAGTTTCTGCTGTTTATTATAATAGTTG-------------------- 24958

NC_006213.1 GCATTTTTGTTGAGGTTAATGCGACTTATTATAATAGTTG-------------------- 25579

NC_002645.1 CTGTTGTTGGTGCTATGTTGTCTGAAAATTTTACTAGTTACGGCTTTTCTAATGTTGTAG 22154

JX504050.1 GCATTATTGGTGCCATGACCGCTGTTAATGAGTCTAGATATGGCTTGCAAAACTTACTAC 22599

* *

NC_019843.3 GTGTTCGACAGCAGCGCTTTGTTTATGATGCGTACCAGAATTTAGTTGGCTATTATTCTG 23385

NC_004718.3 CATTTCAACAATTTGGCCGTGATGTTTCTGATTTCACTGATTCCGTTCGAGATCCTAAAA 23190

EPI_ISL_6640916_omicron CTTTCCAACAATTTGGCAGAGACATTGCTGACACTACTGATGCTGTCCGTGATCCACAGA 23228

MW494315.1 CTTTCCAACAATTTGGCAGAGACATTGCTGACACTACTGATGCTGTCCGTGATCCACAGA 23303

KF686346.1 ----GCAAAATCTTTTGTATGATTCTAATGGCAACATTATTGGTTTTAAAGATTTTGTTA 25014

NC_006213.1 ----GCAGAACCTTTTATATGATTCTAATGGTAATCTCTACGGTTTTAGAGACTACATAA 25635

NC_002645.1 AACTGCCGAAATTTTTCTATGCGTCCAATGGCACTTATAATTGCACAGACGCTGTTTTAA 22214

JX504050.1 AGTTACCTAACTTTTATTATGTTAGTAATGGTGGTAACAATTGCACTACGGCCGTTATGA 22659

* * * **

NC_019843.3 ATGATGGCAACTACTACTGTTTGCGTGCTTGTGTTAGTGTTCCTGTTTCTGTCATCTATG 23445

NC_004718.3 CATCTGAAATATTAGACATTTCACCTTGCGCTTTTGGGGGTGTAAGTGTAATTACACCTG 23250

EPI_ISL_6640916_omicron CACTTGAGATTCTTGACATTACACCATGTTCTTTTGGTGGTGTCAGTGTTATAACACCAG 23288

MW494315.1 CACTTGAGATTCTTGACATTACACCATGTTCTTTTGGTGGTGTCAGTGTTATAACACCAG 23363

KF686346.1 CTAATAAAACATATAATATTT---TCCCTTGTTATGCAGGAAGAGTTTCTGCTGCTTTTC 25071

NC_006213.1 CAAACAGAACTTTTATGATTC---GTAGTTGCTATAGCGGTCGTGTTTCTGCGGCCTTTC 25692

NC_002645.1 CTTATTCTAGTTTTGGCGTTT---GTGC-------------------------------- 22239

JX504050.1 CTTATTCTAATTTTGGTATTT---GTGC-------------------------------- 22684

* *

NC_019843.3 ATAAAGAAACTAAAACCCAC---GCTACTCTATTTGGTAGTGTTGCATGTGAACACATTT 23502

NC_004718.3 GAACAAATGCTTCATCTGAAGTTGCTGTTCTATATCAAGATGTTAACTGCACTGATGTTT 23310

EPI_ISL_6640916_omicron GAACAAATACTTCTAACCAGGTTGCTGTTCTTTATCAGGGTGTTAACTGCACAGAAGTCC 23348

MW494315.1 GAACAAATACTTCTAACCAGGTTGCTGTTCTTTATCAGGRTGTTAACTGCACAGAAGTCC 23423

KF686346.1 ATCAAAATGCTTCCTCTTTG---GCTTTACTTTATCGTAATTTAAAATGTAGCTATGTTT 25128

NC_006213.1 ACGCTAACTCTTCCGAACCA---GCATTGCTATTTCGGAATATTAAATGCAACTACGTTT 25749

NC_002645.1 ------------------------------------------------------------ 22239

JX504050.1 ------------------------------------------------------------ 22684

NC_019843.3 CTTCTACCATGTCTCAATACTCCCGTTCTAC-GCGATCAATGCTTAAACGGCGAGATTCT 23561

NC_004718.3 CTACAGCAATTCATGCAGATCAACTCACACCAGCTTGGCGCATATATTCTACTGGAAACA 23370

EPI_ISL_6640916_omicron CTGTTGCTATTCATGCAGATCAACTTACTCCTACTTGGCGTGTTTATTCTACAGGTTCTA 23408

MW494315.1 CTGTTGCTATTCATGCAGATCAACTTACTCCTACTTGGCGTGTTTATTCTACAGGTTCTA 23483

KF686346.1 TGAATAATATTTCTTTAGCTACTCAGCCA------------------------------T 25158

NC_006213.1 TTAATAATAGTCTTACACGACAGCTGCAACC---------------------CATTAACT 25788

NC_002645.1 ----AGATGGTTCTATAATTGCTGTTCAACC---------------------ACGTAATG 22274

JX504050.1 ----TGATGGTTCTTTGATTCCTGTTCGTCC---------------------GCGTAATT 22719

*

NC_019843.3 ACATATGGCCCCCTTCAGACACCTGTTGGTTGTGTCCTAGGACTTGTTAATTCCTCTTTG 23621

NC_004718.3 ATGTAT-------TCCAGACTCAAGCAGGCTGTCTTATAGGAGCTGAGCATGTCGACACT 23423

EPI_ISL_6640916_omicron ATGTTT-------TTCAAACACGTGCAGGCTGTTTAATAGGGGCTGAATATGTCAACAAC 23461

MW494315.1 ATGTTT-------TTCAAACACGTGCAGGCTGTTTAATAGGGGCTGAACATGTCAACAAC 23536

KF686346.1 ATTTTG-------ATAGTTATCTTGGTTGCGTTTTTAATGCTGATAATTTAACTGATTAT 25211

NC_006213.1 ATTTTG-------ATAGTTATCTTGGTTGTGTTGTCAATGCTTATAATAGTACTGCTATT 25841

NC_002645.1 TTTCAT-------ATGATAGTGTTTCAGCTATCGTCACAGCTAAT--------------- 22312

JX504050.1 CTAGTG-------ATAATGGTATTTCAGCCATAATCACTGCTAAT--------------- 22757

* * *

NC_019843.3 TTCGTAGAGGACTGCAAGTTGCCTCTTGGTCAATCTCTCTGTGCTCTTCCTGACACACCT 23681

NC_004718.3 T---CTTATGAGTGCGACATTCCTATTGGAGCTGGCATTTGTGCTAGTTACCATACAGT- 23479

EPI_ISL_6640916_omicron T---CATATGAGTGTGACATACCCATTGGTGCAGGTATATGCGCTAGTTATCAGACTCA- 23517

MW494315.1 T---CATATGAGTGTGACATACCCATTGGTGCAGGTATATGCGCTAGTTATCAGACTCA- 23592

KF686346.1 TCTGTTTCTTCTTGTGCTCTTCGCATGGGTAGTGGTTTTTGTGTTGATTATAACTCACCT 25271

NC_006213.1 TCTGTTCAAACATGTGATCTCACAGTAGGTAGTGGTTACTGTGTGGATTA---------- 25891

NC_002645.1 ------------------------------------------------------------ 22312

JX504050.1 ------------------------------------------------------------ 22757

NC_019843.3 AGTACTCTCACACCTCGCAGTGTGCG---------CTCTGTTCCAGGTGAAATGCGCTTG 23732

NC_004718.3 --TTCTTTAT------------TACG---------TAGTACTAGCCAAAAATCTATTGTG 23516

EPI_ISL_6640916_omicron --GACTAAGTCTCATCGGCGGGCACG---------TAGTGTAGCTAGTCAATCCATCATT 23566

MW494315.1 --GACTAATTCTCCTCGGCGGGCACG---------TAGTGTAGCTAGTCAATCCATCATT 23641

KF686346.1 TCTTTTTCCTCTTCGCGTCGTAAACGTAGAAGTATTTCTGCTTCTTATCGGTTTGTTACT 25331

NC_006213.1 -----CTCTAAAAACAGACGAAGTCGTGGAGCGATTACCACTGGTTATCGGTTTACTAAT 25946

NC_002645.1 ------------------------------------------------------------ 22312

JX504050.1 ------------------------------------------------------------ 22757

NC_019843.3 GCAT---CCATTGCTTTTAATCATCCTATTCAGGTTGATCAACTTAATAGTAGTTATTTT 23789

NC_004718.3 GCTTATACTATGTCTTTAGGTGCTGATAGTTCAATTGCTTACTCTAATAACACC------ 23570

EPI_ISL_6640916_omicron GCCTACACTATGTCACTTGGTGCAGAAAATTCAGTTGCTTACTCTAATAACTCT------ 23620

MW494315.1 GCCTACACTATGTCACTTGGTGCAGAAAATTCAGTTGCTTACTCTAATAACTCT------ 23695

KF686346.1 TTTGAACCCTTTAATGTCAGTTTTGTTAATGACAGTATTGAGTCTGTGGGTGGTCTTTAT 25391

NC_006213.1 TTTGAGCCATTTACTGTTAATTCAGTAAACGATAGTTTAGAACCTGTAGGTGGTTTGTAT 26006

NC_002645.1 ------------------------------------------------------------ 22312

JX504050.1 ------------------------------------------------------------ 22757

NC_019843.3 AAATTAAGTATACCCACTAATTTTTCCTTTGGTGTGACTCAGGAGTACATTCAGACAACC 23849

NC_004718.3 ---ATTGCTATACCTACTAACTTTTCAATTAGCATTACTACAGAAGTAATGCCTGTTTCT 23627

EPI_ISL_6640916_omicron ---ATTGCCATACCCACAAATTTTACTATTAGTGTTACCACAGAAATTCTACCAGTGTCT 23677

MW494315.1 ---ATTGCCATACCCACAAATTTTACTATTAGTGTTACCACAGAAATTCTACCAGTGTCT 23752

KF686346.1 GAGATCAAAATTCCCACTAACTTTACTATAGTTGGTCAAGAGGAATTTATTCAAACTAAT 25451

NC_006213.1 GAAATTCAAATACCTTCAGAGTTTACTATAGGTAATATGGTGGAGTTTATTCAAACAAGC 26066

NC_002645.1 ---TTGTCTATACCTTCCAATTGGACCACTTCGGTCCAGGTTGAGTATTTACAAATTACA 22369

JX504050.1 ---TTATCCATTCCTTCTAACTGGACTACTTCAGTTCAAGTTGAGTACCTCCAAATTACT 22814

* ** ** * * * * ** * *

NC_019843.3 ATTCAGAAAGTTACTGTTGATTGTAAACAGTACGTTTGCAATGGTTTCCAGAAGTGTGAG 23909

NC_004718.3 ATGGCTAAAACCTCCGTAGATTGTAATATGTACATCTGCGGAGATTCTACTGAATGTGCT 23687

EPI_ISL_6640916_omicron ATGACCAAGACATCAGTAGATTGTACAATGTACATTTGTGGTGATTCAACTGAATGCAGC 23737

MW494315.1 ATGACCAAGACATCAGTAGATTGTACAATGTACATTTGTGGTGATTCAACTGAATGCAGC 23812

KF686346.1 TCTCCTAAAGTTACTATTGATTGTTCTTTATTTGTCTGTTCTAATTATGCAGCTTGCCAT 25511

NC_006213.1 TCTCCTAAAGTTACTATTGATTGTGCTGCATTTGTCTGTGGTGATTATGCAGCATGTAAA 26126

NC_002645.1 AGTACACCTATCGTAGTTGATTGCTCCACTTATGTTTGCAATGGTAATGTGCGCTGTGTT 22429

JX504050.1 AGTACTCCAATAGTTGTTGATTGTGCTACTTATGTGTGTAATGGTAACCCTCGCTGTAAG 22874

* ***** * * ** * **

NC_019843.3 CAATTACTGCGCGAGTATGGCCAGTTTTGTTCCAAAATA------------------AAC 23951

NC_004718.3 AATTTGCTTCTCCAATATGGTAGCTTTTGCACACAACTA------------------AAT 23729

EPI_ISL_6640916_omicron AATCTTTTGTTGCAATATGGCAGTTTTTGTACACAATTA------------------AAA 23779

MW494315.1 AATCTTTTGTTGCAATATGGCAGTTTTTGTACACAATTA------------------AAC 23854

KF686346.1 GACTTATTGTCAGAGTATGGCACTTTTTGTGATAATATTAATAGTATTTTAGATGAAGTT 25571

NC_006213.1 TCACAGTTGGTTGAATATGGTAGTTTCTGTGATAACATTAATGCCATACTCACAGAAGTA 26186

NC_002645.1 GAATTGCTTAAGCAGTATACTTCTGCTTGTAAAACTATT------------------GAA 22471

JX504050.1 AATCTACTTAAGCAGTATACTTCTGCTTGTAAAACTATT------------------GAA 22916

* * *** ** *

NC_019843.3 CAGGCTCTCCATGGTGCCAATTTACGCCAGGATGATTCTGTACGTAATTTGTTTGCGAGC 24011

NC_004718.3 CGTGCACTCTCAGGTATTGCTGCTGAACAGGATCGCAACACACGTGAAGTGTTCGCTCAA 23789

EPI_ISL_6640916_omicron CGTGCTTTAACTGGAATAGCTGTTGAACAAGACAAAAACACCCAAGAAGTTTTTGCACAA 23839

MW494315.1 CGTGCTTTAACTGGAATAGCTGTTGAACAAGACAAAAACACCCAAGAAGTTTTTGCACAA 23914

KF686346.1 AATGGTTTACTTGATACTACTCAATTGCATGTAGCTGATACTCTTATGCAAGGTGTCACA 25631

NC_006213.1 AATGAACTACTTGACACTACACAGTTGCAAGTAGCTAATAGTTTAATGAATGGTGTTACT 26246

NC_002645.1 GACGCCTTAAGAAATAGCGCCAGGCTGGAGTCTGCAGATGTTAGTGAGATGCTCACTTTT 22531

JX504050.1 GATGCCTTACGACTTAGTGCTCATTTGGAAACTAATGATGTTAGTAGTATGCTAACTTTC 22976

* * *

NC_019843.3 GTGAAAAGCTCTCAATCATCTCCTATCATACCAGGTTTTGGAGGTGACTTTAATTTGACA 24071

NC_004718.3 GTCAAACAAATGTACAAAACCCCAACTTTGAAATATTTTGGTGGT---TTTAATTTTTCA 23846

EPI_ISL_6640916_omicron GTCAAACAAATTTACAAAACACCACCAATTAAATATTTTGGTGGT---TTTAATTTTTCA 23896

MW494315.1 GTCAAACAAATTTACAAAACACCACCAATTAAAGATTTTGGTGGT---TTTAATTTTTCA 23971

KF686346.1 CTTAGCTCCAATCTTAATACTAATTTGCATTTTGATGTTGATAAT---ATTAATTTTAAA 25688

NC_006213.1 CTTAGCACTAAGCTTAAAGATGGCGTTAATTTCAATGTAGACGAC---ATCAATTTTTCC 26303

NC_002645.1 GACAAGAAAGCGTTTACACTTGCTAATGTTAGTAGTTTTGGTGAC---TACAACCTTAGC 22588

JX504050.1 GATAGCAATGCTTTTAGTTTGGCTAATGTTACTAGTTTTGGAGAT---TATAACCTTTCT 23033

* * * * ** *

NC_019843.3 CTTCTAGAACCTGTTTCTATATCTACTGGCAGT---CGTAGTGCACGTAGTGCTATTGAG 24128

NC_004718.3 CAAATATTACCTGACCCTCTA---------AAG---CCAACTAAGAGGTCTTTTATTGAG 23894

EPI_ISL_6640916_omicron CAAATATTACCAGATCCATCA---------AAA---CCAAGCAAGAGGTCATTTATTGAA 23944

MW494315.1 CAAATATTACCAGATCCATCA---------AAA---CCAAGCAAGAGGTCATTTATTGAA 24019

KF686346.1 TCCCTAGTTGGATGTTTAGGTCCACACTGCGGT---TCTTCTTCTCGTTCTTTTTTTGAA 25745

NC_006213.1 CCTGTATTAGGTTGTCTAGGCAGCGAATGTAGTAAAGCTTCCAGTAGATCTGCTATAGAG 26363

NC_002645.1 AGCGTCATACCTAGCTTGCCCACAAGTGGTAGTAGAGTGGCTGGTCGCAGTGCCATAGAA 22648

JX504050.1 AGTGTTTTACCTCAGAGAAACATTCGTTCAAGCCGTATAGCAGGACGTAGTGCTTTGGAA 23093

* * * **

NC_019843.3 GATTTGCTATTTGACAAAGTCACTATAGCTGATCCTGGTTATATGCAAGGT---TACGAT 24185

NC_004718.3 GACTTGCTCTTTAATAAGGTGACACTCGCTGATGCTGGCTTCATGAAGCAA---TATGGC 23951

EPI_ISL_6640916_omicron GATCTACTTTTCAACAAAGTGACACTTGCAGATGCTGGCTTCATCAAACAA---TATGGT 24001

MW494315.1 GATCTACTTTTCAACAAAGTGACACTTGCAGATGCTGGCTTCATCAAACAA---TATGGT 24076

KF686346.1 GATTTATTGTTTGACAAAGTTAAACTTTCAGATGTTGGTTTTGTTGAAGCT---TATAAC 25802

NC_006213.1 GATTTACTTTTTGATAAAGTAAAGTTATCTGATGTCGGTTTTGTTGAGGCT---TATAAT 26420

NC_002645.1 GACATACTTTTTAGCAAACTTGTTACTTCTGGACTTGGCACTGTGGACGCAGACTACAAA 22708

JX504050.1 GATTTGTTGTTTAGCAAAGTTGTTACATCTGGTTTGGGTACTGTTGATGTTGACTATAAG 23153

** * * ** ** * * * ** * * **

NC_019843.3 GATTGCATGCAGCAAGGTCCAGCATCAGCTCGTGATCTTATTTGTGCTCAATATGTGGCT 24245

NC_004718.3 GAATGC------CTAGGTGATATTAATGCTAGAGATCTCATTTGTGCGCAGAAGTTCAAT 24005

EPI_ISL_6640916_omicron GATTGC------CTTGGTGATATTGCTGCTAGAGACCTCATTTGTGCACAAAAGTTTAAA 24055

MW494315.1 GATTGC------CTTGGTGATATTGCTGCTAGAGACCTCATTTGTGCACAAAAGTTTAAC 24130

KF686346.1 AATTGT------ACTGGTGGTAGTGAAATTAGAGATCTTCTTTGTGTACAATCCTTTAAT 25856

NC_006213.1 AATTGT------ACAGGAGGTGCCGAAATTAGGGACCTCATTTGTGTGCAAAGTTATAAA 26474

NC_002645.1 AAGTGC------ACTAAGGGTCTTTCCATTGCTGACTTGGCTTGTGCTCAATATTATAAT 22762

JX504050.1 TCTTGT------ACTAAAGGTCTTTCTATTGCTGACCTTGCTTGTGCTCAGTACTACAAT 23207

** * ** * ***** **

NC_019843.3 GGTTACAAAGTATTACCTCCTCTTATGGATGTTAATATGGAAGCCGCGTATACTTCATCT 24305

NC_004718.3 GGACTTACAGTGTTGCCACCTCTGCTCACTGATGATATGATTGCTGCCTACACTGCTGCT 24065

EPI_ISL_6640916_omicron GGCCTTACTGTTTTGCCACCTTTGCTCACAGATGAAATGATTGCTCAATACACTTCTGCA 24115

MW494315.1 GGCCTTACTGTTTTGCCACCTTTGCTCACAGATGAAATGATTGCTCAATACACTTCTGCA 24190

KF686346.1 GGTATTAAAGTTTTGCCTCCTATTTTGTCTGAATCTCAAATTTCTGGTTACACCACAGCC 25916

NC_006213.1 GGCATCAAAGTGTTGCCTCCACTGCTCTCAGAAAATCAGATCAGTGGATACACTTTGGCT 26534

NC_002645.1 GGCATTATGGTTTTGCCTGGCGTCGCTGATGCTGAACGAATGGCCATGTATACAGGTTCT 22822

JX504050.1 GGCATAATGGTTTTGCCAGGTGTTGCTGATGCTGAACGTATGGCCATGTACACAGGTTCT 23267

** * ** ** ** * * ** ** *

NC_019843.3 TTGCTTGGCAGCATAGCAGGTGTTGGCTGGACTGCTGGCTTATCCTCCTTTGCTGCTATT 24365

NC_004718.3 CTAGTTAGTGGTACTGCCACTGCTGGATGGACATTTGGTGCTGGCGCTGCTCTTCAAATA 24125

EPI_ISL_6640916_omicron CTGTTAGCGGGTACAATCACTTCTGGTTGGACCTTTGGTGCAGGTGCTGCATTACAAATA 24175

MW494315.1 CTGTTAGCGGGTACAATCACTTCTGGTTGGACCTTTGGTGCAGGTGCTGCATTACAAATA 24250

KF686346.1 GCTACTGTTGCTGCTATGTTTCCACCATGGTCA------------GCAGCAGCTGGCATA 25964

NC_006213.1 GCCACCTCTGCTAGTCTATTTCCTCCTTGGACA------------GCAGCAGCAGGTGTA 26582

NC_002645.1 TTAATTGGTGGAATTGCTTTAGGAGGTCTAACA------------TCAGCCGTTTCAATA 22870

JX504050.1 CTTATAGGTGGCATGGTGCTCGGAGGTCTTACA------------TCAGCAGCCGCCATA 23315

* * *

NC_019843.3 CCATTTGCACAGAGTATCTTTTATAGGTTAAACGGTGTTGGCATTACTCAACAGGTTCTT 24425

NC_004718.3 CCTTTTGCTATGCAAATGGCATATAGGTTCAATGGCATTGGAGTTACCCAAAATGTTCTC 24185

EPI_ISL_6640916_omicron CCATTTGCTATGCAAATGGCTTATAGGTTTAATGGTATTGGAGTTACACAGAATGTTCTC 24235

MW494315.1 CCATTTGCTATGCAAATGGCTTATAGGTTTAATGGTATTGGAGTTACACAGAATGTTCTC 24310

KF686346.1 CCATTTTCTCTTAATGTACAATATAGAATTAATGGTTTGGGTGTTACTATGGATGTTCTT 26024

NC_006213.1 CCATTTTATTTAAATGTTCAGTATCGCATTAATGGGCTTGGTGTCACCATGGATGTGCTA 26642

NC_002645.1 CCATTTTCATTAGCAATTCAGGCACGTTTAAATTATGTTGCATTGCAGACTGATGTTTTA 22930

JX504050.1 CCTTTTTCTTTGGCACTGCAAGCACGACTTAACTATGTTGCTTTACAAACTGATGTGCTT 23375

** *** * * * ** * * * * ** *

NC_019843.3 TCAGAGAACCAAAAGCTTATTGCCAATAAGTTTAATCAGGCTCTGGGAGCTATGCAAACA 24485

NC_004718.3 TATGAGAACCAAAAACAAATCGCCAACCAATTTAACAAGGCGATTAGTCAAATTCAAGAA 24245

EPI_ISL_6640916_omicron TATGAGAACCAAAAATTGATTGCCAACCAATTTAATAGTGCTATTGGCAAAATTCAAGAC 24295

MW494315.1 TATGAGAACCAAAAATTGATTGCCAACCAATTTAATAGTGCTATTGGCAAAATTCAAGAC 24370

KF686346.1 AATAAAAATCAAAAGTTGATAGCTACTGCTTTTAATAATGCTCTTCTTTCTATTCAGAAT 26084

NC_006213.1 AGTCAAAATCAAAAGCTTATTGCTAATGCATTTAACAATGCCCTTTATGCTATTCAGGAA 26702

NC_002645.1 CAAGAAAATCAGAAAATTCTTGCTGCATCTTTTAACAAAGCAATGACCAACATAGTAGAT 22990

JX504050.1 CAAGAAAATCAGAAAATTTTGGCTGCATCATTTAATAAGGCTATTAATAATATTGTTGCT 23435

* ** ** ** * ** ***** ** * **

NC_019843.3 GGCTTCACTA------------------------------------------CAACTAAT 24503

NC_004718.3 TCACTTACAA------------------------------------------CAACATCA 24263

EPI_ISL_6640916_omicron TCACTTTCTT------------------------------------------CCACAGCA 24313

MW494315.1 TCACTTTCTT------------------------------------------CCACAGCA 24388

KF686346.1 GGTTTTAGTG------------------------------------------CTACCAAC 26102

NC_006213.1 GGGTTCGATG------------------------------------------CAACTAAT 26720

NC_002645.1 GCCTTTACTGGTGTTAATGATGCTATTACACAAACTTCACAAGCCCTACAAACAGTTGCT 23050

JX504050.1 TCTTTTAGTAGCGTTAATGATGCTATTACACAAACTGCAGAGGCTATACATACTGTTACT 23495

* *

NC_019843.3 GAAGCTTTTCAGAAGGTTCAGGATGCTGTGAACAACAATGCACAGGCTCTATCCAAATTA 24563

NC_004718.3 ACTGCATTGGGCAAGCTGCAAGACGTTGTTAACCAGAATGCTCAAGCATTAAACACACTT 24323

EPI_ISL_6640916_omicron AGTGCACTTGGAAAACTTCAAGATGTGGTCAACCATAATGCACAAGCTTTAAACACGCTT 24373

MW494315.1 AGTGCACTTGGAAAACTTCAAGATGTGGTCAACCAAAATGCACAAGCTTTAAACACGCTT 24448

KF686346.1 TCTGCACTTGCTAAAATACAAAGTGTTGTTAATTCTAATGCTCAAGCACTTAATAGTTTG 26162

NC_006213.1 TCTGCTTTAGTTAAAATTCAAGCTGTTGTTAATGCAAATGCTGAAGCTCTTAATAACTTA 26780

NC_002645.1 ACTGCACTTAACAAGATCCAGGATGTTGTTAATCAACAAGGCAACTCATTGAACCATTTA 23110

JX504050.1 ATTGCACTTAATAAGATTCAGGATGTTGTTAATCAACAGGGTAGTGCTCTTAACCATCTC 23555

** * ** * ** * ** ** * * * * *

NC_019843.3 GCTAGCGAGCTATCTAATACTTTTGGTGCTATTTCCGCCTCTATTGGAGACATCATACAA 24623

NC_004718.3 GTTAAACAACTTAGCTCTAATTTTGGTGCAATTTCAAGTGTGCTAAATGATATCCTTTCG 24383

EPI_ISL_6640916_omicron GTTAAACAACTTAGCTCCAAATTTGGTGCAATTTCAAGTGTTTTAAATGATATCNTTTCA 24433

MW494315.1 GTTAAACAACTTAGCTCCAATTTTGGTGCAATTTCAAGTGTTTTAAATGATATCCTTTCA 24508

KF686346.1 TTACAGCAATTATTTAATAAATTTGGTGCAATTAGTTCTTCTTTACAAGAAATTTTATCT 26222

NC_006213.1 TTGCAACAACTCTCTAATAGATTTGGTGCTATAAGTGCTTCTTTACAAGAAATTCTATCT 26840

NC_002645.1 ACTTCTCAGTTGAGGCAGAATTTTCAAGCTATCTCTAGCTCTATTCAGGCTATCTATGAC 23170

JX504050.1 ACTTCACAATTGAGACATAATTTTCAGGCCATTTCTAATTCAATTCAGGCTATTTATGAC 23615

* * * *** ** ** * * **

NC_019843.3 CGTCTTGATGTTCTCGAACAGGACGCCCAAATAGACAGACTTATTAATGGCCGTTTGACA 24683

NC_004718.3 CGACTTGATAAAGTCGAGGCGGAGGTACAAATTGACAGGTTAATTACAGGCAGACTTCAA 24443

EPI_ISL_6640916_omicron CGTCTTGACAAAGTTGAGGCTGAAGTGCAAATTGATAGGTTGATCACAGGCAGACTTCAA 24493

MW494315.1 CGTCTTGACAAAGTTGAGGCTGAAGTGCAAATTGATAGGTTGATCACAGGCAGACTTCAA 24568

KF686346.1 CGTCTCGATGCTTTAGAGGCTCAGGTTCAGATTGATAGGCTTATTAATGGTCGTTTAACT 26282

NC_006213.1 AGACTTGATGCTCTTGAAGCGGAAGCTCAGATAGATAGACTTATTAATGGTCGTCTTACC 26900

NC_002645.1 AGACTTGACACTATTCAGGCTGATCAACAAGTAGATAGGCTGATTACTGGTAGATTGGCT 23230

JX504050.1 CGGCTTGATTCAATTCAAGCCGATCAACAAGTTGACAGATTAATTACTGGACGGCTTGCA 23675

* ** ** * * * ** * ** ** * ** * ** * *

NC_019843.3 ACACTAAATGCTTTTGTTGCACAGCAGCTTGTTCGTTCCGAATCAGCTGCTCTTTCCGCT 24743

NC_004718.3 AGCCTTCAAACCTATGTAACACAACAACTAATCAGGGCTGCTGAAATCAGGGCTTCTGCT 24503

EPI_ISL_6640916_omicron AGTTTGCAGACATATGTGACTCAACAATTAATTAGAGCTGCAGAAATCAGAGCTTCTGCT 24553

MW494315.1 AGTTTGCAGACATATGTGACTCAACAATTAATTAGAGCTGCAGAAATCAGAGCTTCTGCT 24628

KF686346.1 GCTTTAAATGCTTATGTCTCTCAACAACTTAGTGATATTTCTCTTGTAAAATTTGGTGCT 26342

NC_006213.1 GCTCTTAATGCTTATGTTTCTCAACAGCTTAGTGATTCTACACTGGTAAAATTTAGTGCA 26960

NC_002645.1 GCTTTGAATGTATTCGTTTCTCATACATTGACTAAGTACACTGAAGTTCGTGCTTCCAGA 23290

JX504050.1 GCTTTGAATGCATTTGTTTCCCAAGTTTTGAATAAATATACTGAAGTTCGTGGTTCAAGA 23735

* * * ** * ** * *

NC_019843.3 CAATTGGCTAAAGATAAAGTCAATGAGTGTGTCAAGGCACAATCCAAGCGTTCTGGATTT 24803

NC_004718.3 AATCTTGCTGCTACTAAAATGTCTGAGTGTGTTCTTGGACAATCAAAAAGAGTTGACTTT 24563

EPI_ISL_6640916_omicron AATCTTGCTGCTACTAAAATGTCAGAGTGTGTACTTGGACAATCAAAAAGAGTTGATTTT 24613

MW494315.1 AATCTTGCTGCTACTAAAATGTCAGAGTGTGTACTTGGACAATCAAAAAGAGTTGATTTT 24688

KF686346.1 GCTTTAGCTATGGAGAAGGTTAATGAGTGTGTTAAAAGTCAATCTCCTCGTATTAATTTT 26402

NC_006213.1 GCACAAGCTATGGAGAAGGTTAATGAATGTGTCAAAAGCCAATCATCTAGGATAAATTTC 27020

NC_002645.1 CAGCTTGCACAACAAAAAGTGAATGAGTGTGTCAAATCCCAGTCTAAGCGTTATGGCTTC 23350

JX504050.1 CGCTTAGCACAGCAGAAGATTAATGAATGTGTCAAGTCACAATCTAATAGATATGGTTTT 23795

** ** * ** ***** ** ** * **

NC_019843.3 TGCGGTCAAGGCACACATATAGTGTCCTTTGTTGTAAATGCCCCTAATGGCCTTTACTTC 24863

NC_004718.3 TGTGGAAAGGGCTACCACCTTATGTCCTTCCCACAAGCAGCCCCGCATGGTGTTGTCTTC 24623

EPI_ISL_6640916_omicron TGTGGAAAGGGCTATCATCTTATGTCCTTCCCTCAGTCAGCACCTCATGGTGTAGTCTTC 24673

MW494315.1 TGTGGAAAGGGCTATCATCTTATGTCCTTCCCTCAGTCAGCACCTCATGGTGTAGTCTTC 24748

KF686346.1 TGTGGTAATGGTAATCATATTTTGTCATTAGTTCAAAATGCTCCTTATGGTTTGTTGTTT 26462

NC_006213.1 TGTGGTAATGGTAATCATATTATATCATTAGTGCAGAATGCTCCATATGGTTTGTATTTT 27080

NC_002645.1 TGTGGAAATGGCACTCACATTTTCTCAATTGTTAATGCTGCTCCTGAGGGGCTTGTTTTT 23410

JX504050.1 TGTGGCAATGGCACTCACATCTTTTCAATCGTCAACTCAGCTCCAGATGGTTTGCTTTTT 23855

** ** * ** ** * * ** * ** ** * ** * **

NC_019843.3 ATGCATGTTGGTTATTACCCTAGCAACCACATTGAGGTTGTTTCTGCTTATGGTCTTTGC 24923

NC_004718.3 CTACATGTCACGTATGTGCCATCCCAGGAGAGGAACTTCACCACAGCGCCAGCAATTTGT 24683

EPI_ISL_6640916_omicron TTGCATGTGACTTATGTCCCTGCACAAGAAAAGAACTTCACAACTGCTCCTGCCATTTGT 24733

MW494315.1 TTGCATGTGACTTATGTCCCTGCACAAGAAAAGAACTTCACAACTGCTCCTGCCATTTGT 24808

KF686346.1 ATGCATTTTAGTTATAAACCTATTTCTTTTAAAACTGTTTTAGTAAGTCCTGGTTTGTGT 26522

NC_006213.1 ATCCACTTTAGTTATGTCCCTACTAAGTATGTCACAGCGAGGGTTAGTCCTGGTCTGTGC 27140

NC_002645.1 CTCCACACTGTCTTGTTGCCGACACAATATAAGGATGTTGAAGCGTGGTCTGGGTTGTGC 23470

JX504050.1 CTTCATACTGTTTTGCTGCCAACTGATTACAAGAATGTAAAGGCGTGGTCTGGTATCTGT 23915

* ** * ** * * **

NC_019843.3 GATGCAGCTAACCCTACTAATTGTATAGCCCCTGTTAATGGCTACTTTATTAAAACTAAT 24983

NC_004718.3 CATGAAGGCAAAGCATA---------CTTCCCTCGTGAAGGTGTTTTTGTGTTTAATGGC 24734

EPI_ISL_6640916_omicron CATGATGGAAAAGCACA---------CTTTCCTCGTGAAGGTGTCTTTGTTTCAAATGGC 24784

MW494315.1 CATGATGGAAAAGCACA---------CTTTCCTCGTGAAGGTGTCTTTGTTTCAAATGGC 24859

KF686346.1 ATATCAGGTGA------TGTAGGTATTGCACCTAAACAAGGGTATTTTATTAAACACAAT 26576

NC_006213.1 ATTGCTGGTGA------TAGAGGTATAGCTCCTAAGAGTGGTTATTTTGTTAATGTAAAT 27194

NC_002645.1 GTTGATGGTAC------AAACGGTTATGTGTTGCGACAACCTAATCTTGCTCTTTACAAA 23524

JX504050.1 GTTGATGGCAT------TTATGGCTATGTTCTGCGTCAACCTAACTTGGTTCTTTATTCT 23969

* *

NC_019843.3 AACACTAGGATTGTTGATGAGTGGTCATATACTGGCTCGTCCTTCTATGCACCTGAGC-- 25041

NC_004718.3 ACTTCTTGGTTTATTACACAGAGGAACTTCTTTTCTCCACAAATAATTACTACAGACA-- 24792

EPI_ISL_6640916_omicron ACACACTGGTTTGTAACACAAAGGAATTTTTATGAACCACAAATCATTACTACAGACA-- 24842

MW494315.1 ACACACTGGTTTGTAACACAAAGGAATTTTTATGAACCACAAATCATTACTACAGACA-- 24917

KF686346.1 GATCATTGGATGTTCACTGGTAGTTCTTACTATTATCCTGAACCAATTTCAGATAAAA-- 26634

NC_006213.1 AATACTTGGATGTACACTGGTAGTGGTTACTACTACCCTGAACCTATAACTGAAAATA-- 27252

NC_002645.1 GAAGGCAATTATTATAGAATCACATCTCGCATAATGTTTGAACCACGTATTCCTACCATG 23584

JX504050.1 GATAATGGTGTCTTTCGTGTAACTTCCAGGATCATGTTTCAACCTCGCTTACCTGTTTTG 24029

NC_019843.3 ----CCATTACCTCCCTTAATACTAAGTATGTTGCACCACAGGTGACATACCAAAACATT 25097

NC_004718.3 ----ATACATTTGTCTCAGGAAATTGTGATGTCGTTATTGGCATCATTAACAACACAGTT 24848

EPI_ISL_6640916_omicron ----ACACATTTGTGTCTGGTAACTGTGATGTTGTAATAGGAATTGTCAACAACACAGTT 24898

MW494315.1 ----ACACATTTGTGTCTGGTAACTGTGATGTTGTAATAGGAATTGTCAACAACACAGTT 24973

KF686346.1 ----ATGTTGTTTTTATGAATACTTGTTCTGTTAATTTTACTAAAGCGCCTCTTGTTTAT 26690

NC_006213.1 ----ATGTTGTTGTTATGAGTACCTGCGCTGTTAATTATACTAAAGCGCCGTATGTAATG 27308

NC_002645.1 GCAGATTTTGTTCAAATTGAAAATTGCAATGTCACATTTGTTAACATTTCTCGCTCTGAG 23644

JX504050.1 TCTGATTTTGTGCAAATATATAATTGTAATGTTACTTTTGTTAACATATCTCGTGTTGAG 24089

* ***

NC_019843.3 TCTACTAACCTCCCTCCTCCTCTTCTCGGCAATTCCACCGGGATTGACTTCCAAGATGAG 25157

NC_004718.3 TATGATCCTCTGCAACCTGAGCTTGACTC------------------ATTCAAAGAAGAG 24890

EPI_ISL_6640916_omicron TATGATCCTTTGCAACCTGAATTAGATTC------------------ATTCAAGGAGGAG 24940

MW494315.1 TATGATCCTTTGCAACCTGAATTAGACTC------------------ATTCAAGGAGGAG 25015

KF686346.1 TTGAATCATTCTGTACCAAAATTGTCTGA------------------TTTTGAATCTGAG 26732

NC_006213.1 CTGAACACTTCAATACCCAACCTTCCTGA------------------TTTTAAGGAAGAG 27350

NC_002645.1 TTGCAAACCATTGTGCCAGAGTATATTGA------------------TGTTAATAAGACG 23686

JX504050.1 TTACATACTGTCATACCTGACTACGTTGA------------------TGTTAATAAAACA 24131

** * *

NC_019843.3 TTGGATGAGTTTTTCAAAAAT---GTTAGCACCAGTATACCTAATTTTGGTT---CCCTA 25211

NC_004718.3 CTGGACAAGTACTTCAAAAAT---CATACATCACCAGATGTTGATCTTGGCG---ACATT 24944

EPI_ISL_6640916_omicron TTAGATAAATATTTTAAGAAT---CATACATCACCAGATGTTGATTTAGGTG---ACATC 24994

MW494315.1 TTAGATAAATATTTTAAGAAT---CATACATCACCAGATGTTGATTTAGGTG---ACATC 25069

KF686346.1 TTATCTCATTGGTTTAAAAAT---CAAACATCCATTGCTCCTAATTTGACTTTAAATCTT 26789

NC_006213.1 TTGGATCAATGGTTTAAAAAT---CAAACATCAGTGGCACCAGATTTGTC------ACTT 27401

NC_002645.1 CTGCAAGAATTAAGTTACAAATTGCCAAATTACACTGTTCCAGACCTAGT------TGTC 23740

JX504050.1 TTACAAGAGTTTGCACAAAACTTACCAAAGTATGTTAAGCCTAATTTTGA------CTTG 24185

* * * * ** * * * *

NC_019843.3 ACACAGATTAATACTACATTACTCGATCTTACCTACGAGATGTT---------------- 25255

NC_004718.3 TCAGGCATTAACGCTTCTGTCGTCAACATTCAAAAAGAAATTGA---------------- 24988

EPI_ISL_6640916_omicron TCTGGCATTAATGCTTCAGTTGTAAACATTCAAAAAGAAATTGA---------------- 25038

MW494315.1 TCTGGCATTAATGCTTCAGTTGTAAACATTCAAAAAGAAATTGA---------------- 25113

KF686346.1 CATACTATTAATGCTACTTTTTTAGATTTGTATTATGAGATGAA---------------- 26833

NC_006213.1 GATTATATAAATGTTACATTCTTGGACCTACAAGTTGAAATGAA---------------- 27445

NC_002645.1 GAACAGTACAACCAGACTATTTTGAATTTGACCAGTGAAATTAGCACCCTTGAAAATAAA 23800

JX504050.1 ACTCCTTTTAATTTAACATATCTTAATTTGAGTTCTGAGTTGAAGCAACTCGAAGCTAAA 24245

** * * * * ** *

NC_019843.3 --------------------------GTCTCTTCAACAAGTTGTTAAAGCCCTTAATGAG 25289

NC_004718.3 --------------------------CCGCCTCAATGAGGTCGCTAAAAATTTAAATGAA 25022

EPI_ISL_6640916_omicron --------------------------CCGCCTCAATGAGGTTGCCAAGAATTTAAATGAA 25072

MW494315.1 --------------------------CCGCCTCAATGAGGTTGCCAAGAATTTAAATGAA 25147

KF686346.1 --------------------------TCTTATTCAAGAGTCTATTAAGTCTTTGAATAAT 26867

NC_006213.1 --------------------------TAGGTTACAGGAGGCAATAAAAGTCTTAAATCAG 27479

NC_002645.1 TCTGCGGAGCTTAATTACACTGTTCAAAAATTGCAAACTCTGATTGACAACATAAATAGC 23860

JX504050.1 ACTGCTAGTCTTTTTCAAACTACTGTTGAATTACAAGGTCTTATTGATCAGATTAACAGT 24305

* * * * **

NC_019843.3 TCTTACATAGACCTTAAAGAGCTTGGCAATTATACTTATTACAACAAATGGCCGTGGTAC 25349

NC_004718.3 TCACTCATTGACCTTCAAGAATTGGGAAAATATGAGCAATATATTAAATGGCCTTGGTAT 25082

EPI_ISL_6640916_omicron TCTCTCATCGATCTCCAAGAACTTGGAAAGTATGAGCAGTATATAAAATGGCCATGGTAC 25132

MW494315.1 TCTCTCATCGATCTCCAAGAACTTGGAAAGTATGAGCAGTATATAAAATGGCCATGGTAC 25207

KF686346.1 AGTTATATCAATCTTAAAGATATAGGTACATATGAAATGTATGTAAAATGGCCTTGGTAT 26927

NC_006213.1 AGCTACATCAATCTCAAGGACATTGGTACATATGAATATTATGTAAAATGGCCTTGGTAT 27539

NC_002645.1 ACATTAGTCGACTTAAAGTGGCTCAACCGGGTTGAGACTTACATCAAGTGGCCGTGGTGG 23920

JX504050.1 ACATATGTTGATTTGAAGTTGCTTAATAGGTTTGAAAATTATATCAAATGGCCTTGGTGG 24365

* * * * * * ** ** ***** ****

NC_019843.3 ATTTGGCTTGGTTTCATTGCTGGGCTTGTTGCCTTAGCTCTATGCGTCTTCTTCATACTG 25409

NC_004718.3 GTTTGGCTCGGCTTCATTGCTGGACTAATTGCCATCG---TCATGGTTACAATCTTGCTT 25139

EPI_ISL_6640916_omicron ATTTGGCTAGGTTTTATAGCTGGCTTGATTGCCATAG---TAATGGTGACAATTATGCTT 25189

MW494315.1 ATTTGGCTAGGTTTTATAGCTGGCTTGATTGCCATAG---TAATGGTGACAATTATGCTT 25264

KF686346.1 GTTTGGCTACTAATTTCTTTTTCATTTATAATATTCCTTGTATTGCTCTTTTTTATATGT 26987

NC_006213.1 GTATGGCTTTTAATCTGCCTTGCTGGTGTAGCTATGCTTGTTTTACTATTCTTCATATGC 27599

NC_002645.1 GTGTGGTTGTGCATTTCAGTCGTGCTCATCTTTGTGGTGAGTATGTTGCTATTATGTTGT 23980

JX504050.1 GTTTGGCTCATTATTTCTGTTGTTTTTGTTGTATTGTTGAGTCTTCTTGTGTTTTGTTGT 24425

* *** * * * * * *

NC_019843.3 TGCTGCACTGGTTGTGGC------ACAAACTGTATGGGAAAACTTAAGTGTAATCGTTGT 25463

NC_004718.3 TGTTGCATGACTAGTTGT------TGCAGTTGCCTCAAGGGTGCATGCTCTTGTGGTTCT 25193

EPI_ISL_6640916_omicron TGCTGTATGACCAGTTGC------TGTAGTTGTCTCAAGGGCTGTTGTTCTTGTGGATCC 25243

MW494315.1 TGCTGTATGACCAGTTGC------TGTAGTTGTCTCAAGGGCTGTTGTTCTTGTGGATCC 25318

KF686346.1 TGTTGTACTGGTTGTGGT------TCTGCATGTTTTAGTAAATGTCATAATTGTTGTGAT 27041

NC_006213.1 TGTTGTACAGGATGTGGG------ACTAGTTGTTTTAAGAAATGTGGTGGTTGTTGTGAT 27653

NC_002645.1 TGTTCTACTGGTTGCTGTGGCTTCTTTAGTTGTTTTGCATCTTCTATTAGAGGTTGTTGT 24040

JX504050.1 CTTTCTACAGGTTGTTGTGGTTGTTGCAATTGTTTAACTTCATCAATGCGAGGCTGTTGT 24485

* * * * ** * *

NC_019843.3 TG---------TGATAGATACGAGGAATACGACCTCGAGCCGCATAAGGTTCATGTTCAC 25514

NC_004718.3 TG---------CTGCAAGTTTGATGAGGATGACTCTGAGCCAG----------------- 25227

EPI_ISL_6640916_omicron TG---------CTGCAAATTTGATGAAGACGACTCTGAGCCAG----------------- 25277

MW494315.1 TG---------CTGCAAATTTGATGAAGACGACTCTGAGCCAG----------------- 25352

KF686346.1 GA------GTATGGTGGTCATCATGATTTTGTTATCAAAACAT----------------- 27078

NC_006213.1 GA------TTATACTGGATACCAGGAGTTAGTAATCAAAACTT----------------- 27690

NC_002645.1 GA------ATCAACTAAACTTCCTTATTACGACGTTGAAAAGA----------------- 24077

JX504050.1 GATTGTGGTTCAACTAAACTTCCTTATTACGAATTTGAAAAGG----------------- 24528

* * *

NC_019843.3 TAATTAACGAACTATTAATGAGAGTTCAAAGACCACCCACTCTCTTGTTAGTGTTTTCAC 25574

NC_004718.3 ------------------------------------------------------------ 25227

EPI_ISL_6640916_omicron ------------------------------------------------------------ 25277

MW494315.1 ------------------------------------------------------------ 25352

KF686346.1 ------------------------CTCATGATGATTAGAATCTCTTGTC----------- 27103

NC_006213.1 ------------------------CACATGACGACTAAGTTCGTCTTTG----------- 27715

NC_002645.1 ------------------------TCCACATACAGTAATGGCTCTAGGT---TTGTTCAC 24110

JX504050.1 ------------------------TCCACGTTCAATAATGCCTTTTGGTGGCCTATTTCA 24564

NC_019843.3 TCTCTCTTTTGGTCACTGCATCCTCAAAACCTCTCTATGTACCTGAGCATTGTCAGAATT 25634

NC_004718.3 ------------------------------------------------------------ 25227

EPI_ISL_6640916_omicron ------------------------------------------------------------ 25277

MW494315.1 ------------------------------------------------------------ 25352

KF686346.1 --A----------------------------------------------------GATCT 27109

NC_006213.1 --ATTCATTGC---ACTGATCTCTTGTTAGATCTTTTTGCAATCTAGCATTTGTTAAAGT 27770

NC_002645.1 ATTGCAACTTG---TGTCTGCTGTTAATCAATCGCTTAGCAATGCGAAAGT-----TAGT 24162

JX504050.1 ACTTACTCTTG---AAAGTACTATTAATAAGAGTGTGGCTAATCTCAAATT-----ACCA 24616

NC_019843.3 ATTCTGGTTGCATGCTTAGGGCTTGTATTAAAACTGCCCAAGCTGATACAGCTGGTCTTT 25694

NC_004718.3 ------------------------------------------------------------ 25227

EPI_ISL_6640916_omicron ------------------------------------------------------------ 25277

MW494315.1 ------------------------------------------------------------ 25352

KF686346.1 CATTAAATCTAAACTTTATTTATGGACGTTTGGAGACCTAGCTACACACATTCTCTTGTT 27169

NC_006213.1 TCTTAAGGCCACGCCCTATTAATGGACATTTGGAGACCTGAGAAGAAATATCTCCGTTAT 27830

NC_002645.1 GCTGAAGTTTCACGACAGGTTATCCAAGACGTGAAAGATGGCACTGTTACCTTCAACTTG 24222

JX504050.1 CCTCATGATGTTACTGTCTTGCGTGACAATCTTAAACCTGTTACTACACTTAGTACTATT 24676

NC_019843.3 ATACAAATTTTCGAATTGACGTCCCATCTGCAGAATCAACTGGTACTCAATCAGTTTCTG 25754

NC_004718.3 -----------------------------------------------------------T 25228

EPI_ISL_6640916_omicron -----------------------------------------------------------T 25278

MW494315.1 -----------------------------------------------------------T 25353

KF686346.1 ATTAGAGAATTTGGTGTTACAAACCTTGAAGATTTGTGTCTAAAGTATAATTACTGTCAA 27229

NC_006213.1 ATTAACGGTTTTAATGTCTCAGAATTAGAAGATGCTTGTTTTAAATTTAACTATCAATTT 27890

NC_002645.1 CTAGCGTATACACTAATGAGCCTCTTTGTTGTGTATTTTGCTTTATTTAAAGCAAGATCA 24282

JX504050.1 ACTGCTTATTTGTTAGTTAGTTTGTTTGTCACTTACTTTGCTTTATTCAAACCTCTTACT 24736

NC_019843.3 TCGATCTTGAGTCAACTTCAACTCATGATGGTCCTACCGAA-CATGTTACTAGTGTGAAT 25813

NC_004718.3 TCTCAAGGGTGTCAAATTACATTACACATAAACGAACTTAT-GGATTTGTTTATGAGATT 25287

EPI_ISL_6640916_omicron GCTCAAAGGAGTCAAATTACATTACACATAAACGAACTTAT-GGATTTGTTTATGAGAAT 25337

MW494315.1 GCTCAAAGGAGTCAAATTACATTACACATAAACGAACTTAT-GGATTTGTTTATGAGAAT 25412

KF686346.1 CCTATTGTTGGTTACTGTATTGTA------------------------------------ 27253

NC_006213.1 CCTAAAGTAGGATATTGTAGAGTT------------------------------------ 27914

NC_002645.1 CACCGTGGCAGAGCTGCTCTTATAGTGTTTAAAATTCTAATCCTTTTCGTTTATGTGCCA 24342

JX504050.1 GCTAGAGGTCGTGTTGCTTGTTTTGTTTTAAAACTATTGACACTATTTGTCTATGTGCCT 24796

* * *

NC_019843.3 CTTTTTGACGTTGGTTACTCAGTTAATTAACGAACTCTATGGATTACGTGTCTCTGCTTA 25873

NC_004718.3 TTT--------------------------------------------------------- 25290

EPI_ISL_6640916_omicron CTT--------------------------------------------------------- 25340

MW494315.1 CTT--------------------------------------------------------- 25415

KF686346.1 ------------------------------------------------------------ 27253

NC_006213.1 ------------------------------------------------------------ 27914

NC_002645.1 TTG--------------------------------------------------------- 24345

JX504050.1 TTA--------------------------------------------------------- 24799

NC_019843.3 ATCAAATTTGGCAGAAGTACCTTAACTCACCGTATACTACTTGTTTGTACATCCCTAAAC 25933

NC_004718.3 ------------------------------------------------------------ 25290

EPI_ISL_6640916_omicron ------------------------------------------------------------ 25340

MW494315.1 ------------------------------------------------------------ 25415

KF686346.1 ------------------------------------------------------------ 27253

NC_006213.1 ------------------------------------------------------------ 27914

NC_002645.1 ------------------------------------------------------------ 24345

JX504050.1 ------------------------------------------------------------ 24799

NC_019843.3 CCACAGCTAAGTATACACCTTTAGTTGGCACTTCATTGCACCCTGTGCTGTGGAACTGTC 25993

NC_004718.3 ------------------------------------------------------------ 25290

EPI_ISL_6640916_omicron ------------------------------------------------------------ 25340

MW494315.1 ------------------------------------------------------------ 25415

KF686346.1 ------------------------------------------------------------ 27253

NC_006213.1 ------------------------------------------------------------ 27914

NC_002645.1 ------------------------------------------------------------ 24345

JX504050.1 ------------------------------------------------------------ 24799

NC_019843.3 AGCTATCCTTTGCTGGTTATACTGAATCTGCTGTTAATTCTACAAAAGCTTTGGCCAAAC 26053

NC_004718.3 ------------------------------------------------------------ 25290

EPI_ISL_6640916_omicron ------------------------------------------------------------ 25340

MW494315.1 ------------------------------------------------------------ 25415

KF686346.1 ------------------------------------------------------------ 27253

NC_006213.1 ------------------------------------------------------------ 27914

NC_002645.1 ------------------------------------------------------------ 24345

JX504050.1 ------------------------------------------------------------ 24799

NC_019843.3 AGGACGCAGCTCAGCGAATCGCTTGGTTGCTACATAAGGATGGAGGAATCCCTGATGGAT 26113

NC_004718.3 ------------------------------------------------------------ 25290

EPI_ISL_6640916_omicron ------------------------------------------------------------ 25340

MW494315.1 ------------------------------------------------------------ 25415

KF686346.1 ------------------------------------------------------------ 27253

NC_006213.1 ------------------------------------------------------------ 27914

NC_002645.1 ------------------------------------------------------------ 24345

JX504050.1 ------------------------------------------------------------ 24799

NC_019843.3 GTTCCCTCTACCTCCGGCACTCAAGTTTATTCGCGCAAAGCGAGGAAGAGGAGCCATTCT 26173

NC_004718.3 ------------------------------------------------------------ 25290

EPI_ISL_6640916_omicron ------------------------------------------------------------ 25340

MW494315.1 ------------------------------------------------------------ 25415

KF686346.1 ------------------------------------------------------------ 27253

NC_006213.1 ------------------------------------------------------------ 27914

NC_002645.1 ------------------------------------------------------------ 24345

JX504050.1 ------------------------------------------------------------ 24799

NC_019843.3 CCAACTAAGAAACTGCGCTACGTTAAGCGTAGATTTTCTCTTCTGCGCCATGAAGACCTT 26233

NC_004718.3 ------------------------------------------------------------ 25290

EPI_ISL_6640916_omicron ------------------------------------------------------------ 25340

MW494315.1 ------------------------------------------------------------ 25415

KF686346.1 ------------------------------------------------------------ 27253

NC_006213.1 ------------------------------------------------------------ 27914

NC_002645.1 ------------------------------------------------------------ 24345

JX504050.1 ------------------------------------------------------------ 24799

NC_019843.3 AGTGTTATTGTCCAACCAACACACTATGTCAGGGTTACATTTTCAGACCCCAACATGTGG 26293

NC_004718.3 ------------------------------------------------------------ 25290

EPI_ISL_6640916_omicron ------------------------------------------------------------ 25340

MW494315.1 ------------------------------------------------------------ 25415

KF686346.1 ------------------------------------------------------------ 27253

NC_006213.1 ------------------------------------------------------------ 27914

NC_002645.1 ------------------------------------------------------------ 24345

JX504050.1 ------------------------------------------------------------ 24799

NC_019843.3 TATCTACGTTCGGGTCATCATTTACACTCAGTTCACAATTGGCTTAAACCTTATGGCGGC 26353

NC_004718.3 ------------------------------------------------------------ 25290

EPI_ISL_6640916_omicron ------------------------------------------------------------ 25340

MW494315.1 ------------------------------------------------------------ 25415

KF686346.1 ------------------------------------------------------------ 27253

NC_006213.1 ------------------------------------------------------------ 27914

NC_002645.1 ------------------------------------------------------------ 24345

JX504050.1 ------------------------------------------------------------ 24799

NC_019843.3 CAACCTGTTTCTGAGTACCATATTACTCTAGCTTTGCTAAATCTCACTGATGAAGATTTA 26413

NC_004718.3 ------------------------------------------------------------ 25290

EPI_ISL_6640916_omicron ------------------------------------------------------------ 25340

MW494315.1 ------------------------------------------------------------ 25415

KF686346.1 ------------------------------------------------------------ 27253

NC_006213.1 ------------------------------------------------------------ 27914

NC_002645.1 ------------------------------------------------------------ 24345

JX504050.1 ------------------------------------------------------------ 24799

NC_019843.3 GCTAGAGATTTTTCACCCATTGCGCTCTTTTTGCGCAATGTCAGATTTGAGCTACATGAG 26473

NC_004718.3 ------------------------------------------------------------ 25290

EPI_ISL_6640916_omicron ------------------------------------------------------------ 25340

MW494315.1 ------------------------------------------------------------ 25415

KF686346.1 ------------------------------------------------------------ 27253

NC_006213.1 ------------------------------------------------------------ 27914

NC_002645.1 ------------------------------------------------------------ 24345

JX504050.1 ------------------------------------------------------------ 24799

NC_019843.3 TTCGCCTTGCTGCGCAAAACTCTTGTTCTTAATGCATCAGAGATCTACTGTGCTAACATA 26533

NC_004718.3 --------------------------------------------------------TACT 25294

EPI_ISL_6640916_omicron --------------------------------------------------------CACA 25344

MW494315.1 --------------------------------------------------------CACA 25419

KF686346.1 ------------------------------------------------------------ 27253

NC_006213.1 ------------------------------------------------------------ 27914

NC_002645.1 ------------------------------------------------------------ 24345

JX504050.1 ------------------------------------------------------------ 24799

NC_019843.3 CATAGATTTAAGCCTGTGTATAGAGTTAACACGGCAATCCCTACTATTAAGGATTGGCTT 26593

NC_004718.3 CTTAGATCAATTACTGCACAGCCAGTAAAAATTGACAATGCTTCTCCTGCAAGTACTGTT 25354

EPI_ISL_6640916_omicron ATTGGAACTGTAACTTTGAAGCAAGGTGAAATCAAGGATGCTACTCCTTCAGATTTTGTT 25404

MW494315.1 ATTGGAACTGTAACTTTGAAGCAAGGTGAAATCAAGGATGCTACTCCTTCAGATTTTGTT 25479

KF686346.1 ------------------------------------------------------------ 27253

NC_006213.1 ------------------------------------------------------------ 27914

NC_002645.1 ------------------------------------------------------------ 24345

JX504050.1 ------------------------------------------------------------ 24799

NC_019843.3 CTCGTT-CAGGGATTTTCCCTTTACCATAGTGGCCTCCCTTTACATATGTCAATCTCTAA 26652

NC_004718.3 CATGCTACAGCAACGATACCGCTACAAGCCTCACTCCCTTTCGGATGGCTTGTTATTGGC 25414

EPI_ISL_6640916_omicron CGCGCTACTGCAACGATACCGATACAAGCCTCACTCCCTTTCGGATGGCTTATTGTTGGC 25464

MW494315.1 CGCGCTACTGCAACGATACCGATACAAGCCTCACTCCCTTTCGGATGGCTTATTGTTGGC 25539

KF686346.1 ------------------------------------------------------------ 27253

NC_006213.1 ------------------------------------------------------------ 27914

NC_002645.1 -------CTGTATTGGTCTCAAGCATATATTTACGCAACTTTGATTGCTGTAATTTTGCT 24398

JX504050.1 -------TTGGTTCTTTTTGGTATGTATCTTGACAGTTTTATAATTTTTTCTACGCTGTT 24852

NC_019843.3 ATTGCATGCACTGGATGATGTTACTCGCAATTACATCATTACAATGC-----CATGCTTT 26707

NC_004718.3 GTTGCATTT-CTTGCTGTTTTTCAGAGCGCTACCAAAATAATTGCGCTCAATAAAAGATG 25473

EPI_ISL_6640916_omicron GTTGCACTT-CTTGCTGTTTTTCAGAGCGCTTCCAAAATCATAACTCTCAAAAAGAGATG 25523

MW494315.1 GTTGCACTT-CTTGCTGTTTTTCATAGCGCTTCCAAAATCATAACCCTCAAAAAGAGATG 25598

KF686346.1 ------------------------------------------------------------ 27253

NC_006213.1 ------------------------------------------------------------ 27914

NC_002645.1 TGGAAGATT--------------------------------------------------- 24407

JX504050.1 GTTTCGATT--------------------------------------------------- 24861

NC_019843.3 AGAACTTACCCTCAACAAATGTTTGTTACTCCTTTGGCCGTAGATGTTGTCTCCATACGG 26767

NC_004718.3 GCAGCTAGCCCTTTATAAGGGCTTCCAGTTCATTTGCAATTTACTGCTG----------- 25522

EPI_ISL_6640916_omicron GCAACTAGCACTCTCCAAGGGTGTTCACTTTGTTTGCAACTTGCTGTTG----------- 25572

MW494315.1 GCAACTAGCACTCTCCAAGGGTGTTCACTTTGTTTGCAACTTGCTGTTG----------- 25647

KF686346.1 ------------------------------------------------------------ 27253

NC_006213.1 ------------------------------------------------------------ 27914

NC_002645.1 -------------------------------------------------TTTCCATACAG 24418

JX504050.1 -------------------------------------------------CATACATGTTG 24872

NC_019843.3 TCTTCCAATCAGGGTAATAAACAAATTGTTCATTCTTATCCCATTTTACATCATCCAGGA 26827

NC_004718.3 ------------------------------------------------------------ 25522

EPI_ISL_6640916_omicron ------------------------------------------------------------ 25572

MW494315.1 ------------------------------------------------------------ 25647

KF686346.1 ------------------------------------------------------------ 27253

NC_006213.1 ------------------------------------------------------------ 27914

NC_002645.1 CTTGGCACTGCTGGCTCTACAAGACATGGGATTTCA------------------------ 24454

JX504050.1 GCTATTATGCCTATCTCTATAAAAATTTTTCATTTG------------------------ 24908

NC_019843.3 TTTTAACGAACTATGGCTTTCTCGGCGTCTTTATTTAAACCCGTCCAGCTAGTCCCAGTT 26887

NC_004718.3 ------------------------------------------------------------ 25522

EPI_ISL_6640916_omicron ------------------------------------------------------------ 25572

MW494315.1 ------------------------------------------------------------ 25647

KF686346.1 ------------------------------------------------------------ 27253

NC_006213.1 ------------------------------------------------------------ 27914

NC_002645.1 ------------------------------------------------------------ 24454

JX504050.1 ------------------------------------------------------------ 24908

NC_019843.3 TCTCCTGCATTTCATCGCATTGAGTCTACTGACTCTATTGTTTTCACATACATTCCTGCT 26947

NC_004718.3 -------------------------CTATTTGTTACCATCTATTCACATCTTTTGCTTGT 25557

EPI_ISL_6640916_omicron -------------------------TTGTTTGTAACAGTTTACTCACACCTTTTGCTCGT 25607

MW494315.1 -------------------------TTGTTTGTAACAGTTTACTCACACCTTTTGCTCGT 25682

KF686346.1 ------------------------------------------------------------ 27253

NC_006213.1 ------------------------------------------------------------ 27914

NC_002645.1 ------------------------------------------------------------ 24454

JX504050.1 ------------------------------------------------------------ 24908

NC_019843.3 AGCGGC------------------------------------------------------ 26953

NC_004718.3 CGCTGCAGGTATGGAGGCGCAATTTTTGTACCTCTATGCCTTGATATATTTTCTACAATG 25617

EPI_ISL_6640916_omicron TGCTGCTGGCCTTGAAGCCCCTTTTCTCTATCTTTATGCTTTAGTCTACTTCTTGCAGAG 25667

MW494315.1 TGCTGCTGGCCTTGAAGCCCCTTTTCTCTATCTTTATGCTTTAGTCTACTTCTTGCAGAG 25742

KF686346.1 ------------------------------------------------------------ 27253

NC_006213.1 ------------------------------------------------------------ 27914

NC_002645.1 ------------------------------------------------------------ 24454

JX504050.1 ------------------------------------------------------------ 24908

NC_019843.3 -----------------------TATGTAGCTGCTTTAGCTGTCAATGTGTGTCTCATTC 26990

NC_004718.3 CATCAACGCATGTAGAATTATTATGAGATGTTGGCTTTGTTGGAAGTGCAAATCCAAGAA 25677

EPI_ISL_6640916_omicron TATAAACTTTGTAAGAATAATAATGAGGCTTTGGCTTTGCTGGAAATGCCGTTCCAAAAA 25727

MW494315.1 TATAAACTTTGTAAGAATAATAATGAGGCTTTGGCTTTGCTGGAAATGCCGTTCCAAAAA 25802

KF686346.1 ------------------------------------------------------------ 27253

NC_006213.1 ------------------------------------------------------------ 27914

NC_002645.1 ------------------------------------------------------------ 24454

JX504050.1 ------------------------------------------------------------ 24908

NC_019843.3 CCCTATTATTACTGCTACGTCAAGATACTTGTCGTCGCAGCATTATCAGAACTATGGTTC 27050

NC_004718.3 CCCATTACTTTATGATGCCAACTACTTTGTTTGCTGGCACACACATAACTATGACTACTG 25737

EPI_ISL_6640916_omicron CCCATTACTTTATGATGCCAACTATTTTCTTTGCTGGCATACTAATTGTTACGACTATTG 25787

MW494315.1 CCCATTACTTTATGATGCCAACTATTTTCTTTGCTGGCATACTAATTGTTACGACTATTG 25862

KF686346.1 ------------------------------------------------------------ 27253

NC_006213.1 ------------------------------------------------------------ 27914

NC_002645.1 ------------------------------------------------------------ 24454

JX504050.1 ------------------------------------------------------------ 24908

NC_019843.3 TCTATTTCCTTGTTCTGTATAACTTTTTATTAGCCATTGTACTAGTCAATGGTGTACATT 27110

NC_004718.3 TATACCATATAACAGTGTCACAGATACAATTGTCGTTACTGAAGGTGACGGCATTTCAAC 25797

EPI_ISL_6640916_omicron TATACCTTACAATAGTGTAACTTCTTCAATTGTCATTACTTCAGGTGATGGCACAACAAG 25847

MW494315.1 TATACCTTACAATAGTGTAACTTCTTCAATTGTCATTACTTCAGGTGATGGCACAACAAG 25922

KF686346.1 ------------------------------------------------------------ 27253

NC_006213.1 ------------------------------------------------------------ 27914

NC_002645.1 ------------------------------------------------------------ 24454

JX504050.1 ------------------------------------------------------------ 24908

NC_019843.3 ATCCAACTGGAAGTTGCCTGATAGCCTTCTTAGTTATCCTCATAATACTTTGGTTTGTAG 27170

NC_004718.3 ACCAAAACTCAAAGAAGACTACCAAATTGGTGGTTATTCTGAGGATAG-------GCACT 25850

EPI_ISL_6640916_omicron TCCTATTTCTGAACATGACTACCAGATTGGTGGTTATACTGAAAAATG-------GGAAT 25900

MW494315.1 TCCTATTTCTGAACATGACTACCAGATTGGTGGTTATACTGAAAAATG-------GGAAT 25975

KF686346.1 -----------------------------------------------------------C 27254

NC_006213.1 -----------------------------------------------------------C 27915

NC_002645.1 ---------------------------TTGTCTTCAATGTAACCACACTTTGCTATG--C 24485

JX504050.1 ---------------------------TTTTGTTCAATGTTACTAAACTATGCTTCGTTT 24941

NC_019843.3 ATAGAATTCGTTTCTGTCTCATGCTGAATTCCTACATTCCACTGTTTGACATGCGTTCCC 27230

NC_004718.3 CAGGTGTTAAAGACTATGTCGTTGTACATGGCTATTTCACCGAAGTTTACTACCAGCTTG 25910

EPI_ISL_6640916_omicron CTGGAGTAAAAGACTGTGTTGTATTACACAGTTACTTCACTTCAGACTATTACCAGCTGT 25960

MW494315.1 CTGGAGTAAAAGACTGTGTTGTATTACACAGTTACTTCACTTCAGACTATTACCAGCTGT 26035

KF686346.1 CTTTAAATGTTTGGTGTCGCAAGTTTGGCAAATTTGCATCTCACTTTACATTACGTAGTC 27314

NC_006213.1 CTAGTCATGCTTGGTGCCGTAATCAAGGTAGATTTTGTGCTACATTCACTCTTTATGGTA 27975

NC_002645.1 AAGGTAAGTGTTGGTTTCTTGAA-AATAAGGCTCTGAAA--CCATTCGT---TTGTTTTT 24539

JX504050.1 CAGGCAAGTGTTGGTATCTTGAACAATCATTTTATGAAAATCGTTTTGC---TGCTATTT 24998

* *

NC_019843.3 ACTTTATTCGTGTTAGTACAGTTTCTTCTCATGGTATGGTCCCTGTAATACACACCAAAC 27290

NC_004718.3 AGTCTACACAAATTACTACAGACACTGGTATTGAAAATGCTACATTCTTCATCTTTAACA 25970

EPI_ISL_6640916_omicron ACTCAACTCAATTGAGTACAGACACTGGTGTTGAACATGTTACCTTCTTCATCTACAATA 26020

MW494315.1 ACTCAACTCAATTGAGTACAGACACTGGTGTTGAACATGTTACCTTCTTCATCTACAATA 26095

KF686346.1 ACGATATTTCCCATAGTAATAATTTTGGTGTTGTAACTAGTTTTACTACTTATGGTAATA 27374

NC_006213.1 AATCCAAACATTATGATAAATATTTTGGAGTAATAAATGGTTTCACAGCATTCGCTAATA 28035

NC_002645.1 ACGGAGGGGATCAATTCCTTTACATAGGCGACAGAATTGTTTCTTATTTCTCAACTAACG 24599

JX504050.1 ATGGTGGTGACCACTATGTCGTTTTAGGTGGTGAAACTATTACTTTTGTTTCTTTTGATG 25058

* *

NC_019843.3 CATTATTTATTAGAAACTTCGATCAGCGTTGCAGCTGTTCTCGTTGTTTTTATTTGCACT 27350

NC_004718.3 AGCT-------------------------------------------------------- 25974

EPI_ISL_6640916_omicron AAAT-------------------------------------------------------- 26024

MW494315.1 AAAT-------------------------------------------------------- 26099

KF686346.1 CTGT-------------------------------------------------------- 27378

NC_006213.1 CTGT-------------------------------------------------------- 28039

NC_002645.1 ACTT-------------------------------------------------------- 24603

JX504050.1 ACCT-------------------------------------------------------- 25062

*

NC_019843.3 CTTCCACTTATATAGAGTGCACTTATATTAGCCGTTTTAGTAAGATTAGCCTAGTTTCTG 27410

NC_004718.3 ------------------------------------------------------------ 25974

EPI_ISL_6640916_omicron ------------------------------------------------------------ 26024

MW494315.1 ------------------------------------------------------------ 26099

KF686346.1 ------------------------------------------------------------ 27378

NC_006213.1 ------------------------------------------------------------ 28039

NC_002645.1 ------------------------------------------------------------ 24603

JX504050.1 ------------------------------------------------------------ 25062

NC_019843.3 TAACTGACTTCTCCTTAAACGGCAATGTTTCCACTGTTTTCGTGCCTGCAACGCGCGATT 27470

NC_004718.3 ----------------------------------TGTTAAAGACCC---ACCGAAT---- 25993

EPI_ISL_6640916_omicron ----------------------------------TGTTGATGAGCCTGAAGAACAT---- 26046

MW494315.1 ----------------------------------TGTTGATGAGCCTGAAGAACAT---- 26121

KF686346.1 ------------------------------------------------------------ 27378

NC_006213.1 ------------------------------------------------------------ 28039

NC_002645.1 ------------------------------------------------------------ 24603

JX504050.1 ------------------------------------------------------------ 25062

NC_019843.3 CAGTTCCTCTTCACATAATCGCCCCGAGCTCGCTTATCGTTTAAGCAGCTCTGCGCTACT 27530

NC_004718.3 --GTGCAAATACACACAATCGACGGCTCTTCAGGAGTTGCTAATCCAGCAATGGATCCAA 26051

EPI_ISL_6640916_omicron --GTCCAAATTCACACAATCGACGGTTCATCCGGAGTTGTTAATCCAGTAATGGAACCAA 26104

MW494315.1 --GTCCAAATTCACACAATCGACGGTTCATCCGGAGTTGTTAATCCAGTAATGGAACCAA 26179

KF686346.1 ----------------TTCTGAGGCTGTGTCTAGATTAGTTGAATCAGCTTCTGAATTTA 27422

NC_006213.1 ----------------AGAGGATGCTGTTAACAAACTGGTTTTCTTAGCTGTTGACTTTA 28083

NC_002645.1 ----------------GTACGTTGCTCTTAGAGGACGTATTGATAAAGACCTCAGCCTTT 24647

JX504050.1 ----------------TTATGTTGCTATTAGAGGTTCTTGTGAAAAGAACCTACAACTTA 25106

* *

NC_019843.3 ATGGGTCCCGTGTAGAGGCTAATCCATTAGTCTCTCTTTGGACATATG-----GAAAACG 27585

NC_004718.3 TTTATGATGAGCCGACGACGACTACTAGCGTGCCTTTGTAAGCACAAGAAAGTGAGTACG 26111

EPI_ISL_6640916_omicron TTTATGATGAACCGACGACGACTACTAGCGTGCCTTTGTAAGCACAAGCTGATGAGTACG 26164

MW494315.1 TTTATGATGAACCGACGACGACTACTAGCGTGCCTTTGTAAGCACAAGCTGATGAGTACG 26239

KF686346.1 TTGTTTGGCGTGCAGAGGCACTTAATAAG------------------------------- 27451

NC_006213.1 TTACCTGGCGCAGACAGGAGTTAAATGTT------------------------------- 28112

NC_002645.1 CTAGAAAG---GTTGAGTTATATAACGGT------------------------------- 24673

JX504050.1 TGCGTAAG---GTTGACTTGTATAATGGT------------------------------- 25132

NC_019843.3 AAC-TATGTTACCCTTTGTCCAAGAACGAATAGGGTTGTTCATAGTAAACTTTTTCAT-- 27642

NC_004718.3 AACTTATGTACTCATTCGTTTCGGAAGAAACAGGTACGTTAATAGTTAATAGCGTACT-- 26169

EPI_ISL_6640916_omicron AACTTATGTACTCATTCGTTTCGGAAGAGATAGGTACGTTAATAGTTAATAGCGTACT-- 26222

MW494315.1 AACTTATGTACTCATTCGTTTCGGAAGAGACAGGTACGTTAATAGTTAATAGCGTACT-- 26297

KF686346.1 ----TATGGTTGATTTATTTTTCAATGATACTGCTTGGTACATAGGACAGATTTTAGT-- 27505

NC_006213.1 ----TATGGCTGATGCTTATCTTGCAGACACTGTGTGGTATGTGGGGCAAATAATTTT-- 28166

NC_002645.1 ----GAATGTGTATACTTGTTTTGTGAACACCCAGCTGTTGGAATAGTCAACACAGATTT 24729

JX504050.1 ----GCTGTCATTTACATTTTTGCCGAAGAGCCTGTTGTTGGTATAGTCTACTCTTCT-- 25186

* ** *

NC_019843.3 ------------------------------------------------------------ 27642

NC_004718.3 ------------------------------------------------------------ 26169

EPI_ISL_6640916_omicron ------------------------------------------------------------ 26222

MW494315.1 ------------------------------------------------------------ 26297

KF686346.1 ------------------------------------------------------------ 27505

NC_006213.1 ------------------------------------------------------------ 28166

NC_002645.1 CAAATTAGAAATCCACTAAGATGTTCCTTAAGCTAGTGGATGATCATGCTTTGGTTGTTA 24789

JX504050.1 -------CAACTATACGAAGATGTTCCTTCGATTAATTGATGACAATGGTATTGTCCTCA 25239

NC_019843.3 -TTTTACCGTAGTATGTGCTATAACACTCTTGGTGTGTATGGCTTTCCTTACGGCTACTA 27701

NC_004718.3 -TCTTTTTCTTGCTTTCGTGGTATTCTTGCTAGTCACACTAGCCATCCTTACTGCGCTTC 26228

EPI_ISL_6640916_omicron -TCTTTTTCTTGCTTTCGTGGTATTCTTGCTAGTTACACTAGCCATCCTTACTGCGCTTC 26281

MW494315.1 -TCTTTTTCTTGCTTTCGTGGTATTCTTGCTAGTTACACTAGCCATCCTTACTGCGCTTC 26356

KF686346.1 -TTTAGTTTTATTTTGTCTTATTTCTTTAATCTTTGTTGTTGCTTTTTTAGCAACTATTA 27564

NC_006213.1 -TATAGTTGCCATTTGTTTATTGGTTACAATAGTTGTAGTGGCATTTTTGGCAACTTTTA 28225

NC_002645.1 ATGTACTACTCTGGTGTGTGGTGCTTATAGTGATACTACTAGTGTGTATTACAATAATTA 24849

JX504050.1 ATTCCATTTTATGGCTCCTTGTTATGATATTTTTCTTTGTGTTGGCAATGACCTTTATTA 25299

* * * * * * * *

NC_019843.3 GATTATGTGTGCAATGTATGACAGGCTTCAATACCCTGTTAGTTCAGCCCGCATTATACT 27761

NC_004718.3 GATTGTGTGCGTACTGCTGCAATATTGTTAACGTGAGTTTAGTAAAACCAACGGTTTACG 26288

EPI_ISL_6640916_omicron GATTGTGTGCGTACTGCTGCAATATTGTTAACGTGAGTCTTGTAAAACCTTCTTTTTACG 26341

MW494315.1 GATTGTGTGCGTACTGCTGCAATATTGTTAACGTGAGTCTTGTAAAACCTTCTTTTTACG 26416

KF686346.1 AGCTTTGTATGCAACTTTGTGGTTTTTGTAATTTCTTTATTATTTCACCTTCGGCTTACG 27624

NC_006213.1 AATTGTGTATTCAACTTTGCGGTATGTGTAATACCTTAGTACTGTCCCCTTCTATTTATG 28285

NC_002645.1 AACTA---ATTAAGCTTTGTTTCACTTGCCATATGTTTTGTAATAGAACAGTTTATGGCC 24906

JX504050.1 AACTG---ATTCAATTGTGTTTTACTTGTCATTATTTTTTTAGTAGGACATTATATCAAC 25356

* * * *

NC_019843.3 TGTATAATACTGGACGTTCAGTCTATGTAAAATTCCAGGATAGTAAACCCCCTCTACCAC 27821

NC_004718.3 TCTACTCG------CGTGTTAAAAATCTGAACTCTTCTGAAGGAGTTCCTGATCTTC--- 26339

EPI_ISL_6640916_omicron TTTACTCT------CGTGTTAAAAATCTGAATTCTTCT---AGAGTTCCTGATCTTC--- 26389

MW494315.1 TTTACTCT------CGTGTTAAAAATCTGAATTCTTCT---AGAGTTCCTGATCTTC--- 26464

KF686346.1 TTTATAAAAGAGGTATGCAGTTGTATAAGTCTTATAGTGAACAAGTTATACCACCCACTT 27684

NC_006213.1 TGTTTAATAGAGGTAGGCAGTTTTATGAGTTTTACAATGATGTAAAACCACCAGTCCTTG 28345

NC_002645.1 CCATTAAAAATGTGTACCACATTTACCAATCATATATGCACATAGACCCTTTCCCTAAAC 24966

JX504050.1 CAGTTTATAAAATTTTTCTTGCTTACCAAGATTATATGCAAATAGCACCTGTTCCAGCTG 25416

* *

NC_019843.3 CTGACGAGTGGGTTTAACGAACTCCTTCATA----------------------------- 27852

NC_004718.3 --------TGGTCTAAACGAACTAACTATTA---TTATTATTCTGTTTGGAACTTTAACA 26388

EPI_ISL_6640916_omicron --------TGGTCTAAACGAACTAAATATTATATTAGTTTTTCTGTTTGGAACTTTAATT 26441

MW494315.1 --------TGGTCTAAACGAACTAAATATTATATTAGTTTTTCTGTTTGGAACTTTAATT 26516

KF686346.1 CAGATTATTTAATCTA---AATCTAAACATT----------------------------- 27712

NC_006213.1 ATGTGGATGACGTTTAGGTAATCCAAACATT----------------------------- 28376

NC_002645.1 GAGTTATTGATTTCTA---AACTAAACGACA----------------------------- 24994

JX504050.1 AAGTACTAAATGTCTA---AACTAAACGATG----------------------------- 25444

* **

NC_019843.3 ---------ATGTCTAATATGACGCAA------------------CTCACTGAGGCGCAG 27885

NC_004718.3 TTGCTTATCATGGCAGACAACGGTACT------------------ATTACCGTTGAGGAG 26430

EPI_ISL_6640916_omicron TTAGCCATGGCAGGTTCCAACGGTACT------------------ATTACCGTTGAAGAG 26483

MW494315.1 TTAGCCATGGCAGATTCCAACGGTACT------------------ATTACCGTTGAAGAG 26558

KF686346.1 ---------ATGAATAAATCTTTTTTCCCTCAA------------TTTACTTCTGATCAA 27751

NC_006213.1 ---------ATGAGTAGTAAAACTACACCAGCACCAGTTTATATCTGGACTGCTGATGAA 28427

NC_002645.1 ---------ATGTCAAATGACAATTGTACGGGT------------------------GAC 25021

JX504050.1 ---------TCTAATAGTAGTGTGCCTCTTTTA------------------------GAG 25471

*

NC_019843.3 ATTATTGCCATTATTAAAGACTGGAACTTTGCATGGTCCCTGATCTTTCTCTTAATTACT 27945

NC_004718.3 CTTAAACAACTCCTGGAACAATGGAACCTAGTAATAGGTTTCCTATTCCTAGCCTGGATT 26490

EPI_ISL_6640916_omicron CTTAAAAAGCTCCTTGAAGAATGGAACCTAGTAATAGGTTTCCTATTCCTTACATGGATT 26543

MW494315.1 CTTAAAAAGCTCCTTGAACAATGGAACCTAGTAATAGGTTTCCTATTCCTTACATGGATT 26618

KF686346.1 GCTGTTACATTCTTAAAAGAATGGAATTTCTCTTTGGGTGTAATACTACTTTTTATTACT 27811

NC_006213.1 GCTATTAAATTCCTAAAGGAATGGAATTTTTCTTTGGGTATTATACTACTTTTTATTACA 28487

NC_002645.1 ATTGTCACCCATTTGAAGAATTGGAATTTTGGTTGGAATGTTATTCTAACCATATTCATT 25081

JX504050.1 GTTTATGTCCATTTACGTAACTGGAACTTTAGTTGGAATTTAATTCTAACGCTTTTTATA 25531

* * * ***** * * * * *

NC_019843.3 ATCGTACTACAGTATGGATACCCATCCCGTAGTATGACTGTCTATGTCTTTAAAATGTTT 28005

NC_004718.3 ATGTTACTACAATTTGCCTATTCTAATCGGAACAGGTTTTTGTACATAATAAAGCTTGTT 26550

EPI_ISL_6640916_omicron TGTCTTCTACAATTTGCCTATGCCAACAGGAATAGGTTTTTGTATATAATTAAGTTAATT 26603

MW494315.1 TGTCTTCTACAATTTGCCTATGCCAACAGGAATAGGTTTTTGTATATAATTAAGTTAATT 26678

KF686346.1 ATCATATTGCAGTTCGGTTATACGAGCCGTAGTATGTTTGTTTATTTTATCAAGATGATT 27871

NC_006213.1 ATCATATTGCAATTTGGATATACAAGTCGCAGTATGTTTGTTTATGTTATTAAGATGATT 28547

NC_002645.1 GTTATTCTTCAGTTTGGACACTATAAATACTCCAGATTGTTTTATGGTTTGAAGATGCTT 25141

JX504050.1 GTTGTGTTGCAGTATGGGCATTATAAGTATAGCAGACTTCTTTATGGTTTAAAGATGTCT 25591

* * ** * * * * * ** * ** * *

NC_019843.3 GTTTTATGGCTCCTATGGCCATCTTCCATGGCGCTATCAATATTTAGCGCCGT------T 28059

NC_004718.3 TTCCTCTGGCTCTTGTGGCCAGTAACACTTGCTTGTTTTGTGCTTGCTGCTGT------C 26604

EPI_ISL_6640916_omicron TTCCTCTGGCTGTTATGGCCAGTAACTTTAACTTGTTTTGTGCTTGCTGCTGT------T 26657

MW494315.1 TTCCTCTGGCTGTTATGGCCAGTAACTTTAGCTTGTTTTGTGCTTGCTGCTGT------T 26732

KF686346.1 ATTCTTTGGCTTATGTGGCCATTGACTATCACCTTGACTATATTTAATTGCTT------T 27925

NC_006213.1 ATTTTGTGGCTTATGTGGCCCCTTACTATAATCTTAACTATTTTCAATTGCGT------A 28601

NC_002645.1 GTACTGTGGCTTCTTTGGCCACTCGTACTTGCTTTGTCAATCTTTGACACCTGGGCTAAT 25201

JX504050.1 GTTTTATGGTGTTTATGGCCACTTGTTCTAGCTTTGTCTATTTTTGACTGTTTTGTCAAT 25651

* * *** * ***** * * *

NC_019843.3 TATCCAATTGATCTAGCTTCCCAGATAATCTCTGGCATTGTAGCAGCTGTTTCAGCTATG 28119

NC_004718.3 TACAGAATTAATTGGGTGACTGGCGGGATTGCGATTGCAATGGCTTGTATTGTAGGCTTG 26664

EPI_ISL_6640916_omicron TACAGAATAAATTGGATCACCGGTGGAATTGCTATCGCAATGGCTTGTCTTGTAGGCTTG 26717

MW494315.1 TACAGAATAAATTGGATCACCGGTGGAATTGCTATCGCAATGGCTTGTCTTGTAGGCTTG 26792

KF686346.1 TATGCTCTGAATAATGCTTTTCTTGCATTTTCTATAGTGTTTACTATTATTTCTATTGTT 27985

NC_006213.1 TACGCATTGAATAATGTGTATCTTGGCCTTTCTATAGTTTTTACCATAGTGGCCATTATT 28661

NC_002645.1 TGGGATTCTAATTGGGCCTTTGTTGCATTTAGCTTTTTTATGGCCGTATCAACACTCGTT 25261

JX504050.1 TTTAATGTGGACTGGGTCTTTTTTGGTTTTAGTATTCTTATGTCTATTATTACACTTTGT 25711

* * * * *

NC_019843.3 ATGTGGATTTCCTACTTTGTGCAGAGTATCCGGCTGTTTATGAGAACTGGATCATGGTGG 28179

NC_004718.3 ATGTGGCTTAGCTACTTCGTTGCTTCCTTCAGGCTGTTTGCTCGTACCCGCTCAATGTGG 26724

EPI_ISL_6640916_omicron ATGTGGCTCAGCTACTTCATTGCTTCTTTCAGACTGTTTGCGCGTACGCGTTCCATGTGG 26777

MW494315.1 ATGTGGCTCAGCTACTTCATTGCTTCTTTCAGACTGTTTGCGCGTACGCGTTCCATGTGG 26852

KF686346.1 ATATGGATTCTTTATTTTGTTAATAGTATTCGGCTTTTTATTAGAACTGGCAGTTGGTGG 28045

NC_006213.1 ATGTGGATTGTGTATTTTGTGAATAGTATCAGGTTGTTTATTAGAACTGGAAGTTTTTGG 28721

NC_002645.1 ATGTGGGTGATGTACTTCGCAAACAGTTTCAGACTTTTCCGACGTGCTCGAACTTTTTGG 25321

JX504050.1 TTATGGGTTATGTATTTTGTTAATAGTTTCAGACTTTGGCGCCGTGTTAAAACTTTTTGG 25771

* *** * ** ** * * * * * ***

NC_019843.3 TCATTCAATCCTGAGACTAATTGCCTTTTGAACGTTCCATTTGGTGGTACAACTGTCGTA 28239

NC_004718.3 TCATTCAACCCAGAAACAAACATTCTTCTCAATGTGCCTCTCCGGGGGACAATTGTGACC 26784

EPI_ISL_6640916_omicron TCATTCAATCCAGAAACTAACATTCTTCTCAACGTGCCACTCCATGGCACTATTCTGACC 26837

MW494315.1 TCATTCAATCCAGAAACTAACATTCTTCTCAACGTGCCACTCCATGGCACTATTCTGACC 26912

KF686346.1 AGTTTTAATCCAGAGACCAATAATCTTATGTGTATTGATATGAAAGGCAAGATGTTTGTT 28105

NC_006213.1 AGTTTCAACCCAGAAACAAACAACTTGATGTGTATAGATATGAAAGGAACAATGTATGTT 28781

NC_002645.1 GCATGGAATCCTGAGGTTAATGCAATCACTGTCACAACCGTGTTGGGACAGACATACTAT 25381

JX504050.1 GCTTTTAATCCTGAAACTAATGCAATCATCTCTCTCCAGGTTTACGGACATAATTATTAC 25831

* ** ** ** ** * * ** *

NC_019843.3 CGTCCACTCGTAGAGGACTCTACCAGTGTAACTGCTGTTGTAACCAATGGCCACCTCAAA 28299

NC_004718.3 AGACCGCTCATGGAAAGTGAACTTGTCATTGGTGCTGTGATCATTCGTGGTCACTTGCGA 26844

EPI_ISL_6640916_omicron AGACCGCTTCTAGAAAGTGAACTCGTAATCGGAGCTGTGATCCTTCGTGGACATCTTCGT 26897

MW494315.1 AGACCGCTTCTAGAAAGTGAACTCGTAATCGGAGCTGTGATCCTTCGTGGACATCTTCGT 26972

KF686346.1 AGGCCAGTTATTGAGGACTATCACACATTAACTGCTACTGTTATTCGTGGTCATCTTTAT 28165

NC_006213.1 AGGCCGATAATTGAGGACTATCATACTCTGACGGTCACAATAATACGCGGCCATCTTTAC 28841

NC_002645.1 CAACCCATTCAACAAGCTCCAACAGGCATTACTGTGACCTTGCTGAGCGGCGTGCTTTAC 25441

JX504050.1 TTACCGGTGATGGCTGCACCTACAGGTGTTACATTAACACTTCTTAGTGGTGTACTTCTT 25891

** * * * ** *

NC_019843.3 ATGGCTGGCATGCATTTCGGTGCTTGTGACT---ACGACAGACTTCCTAATGAAGTCACC 28356

NC_004718.3 ATGGCCGGACACTCCCTAGGGCGCTGTGACA---TTAAGGACCTGCCAAAAGAGATCACT 26901

EPI_ISL_6640916_omicron ATTGCTGGACACCATCTAGGACGCTGTGACA---TCAAGGACCTGCCTAAAGAAATCACT 26954

MW494315.1 ATTGCTGGACACCATCTAGGACGCTGTGACA---TCAAGGACCTGCCTAAAGAAATCACT 27029

KF686346.1 ATACAGGGTGTCAAACTTGGCACTGGTTATACTCTTTCAGATTTGCCCGTATATGTTACT 28225

NC_006213.1 ATTCAAGGTATAAAACTAGGTACTGGCTATTCTTTGGCAGATTTGCCAGCTTATATGACT 28901

NC_002645.1 GTTGACGGACATAGATTGGCTTCAGGTGTTCAGGTTCATAACCTACCTGAATACATGACA 25501

JX504050.1 GTTGATGGCCATAAGATTGCTACTCGTGTTCAAGTGGGTCAGTTGCCTAAATATGTAATA 25951

* ** * * * * ** * * *

NC_019843.3 GTGGCCAAACCCAATGTGCTGATTGCTTTAAAAATGGTGAAGC---GGCAAAGCTACGGA 28413

NC_004718.3 GTGGCTACATCACGAACGCTTTCTTATTACAAATTAGGAGCGT---CGCAGCGTGTAGGC 26958

EPI_ISL_6640916_omicron GTTGCTACATCACGAACGCTTTCTTATTACAAATTGGGAGCTT---CGCAGCGTGTAGCA 27011

MW494315.1 GTTGCTACATCACGAACGCTTTCTTATTACAAATTGGGAGCTT---CGCAGCGTGTAGCA 27086

KF686346.1 GTAGCTAAGGTGCAAGTACTTTGTACCTATAAACGTGCCTTTT---TAGATAAGTTAGAT 28282

NC_006213.1 GTTGCTAAGGTTACACACCTGTGCACATATAAGCGTGGTTTTC---TTGACAGGATAAGC 28958

NC_002645.1 GTTGCCGTGCCGAGCACTACTATAATTTATAGTAGAGTCGGAAGGTCCGTAAATTCACAA 25561

JX504050.1 GTTGCTACGCCTAGTACCACAATTGTTTGTGACCGTGTTGGTCGCTCTGTTAATGAAACA 26011

** ** * *

NC_019843.3 ACTAATTCCGGCGTTGCCATTTACCATAGATATAAGGCAGGTAATTACAG---------- 28463

NC_004718.3 ACTGATTCAGGTTTTGCTGCATACAACCGCTACCGTATTGGAAACTATAAATTAAATACA 27018

EPI_ISL_6640916_omicron GGTGACTCAGGTTTTGCTGCATACAGTCGCTACAGGATTGGCAACTATAAATTAAACACA 27071

MW494315.1 GGTGACTCAGGTTTTGCTGCATACAGTCGCTACAGGATTGGCAACTATAAATTAAACACA 27146

KF686346.1 GTTAATAGTGGTTTTGCTGTTTTTGTTAAGTCTAAAGTTGGTAACTATCGTTTACCGTCT 28342

NC_006213.1 GATACTAGTGGTTTTGCTGTTTATGTTAAGTCCAAAGTCGGTAATTACCGACTGCCATCA 29018

NC_002645.1 AATAGCACAGGCTGGGTTTTCTACGTACGAGTAAAACACGGTGATTTTTCTGCAGTGAGC 25621

JX504050.1 AGCCAGACTGGTTGGGCATTCTACGTCCGTGCTAAACATGGTGATTTTTCTGGTGTTGCC 26071

** * * ** * *

NC_019843.3 ------------------------------------------------------------ 28463

NC_004718.3 GACCACGCCGGTAGCAACGACAATATTGCTTTGCTAGTACAGTAAGTGACAACAGATGTT 27078

EPI_ISL_6640916_omicron GACCATTCCAGTAGCAGTGACAATATTGCTTTGCTTGTACAGTAAGTGACAACAGATGTT 27131

MW494315.1 GACCATTCCAGTAGCAGTGACAATATTGCTTTGCTTGTACAGTAAGTGACAACAGATGTT 27206

KF686346.1 AGTAAACCTAG---TGGTATGGATACTGCCTTGTTAAGAGCTTAAATCTAAACTAT---- 28395

NC_006213.1 ACCCAAAAGGGTTCTGGCATGGACACCGCATTGTTGAGAAATAATATCTAAATTTT---- 29074

NC_002645.1 TCTCCCATGAGCAACATGACAGAAAACGAAAGATTGCTTCATTTTTTCTAAACTG----- 25676

JX504050.1 TCTCAGGAGGGTGTTTTGTCAGAAAGAGAGAAGTTGCTTCATTTAATCTAAACTA----- 26126

NC_019843.3 ------------------------------------------------------------ 28463

NC_004718.3 TCATCTTGTTGACTTCCAGGTTACAATAGCAGAGATATTGATTATCATTATGAGGACTTT 27138

EPI_ISL_6640916_omicron TCATCTCGTTGACTTTCAGGTTACTATAGCAGAGATATTACTAATTATTATGCGGACTTT 27191

MW494315.1 TCATCTCGTTGACTTTCAGGTTACTATAGCAGAGATATTACTAATTATTATGAGGACTTT 27266

KF686346.1 ------------------------------------------------------------ 28395

NC_006213.1 ------------------------------------------------------------ 29074

NC_002645.1 ------------------------------------------------------------ 25676

JX504050.1 ------------------------------------------------------------ 26126

NC_019843.3 ------------------------------------------------------GAGTCC 28469

NC_004718.3 CAGGATTGCTATTTGGAATCTTGACGTTATAATAAGTTCAATAGTGAGACAATTATTTAA 27198

EPI_ISL_6640916_omicron TAAAGTTTCCATTTGGAATCTTGATTACATCATAAACCTCATAATTAAAAATTTATCTAA 27251

MW494315.1 TAAAGTTTCCATTTGGAATCTTGATTACATCATAAACCTCATAATTAAAAATTTATCTAA 27326

KF686346.1 ------------------------------------------------------TAGGAT 28401

NC_006213.1 ------------------------------------------------------AAGGAT 29080

NC_002645.1 ------------------------------------------------------------ 25676

JX504050.1 ------------------------------------------------------------ 26126

NC_019843.3 GCCTATTACGGCGGATATTGAACTTGCATTGCTTCGAGCTTAGGCTCTTTAGTAAGAGT- 28528

NC_004718.3 GCCTCTAACTAAGAAGAATTATTCGGAGTTAGATGATGAAGAACCTATGGAGTTAGATTA 27258

EPI_ISL_6640916_omicron GTCACTAACTGAGAATAAATATTCTCAATTAGATGAAGAGCAACCAATGGAGATTGATT- 27310

MW494315.1 GTCACTAACTGAGAATAAATATTCTCAATTAGATGAAGAGCAACCAATGGAGATTGATT- 27385

KF686346.1 GTCTTATACTCCCGGTCATTATGC-----TGGAAGTAGAAGCTCCTCTGGAAATCGTTC- 28455

NC_006213.1 GTCTTTTACTCCTGGTAAGCAATC-----CAGTAGTAGAGCGTCCTCTGGAAATCGTTC- 29134

NC_002645.1 ------------------------------------------------------------ 25676

JX504050.1 ------------------------------------------------------------ 26126

NC_019843.3 ------------------------------------------------------------ 28528

NC_004718.3 TCCATAAAACGAACATGAAAATTATTCTCTTCCTGACATTGATTGTATTTACATCTTGCG 27318

EPI_ISL_6640916_omicron ------AAACGAACATGAAAATTATTCTTTTCTTGGCACTGATAACACTCGCTACTTGTG 27364

MW494315.1 ------AAACGAACATGAAAATTATTCTTTTCTTGGCACTGATAACACTCGCTACTTGTG 27439

KF686346.1 ------------------------------------------------------------ 28455

NC_006213.1 ------------------------------------------------------------ 29134

NC_002645.1 ------------------------------------------------------------ 25676

JX504050.1 ------------------------------------------------------------ 26126

NC_019843.3 ------------------------------------------------------------ 28528

NC_004718.3 AGCTATATCACTATCAGGAGTGTGTTAGAGGTACGACTGTACTACTAAAAGAACCTTGCC 27378

EPI_ISL_6640916_omicron AGCTTTATCACTACCAAGAGTGTGTTAGAGGTACAACAGTACTTTTAAAAGAACCTTGCT 27424

MW494315.1 AGCTTTATCACTACCAAGAGTGTGTTAGAGGTACAACAGTACTTTTAAAAGAACCTTGCT 27499

KF686346.1 ------------------------------------------------------------ 28455

NC_006213.1 ------------------------------------------------------------ 29134

NC_002645.1 ------------------------------------------------------------ 25676

JX504050.1 ------------------------------------------------------------ 26126

NC_019843.3 ------------------------------------------------------------ 28528

NC_004718.3 CATCAGGAACATACGAGGGCAATTCACCATTTCACCCTCTTGCTGACAATAAATTTGCAC 27438

EPI_ISL_6640916_omicron CTTCTGGAACATACGAGGGCAATTCACCATTTCATCCTCTAGCTGATAACAAATTTGCAC 27484

MW494315.1 CTTCTGGAACATACGAGGGCAATTCACCATTTCATCCTCTAGCTGATAACAAATTTGCAC 27559

KF686346.1 ------------------------------------------------------------ 28455

NC_006213.1 ------------------------------------------------------------ 29134

NC_002645.1 ------------------------------------------------------------ 25676

JX504050.1 ------------------------------------------------------------ 26126

NC_019843.3 ------------------------------------------------------------ 28528

NC_004718.3 TAACTTGCACTAGCACACACTTTGCTTTTGCTTGTGCTGACGGTACTCGACATACCTATC 27498

EPI_ISL_6640916_omicron TGACTTGCTTTAGCACTCAATTTGCTTTTGCTTGTCCTGACGGCGTAAAACACGTCTATC 27544

MW494315.1 TGACTTGCTTTAGCACTCAATTTGCTTTTGCTTGTCCTGACGGCGTAAAACACGTCTATC 27619

KF686346.1 ------------------------------------------------------------ 28455

NC_006213.1 ------------------------------------------------------------ 29134

NC_002645.1 ------------------------------------------------------------ 25676

JX504050.1 ------------------------------------------------------------ 26126

NC_019843.3 ------------------------------------------------------------ 28528

NC_004718.3 AGCTGCGTGCAAGATCAGTTTCACCAAAACTTTTCATCAGACAAGAGGAGGTTCAACAAG 27558

EPI_ISL_6640916_omicron AGTTACGTGCCAGATCAGTTTCACCTAAACTGTTCATCAGACAAGAGGAAGTT---CAAG 27601

MW494315.1 AGTTACGTGCCAGATCAGTTTCACCTAAACTGTTCATCAGACAAGAGGAAGTT---CAAG 27676

KF686346.1 ------------------------------------------------------------ 28455

NC_006213.1 ------------------------------------------------------------ 29134

NC_002645.1 ------------------------------------------------------------ 25676

JX504050.1 ------------------------------------------------------------ 26126

NC_019843.3 ------------------------------------------------------------ 28528

NC_004718.3 AGCTCTACTCGCCACTTTTTCTCATTGTTGCTGCTCTAGTATTTTTAATACTTTGCTTCA 27618

EPI_ISL_6640916_omicron AACTTTACTCTCCAATTTTTCTTATTGTTGCGGCAATAGTGTTTATAACACTTTGCTTCA 27661

MW494315.1 AACTTTACTCTCCAATTTTTCTTATTGTTGCGGCAATAGTGTTTATAACACTTTGCTTCA 27736

KF686346.1 ------------------------------------------------------------ 28455

NC_006213.1 ------------------------------------------------------------ 29134

NC_002645.1 ------------------------------------------------------------ 25676

JX504050.1 ------------------------------------------------------------ 26126

NC_019843.3 ------------------------------------------------------------ 28528

NC_004718.3 CCATTAAGAGAAAGACAGAATGAATGAGCTCACTTTAATTGACTTCTATTTGTGCTTTTT 27678

EPI_ISL_6640916_omicron CACTCAAAAGAAAGACAGAATGATTGAACTTTCATTAATTGACTTCTATTTGTGCTTTTT 27721

MW494315.1 CACTCAAAAGAAAGACAGAATGATTGAACTTTCATTAATTGACTTCTATTTGTGCTTTTT 27796

KF686346.1 ------------------------------------------------------------ 28455

NC_006213.1 ------------------------------------------------------------ 29134

NC_002645.1 ------------------------------------------------------------ 25676

JX504050.1 ------------------------------------------------------------ 26126

NC_019843.3 ------------------------------------------------------------ 28528

NC_004718.3 AGCCTTTCTGCTATTCCTTGTTTTAATAATGCTTATTATATTTTGGTTTTCACTCGAAAT 27738

EPI_ISL_6640916_omicron AGCCTTTCTGTTATTCCTTGTTTTAATTATGCTTATTATCTTTTGGTTCTCACTTGAACT 27781

MW494315.1 AGCCTTTCTGCTATTCCTTGTTTTAATTATGCTTATTATCTTTTGGTTCTCACTTGAACT 27856

KF686346.1 ------------------------------------------------------------ 28455

NC_006213.1 ------------------------------------------------------------ 29134

NC_002645.1 ------------------------------------------------------------ 25676

JX504050.1 ------------------------------------------------------------ 26126

NC_019843.3 ------------------------------------------------------------ 28528

NC_004718.3 CCAGGATCTAGAAGAACCTTGTACCAAAGTCTAAACGAACATGAAACTTCTCATTGTTTT 27798

EPI_ISL_6640916_omicron GCAAGATCATAATGAANNNNNNNNN---NNNNNNNNNNNNNNNNNNNNNNNNNNNNNNNN 27838

MW494315.1 GCAAGATCATAATGAAACTTGTCAC---GCCTAAACGAACATGAAATTTCTTGTTTTCTT 27913

KF686346.1 ------------------------------------------------------------ 28455

NC_006213.1 ------------------------------------------------------------ 29134

NC_002645.1 ------------------------------------------------------------ 25676

JX504050.1 ------------------------------------------------------------ 26126

NC_019843.3 ------------------------------------------------------------ 28528

NC_004718.3 GACTTGTATTTCTCTATGCAGTTGCATATGCACTGTAG-------TACAGCGCTGTGCAT 27851

EPI_ISL_6640916_omicron NNNNNNNNNNNN-NNNNNNNNNNNNNNNNNNNNNNNNNNNNNNNNNNNNNNNNNNNNNNN 27897

MW494315.1 AGGAATCATCAC-AACTGTAGCTGCATTTCACCAAGAATGTAGTTTACAGTCATGTACTC 27972

KF686346.1 ------------------------------------------------------------ 28455

NC_006213.1 ------------------------------------------------------------ 29134

NC_002645.1 ------------------------------------------------------------ 25676

JX504050.1 ------------------------------------------------------------ 26126

NC_019843.3 ------------------------------------------------------------ 28528

NC_004718.3 CTAATAAACCTCATGTGCTTGAAGATCCTTGTAAGGTACAACACTAGGGGTAATACTTAT 27911

EPI_ISL_6640916_omicron NNNNNNNNNNNNNNNNNNNNNNNNNNNNNNNNNNNNNNNNNNNNNN-------------- 27943

MW494315.1 AACATCAACCATATGTAGTTGATGACCCGTGTCCTATTCACTTCTA-------------- 28018

KF686346.1 ------------------------------------------------------------ 28455

NC_006213.1 ------------------------------------------------------------ 29134

NC_002645.1 ------------------------------------------------------------ 25676

JX504050.1 ------------------------------------------------------------ 26126

NC_019843.3 ------------------------------------------------------------ 28528

NC_004718.3 AGCACTGCTTGGCTTTGTGCTCTAGGAAAGGTTTTACCTTTTCATAGATGGCACACTATG 27971

EPI_ISL_6640916_omicron -----------------------------------------NNNNNNNNNNNNNNNNNNN 27962

MW494315.1 -----------------------------------------TTCTAAATGGTATATTAGA 28037

KF686346.1 ------------------------------------------------------------ 28455

NC_006213.1 ------------------------------------------------------------ 29134

NC_002645.1 ------------------------------------------------------------ 25676

JX504050.1 ------------------------------------------------------------ 26126

NC_019843.3 ------------------------------------------------------------ 28528

NC_004718.3 GTTCAAACATGCACACC------------------------------------------- 27988

EPI_ISL_6640916_omicron NNNNNNNNNNNNNNNNNNNNNNNNNNNNNNNNNNNNNNNNNNNNNNNNNNNNNNNNNNNN 28022

MW494315.1 GTAGGAGCTAGAAAATCAGCACCTTTAATTGAATTGTGCGTGGATGAGGCTGGTTCTAAA 28097

KF686346.1 ------------------------------------------------------------ 28455

NC_006213.1 ------------------------------------------------------------ 29134

NC_002645.1 ------------------------------------------------------------ 25676

JX504050.1 ------------------------------------------------------------ 26126

NC_019843.3 ------------------------------------------------------------ 28528

NC_004718.3 -----------------------------------------------TAATGTTACTATC 28001

EPI_ISL_6640916_omicron NNNNNNNNNNNNNNNNNNNNNNNNNNNNNNNNNNNNNNNNNNNNNNNNNNNNNNNNNNNN 28082

MW494315.1 TCACCCATTCAGTACATCGATATCGGTAATTATACAGTTTCCTGTTTACCTTTTACAATT 28157

KF686346.1 ------------------------------------------------------------ 28455

NC_006213.1 ------------------------------------------------------------ 29134

NC_002645.1 ------------------------------------------------------------ 25676

JX504050.1 ------------------------------------------------------------ 26126

NC_019843.3 ------------------------------------------------------------ 28528

NC_004718.3 AACTGTCAAGATCCAGCTGGTGGTGCGCTTATAGCTAGGTGTTGGTACCTTCATGAAGGT 28061

EPI_ISL_6640916_omicron NNNNNNNNNNNNNNNNNNNNNNNNNNNNNNNNNNNNNNNNNNNNNNNNNNNNNNNNNNNN 28142

MW494315.1 AATTGCCAGGAACCTAAATTGGGTAGTCTTGTAGTGCGTTGTTCGTTCTATGAAGACTTT 28217

KF686346.1 ------------------------------------------------------------ 28455

NC_006213.1 ------------------------------------------------------------ 29134

NC_002645.1 ------------------------------------------------------------ 25676

JX504050.1 ------------------------------------------------------------ 26126

NC_019843.3 ----------------------------------------------------ATCTTAAT 28536

NC_004718.3 CACCAAACTGCTGCATTTAGAGACGTACTTGTTGTTTTAAATAAACGAACAAATTAAAAT 28121

EPI_ISL_6640916_omicron NNNNNNNNNNNNNNNNNNNNNNNNNNNNNNN--NNNNNNTCTAAACGAACAAACTTAAAT 28200

MW494315.1 TTAGAGTATCATGACGTTCGTGTTGTTTTAG--ATTTCATCTAAACGAACAAACTAAAAT 28275

KF686346.1 ----------------------------------------------------------AG 28457

NC_006213.1 ----------------------------------------------------TGGTAATG 29142

NC_002645.1 ------------------------------------------------------------ 25676

JX504050.1 ------------------------------------------------------------ 26126

NC_019843.3 TGATTTTAACGAATCTCAATTTCATTGTTATGGCATCCCCTGCTGCACCTCGTGCTGTTT 28596

NC_004718.3 GTCTGATAATGGACCCCAATCAAACCAACGTAGTGCCCCCCGCATTACATT-TGGTGGAC 28180

EPI_ISL_6640916_omicron GTCTGATAATGGACCCCA---AAATCAGCGAAATGCACTCCGCATTACGTT-TGGTGGAC 28256

MW494315.1 GTCTGATAATGGACCCCA---AAATCAGCGAAATGCACCCCGCATTACGTT-TGGTGGAC 28331

KF686346.1 GAATCCTCAAGAAAACTT------------------------------CTTGGGCTGACC 28487

NC_006213.1 GCATCCTCAAGTGGGCCG------------------------------ATCAGTCCGACC 29172

NC_002645.1 ------------------------------------------------------------ 25676

JX504050.1 ------------------------------------------------------------ 26126

NC_019843.3 CCTTTGCCGATAACAATGATATA------------ACAAATACAAACCTATCTCGAGGTA 28644

NC_004718.3 CCACAGATTCAACTGACAATAAC------------CAGAATGGAGGACGCAATGGGGCAA 28228

EPI_ISL_6640916_omicron CCTCAGATTCAACTGGCAGTAAC------------CAGAATG---------GTGGGGCGC 28295

MW494315.1 CCTCAGATTCAACTGGCAGTAAC------------CAGAATGGAGAACGCAGTGGGGCGC 28379

KF686346.1 AATCTGAGCGAAATTACCAAACCTTTAATAGAGGCAGAAAAACCCAACCTAAATTCACTG 28547

NC_006213.1 AGTTT---AGAAATGTTCAAACC------AGGGGTAGAAGAGCTCAACCCAAGCAAACTG 29223

NC_002645.1 -----------------------------AACGAAAAGATGGCTACAGTCAAATGGGCTG 25707

JX504050.1 -----------------------------AAC---AAAATGGCTAGTGTAAATTGGGCCG 26154

*

NC_019843.3 GAGGACGTAATCCAAAACCACGAGCTGCACCAAATAACACTGTCTCTTGGTACACTGGGC 28704

NC_004718.3 GGCCAAAACAGCGCCGACCCCAAGGTTTACCCAATAATACTGCGTCTTGGTTCACAGCTC 28288

EPI_ISL_6640916_omicron GATCAAAACAACGTCGGCCCCAAGGTTTACCCAATAATACTGCGTCTTGGTTCACCGCTC 28355

MW494315.1 GATCAAAACAACGTCGGCCCCAAGGTTTACCCAATAATACTGCGTCTTGGTTCACCGCTC 28439

KF686346.1 TGTCTACTCAACCACA------AGGAAATACTATCCCACATTATTCCTGGTTCTCCGGGA 28601

NC_006213.1 CTACCTCTCAGCAACCATCAGGAGGGAATGTTGTACCCTACTATTCTTGGTTCTCTGGAA 29283

NC_002645.1 ATGCATCTGAACCACAACGTGGTCGTCAGGGTAGAATACCTTATTCTCTTTATAGCCCTT 25767

JX504050.1 ATG---------ACAGAGCTGCTAGGAAGAAATTTCCTCCTCCTTCATTTTACATGCCTC 26205

** *

NC_019843.3 TTACCCAACACGGGAAAGT---CCCTCTTACCTTTCCACCTGGGCAGGGTGTACCTCTTA 28761

NC_004718.3 TCACTCAGCATGGCAAGGA---GGAACTTAGATTCCCTCGAGGCCAGGGCGTTCCAATCA 28345

EPI_ISL_6640916_omicron TCACTCAACATGGCAAGGA---AGACCTTAAATTCCCTCGAGGACAAGGCGTTCCAATTA 28412

MW494315.1 TCACTCAACATGGCAAGGA---AGACCTTAAATTCCCTCGAGGACAAGGCGTTCCAATTA 28496

KF686346.1 TCACTCAATTTCAAAAAGGTAGAGACTTTAAATTTTCAGATGGTCAAGGAGTTCCCATTG 28661

NC_006213.1 TTACTCAGTTTCAAAAGGGAAAGGAGTTTGAGTTTGTAGAAGGACAAGGTGTGCCTATTG 29343

NC_002645.1 TGCTTGTTGATAGTGAACA---ACCTTGGAAGGTGATACCTCGTAATTTGGTACCCATCA 25824

JX504050.1 TTTTGGTTAGTTCTGATAAGGCACCATATAGGGTCATTCCCAGGAATCTTGTCCCTATTG 26265

* * * * * ** ** *

NC_019843.3 ATGCCAATTCTACCCCTGCGCAAAATGCTGGGTATTGGCGGAGACAGGACAGAAAAA--- 28818

NC_004718.3 ACACCAATAGTGGTCCAGATGACCAAATTGGCTACTACCGAAGAGCTACCCGACGAG--- 28402

EPI_ISL_6640916_omicron ACACCAATAGCAGTCCAGATGACCAAATTGGCTACTACCGAAGAGCTACCAGACGAA--- 28469

MW494315.1 ACACCAATAGCAGTCCAGATGACCAAATTGGCTACTACCGAAGAGCTACCAGACGAA--- 28553
[truncated: 59,660 more chars]
